# Supplementary material for: Organomediated electrochemical fluorosulfonylation of aryl triflates via selective C–O bond cleavage
Source: Nat Commun. 2023 Oct 31;14:6933. doi: 10.1038/s41467-023-42699-0 (PMC10618246; doi:10.1038/s41467-023-42699-0)
Supplement: Supplementary file 1 — Supplementary Information [file 41467_2023_42699_MOESM1_ESM.pdf]

Supplementary Information

Organomediated Electrochemical Fluorosulfonylation of Aryl Triflates via Selective C–O Bond Cleavage

Xianqiang Kong[1]*, Yiyi Chen[1], Xiaohui Chen[1], Cheng Ma[2], Ming Chen[3], Wei Wang[1], Yuan-Qing Xu[4], Shao-Fei Ni[2]*, Zhong-Yan Cao[4]*

[1] School of Chemical Engineering and Materials, Changzhou Institute of Technology, No. 666 Liaohe Road, Changzhou, 213032, China

[2] Department of Chemistry, Shantou University, Shantou 515063, Guangdong, China

[3] Jiangsu Key Laboratory of Advanced Catalytic Materials & Technology, School of Petrochemical Engineering, Changzhou University, 21 Gehu Road 213164, Changzhou, China

[4] College of Chemistry and Molecular Sciences, Henan University, Kaifeng 475004, China

E-mail: kongxq@czu.cn; sfni@stu.edu.cn; zycao@henu.edu.cn

Table of content	Page
**1. General Information**	**S3**
**2. General Procedures for the Synthesis of Aryl Triflates**	**S4**
**3. General Procedure for Fluorosulfonylation**	**S4**
**4. One-Pot and Gram Scale Reaction**	**S19**
**5. Procedure for the Synthesis of 1-((2-Bromophenyl)sulfonyl)pyrrolidine**	**S20**
**6. Fluorescence Spectroscopy of 7 and 8**	**S20**
**7. Mechanistic Studies**	**S21**
**8. Cyclic Voltammetric (CV) Analysis**	**S23**
**9. Unsuccessful Substrates**	**S24**
**10. DFT Calculations**	**S25**
**11. Copies of NMR Spectra**	**S26**
**12. References**	**S171**

1. General Information

Commercial reagents and solvents were obtained from the commercial providers and used without further purification. The products were purified using a commercial flash chromatography system or a regular glass column. TLC was developed on silica gel 60 F254 glassplates. ^{1}H NMR (500 MHz or 400 MHz), ^{13}C NMR (126 MHz or 101 MHz) and ^{19}F NMR (471 MHz) spectra were recorded on a Bruker NMR apparatus. The chemical shifts are reported in δ (ppm) values (^{1}H and ^{13}C NMR relative to $CHCl_3$, δ 7.26 ppm for ^{1}H NMR and δ 77.0 ppm for ^{13}C NMR). Or alternatively, ^{1}H NMR chemical shifts were referenced to tetramethylsilane signal (0 ppm). Multiplicities are recorded by s (singlet), d (doublet), t (triplet), q (quartet), p (pentet), h (hextet), m (multiplet), dd (doublet of doublets), dt (doublet of triplets), td (triplet of doublets), tq (triplet of quartets), ddd (doublet of doublet of doublets), and br (broad). Coupling constants (*J*), are reported in Hertz (Hz). Commercial reagents were used without any further purification. Photoluminescence and excitation spectra were recorded on a FS 5 spectrofluorometer. Absolute fluorescence quantum yields were measured on a Fluormax-4p spectrometer.

2. General Procedure for the Synthesis of Aryl Triflates

The phenol (4 mmol) was dissolved in DCM (40 mL) and cooled to 0 ℃. NEt_3 (1.1 mL, 8.0 mmol, 2 equiv) was added dropwise to the solution, which was followed by the addition of triflic anhydride (733 μL, 4.4 mmol, 1.1 equiv). After 5 mins, the ice bath was removed, and the reaction was monitored by TLC. Once the phenol was completely consumed, the reaction was stopped. The solvent was evaporated under vacuum and the residue was purified by flash column chromatography to get the desired aryl triflate.[1-2]

3. General Procedure for Fluorosulfonylation

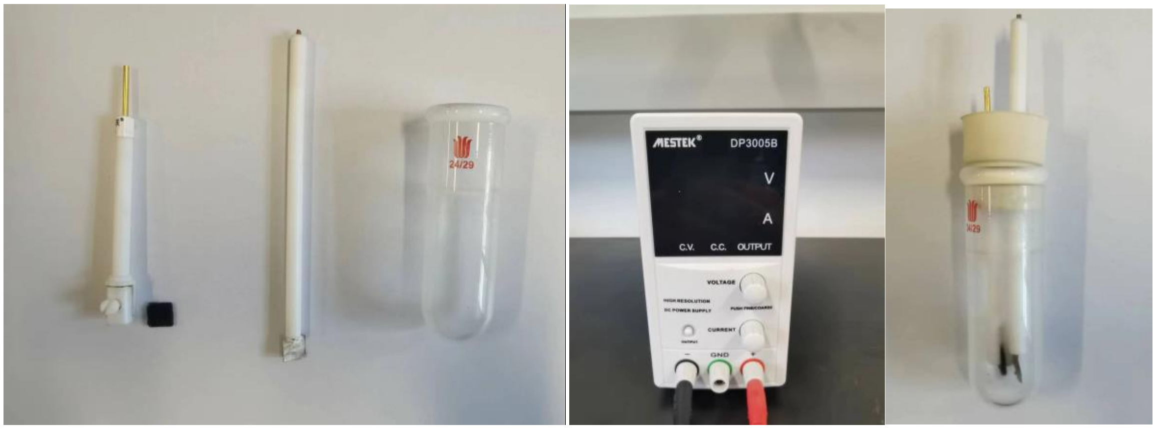

Supplementary Fig. 1 Electrolysis setup. Left）The reticulated vitreous carbon (RVC) (size: 10 mm × 10 mm × 2 mm, 100 PPi, Bought from Beijing Jingke Scientific Instrument Co., Ltd, China), Pt plate (10 mm × 10 mm × 1 mm); Middle) Constant potential rectifier was used as the power sources for our reaction (Bought from Shenzhen Maxtec Electronics Co., Ltd China); Right) Reaction tube used for the electrochemical transformation.

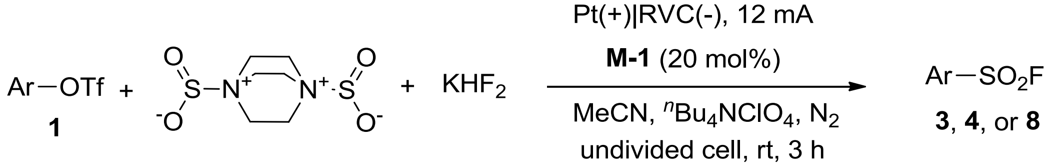

To the cell was added aryl triflate **1** (0.2 mmol), DABSO (36 mg, 0.15 mmol), KHF_2 (46.8 mg, 0.6 mmol), *n*-Bu_4NClO_4 (0.05 M, 68.2 mg), 9, 10-dicyanoanthracene (**M-1**, 9.12 mg, 0.04 mmol), CH_3CN (4 mL). The tube was installed with a Pt plate (1.0 × 1.0 cm^2) as the cathode and reticulated vitreous carbon (RVC) (1.0 × 1.0 cm^2) as the anode. The mixture was electrolyzed using 12 mA at room temperature under magnetic stirring. The reaction mixture was poured into ethyl acetate (40 mL), washed with water two times (10 mL × 2), dried over Na_2SO_4, and concentrated in vacuo. The residue was purified by column chromatography on silica gel using a mixture of petroleum ether/EtOAc as eluent to afford the desired pure product **3**, **4**, or **8**. Before the next use, the electrodes were washed with CH_2Cl_2, acetone and EtOH three times.

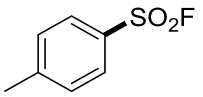

3a

4-Methylbenzenesulfonyl fluoride (**3a**),[3] 26.1 mg, 75% yield. ^{1}H NMR (500 MHz, $CDCl_3$) δ 7.89 (dd, *J* = 8.4, 1.7 Hz, 2H), 7.42 (d, *J* = 8.1 Hz, 2H), 2.49 (s, 3H). ^{13}C NMR (126 MHz, $CDCl_3$) δ 147.1, 130.3, 130.1 (d, *J* = 19 Hz), 128.4, 21.8. ^{19}F NMR (376 MHz, Chloroform-d) δ 66.30. The analytical data are consistent with literature values.

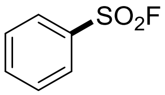

3b

Benzenesulfonyl fluoride (**3b**),[3] 26.1 mg, 71% yield. ^{1}H NMR (400 MHz, $CDCl_3$) δ 8.09 – 7.96 (m, 2H), 7.82 – 7.72 (m, 1H), 7.64 (td, *J* = 8.0, 1.1 Hz, 2H). ^{13}C NMR (126 MHz, $CDCl_3$) δ 135.65, 133.0 (d, *J* = 19 Hz), 129.72, 128.39. ^{19}F NMR (376 MHz, $CDCl_3$) δ 65.87.

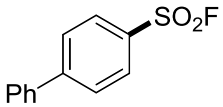

3c

[1,1'-Biphenyl]-4-sulfonyl fluoride (**3c**),[3] 38.2 mg, 81% yield. ^{1}H NMR (500 MHz, $CDCl_3$) δ 8.10 – 8.01 (m, 2H), 7.85 – 7.73 (m, 2H), 7.67 – 7.59 (m, 2H), 7.55 – 7.41 (m, 3H). ^{13}C NMR (126 MHz, $CDCl_3$) δ 148. 7, 138.5, 131.4 (d, *J* = 20 Hz), 129.3, 129.2, 129.0, 128.2, 127.5. ^{19}F NMR (376 MHz, $CDCl_3$) δ 66.52.

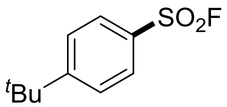

3d

4-(*tert*-Butyl)benzenesulfonyl fluoride (**3d**),[4] 30.7 mg, 71% yield. ^{1}H NMR (500 MHz, $CDCl_3$) δ 7.93 (d, *J* = 8.7 Hz, 2H), 7.64 (d, *J* = 8.6 Hz, 2H), 1.37 (s, 9H). ^{13}C NMR (126 MHz, $CDCl_3$) δ 160.0, 129.9 (d, *J* = 19 Hz), 128.3, 126.7, 35.5, 30.9. ^{19}F NMR (471 MHz, $CDCl_3$) δ 66.21.

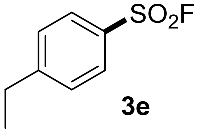

3e

4-Ethylbenzenesulfonyl fluoride (**3e**),[5] 27.4 mg, 73% yield. ^{1}H NMR (400 MHz, $CDCl_3$) δ 7.99 – 7.83 (m, 2H), 7.45 (d, *J* = 8.2 Hz, 2H), 2.78 (q, *J* = 7.6 Hz, 2H), 1.29 (t, *J* = 7.6 Hz, 3H). ^{13}C NMR (101 MHz, $CDCl_3$) δ 153.2 130.2 (d, *J* = 24 Hz), 129.2, 128.6, 29.1, 15.0. ^{19}F NMR (376 MHz, $CDCl_3$) δ 66.24.

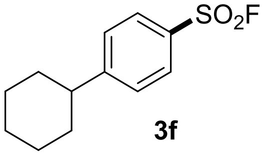

3f

4-Cyclohexylbenzenesulfonyl fluoride (**3f**),[6] 37.3 mg, 77% yield. ^{1}H NMR (400 MHz, $CDCl_3$) δ 7.97 – 7.87 (m, 2H), 7.45 (d, *J* = 8.2 Hz, 2H), 2.64 (tt, *J* = 8.9, 3.4 Hz, 1H), 1.88 (tq, *J* = 9.1, 5.5 Hz, 4H), 1.81 – 1.66 (m, 1H), 1.54 – 1.36 (m, 4H), 1.28 (tdd, *J* = 12.1, 6.9, 3.9 Hz, 1H). ^{13}C NMR (101 MHz, $CDCl_3$) δ 156.8, 130.2 (d, *J* = 24 Hz), 128.6, 128.2, 44.8, 33.9, 26.5, 25.8. ^{19}F NMR (376 MHz, $CDCl_3$) δ 66.25.

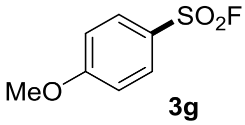

3g

4-Methoxybenzenesulfonyl fluoride (**3g**),[3] 30.1 mg, 79% yield. ^{1}H NMR (500 MHz, $CDCl_3$) δ 7.89 (dd, *J* = 8.4, 1.7 Hz, 2H), 7.42 (d, *J* = 8.1 Hz, 2H), 2.49 (s, 3H). ^{13}C NMR (126 MHz, $CDCl_3$) δ 147.1, 130.3, 130.1 (d, *J* = 2 Hz), 128.4, 21.8. ^{19}F NMR (471 MHz, $CDCl_3$) δ 66.30.

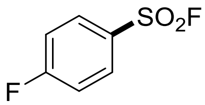

3h

4-Fluorobenzenesulfonyl fluoride (3h),[3] 24.2 mg, 68% yield. ^{1}H NMR (500 MHz, $CDCl_3$) δ 7.97 (dt, *J* = 8.4, 2.8 Hz, 2H), 7.23 (t, *J* = 8.5 Hz, 2H). ^{13}C NMR (126 MHz, $CDCl_3$) δ 167.93 (d, *J* = 133.56 Hz), 131.55 (d, *J* = 8 Hz), 129.04 (d, *J* = 3 Hz), 128.84 (d, *J* = 3 Hz), 117.24 (d, *J* = 19 Hz), 117.15. ^{19}F NMR (471 MHz, $CDCl_3$) δ 66.64.

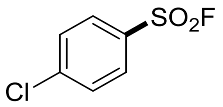

3i

4-Chlorobenzenesulfonyl fluoride (**3i**),[3] 24.7 mg, 64% yield. ^{1}H NMR (400 MHz, $CDCl_3$) δ 7.96 (d, *J* = 8.7 Hz, 2H), 7.69 – 7.55 (m, 2H). ^{13}C NMR (101 MHz, $CDCl_3$) δ 142.7, 131.3 (d, *J* = 26 Hz), 130.1, 129.9. ^{19}F NMR (376 MHz, $CDCl_3$) δ 66.43.

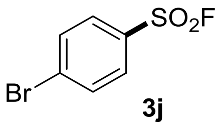

3j

4-Bromobenzenesulfonyl fluoride (**3j**),[5] 31.8 mg, 67% yield. ^{1}H NMR (400 MHz, $CDCl_3$) δ 7.91 – 7.86 (m, 2H), 7.84 – 7.72 (m, 2H).. ^{13}C NMR (126 MHz, $CDCl_3$) δ 133.1, 132.1 (d, *J* = 20 Hz), 131.3, 129.8. ^{19}F NMR (376 MHz, $CDCl_3$) δ 66.38.. HRMS (ESI) m/z: $[M+Na]^+$ Calcd for $C_7H_7FO_3SNa$ 212.9998; Found 212.9992.

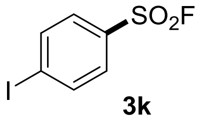

3k

4-Iodobenzenesulfonyl fluoride (**3k**),[6] 24.5 mg, 43% yield. ^{1}H NMR (400 MHz, $CDCl_3$) δ 8.09 – 7.92 (m, 2H), 7.75 – 7.66 (m, 2H). ^{13}C NMR (101 MHz, $CDCl_3$) δ 139.1, 132.6 (d, *J* = 26 Hz), 129.5, 104.1. ^{19}F NMR (376 MHz, $CDCl_3$) δ 66.28.

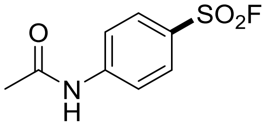

3l

4-Acetamidobenzenesulfonyl fluoride (**3l**),[7] 29.9 mg, 69% yield. ^{1}H NMR (500 MHz, $CDCl_3$) δ 8.01 – 7.92 (m, 2H), 7.79 (d, *J* = 8.7 Hz, 2H), 7.52 (s, 1H), 2.26 (s, 3H). ^{13}C NMR (126 MHz, $CDCl_3$) δ 168.6, 144.4, 130.1, 127.2 (d, *J* = 20 Hz), 119.3, 24.9. ^{19}F NMR (471 MHz, $CDCl_3$) δ 66.81.

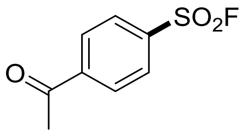

3m

4-Acetylbenzenesulfonyl fluoride (**3m**),[8] 28.3 mg, 70% yield. ^{1}H NMR (400 MHz, $CDCl_3$) δ 8.24 – 8.17 (m, 2H), 8.13 (d, *J* = 8.6 Hz, 2H), 2.71 (s, 3H). ^{13}C NMR (101 MHz, $CDCl_3$) δ 196.2, 142.2, 136.5 (d, *J* = 25 Hz), 129.3, 128.8, 26.9. ^{19}F NMR (376 MHz, $CDCl_3$) δ 65.74.

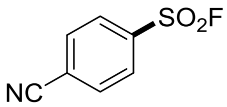

3n

4-Cyanobenzenesulfonyl fluoride (**3n**),[8] 22.9 mg, 62% yield. ^{1}H NMR (400 MHz, $CDCl_3$) δ 8.09 (d, *J* = 8.6 Hz, 2H), 7.94 – 7.84 (m, 2H). ^{13}C NMR (101 MHz, $CDCl_3$) δ 137.0 (d, *J* = 22 Hz), 133.4, 129.1, 119.4, 116.5. ^{19}F NMR (376 MHz, $CDCl_3$) δ 65.97.

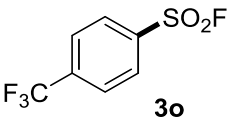

3o

4-(Trifluoromethyl)benzenesulfonyl fluoride (**3o**),[5] 31.0 mg, 68% yield. ^{1}H NMR (400 MHz, $CDCl_3$) δ 8.08 (d, *J* = 8.3 Hz, 2H), 7.84 (d, *J* = 8.3 Hz, 2H). ^{13}C NMR (101 MHz, $CDCl_3$) δ 137.7 – 136.4 (m), 129.1, 126.9 (d, *J* = 3.5 Hz), 124.2 (q, *J* = 260 Hz). ^{19}F NMR (376 MHz, $CDCl_3$) δ 65.68, -63.65.

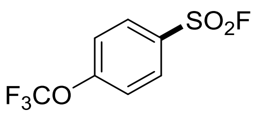

3p

4-(Trifluoromethoxy)benzenesulfonyl fluoride (**3p**),[5] 32.2 mg, 66% yield. ^{1}H NMR (400 MHz, $CDCl_3$) δ 8.18 – 8.05 (m, 2H), 7.59 – 7.43 (m, 2H). ^{13}C NMR (101 MHz, $CDCl_3$) δ 154.4 (q, *J* = 2 Hz), 131.0, 130.9, 121.2, 120.1 (q, *J* = 260 Hz). ^{19}F NMR (376 MHz, $CDCl_3$) δ 66.25, -57.94.

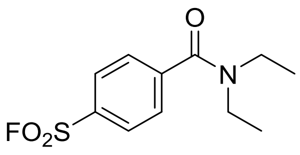

3q

4-(Diethylcarbamoyl)benzenesulfonyl fluoride (**3q**),[9] 32.6 mg, 63% yield. ^{1}H NMR (400 MHz, $CDCl_3$) δ 8.18 – 7.95 (m, 2H), 7.64 (dd, *J* = 8.7, 2.5 Hz, 2H), 3.58 (d, *J* = 7.2 Hz, 2H), 3.22 (d, *J* = 7.2 Hz, 2H), 1.21 (dt, *J* = 56.8, 6.9 Hz, 6H). ^{13}C NMR (126 MHz, $CDCl_3$) δ 168.6, 144.4, 133.4 (d, *J* = 21 Hz), 128.8, 127.5, 43.3, 39.5, 14.2, 12.80. ^{19}F NMR (376 MHz, $CDCl_3$) δ 65.98.

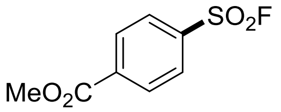

3r

4-(Fluorosulfonyl)phenyl acetate (**3r**),[5] 29.2 mg, 67% yield. ^{1}H NMR (400 MHz, $CDCl_3$) δ 8.32 – 8.26 (m, 2H), 8.12 – 8.07 (m, 2H), 4.00 (s, 3H). ^{13}C NMR (126 MHz, $CDCl_3$) δ 164.9, 136.7 (d, *J* = 20 Hz), 136.5, 130.7, 128.5, 53.0. ^{19}F NMR (376 MHz, $CDCl_3$) δ 65.78.

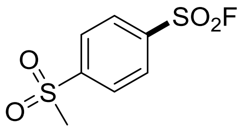

3s

4-(Methylsulfonyl)benzenesulfonyl fluoride (**3s**),[10] 32.8 mg, 69% yield. ^{1}H NMR (400 MHz, $CDCl_3$) δ 8.26 (d, *J* = 2.3 Hz, 4H), 3.15 (d, *J* = 2.4 Hz, 3H). ^{13}C NMR (101 MHz, $CDCl_3$) δ 147.1, 137.9 (d, *J* = 18 Hz), 129.66, 128.96, 44.27. ^{19}F NMR (376 MHz, $CDCl_3$) δ 66.11.

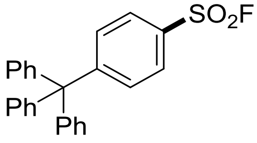

3t

4-Tritylbenzenesulfonyl fluoride (**3t**),[11] 47.4 mg, 59% yield. ^{1}H NMR (400 MHz, $CDCl_3$) δ 7.92 – 7.84 (m, 2H), 7.53 (d, *J* = 8.5 Hz, 2H), 7.37 – 7.22 (m, 10H), 7.20 – 7.17 (m, 5H). ^{13}C NMR (126 MHz, $CDCl_3$) δ 155.7, 145.3, 132.2, 130.9, 128.0, 127.7, 126.6, 65.3. ^{19}F NMR (376 MHz, $CDCl_3$) δ 65.97.

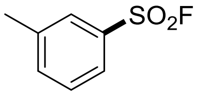

3u

3-Methylbenzenesulfonyl fluoride (**3u**),[3] 27.1 mg, 78% yield. ^{1}H NMR (400 MHz, $CDCl_3$) δ 7.86 – 7.78 (m, 2H), 7.58 (d, *J* = 7.6 Hz, 1H), 7.54 – 7.47 (m, 1H), 2.48 (s, 3H). ^{13}C NMR (101 MHz, $CDCl_3$) δ 140.2, 136.3, 133.2 (d, *J* = 20 Hz), 129.5, 128.7, 125.6, 21.3. ^{19}F NMR (376 MHz, $CDCl_3$) δ 65.77.

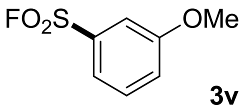

3v

3-Methoxybenzenesulfonyl fluoride (**3v**),[5] 28.5 mg, 75% yield. ^{1}H NMR (400 MHz, $CDCl_3$) δ 7.81 (dd, *J* = 7.9, 1.8 Hz, 1H), 7.61 (ddd, *J* = 8.9, 7.5, 1.8 Hz, 1H), 7.08 – 6.97 (m, 2H), 3.90 (s, 3H). ^{13}C NMR (101 MHz, $CDCl_3$) δ 158.0, 137.6, 131.1, 131.1, 121.1 (d, *J* = 23 Hz), 112.9, 56.5. ^{19}F NMR (376 MHz, $CDCl_3$) δ 58.56.

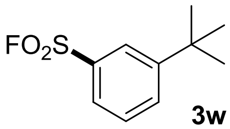

3w

3-(*tert*-Butyl)benzenesulfonyl fluoride (**3w**),[12] 34.6 mg, 80% yield. ^{1}H NMR (500 MHz, $CDCl_3$) δ 8.01 (s, 1H), 7.83 (t, *J* = 7.0 Hz, 2H), 7.57 (t, *J* = 7.9 Hz, 1H), 1.37 (s, 9H). ^{13}C NMR (126 MHz, $CDCl_3$) δ 153.6, 132.9, 132.8, 129.5, 125.6, 125.1, 35.2, 31.0. ^{19}F NMR (471 MHz, $CDCl_3$) δ 65.92.

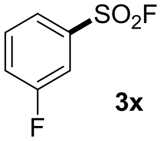

3x

3-Fluorobenzenesulfonyl fluoride (**3x**),[5] 23.7 mg, 67% yield. ^{1}H NMR (400 MHz, $CDCl_3$) δ 7.77 (dt, *J* = 8.0, 1.3 Hz, 1H), 7.66 (dt, *J* = 7.6, 2.1 Hz, 1H), 7.58 (tdd, *J* = 8.0, 5.1, 1.2 Hz, 1H), 7.43 (td, *J* = 8.2, 2.6 Hz, 1H). ^{13}C NMR (101 MHz, $CDCl_3$) δ 162.4 (d, *J* = 253 Hz), 131.6 (d, *J* = 8 Hz), 124.6 (d, *J* = 3 Hz), 123.1 (d, *J* = 21 Hz), 115.95 (d, *J* = 25 Hz). ^{19}F NMR (376 MHz, $CDCl_3$) δ 65.88, -107.61.

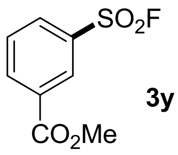

3y

Methyl 3-(fluorosulfonyl)benzoate (**3y**),[8] 30.5 mg, 70% yield. ^{1}H NMR (400 MHz, $CDCl_3$) δ 8.67 (t, *J* = 1.9 Hz, 1H), 8.45 (dt, *J* = 7.9, 1.5 Hz, 1H), 8.20 (dt, *J* = 7.9, 1.6 Hz, 1H), 7.81 – 7.69 (m, 1H), 4.00 (s, 3H). ^{13}C NMR (101 MHz, $CDCl_3$) δ 164.6, 136.3, 133.8 (d, *J* = 26 Hz), 132.2, 132.1, 130.1, 129.5 52.9. ^{19}F NMR (376 MHz, $CDCl_3$) δ 65.93.

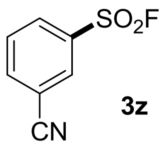

3z

3-Cyanobenzenesulfonyl fluoride (**3z**),[11] 22.6 mg, 61% yield. ^{1}H NMR (400 MHz, $CDCl_3$) δ 8.33 (s, 1H), 8.28 (d, *J* = 8.1 Hz, 1H), 8.10 (d, *J* = 7.8 Hz, 1H), 7.86 (t, *J* = 8.0 Hz, 1H). ^{13}C NMR (101 MHz, $CDCl_3$) δ 138.7, 134.6 (d, *J* = 23 Hz), 132.3, 132.1, 131.0, 116.2, 114.7. ^{19}F NMR (376 MHz, $CDCl_3$) δ 66.41.

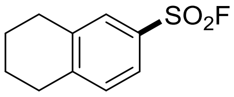

3aa

5,6,7,8-Tetrahydronaphthalene-2-sulfonyl fluoride (**3aa**),[12] 32.1 mg, 75% yield. ^{1}H NMR (500 MHz, $CDCl_3$) δ 7.67 (s, 1H), 7.63 (d, *J* = 8.2 Hz, 1H), 7.27 (d, *J* = 8.2 Hz, 1H), 2.83 (q, *J* = 6.0 Hz, 4H), 1.90 – 1.73 (m, 4H). ^{13}C NMR (126 MHz, $CDCl_3$) δ 146.5, 139.3, 130.4, 129.6 (d, *J* = 18 Hz), 128.9, 125.0, 29.7, 29.2, 22.4, 22.3. ^{19}F NMR (471 MHz, $CDCl_3$) δ 66.19.

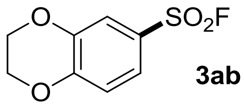

3ab

2,3-Dihydrobenzo[*b*][1,4]dioxine-6-sulfonyl fluoride (**3ab**),[5] 34.4 mg, 79% yield. ^{1}H NMR (400 MHz, $CDCl_3$) δ 7.56 – 7.45 (m, 2H), 7.04 (dd, *J* = 8.4, 1.0 Hz, 1H), 4.40 – 4.35 (m, 2H), 4.35 – 4.30 (m, 2H). ^{13}C NMR (101 MHz, $CDCl_3$) δ 150.0, 144.0, 124.5 (d, *J* = 25 Hz), 122.4, 118.4, 118.1, 64.7, 64.1. ^{19}F NMR (376 MHz, $CDCl_3$) δ 66.95.

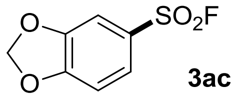

3ac

Benzo[*d*][1,3]dioxole-5-sulfonyl fluoride (**3ac**),[5] 17.3 mg, 72% yield. ^{1}H NMR (500 MHz, $CDCl_3$) δ 7.60 (dd, *J* = 8.3, 1.9 Hz, 1H), 7.37 (d, *J* = 1.9 Hz, 1H), 6.97 (d, *J* = 8.2 Hz, 1H), 6.16 (s, 2H). ^{13}C NMR (126 MHz, $CDCl_3$) δ 153.9, 148.7, 125.7 (d, *J* = 20 Hz), 125.5, 125.2, 108.8, 108.2, 103.0. ^{19}F NMR (471 MHz, $CDCl_3$) δ 66.82.

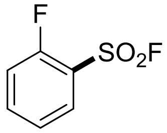

3ad

2-Fluorobenzenesulfonyl fluoride (**3ad**),[10] 21.0 mg, 59% yield. ^{1}H NMR (500 MHz, $CDCl_3$) δ 7.97 (ddd, *J* = 8.2, 6.7, 1.8 Hz, 1H), 7.87 – 7.77 (m, 1H), 7.42 (tt, *J* = 7.7, 1.3 Hz, 1H), 7.36 (ddd, *J* = 9.7, 8.3, 1.0 Hz, 1H). ^{13}C NMR (126 MHz, $CDCl_3$) δ 1159.4 (d, *J* = 210 Hz), 138.3 (d, *J* = 7 Hz), 130.5, 125.0 (d, *J* = 3 Hz), 123.0 (q, *J* = 10 Hz), 117.8 (d, *J* = 15 Hz). ^{19}F NMR (376 MHz, $CDCl_3$) δ 64.21.

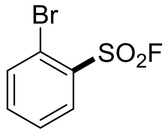

3ae

2-Bromobenzenesulfonyl fluoride (**3ae**),[10] 24.9 mg, 52% yield. ^{1}H NMR (400 MHz, $CDCl_3$) δ 8.12 (dd, *J* = 7.5, 2.2 Hz, 1H), 7.84 (dd, *J* = 7.6, 1.7 Hz, 1H), 7.67 – 7.52 (m, 2H). ^{13}C NMR (101 MHz, $CDCl_3$) δ 136.3, 136.0, 133.8 (d, *J* = 25 Hz), 132.1, 128.1, 120.9. ^{19}F NMR (376 MHz, $CDCl_3$) δ 58.01.

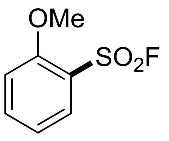

3af

2-Methoxybenzenesulfonyl fluoride (**3af**),[8] 22.0 mg, 58% yield. ^{1}H NMR (500 MHz, $CDCl_3$) δ 7.88 (d, *J* = 8.1 Hz, 1H), 7.70 (t, *J* = 8.0 Hz, 1H), 7.18 – 7.04 (m, 2H), 3.98 (s, 3H). ^{13}C NMR (126 MHz, $CDCl_3$) δ 158.0, 137.7, 130. 9, 121.4 (d, *J* = 82 Hz), 120.5, 113.0, 56.5. ^{19}F NMR (471 MHz, $CDCl_3$) δ 58.6.

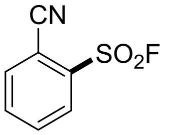

3ag

2-Cyanobenzenesulfonyl fluoride (**3ag**),[11] 19.1 mg, 53% yield. ^{1}H NMR (400 MHz, $CDCl_3$) δ 8.24 (dd, *J* = 7.1, 2.1 Hz, 1H), 8.02 (dd, *J* = 6.9, 2.1 Hz, 1H), 7.98 – 7.87 (m, 2H). ^{13}C NMR (101 MHz, $CDCl_3$) δ 135. 9, 135.6, 135.1 (d, *J* = 26 Hz), 133.5, 130.9, 114.1, 111.9. ^{19}F NMR (376 MHz, $CDCl_3$) δ 64.50.

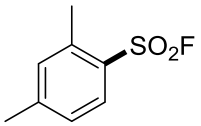

3ah

2,4-Dimethylbenzenesulfonyl fluoride (**3ah**),[12] 24.4 mg, 65% yield. ^{1}H NMR (500 MHz, $CDCl_3$) δ 7.91 – 7.79 (m, 1H), 7.21 (d, *J* = 3.2 Hz, 1H), 7.19 – 7.12 (m, 1H), 2.60 (d, *J* = 4.3 Hz, 3H), 2.40 (d, *J* = 3.9 Hz, 3H). ^{13}C NMR (126 MHz, $CDCl_3$) δ 146.8, 138.7, 133.6, 130.0, 129.2 (d, *J* = 18 Hz), 127.2, 21.4, 20.0. ^{19}F NMR (471 MHz, $CDCl_3$) δ 60.9.

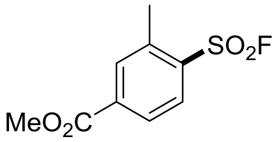

3ai

Methyl 4-(fluorosulfonyl)-3-methylbenzoate (**3ai**),[12] 36.7 mg, 65% yield. ^{1}H NMR (400 MHz, $CDCl_3$) δ 8.16 – 8.08 (m, 2H), 8.04 (dt, *J* = 8.2, 1.7 Hz, 1H), 3.98 (s, 3H), 2.82 – 2.69 (m, 3H). ^{13}C NMR (101 MHz, $CDCl_3$) δ 165.1, 139.4, 136.0, 135.9 (*J* = 23 Hz), 133.7, 130.2, 127.5, 52.9, 20.3. ^{19}F NMR (376 MHz, $CDCl_3$) δ 60.26.

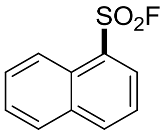

3aj

Naphthalene-1-sulfonyl fluoride (**3aj**),[4] 30.7 mg, 73% yield. ^{1}H NMR (400 MHz, $CDCl_3$) δ 8.40 (dd, *J* = 8.8, 3.0 Hz, 1H), 8.21 (d, *J* = 7.4 Hz, 1H), 8.06 (d, *J* = 8.3 Hz, 1H), 7.83 (dd, *J* = 8.2, 1.4 Hz, 1H), 7.61 (ddd, *J* = 8.6, 6.9, 1.4 Hz, 1H), 7.52 (ddd, *J* = 8.1, 6.9, 1.2 Hz, 1H), 7.44 (ddd, *J* = 8.6, 7.5, 1.5 Hz, 1H). ^{13}C NMR (101 MHz, $CDCl_3$) δ 137.0, 134.0, 131.1 (d, *J* = 0.2 Hz), 129.5, 129.2, 129.1 (d, *J* = 9 Hz), 128.2, 127.7, 124.1 (d, *J* = 2 Hz), 124.0. ^{19}F NMR (376 MHz, $CDCl_3$) δ 62.65.

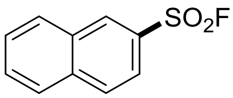

3ak

Naphthalene-2-sulfonyl fluoride (**3ak**),[4] 31.9 mg, 76% yield. ^{1}H NMR (400 MHz, $CDCl_3$) δ 8.54 (d, *J* = 1.9 Hz, 1H), 8.09 – 7.84 (m, 4H), 7.73-7.62 (m, 2H). ^{13}C NMR (101 MHz, $CDCl_3$) δ 136.0, 131.7, 130.9, 130.4, 130.1, 129.8, 129.6, 128.3, 128.1, 122.1. ^{19}F NMR (376 MHz, $CDCl_3$) δ 66.37.

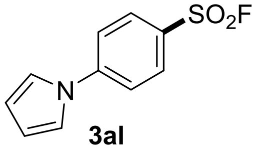

3al

4-(1*H*-Pyrrol-1-yl)benzenesulfonyl fluoride (**3al**), 32.4 mg, 72% yield. ^{1}H NMR (400 MHz, $CDCl_3$) δ 8.12 – 7.98 (m, 2H), 7.63 – 7.55 (m, 2H), 7.19 (t, *J* = 2.2 Hz, 2H), 6.44 (t, *J* = 2.3 Hz, 2H). ^{13}C NMR (126 MHz, $CDCl_3$) δ 146.0, 130.6, 128.6 (d, *J* = 20 Hz), 119.8, 119.0, 112.8. ^{19}F NMR (376 MHz, $CDCl_3$) δ 66.88. HRMS (EI) m/z: [M+Na]+ Calcd for $C_{10}H_8NO_2NaSF$ 248.0157; Found 248.0164.

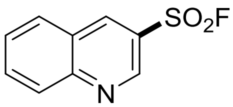

3am

Quinoline-3-sulfonyl fluoride (**3am**),[12] 28.3 mg, 67% yield. ^{1}H NMR (400 MHz, $CDCl_3$) δ 9.35 (d, *J* = 2.3 Hz, 1H), 8.90 (d, *J* = 2.3 Hz, 1H), 8.28 (dd, *J* = 8.5, 1.1 Hz, 1H), 8.09 – 7.97 (m, 2H), 7.79 (ddd, *J* = 8.2, 7.0, 1.2 Hz, 1H). ^{13}C NMR (126 MHz, $CDCl_3$) δ 150.4, 146.1, 138.9, 134.2, 130.0, 129.4, 129.1, 126.3 (d, *J* = 21 Hz), 125.8. ^{19}F NMR (376 MHz, $CDCl_3$) δ 68.77.

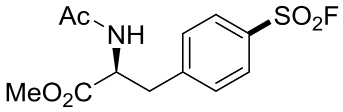

4a

Methyl (*S*)-2-acetamido-3-(4-(fluorosulfonyl)phenyl)propanoate (**4a**), 40.1 mg, 76% yield. ^{1}H NMR (400 MHz, $CDCl_3$) δ 7.94 (d, *J* = 8.2 Hz, 2H), 7.39 (d, *J* = 8.0 Hz, 2H), 6.12 (d, *J* = 7.5 Hz, 1H), 5.00 – 4.86 (m, 1H), 3.76 (s, 3H), 3.27 (ddd, *J* = 46.9, 13.8, 5.9 Hz, 2H), 2.02 (s, 3H). ^{13}C NMR (101 MHz, $CDCl_3$) δ 171.4, 169.7, 145.0, 131.8 (d, *J* = 25 Hz), 130.6, 128.6, 52.8, 52.7, 38.0, 23.2.^{19}F NMR (376 MHz, $CDCl_3$) δ 66.15. HRMS (EI) m/z: $[M+Na]^+$ Calcd for $C_{12}H_{14}NO_5FNaS$ 326.0474; Found 326.0471.

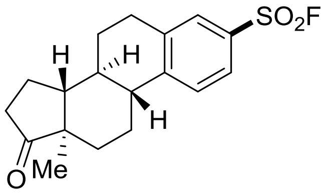

4b

(8*S*,9*R*,13*R*,14*R*)-13-Methyl-17-oxo-7,8,9,11,12,13,14,15,16,17-decahydro-6*H*-cyclopenta[*a*]phenanthrene-3-sulfonyl fluoride (**4b**), 47.7 mg, 71% yield. ^{1}H NMR (400 MHz, $CDCl_3$) δ 7.83 – 7.71 (m, 2H), 7.54 (d, *J* = 8.3 Hz, 1H), 3.01 (td, *J* = 8.2, 7.8, 4.2 Hz, 2H), 2.62 – 2.33 (m, 3H), 2.24 – 1.96 (m, 4H), 1.74 – 1.45 (m, 6H), 0.93 (s, 3H). ^{13}C NMR (126 MHz, $CDCl_3$) δ 148.5, 138.8, 130.2 (d, *J* =19 Hz), 130.1, 128.7, 126.8, 125.6, 50.5, 47.8, 44.7, 37.4, 35.7, 31.4, 29.2, 25.9, 25.5, 21.6, 13.8. ^{19}F NMR (376 MHz, $CDCl_3$) δ 66.14. HRMS (EI) m/z: $[M+H]^+$ Calcd for $C_{18}H_{22}O_3FS$ 337.1274; Found 337.1266.

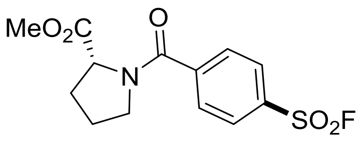

4c

Methyl (4-(fluorosulfonyl)benzoyl)-*D*-prolinate (**4c**), 37.8 mg, 60% yield. Presence of rotamers in a 4:1 ratio. ^{1}H NMR (400 MHz, $CDCl_3$) δ 1H NMR (400 MHz, $CDCl_3$) δ 8.12 – 8.06 (m, 2H), 7.81 (d, J = 8.1 Hz, 2H), 4.70 (dd, J = 8.5, 4.7 Hz, 1H), 3.80 (s, 3H), 3.65 – 3.54 (m, 2H), 3.46 (qd, J = 6.7, 6.2, 4.5 Hz, 1H), 2.44 – 2.25 (m, 1H), 2.19 – 1.88 (m, 3H). ^{13}C NMR (126 MHz, $CDCl_3$) δ 172.2, 167.1, 143.2, 134.3 (d, *J* =20 Hz), 128.7, 128.7, 128.4, 127.9, 61.1, 59.2, 52.6, 52.5, 49.8, 46.8, 31.4, 29.3, 25.3, 22.6. ^{19}F NMR (376 MHz, $CDCl_3$) δ 65.93. HRMS (EI) m/z: $[M+Na]^+$ Calcd for $C_{13}H_{14}NO_5FNaS$ 338.0474; Found 338.0478.

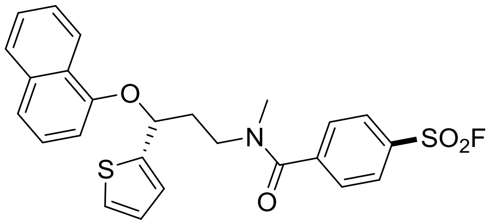

4d

(*R*)-4-(Methyl(3-(naphthalen-1-yloxy)-3-(thiophen-2-yl)propyl)carbamoyl)benzenesulfonyl fluoride (**4d**), 57.0 mg, 59% yield. Presence of rotamers in a 3:2 ratio. ^{1}H NMR (400 MHz, $CDCl_3$) δ 8.41 – 8.27 (m, 1H), 7.94 (d, *J* = 8.0 Hz, 2H), 7.87 (d, *J* = 8.4 Hz, 1H), 7.78 (d, *J* = 7.6 Hz, 2H), 7.59 (d, *J* = 8.0 Hz, 3H), 7.49 (td, *J* = 7.1, 3.0 Hz, 3H), 7.44 – 7.33 (m, 6H), 7.31 – 7.07 (m, 8H), 6.94 (d, *J* = 3.5 Hz, 2H), 6.91 – 6.86 (m, 2H), 6.70 (d, *J* = 7.7 Hz, 1H), 5.84 (dd, *J* = 8.0, 4.5 Hz, 1H), 5.52 (t, *J* = 6.1 Hz, 1H), 3.99 (dt, *J* = 14.3, 7.4 Hz, 1H), 3.66 (ddt, *J* = 22.3, 15.5, 6.7 Hz, 2H), 3.45 (dt, *J* = 13.7, 6.3 Hz, 1H), 3.15 (s, 4H), 2.91 (s, 3H), 2.65 (dt, *J* = 14.8, 7.3 Hz, 1H), 2.41 (dq, *J* = 39.2, 7.2, 5.7 Hz, 4H). ^{13}C NMR (126 MHz, $CDCl_3$) δ 169.4, 168.8, 152.9, 152.4, 144.5, 143.5, 143.5, 142.9, 134.6, 134.5, 133.7, 133.5, 133.4, 133.2, 128.6, 128.4, 128.0, 127.6, 126.8, 126.8, 126.5, 126.00, 125.76, 125.6, 125.5, 125.5, 125.4, 125.1, 125.0, 124.9, 124.7, 121.8, 121.1, 121.0, 107.1, 106.3, 74.6, 72.4, 47.4, 45.2, 37.6, 37.1, 36.0, 32.8. ^{19}F NMR (376 MHz, $CDCl_3$) δ 66.40, 66.02. HRMS (EI) m/z: $[M+Na]^+$ Calcd for $C_{25}H_{22}NO_4FNaS_2$ 506.0872; Found 506.0876.

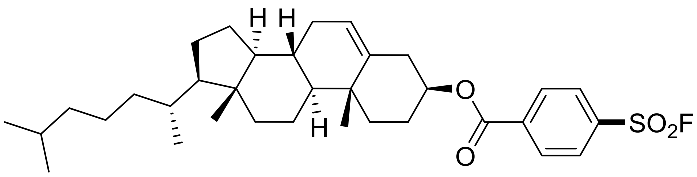

4e

(3*S*,8*S*,9*S*,10*R*,13*R*,14*S*,17*R*)-10,13-Dimethyl-17-((R)-6-methylheptan-2-yl)-2,3,4,7,8,9,10,11,12,13,14,15,16,17-tetradecahydro-1*H*-cyclopenta[a]phenanthren-3-yl 4-(fluorosulfonyl)benzoate (**4e**), 73.2 mg, 64% yield. ^{1}H NMR (400 MHz, $CDCl_3$) δ 8.28 (d, *J* = 8.2 Hz, 2H), 8.14 – 8.00 (m, 2H), 5.49 – 5.36 (m, 1H), 4.91 (tdd, *J* = 11.1, 6.7, 4.5 Hz, 1H), 2.57 – 2.37 (m, 2H), 1.99 (dddd, *J* = 27.2, 13.4, 7.1, 3.5 Hz, 4H), 1.88 – 1.71 (m, 2H), 1.55 – 1.43 (m, 4H), 1.32 – 0.99 (m, 14H), 0.92 (d, *J* = 6.5 Hz, 3H), 0.87 (dd, *J* = 6.6, 1.8 Hz, 6H), 0.69 (s, 3H). ^{13}C NMR (101 MHz, $CDCl_3$) δ 163.8, 139.2, 137.3, 136.5 (d, *J* = 25 Hz), 136.4, 130.7, 128.4, 123.3, 76.0, 56.7, 56.2, 50.1, 42.3, 39.7, 39.5, 38.1, 37.0, 36.6, 36.2, 35.8, 31.9, 31.9, 28.2, 28.0, 27.8, 24.3, 23.8, 22.8, 22.6, 21.1, 19.4, 18.7, 11.9. ^{19}F NMR (376 MHz, $CDCl_3$) δ 65.86. HRMS (EI) m/z: $[M+H]^+$ Calcd for $C_{34}H_{50}NO_4FS$ 573.3414; Found 573.3408.

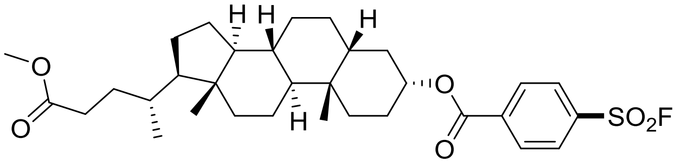

4f

(3*R*,5*R*,8*R*,9*S*,10*S*,13*R*,14*S*,17*R*)-17-((*R*)-5-Methoxy-5-oxopentan-2-yl)-10,13-dimethylhexadecahydro-1H-cyclopenta[*a*]phenanthren-3-yl 4-(fluorosulfonyl)benzoate (**4f**), 72.6 mg, 63% yield. ^{1}H NMR (400 MHz, $CDCl_3$) δ 8.28 (dd, *J* = 7.5, 1.5 Hz, 2H), 8.16 – 8.02 (m, 2H), 5.02 (tt, *J* = 11.3, 4.7 Hz, 1H), 3.67 (s, 3H), 2.36 (ddd, *J* = 15.3, 10.1, 5.1 Hz, 1H), 2.22 (ddd, *J* = 15.6, 9.6, 6.5 Hz, 1H), 2.06 – 1.96 (m, 2H), 1.93 – 1.77 (m, 5H), 1.65 – 1.51 (m, 3H), 1.47 – 1.22 (m, 10H), 1.12 (tdd, *J* = 12.3, 6.3, 3.5 Hz, 5H), 0.97 (s, 3H), 0.92 (d, *J* = 6.3 Hz, 3H), 0.66 (s, 3H). ^{13}C NMR (126 MHz, $CDCl_3$) δ 174.7, 174.7, 163.9, 137.4, 136.4 (d, *J* =20 Hz), 130.7, 128.4, 56.5, 56.0, 51.5, 42.7, 42.0, 40.5, 40.1, 35.8, 35.3, 35.0, 34.6, 32.2, 31.0, 31.0, 28.2, 27.0, 26.7, 26.3, 24.2, 23.3, 20.9, 18.3, 12.1, 12.0. ^{19}F NMR (376 MHz, $CDCl_3$) δ 65.84. HRMS (EI) m/z: $[M+Na]^+$ Calcd for $C_{32}H_{45}O_6FNaS$ 599.2819; Found 599.2813.

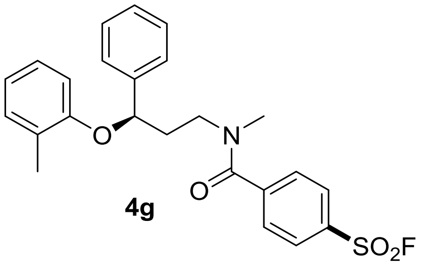

4g

(R)-4-(Methyl(3-phenyl-3-(o-tolyloxy)propyl)carbamoyl)benzenesulfonyl fluoride (**4g**), 48.5 mg, 55% yield. Presence of rotamers in a 1:2 ratio. ^{1}H NMR (400 MHz, $CDCl_3$) δ 7.98 (d, J = 8.0 Hz, 1H), 7.75 (d, J = 8.1 Hz, 2H), 7.48 (d, J = 8.0 Hz, 1H), 7.40 – 7.17 (m, 10H), 7.08 (dd, J = 28.2, 7.4 Hz, 2H), 6.77 (t, J = 7.4 Hz, 1H), 6.44 (d, J = 8.2 Hz, 1H), 5.02 (dd, J = 8.6, 3.5 Hz, 1H), 3.94 – 3.66 (m, 1H), 3.67 – 3.29 (m, 2H), 3.12 (s, 3H), 2.87 (s, 1H), 2.35 (s, 2H), 2.12 (dd, J = 28.8, 4.0 Hz, 2H), 1.89 (s, 3H). ^{13}C NMR (126 MHz, $CDCl_3$) δ 169.4, 168.8, 155.7, 155.1, 143.8, 143.3, 141.5, 140.6, 133.7, 133.5, 133.4, 133.2, 130.9, 130.8, 128.9, 128.8, 128.7, 128.6, 128.13, 128.0, 127.9, 127.8, 127.7, 127.0, 126.8, 126.7, 126.6, 125.8, 125.4, 120.7, 120.6, 112.8, 112.1, 77.7, 77.5, 75.4, 47.6, 45.3, 37.5, 37.0, 36.0, 32.7, 16.6, 16.1. ^{19}F NMR (376 MHz, $CDCl_3$) δ 66.14, 66.01. HRMS (EI) m/z: $[M+Na]^+$ Calcd for $C_{24}H_{24}NO_4FNaS$ 464.1308; Found 464.1302.

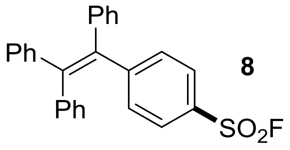

8

4-(1,2,2-Triphenylvinyl)benzenesulfonyl fluoride (**8**), 45.5 mg, 54% yield. ^{1}H NMR (400 MHz, $CDCl_3$) δ 7.74 – 7.67 (m, 2H), 7.25 (dd, J = 8.5, 2.2 Hz, 2H), 7.17 – 7.09 (m, 9H), 7.00 (ddt, J = 8.2, 6.4, 2.8 Hz, 6H). ^{13}C NMR (126 MHz, $CDCl_3$) δ 152.0, 144.3, 142.6, 142.4, 142.4, 138.5, 132.4, 131.2, 131.1, 130.1 (d, J =19 Hz), 130.0, 128.2, 128.2, 127.8, 127.5, 127.2, 127.2. ^{19}F NMR (376 MHz, $CDCl_3$) δ 66.11. HRMS (EI) m/z: $[M+H]^+$ Calcd for $C_{26}H_{20}O_2FS$ 415.1168; Found 415.1161.

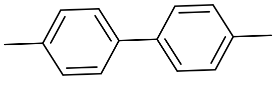

3a''

4,4'-dimethyl-1,1'-biphenyl (**3a''**), 11.5 mg, 63% yield. ^{1}H NMR (400 MHz, $CDCl_3$) δ 7.56 – 7.45 (m, 2H), 7.31 – 7.24 (m, 2H), 2.43 (s, 3H). ^{13}C NMR (101 MHz, $CDCl_3$) δ 138.3, 136.7, 129.5, 126.8, 21.1.

4. One-Pot and Gram Scale Reaction

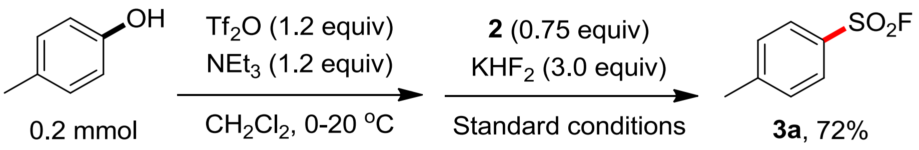

The phenol (21mg，0.2 mmol) was dissolved in DCM (2 mL) and cooled to 0 ℃. NEt_3 (55 μL, 0.4 mmol, 20 equiv) was added dropwise to the solution, which was followed by the addition of triflic anhydride (37 μL, 0.22 mmol, 1.1 equiv). After 5 mins, the ice bath was removed, and the reaction was monitored by TLC. Once the phenol was completely consumed, then the reaction solution is spun dry. The solution of the DABSO (36 mg, 0.15 mmol), KHF_2 (46.8 mg, 0.6 mmol), *n*-Bu_4NClO_4 (0.05 M, 68.2 mg), 9, 10-dicyanoanthracene (**M-1**, 9.12 mg, 0.04 mmol) in 4 mL CH_3CN were added by syringe respectively. The tube was installed with a Pt plate (1.0 × 1.0 cm^2) as the cathode and Carbon felt (1.0 × 1.0 cm^2) as the anode. The mixture was electrolyzed using 12 mA at room temperature under magnetic stirring. The reaction mixture was poured into ethyl acetate (4 mL), washed with water two times (10 mL × 2), dried over Na_2SO_4, and concentrated in vacuo. The residue was purified by column chromatography on silica gel using a mixture of petroleum ether/EtOAc as eluent to afford the desired pure product (24.4 mg, 67%).

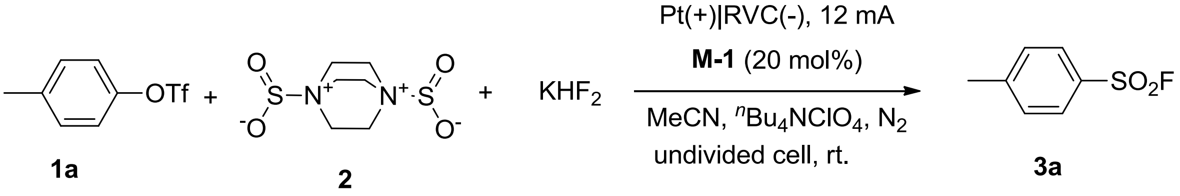

To the cell was added *p*-tolyl trifluoromethanesulfonate **1a** (2.4 g, 10 mmol), DABSO **2** (1.8 g, 7.5 mmol), KHF_2 (2.34 g, 30 mmol), *n*-Bu_4NClO_4 (0.05 M, 3.4 g), 9, 10-dicyanoanthracene (**M-1**, 0.456 g, 2 mmol), CH_3CN (200 mL). The tube was installed with a Pt plate (2.0 × 2.0 cm^2) as the cathode and Reticulated vitreous carbon (2.0 × 2.0 cm^2) as the anode. The mixture was electrolyzed using 12 mA at room temperature under magnetic stirring until complete consumption of the substrate (monitored by TLC). The reaction mixture was poured into ethyl acetate (30 mL), washed with water two times (100 mL × 2), dried over Na_2SO_4, and concentrated in vacuo. The residue was purified by column chromatography on silica gel using a mixture of petroleum ether/EtOAc (50:1) as eluent to afford the desired pure product **3a** (1.17 g, 67%).

5. Procedure for the Synthesis of 1-((2-Bromophenyl)sulfonyl)pyrrolidine

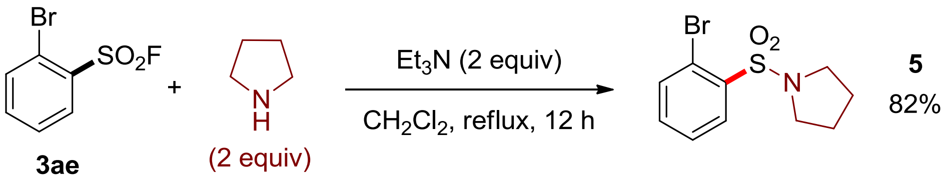

2-Bromobenzenesulfonyl fluoride **3ae** (74 mg, 0.31 mmol) was dissolved in 2 ml DCM followed by pyrrolidine (110 mg, 0. 62 mmol). Triethyl amine (86 μl) was added last and the resulting solution was heated to 40 ℃ (sealed vessel) overnight with stirring. Removal of the volatiles leaves a dark solid which was purified by chromatography to leave the desired compound **5** (73 mg, 82 %). ^{1}H NMR (400 MHz, $CDCl_3$) δ 8.10 (dd, *J* = 7.8, 1.8 Hz, 1H), 7.74 (dd, *J* = 7.7, 1.4 Hz, 1H), 7.45 (td, *J* = 7.6, 1.4 Hz, 1H), 7.39 (td, *J* = 7.6, 1.8 Hz, 1H), 3.49 – 3.35 (m, 3H), 2.88 – 2.74 (m, 1H), 1.96 – 1.86 (m, 3H), 1.23 (q, *J* = 7.7 Hz, 1H). ^{13}C NMR (126 MHz, $CDCl_3$) δ 138.4, 135.6, 133.5, 131.9, 127.6, 120.2, 47.7, 25.7.

6. Fluorescence Spectroscopy of 7 and 8

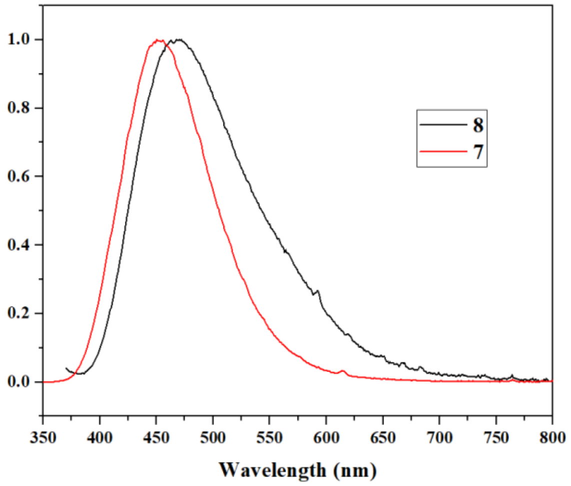

Supplementary Fig. 2 Fluorescence spectra of **7** and **8** in CH_2Cl_2. Concentrations: 10 μM.

7. Mechanistic Studies

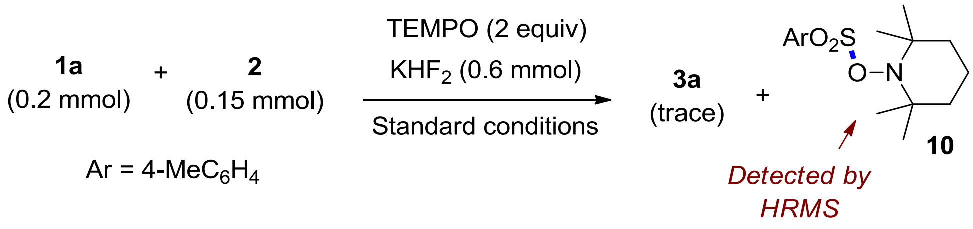

To the cell was added *p*-tolyl trifluoromethanesulfonate **1a** (0.2 mmol), DABSO **2** (36 mg, 0.15 mmol), KHF_2 (46.8 mg, 0.6 mmol), *n*-Bu_4NClO_4 (0.05 M, 68.2 mg), 9, 10-dicyanoanthracene (**M-1**, 9.12 mg, 0.04 mmol), 2,2,6,6-tetramethyl-1-piperidinyloxy (TEMPO, 0.4 mmol, 62.4 mg), CH_3CN (4 mL). The tube was installed with a Pt plate (1.0 × 1.0 cm^2) as the cathode and carbon felt (1.0 × 1.0 cm^2) as the anode. The mixture was electrolyzed using 12 mA at room temperature under magnetic stirring. As showed in the ESI-HRMS spectrum, product **10** were obtained, which implies the existence of *p*-tolyl sulfonyl radical intermediate.

Supplementary Table 1 Trapping aryl sulfonyl radical.

Formula (M)	Ion Formula	Measured	Calc.
$C_{16}H_{25}NO_4S$ (**10**)	$[M+Na]^+$	350.1401	350.1402

Elemental Composition Report **Page 1**

Single Mass Analysis
Tolerance = 20.0 PPM / DBE: min = -1.5, max = 50.0
Element prediction: Off
Number of isotope peaks used for i-FIT = 3

Monoisotopic Mass, Even Electron Ions
703 formula(e) evaluated with 1 results within limits (up to 50 closest results for each mass)
Elements Used:
C: 16-16 H: 25-25 N: 0-20 O: 0-20 Na: 0-3 S: 1-1
10
230409-5-ZYY-4 16 (0.187)

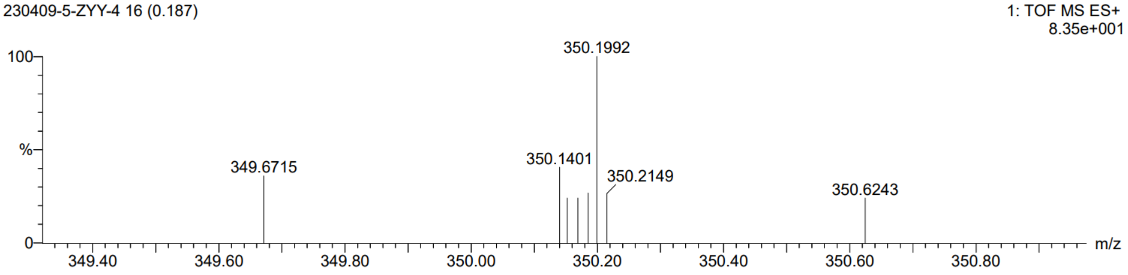

Minimum:				-1.5				
Maximum:		5.0	20.0	50.0				
Mass	Calc. Mass	mDa	PPM	DBE	i-FIT	Norm	Conf(%)	Formula
350.1401	350.1402	-0.1	-0.3	4.5	53.3	n/a	n/a	C16 H25 N O4 Na S

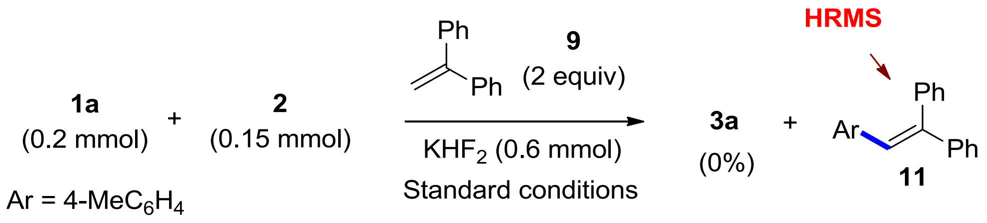

To the cell was added *p*-tolyl trifluoromethanesulfonate **1a** (0.2 mmol), DABSO **2** (36 mg, 0.15 mmol), KHF_2 (46.8 mg, 0.6 mmol), *n*-Bu_4NClO_4 (0.05 M, 68.2 mg), 9, 10-dicyanoanthracene (**M-1**, 9.12 mg, 0.04 mmol), ethene-1,1-diyldibenzene **9** (0.4 mmol, 72.1 mg), CH_3CN (4 mL). The tube was installed with a Pt plate (1.0 × 1.0 cm^2) as the cathode and carbon felt (1.0 × 1.0 cm^2) as the anode. The mixture was electrolyzed using 12 mA at room temperature under magnetic stirring. As showed in the ESI-HRMS spectrum, product **11** were obtained, which imply the existence of 4-methylphenyl radical intermediate.

Supplementary Table 2 Trapping aryl radical.

Formula (M)	Ion Formula	Measured	Calc.
$C_{21}H_{18}O$ (**11**)	$[M+Na]^+$	309.1242	309.1255

Single Mass Analysis
Tolerance = 20.0 PPM / DBE: min = -1.5, max = 50.0
Element prediction: Off
Number of isotope peaks used for i-FIT = 3

Monoisotopic Mass, Even Electron Ions
733 formula(e) evaluated with 1 results within limits (up to 50 closest results for each mass)
Elements Used:
C: 21-21 H: 18-18 N: 0-20 O: 0-20 Na: 0-3
10
230409-5-ZYY-5 5 (0.076)

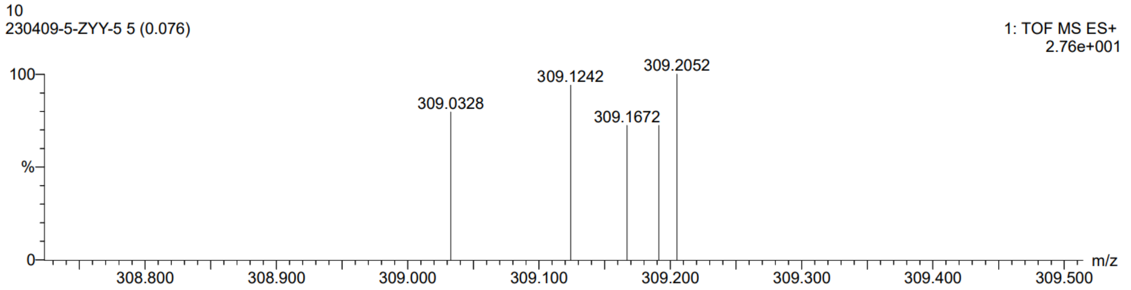

	Mass	Calc. Mass	mDa	PPM	DBE	i-FIT	Norm	Conf(%)	Formula
Minimum:					-1.5				
Maximum:			5.0	20.0	50.0				
	309.1242	309.1255	-1.3	-4.2	12.5	32.2	n/a	n/a	C21 H18 O Na

8. Cyclic Voltammetric (CV) Analysis

Jutand and co-workers have reported that the reduction potential of 4-Me-PhOTf is -2.71 V vs SCE about twenty years ago.[13] Very recently, Prof. Chao-Jun Li and co-workers also measured the reduction potential of 4-Me-PhOTf and found out that the value is around -0.9 V vs. Ag/AgCl.[14] The data of Li is very closed to ours. To further identify this, we also measured the CV of 4-Me-PhOTf (scanning from 0 to -3.0 V), and noticed there are two reduction peaks at -0.95 and -2.4 V, respectively (see below).

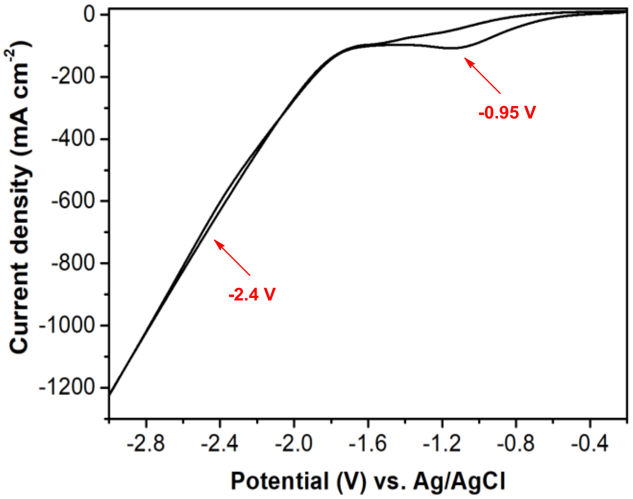

Supplementary Fig. 3 CV experiment of **1a** (35.0 mM), using glass carbon as work electrode, Pt plate and Ag/Ag^+ as counter and reference electrode. Scan rate: 100 mV/s. Solvent: MeCN/$^n Bu_4NClO_4$ (0.1 M).

In fact, as pointed out by Li,[1] there are two electron-deficient sites for ArOTf, as both aryl and S(VI) moiety could acceptor electron. The transfer of electron to these two different sites could result in two different reduction potentials. Considering that S(IV) moiety is much more electron-deficient than that of aryl moiety, it will be much more easily to be reduced at high reduction potential. In addition, the electrolysis at these two different sites could lead to either phenol or arenes, respectively. The electrochemical behavior of ArOTf is different from that of ArBr or ArI, as the latter two compounds can be generally reduced to form aryl radical.

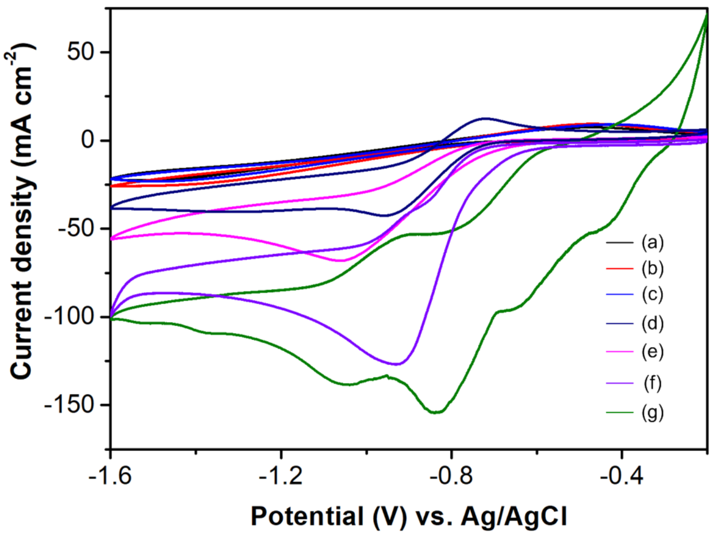

Supplementary Fig. 4 CV experiments, using glass carbon as work electrode, Pt plate and Ag/Ag^{+} as counter and reference electrode. Scan rate: 100 mV/s. Solvent: MeCN/$^{n}Bu_4NClO_4$ (0.1 M). Experiments were conducted under Ar unless otherwise note. (a) Background; (b) DABSO (25.0 mM); (c) KHF_2 (25.0 mM); (d) **M-1** (25.0 mM); (e) **1a** (25.0 mM); (f) **M-1** (5.0 mM) + **1a** (25.0 mM); (g) **1a** (25.0 mM) + **M-1** (5.0 mM) **+** DABSO (25.0 mM) + KHF_2 (25.0 mM)

9. Unsuccessful Substrates

The following three types of substrates fail in our model reaction conditions.

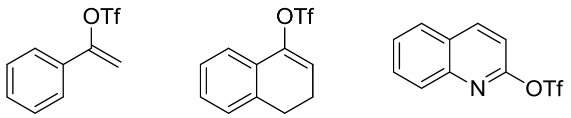

10. DFT Calculations

Computational studies Computational details: All calculations were performed using Gaussian 16, Revision A.03 package.[15] All of the reactants, intermediates, transition states, products were optimized by the DFT with the M06-2X functional.[16] For geometry optimizations and frequency calculations, we employed 6-31+g(d,p) basis sets for H, C, O, S, F, and N. All the structures were characterized with no imaginary frequency. The solvent effect of Acetonitrile was evaluated through the SMD method,[17] in which a better basis system was used. We employed def2-TZVP basis sets for all atoms. All reported energies are free energies at a concentration of 1 M and a temperature of 298.15 K. The Charge decomposition analysis was carried out by the Multiwfn program.[18-19]

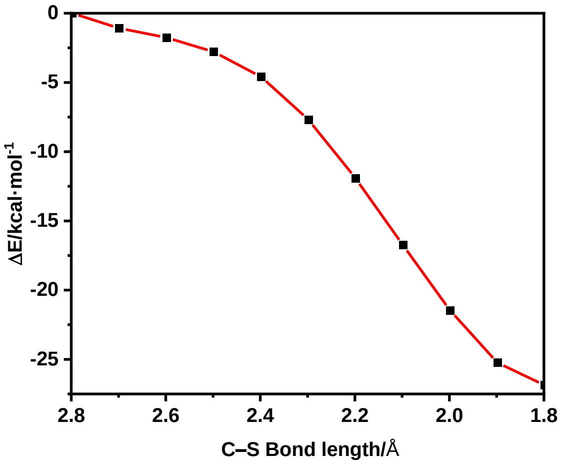

Supplementary Fig. 5 DFT scanned barrierless phenyl radical trap by SO_2 (in kcal/mol).

11. Copies of NMR Spectra

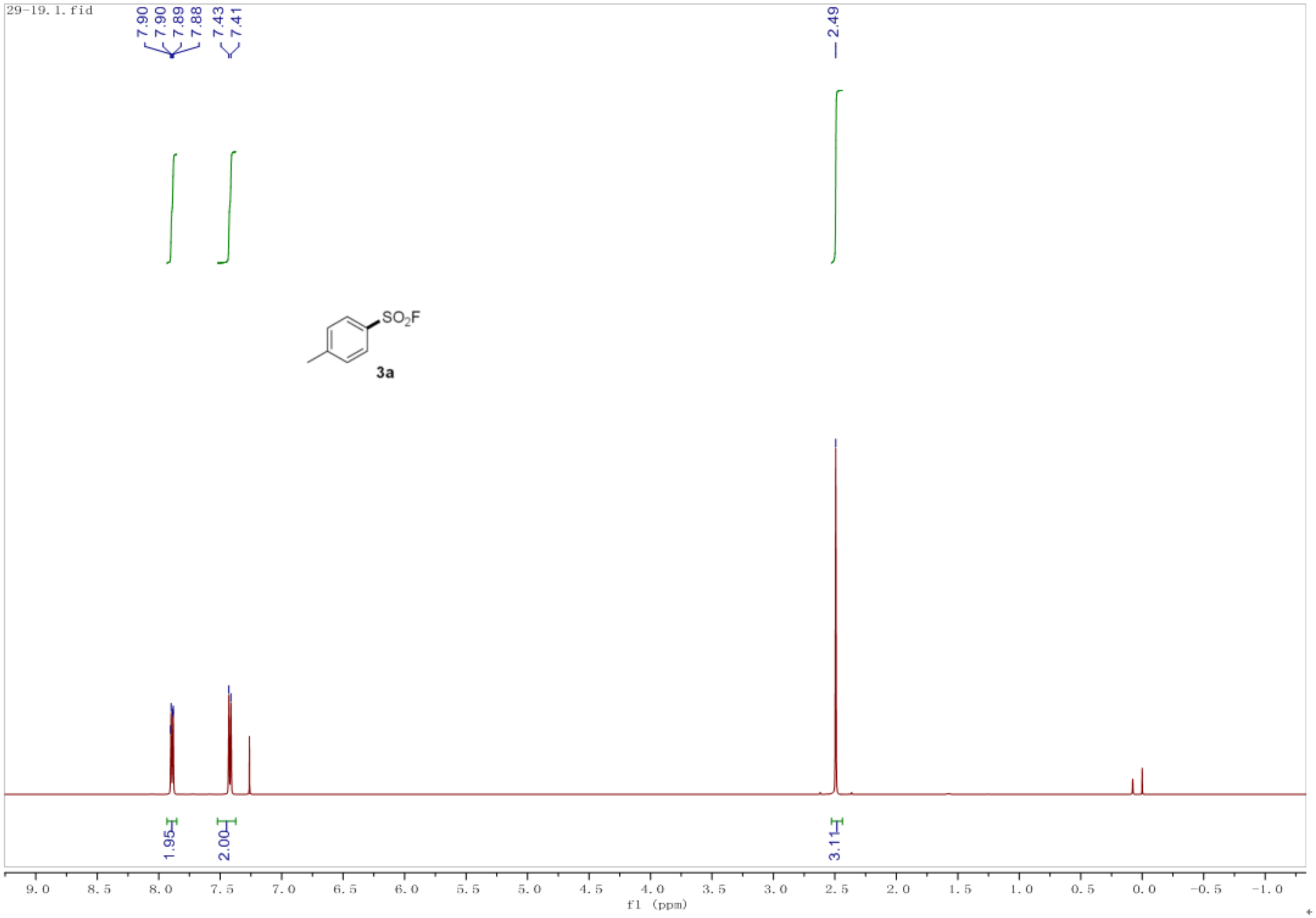

Supplementary Fig. 6 ^{1}H NMR spectrum of compound 3a ($CDCl_3$, 500 MHz, 298K)

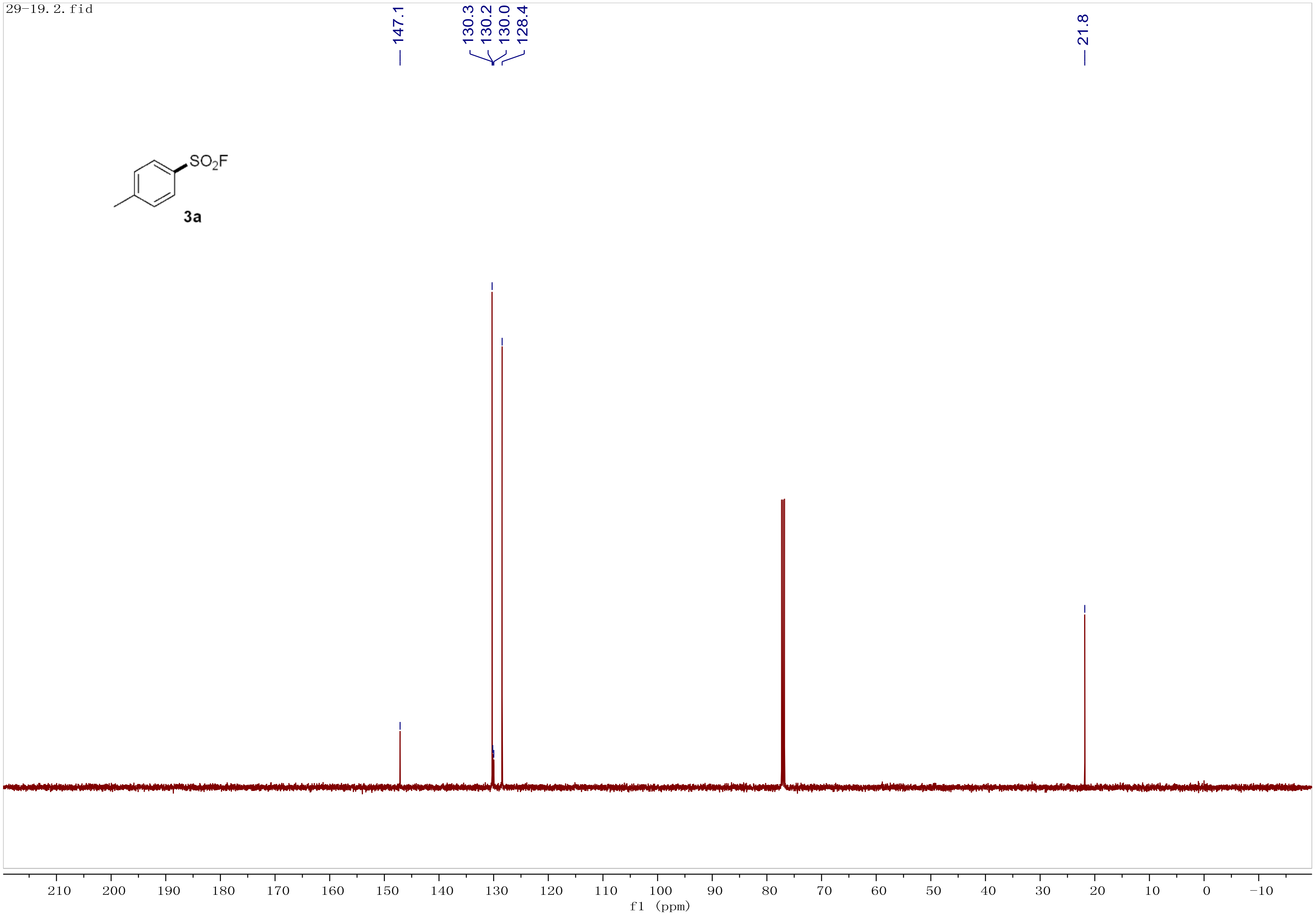

Supplementary Fig. 7 ^{13}C NMR spectrum of compound 3a ($CDCl_3$, 126 MHz, 298K)

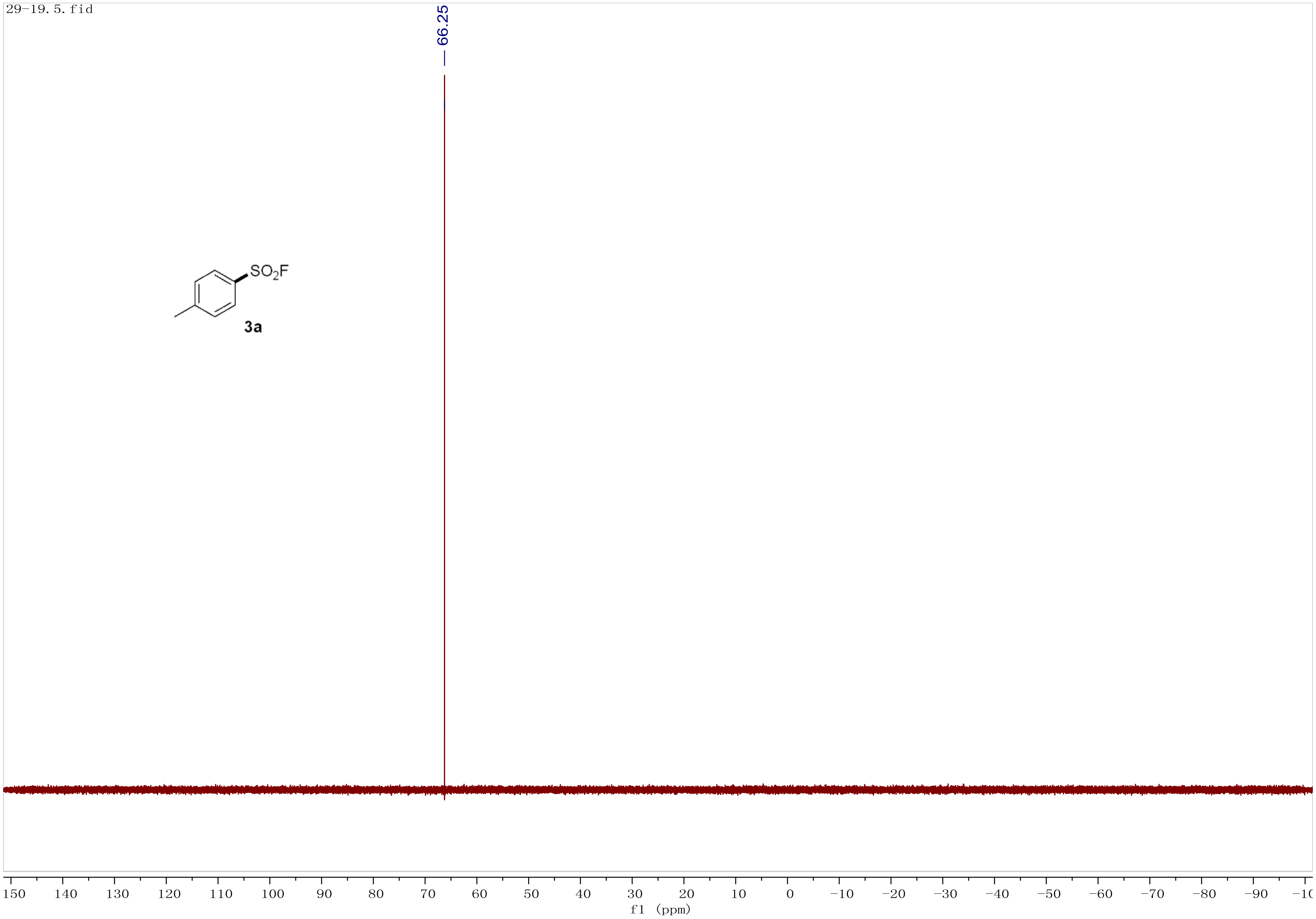

Supplementary Fig. 8 ^{19}F NMR spectrum of compound 3a ($CDCl_3$, 376 MHz, 298K)

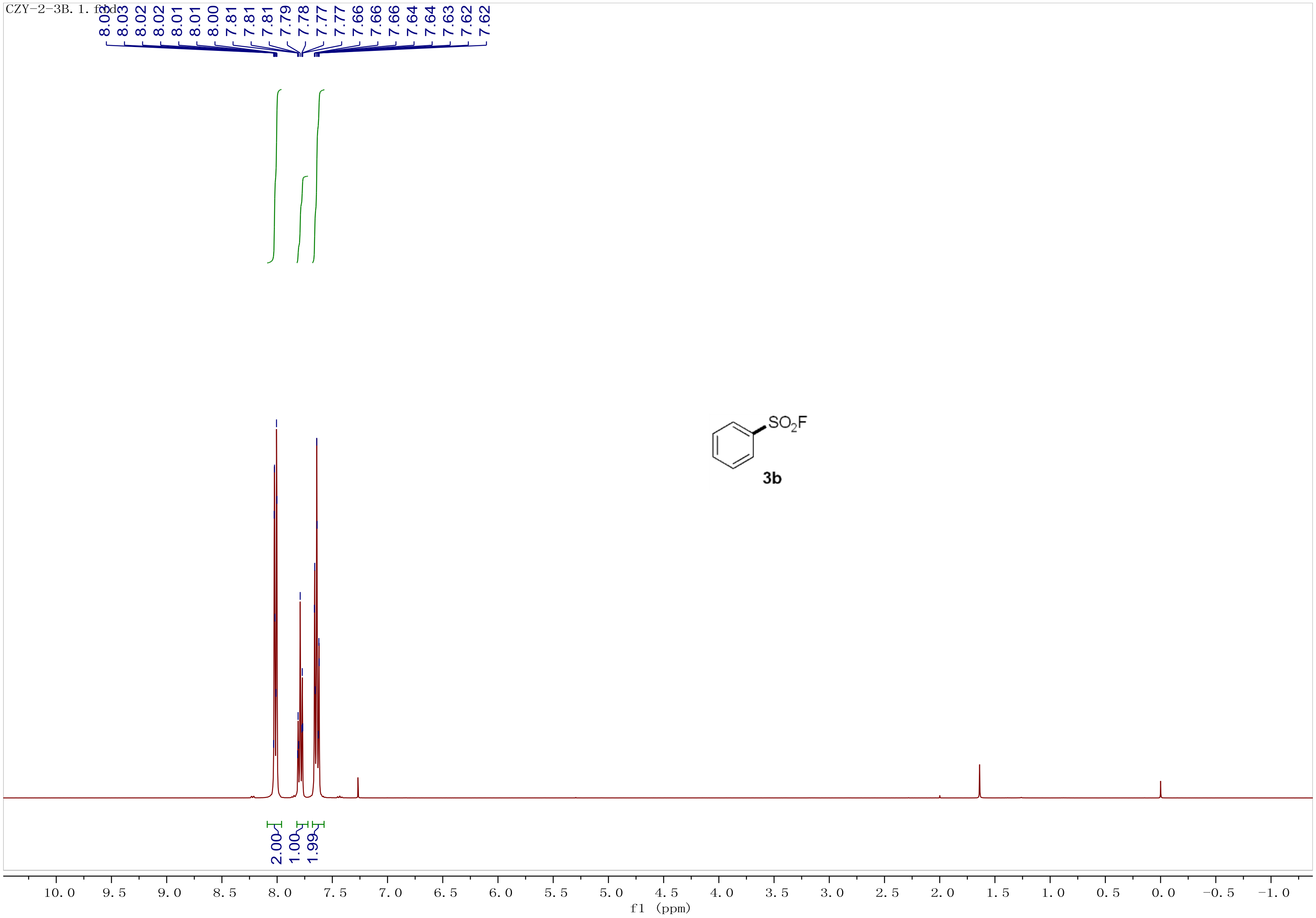

Supplementary Fig. 9 ^{1}H NMR spectrum of compound 3b ($CDCl_3$, 400 MHz, 298K)

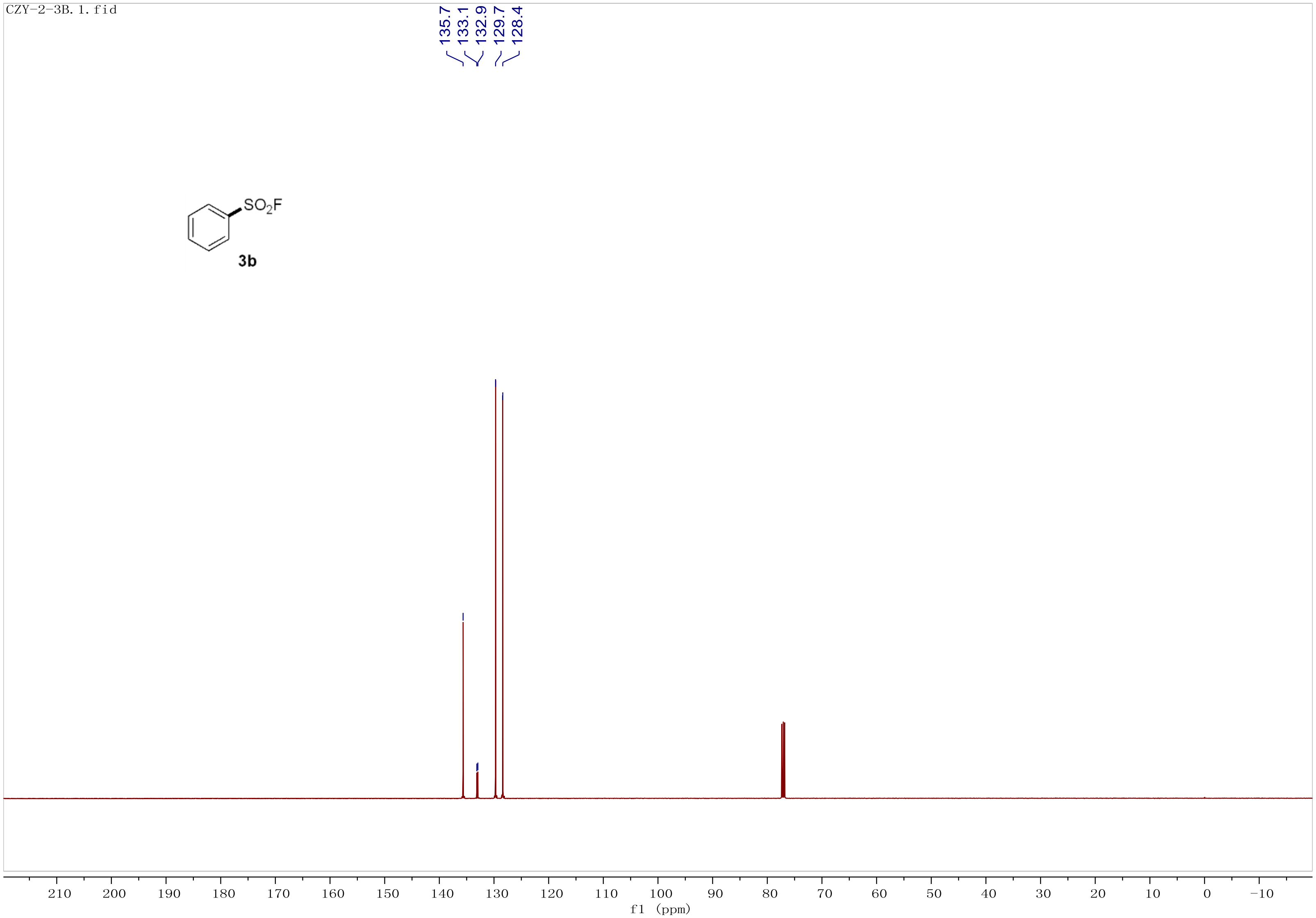

Supplementary Fig. 10 ^{13}C NMR spectrum of compound 3b ($CDCl_3$, 126 MHz, 298K)

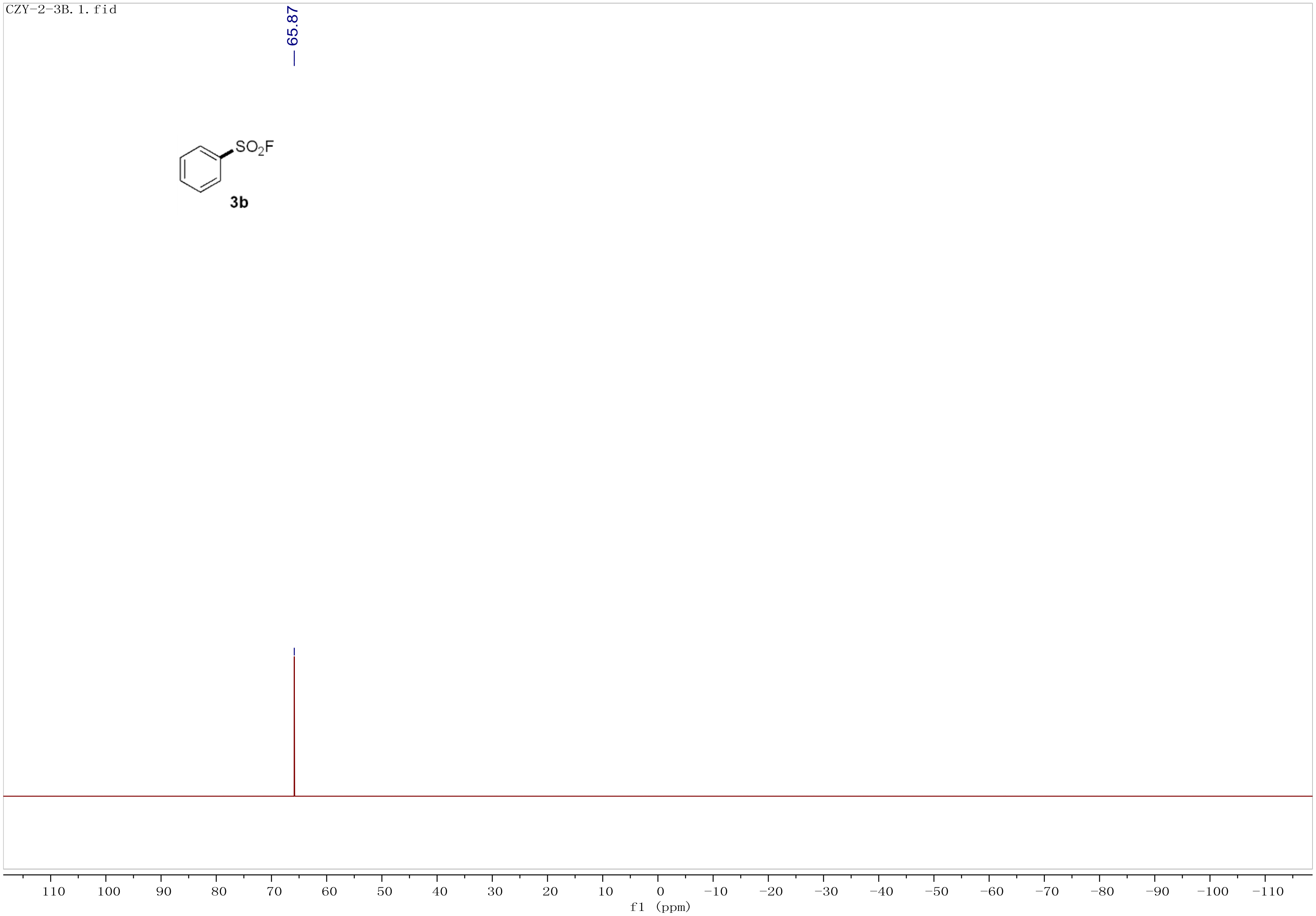

Supplementary Fig. 11 ^{19}F NMR spectrum of compound 3b ($CDCl_3$, 376 MHz, 298K)

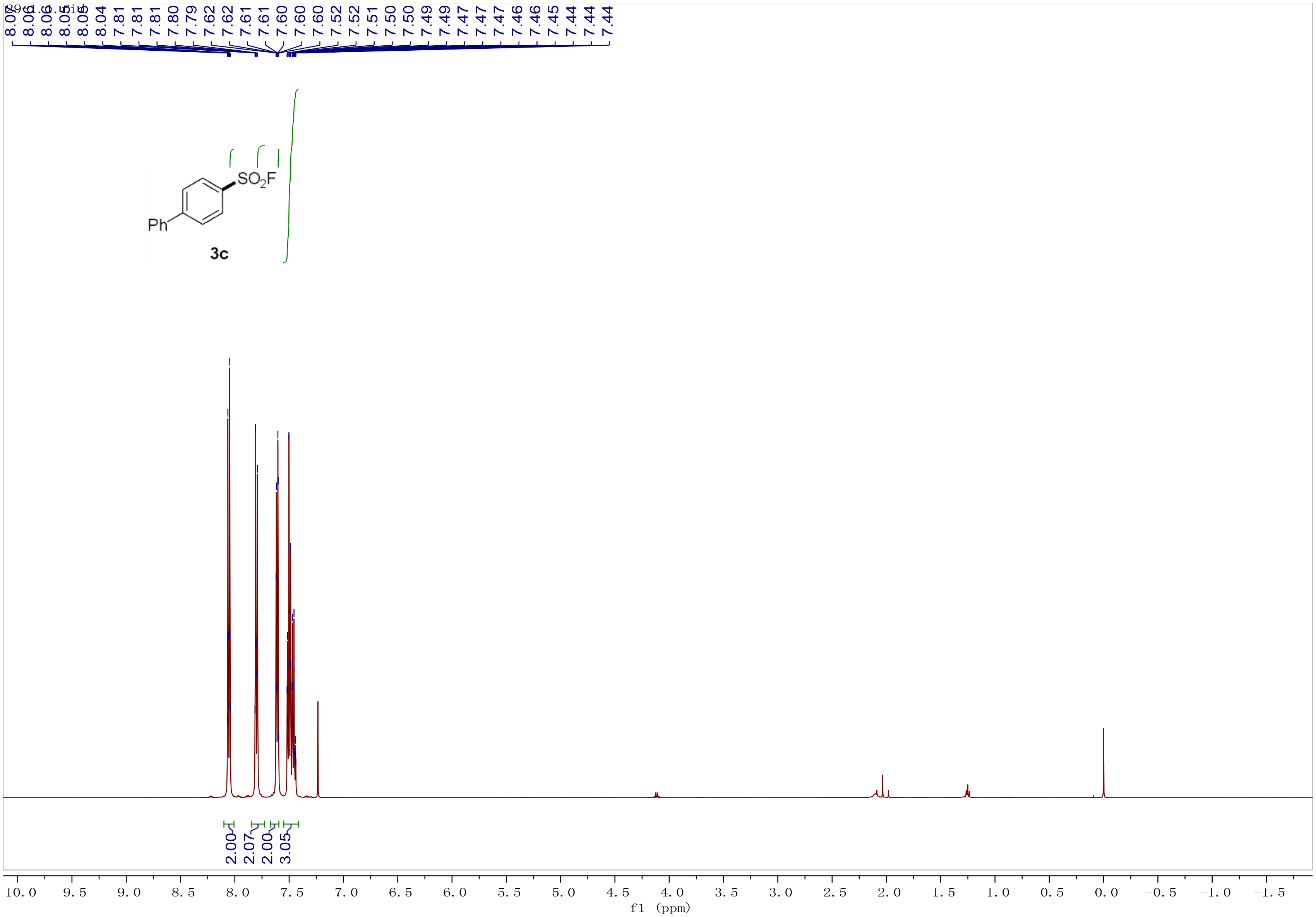

Supplementary Fig. 12 ^{1}H NMR spectrum of compound 3c ($CDCl_3$, 500 MHz, 298K)

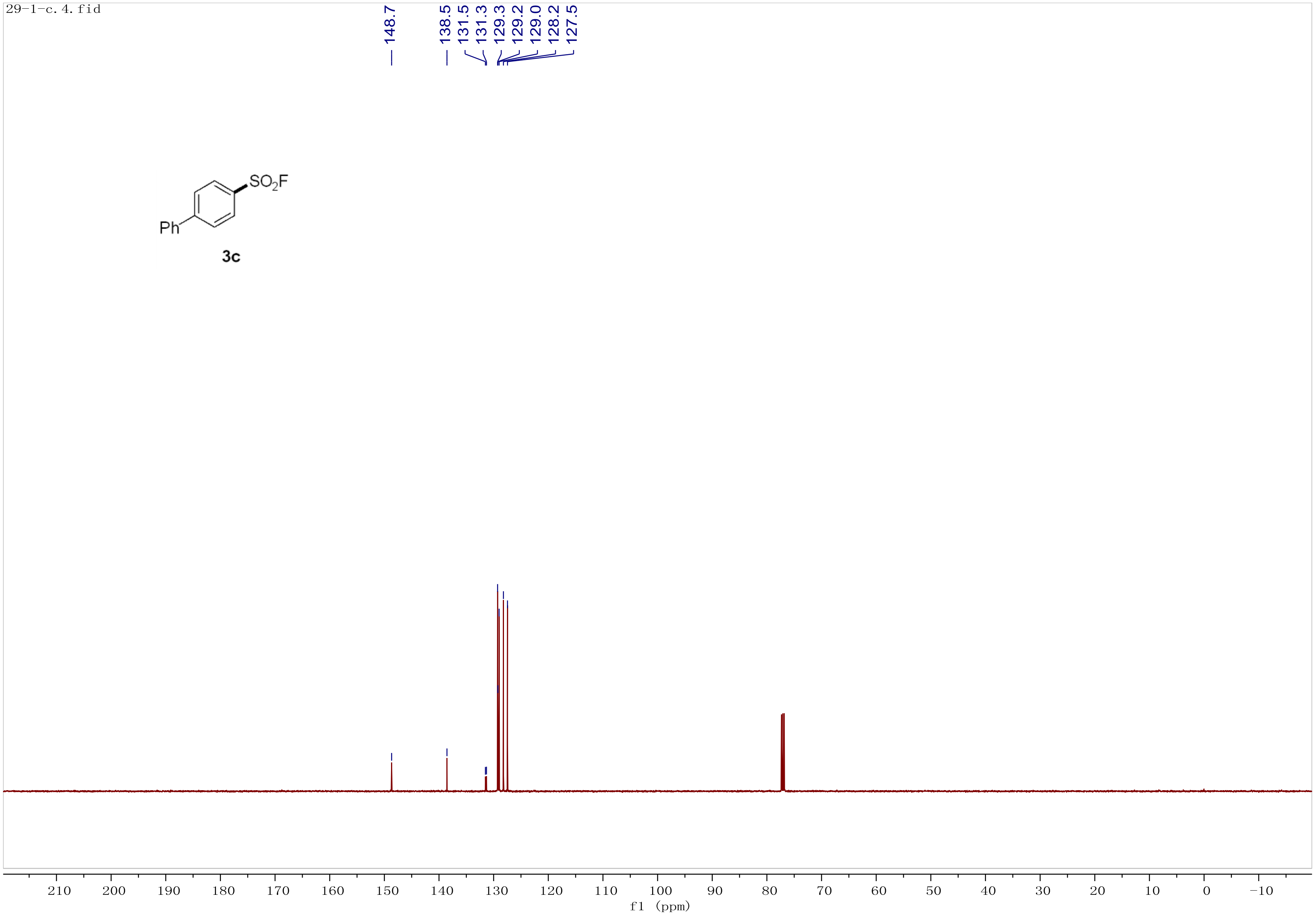

Supplementary Fig. 13 ^{13}C NMR spectrum of compound 3c ($CDCl_3$, 126 MHz, 298K)

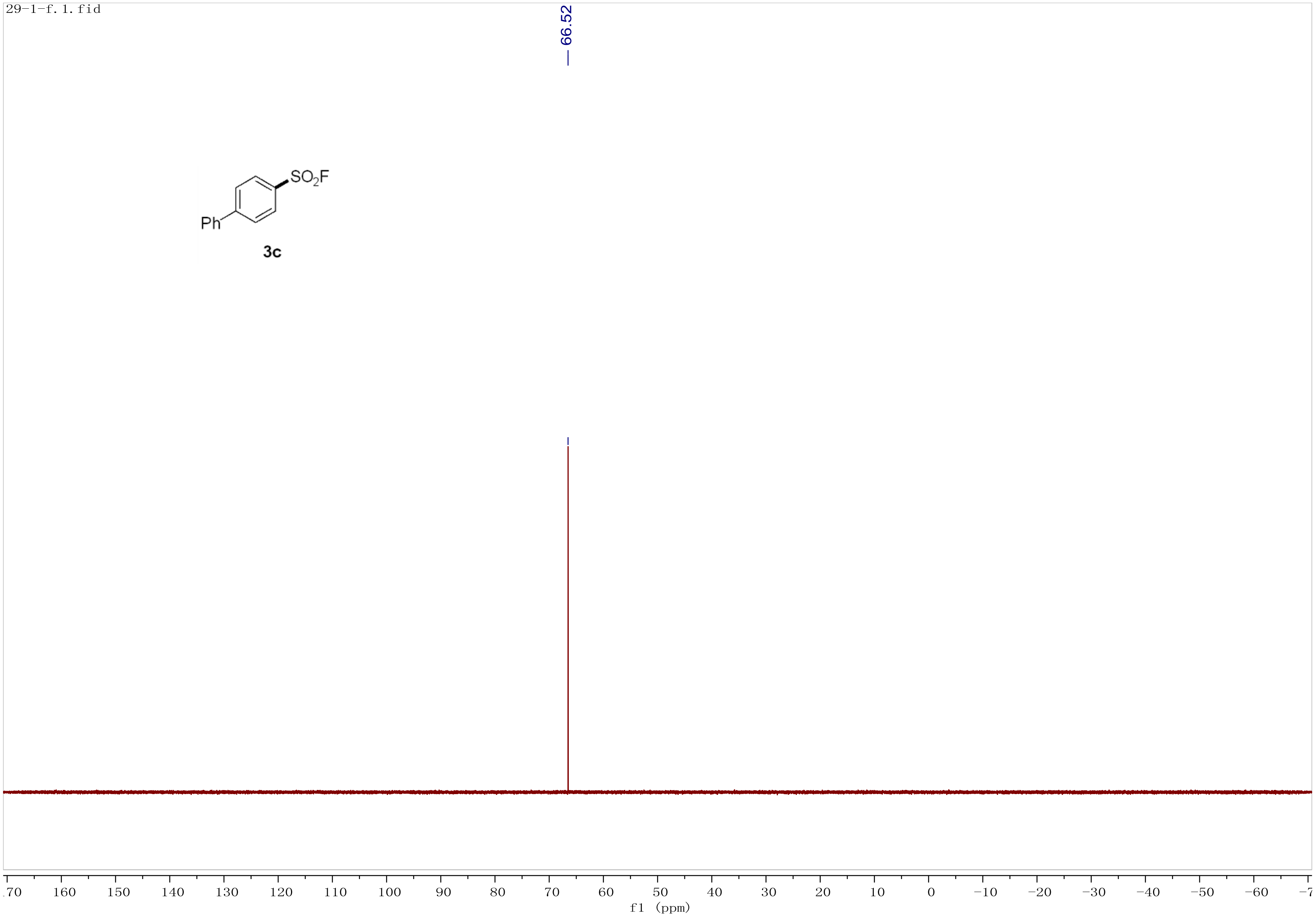

Supplementary Fig. 14 ^{19}F NMR spectrum of compound 3c ($CDCl_3$, 376 MHz, 298K)

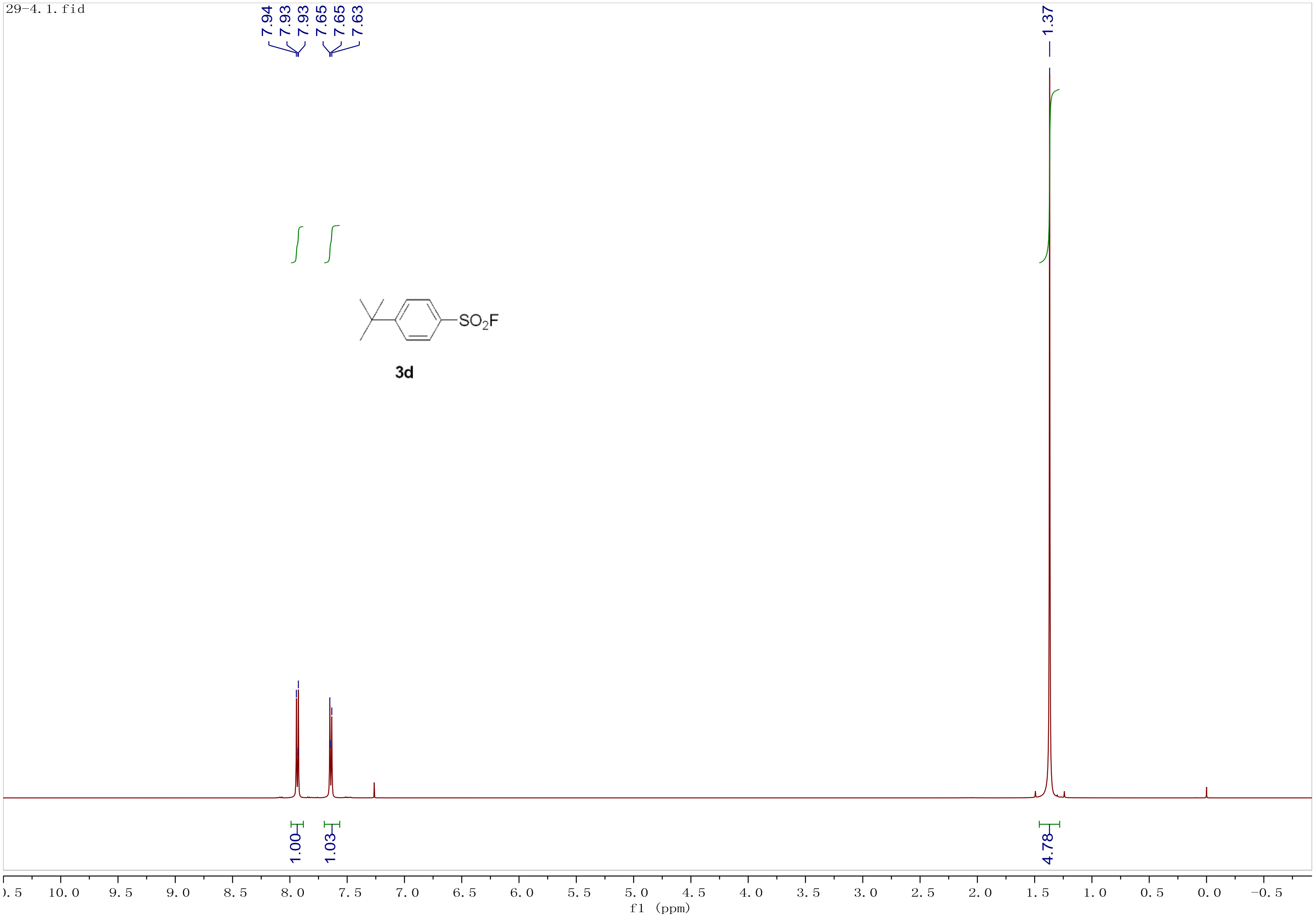

Supplementary Fig. 15 ^{1}H NMR spectrum of compound 3d ($CDCl_3$, 500 MHz, 298K)

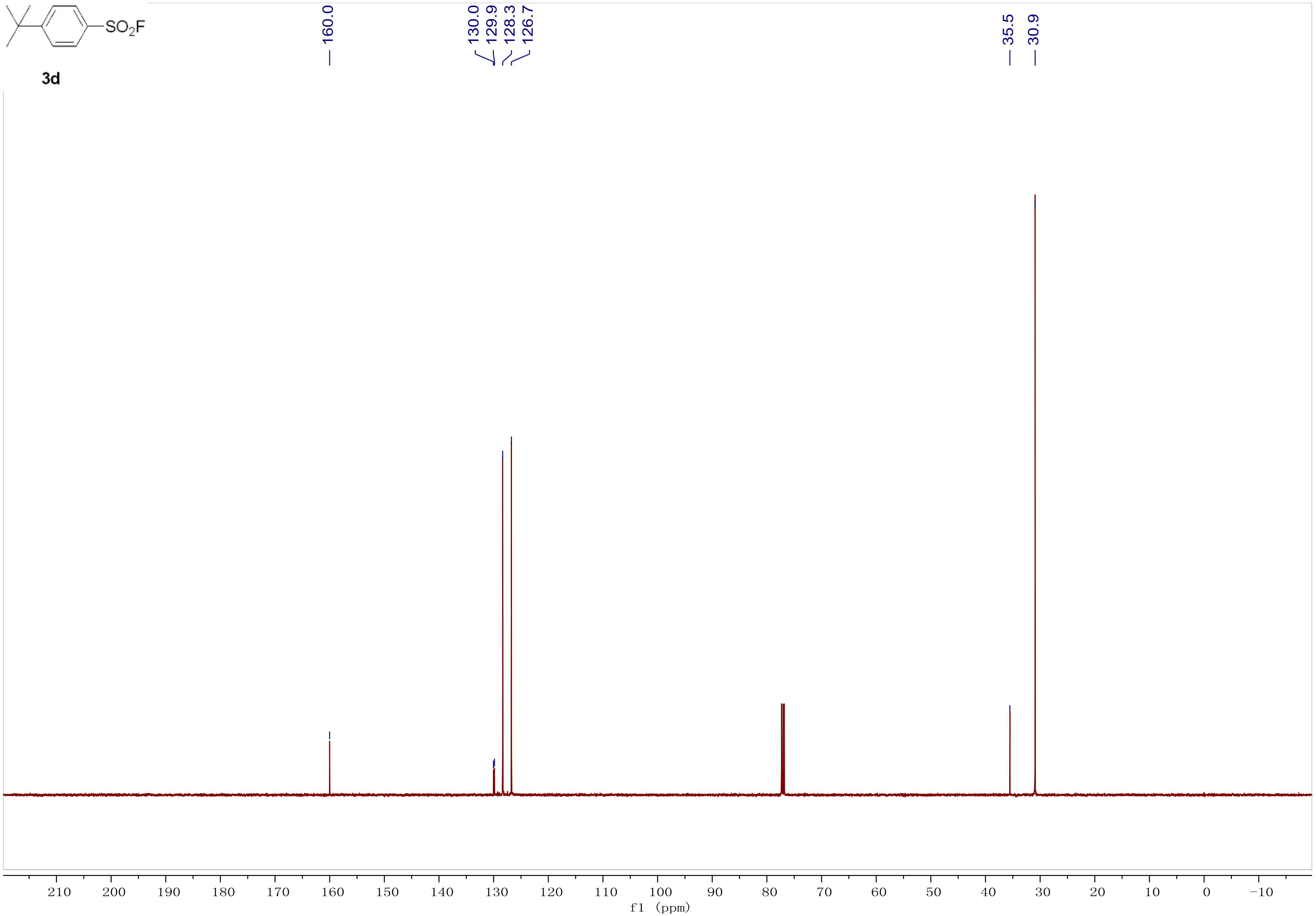

Supplementary Fig. 16 ^{13}C NMR spectrum of compound 3d ($CDCl_3$, 126 MHz, 298K)

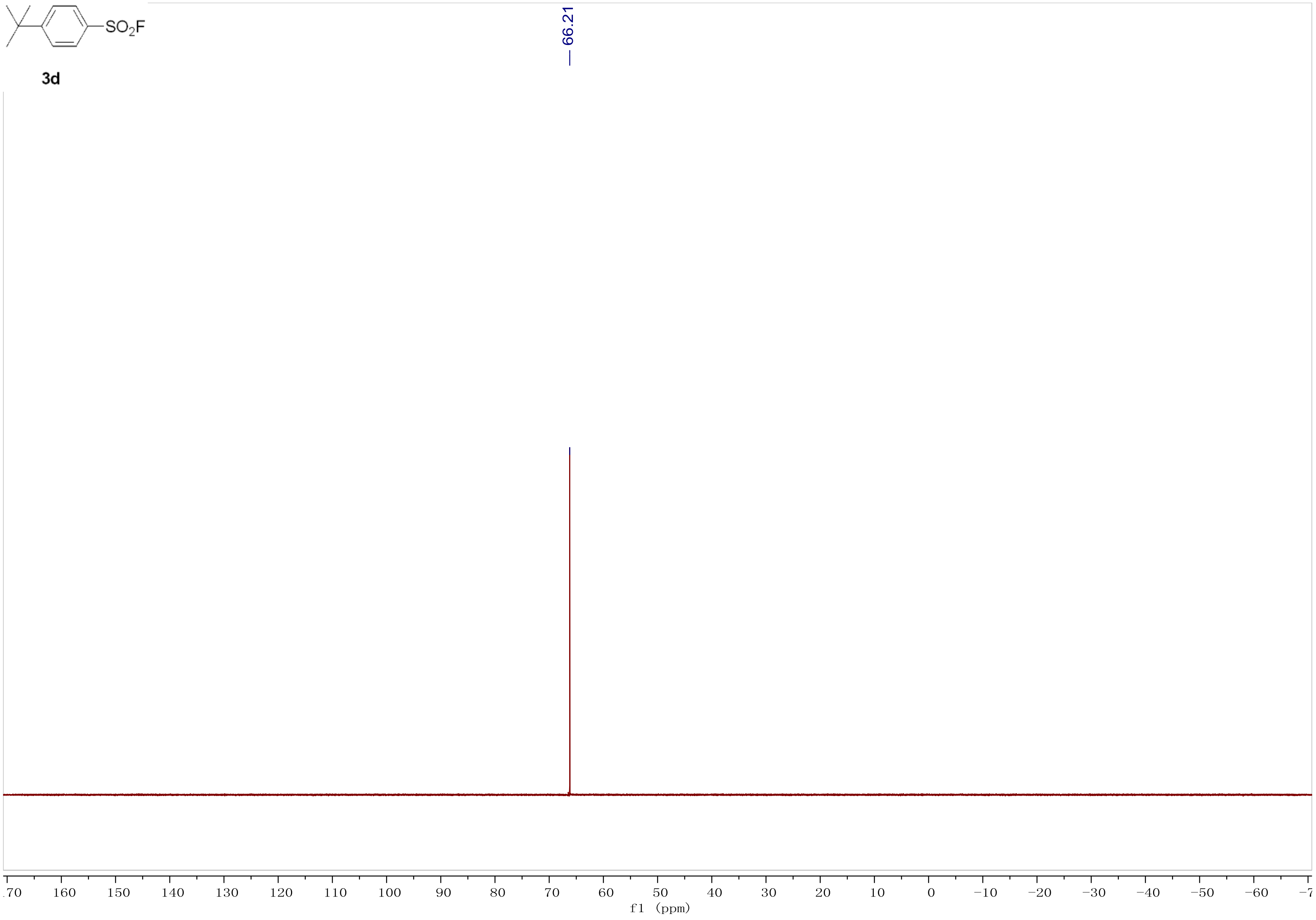

Supplementary Fig. 17 ^{19}F NMR spectrum of compound 3d ($CDCl_3$, 471 MHz, 298K)

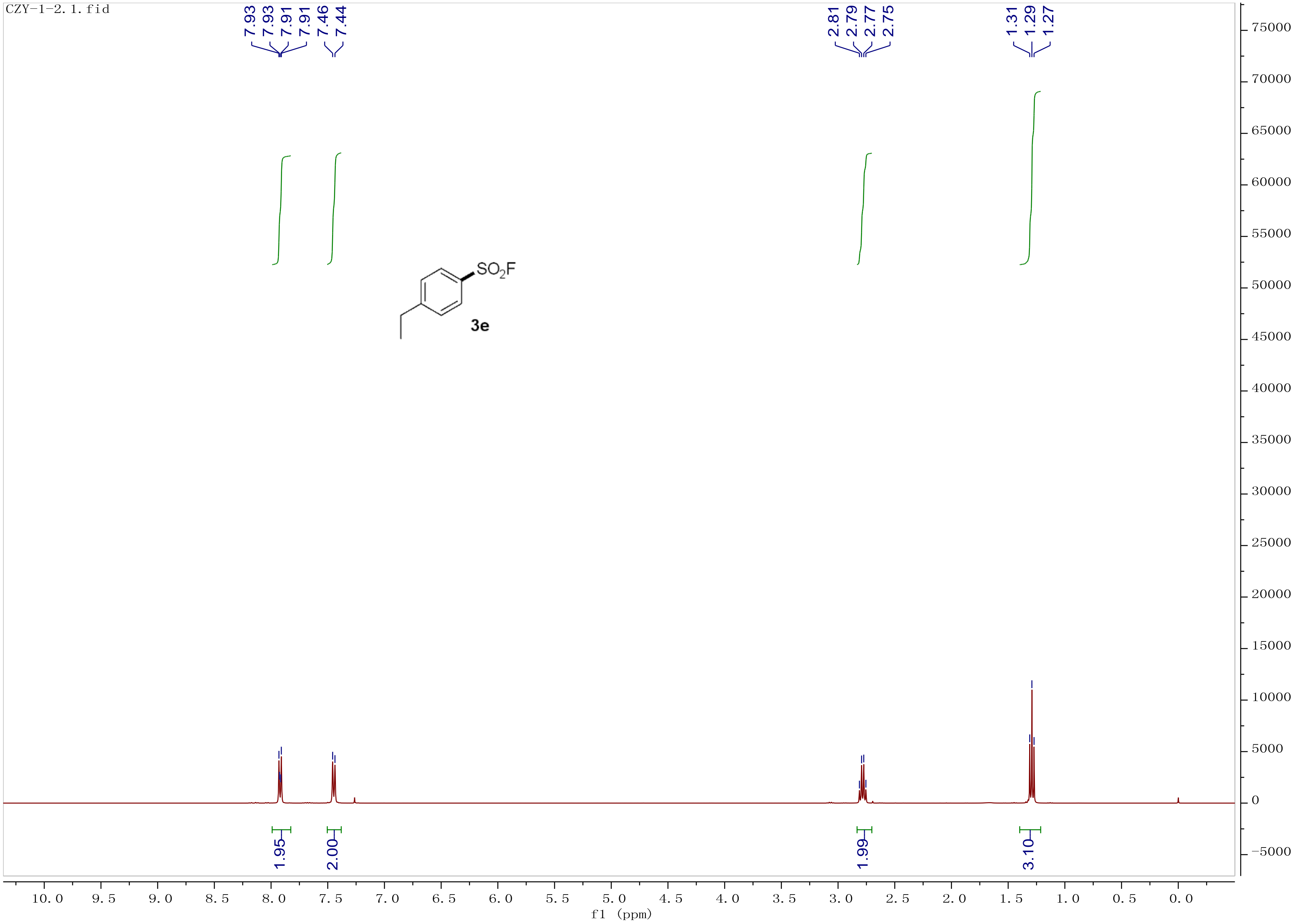

Supplementary Fig. 18 ^{1}H NMR spectrum of compound 3e ($CDCl_3$, 400 MHz, 298K)

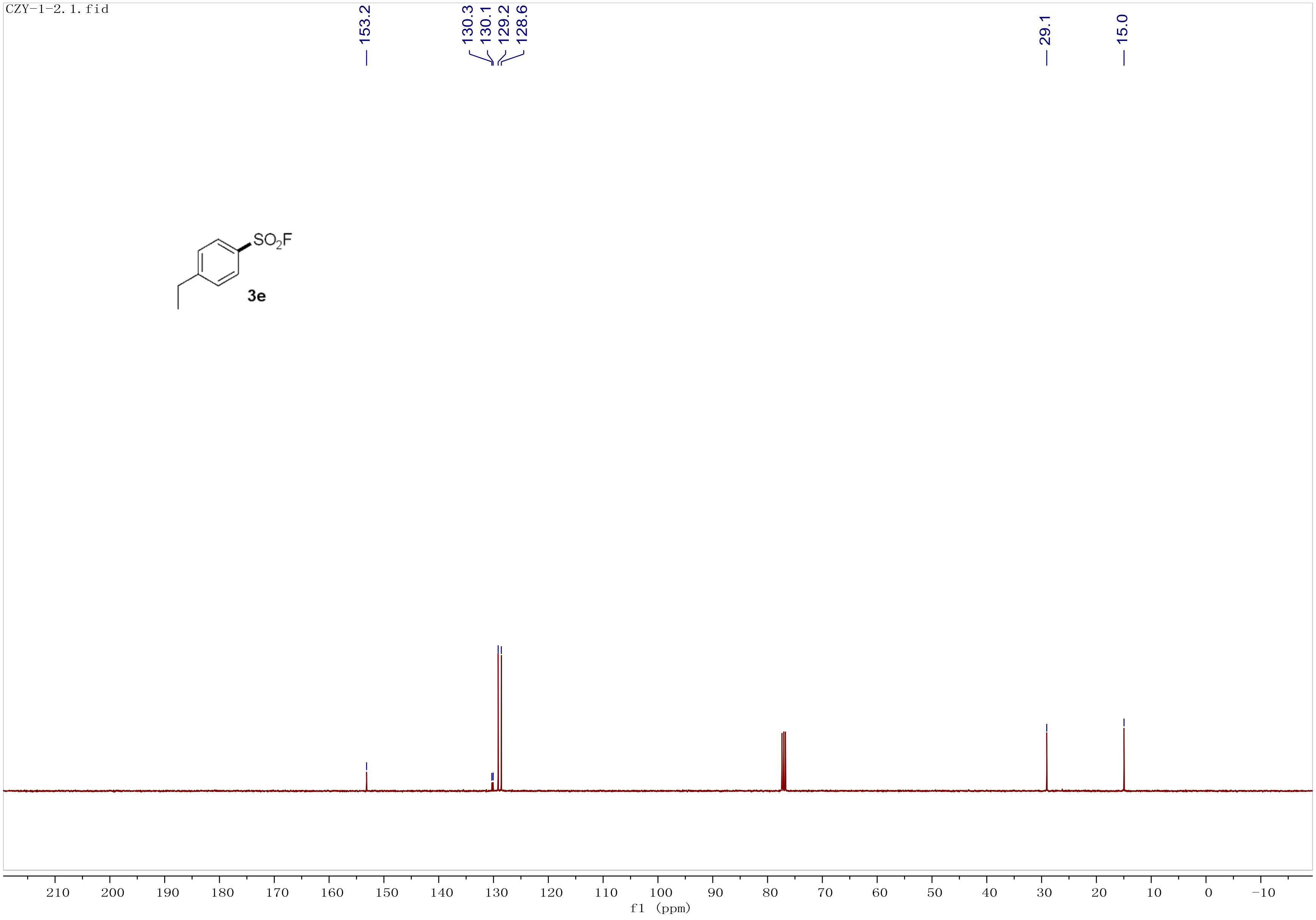

Supplementary Fig. 19 ^{13}C NMR spectrum of compound 3e ($CDCl_3$, 101 MHz, 298K)

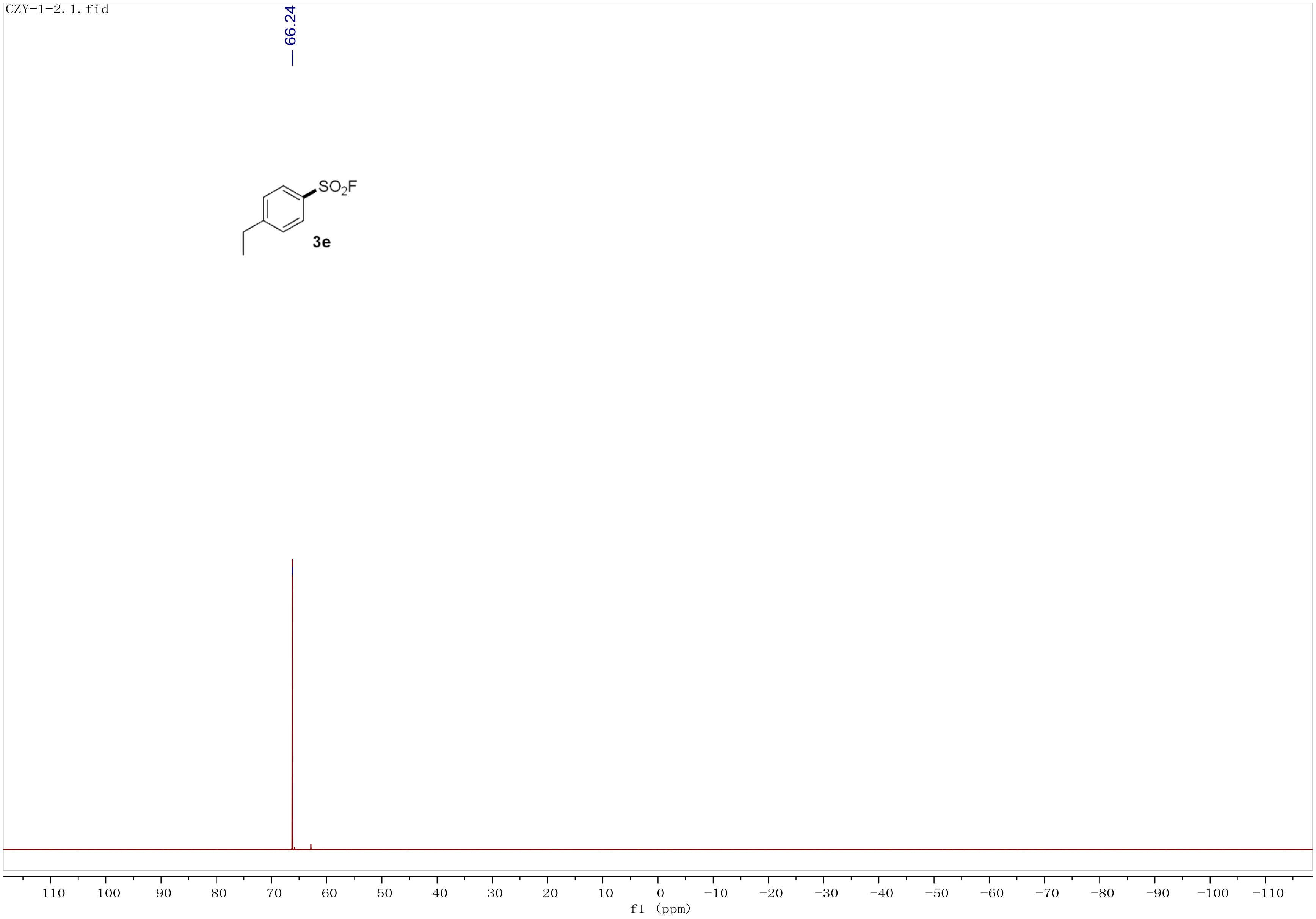

Supplementary Fig. 20 ^{19}F NMR spectrum of compound 3e ($CDCl_3$, 376 MHz, 298K)

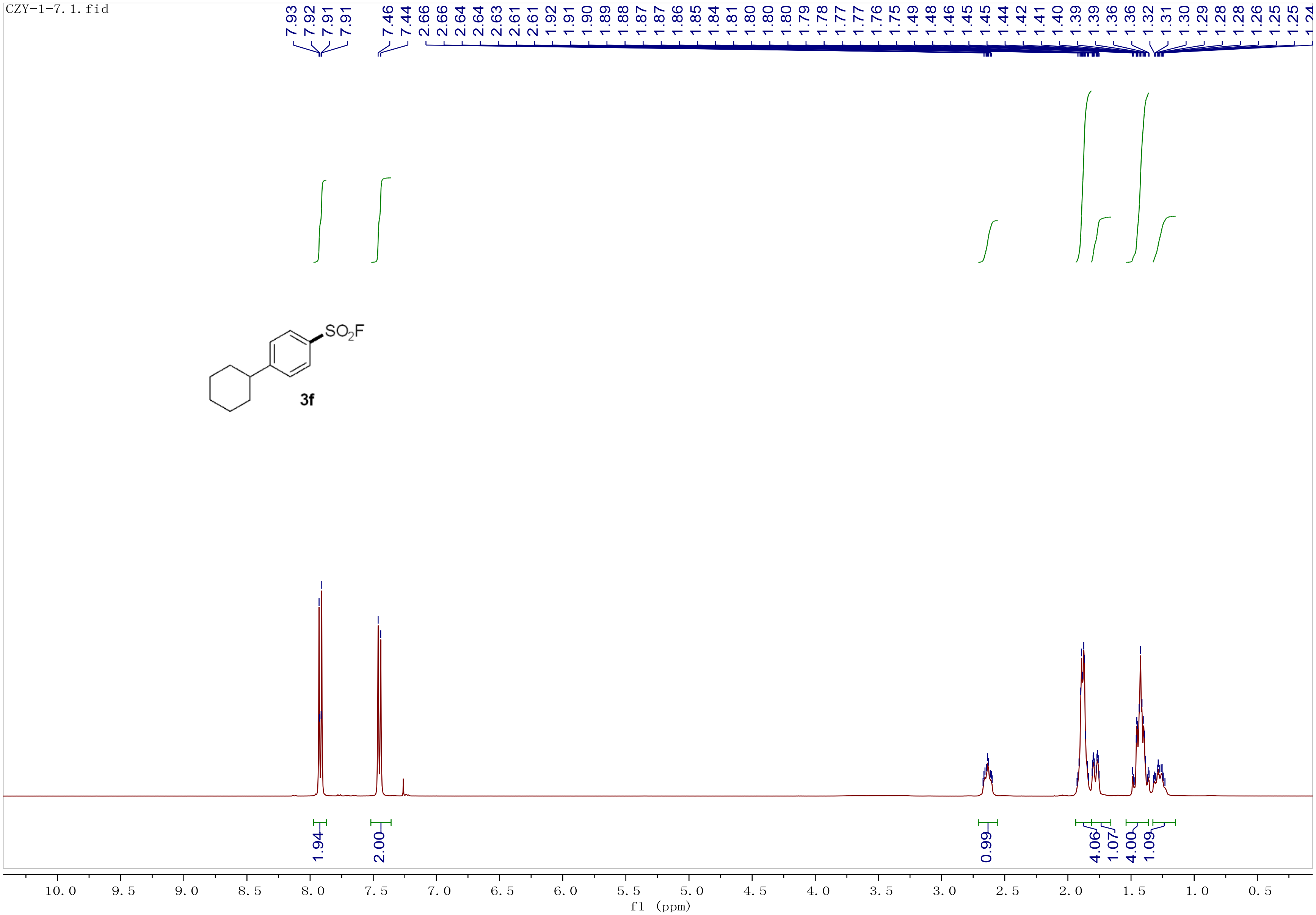

Supplementary Fig. 21 ^{1}H NMR spectrum of compound 3f ($CDCl_3$, 500 MHz, 298K)

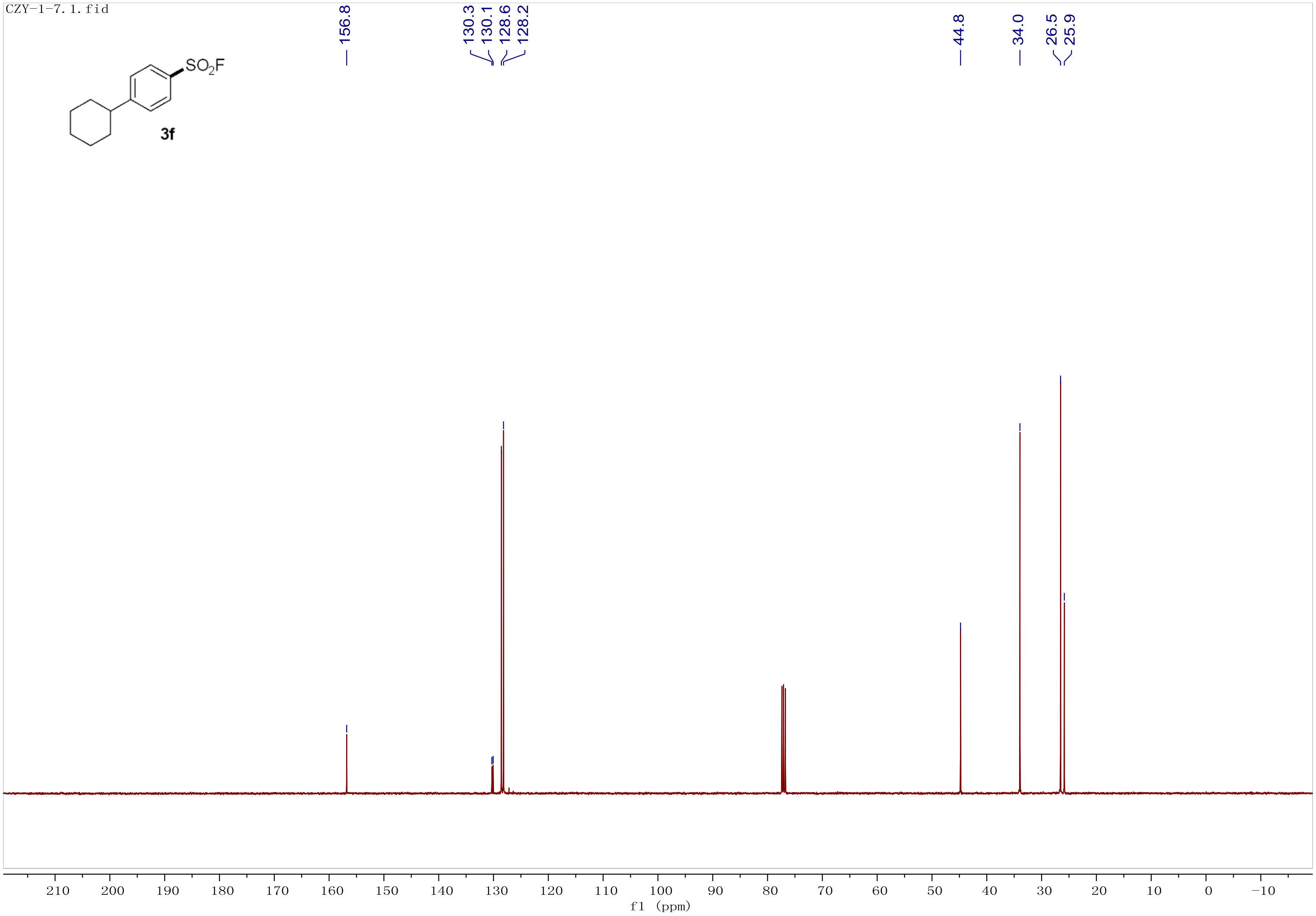

Supplementary Fig. 22 ^{13}C NMR spectrum of compound 3f ($CDCl_3$, 126 MHz, 298K)

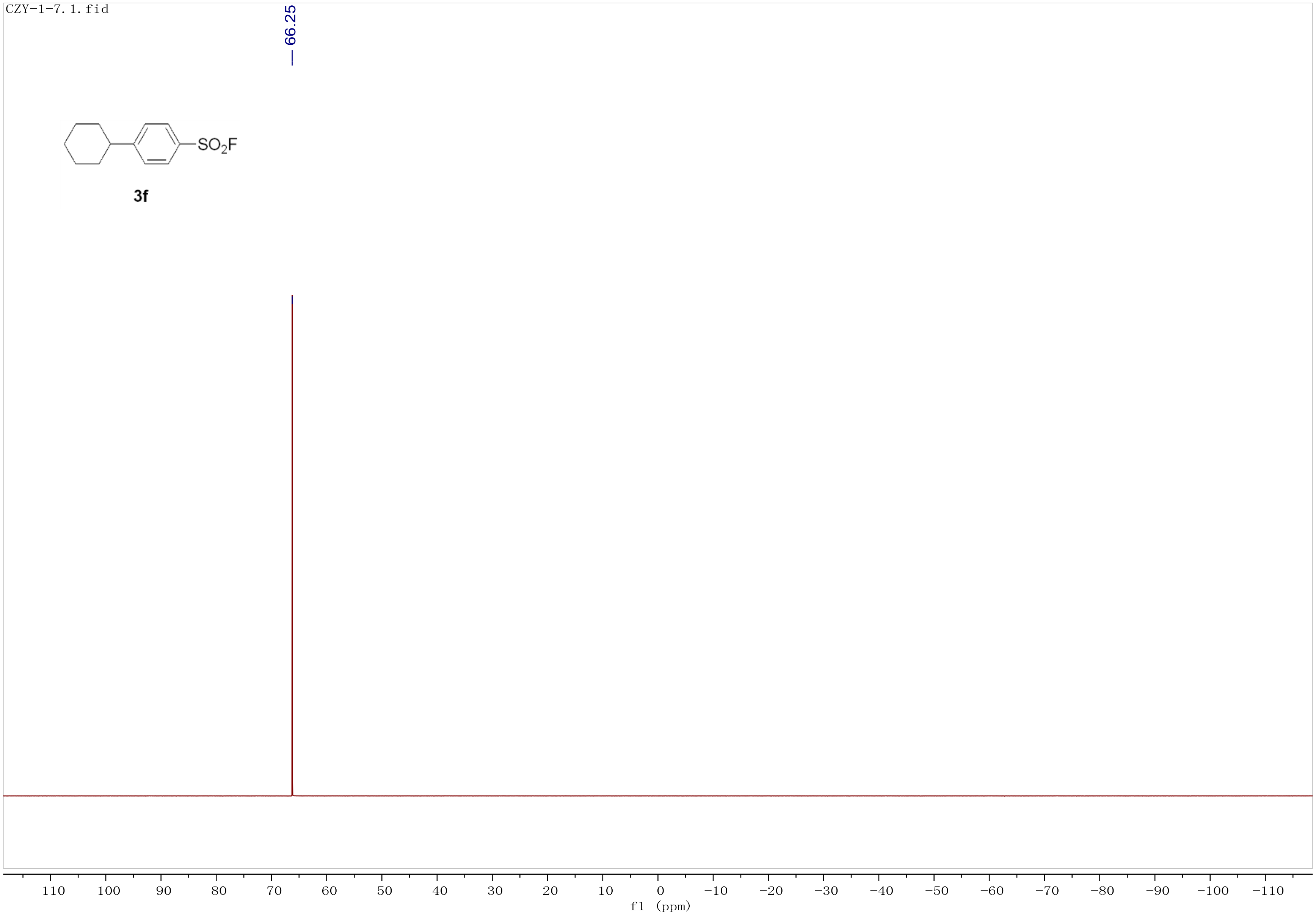

Supplementary Fig. 23 ^{19}F NMR spectrum of compound 3f ($CDCl_3$, 376 MHz, 298K)

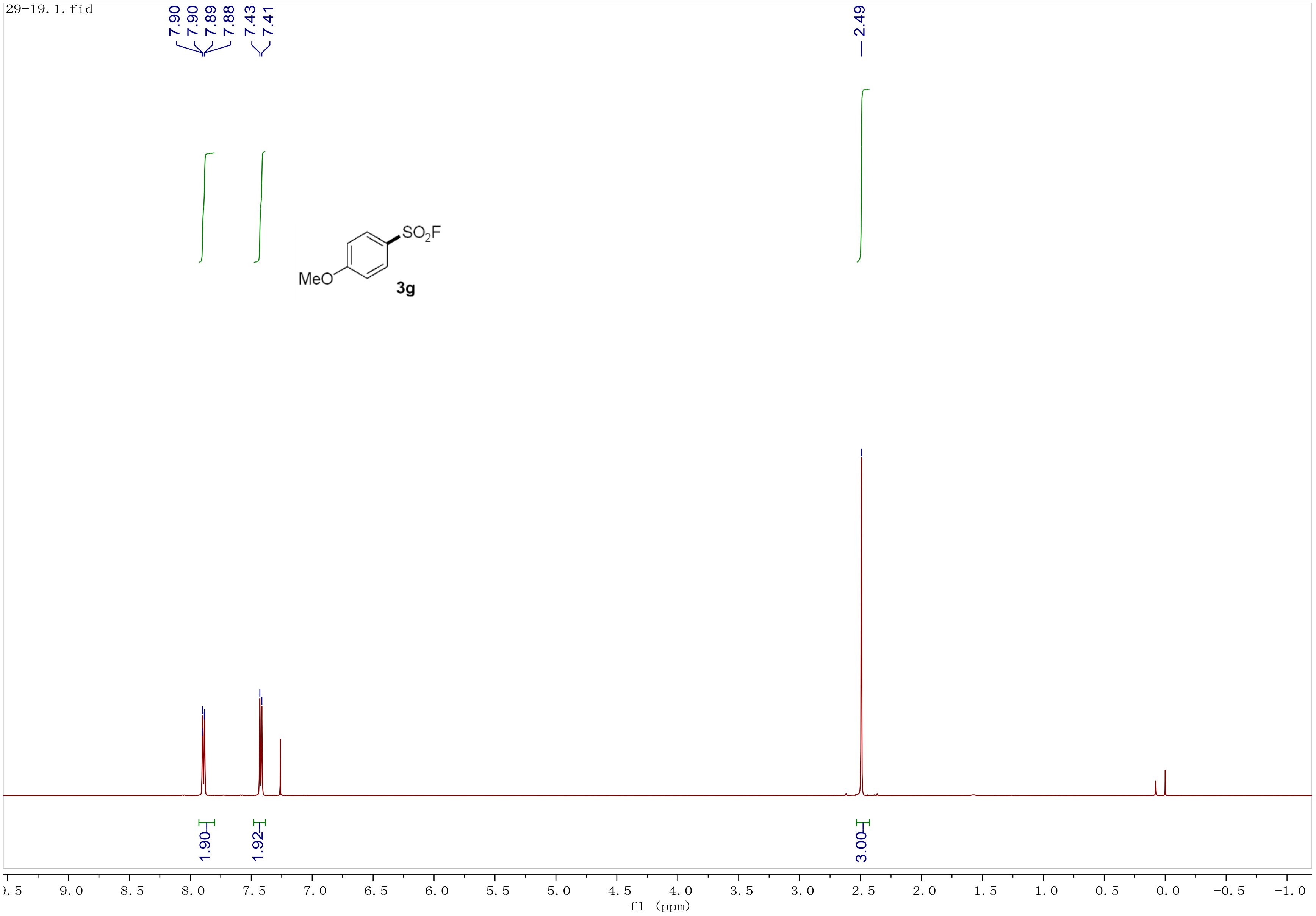

Supplementary Fig. 24 ^{1}H NMR spectrum of compound 3g ($CDCl_3$, 500 MHz, 298K)

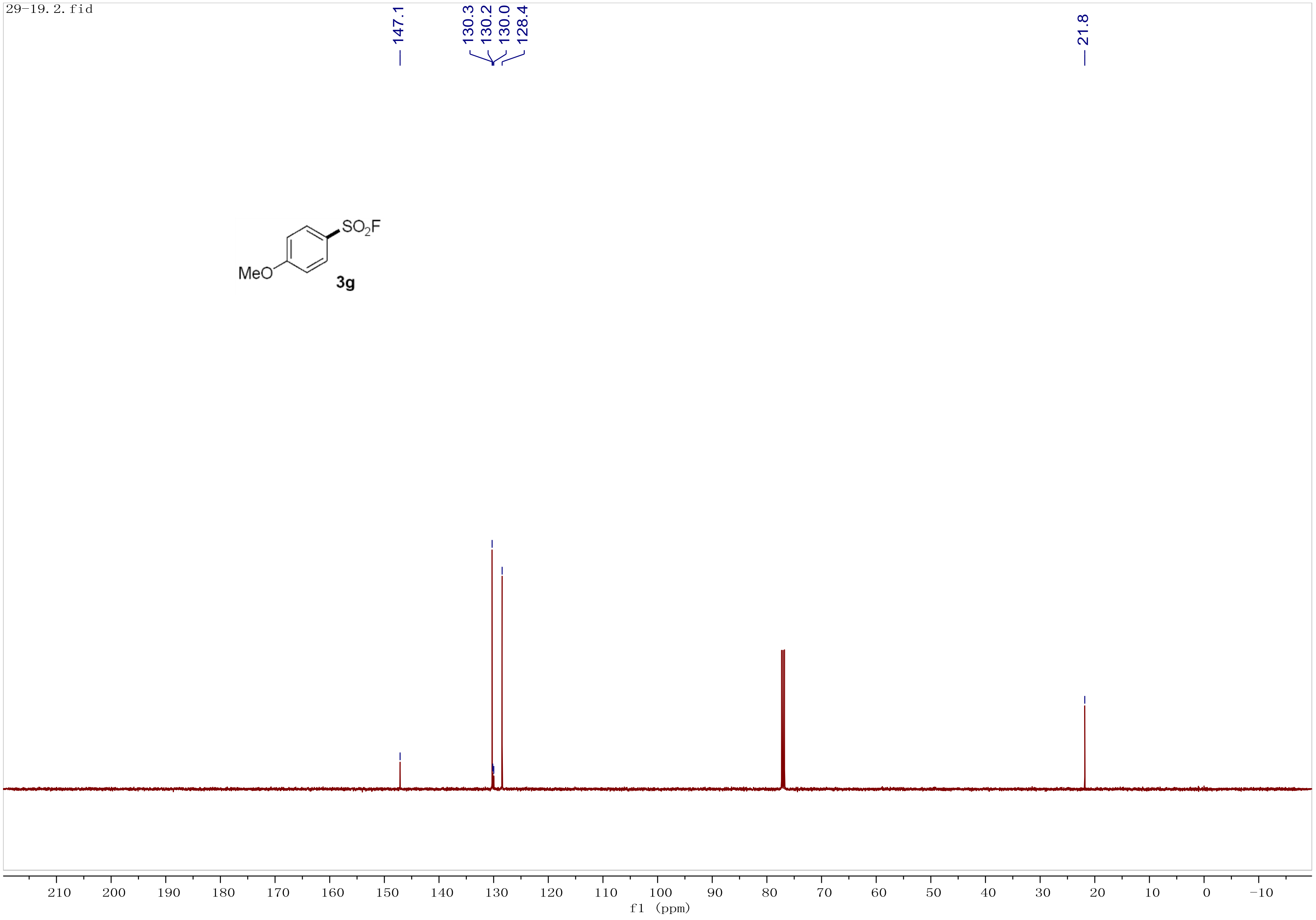

Supplementary Fig. 25 ^{13}C NMR spectrum of compound 3g ($CDCl_3$, 126 MHz, 298K)

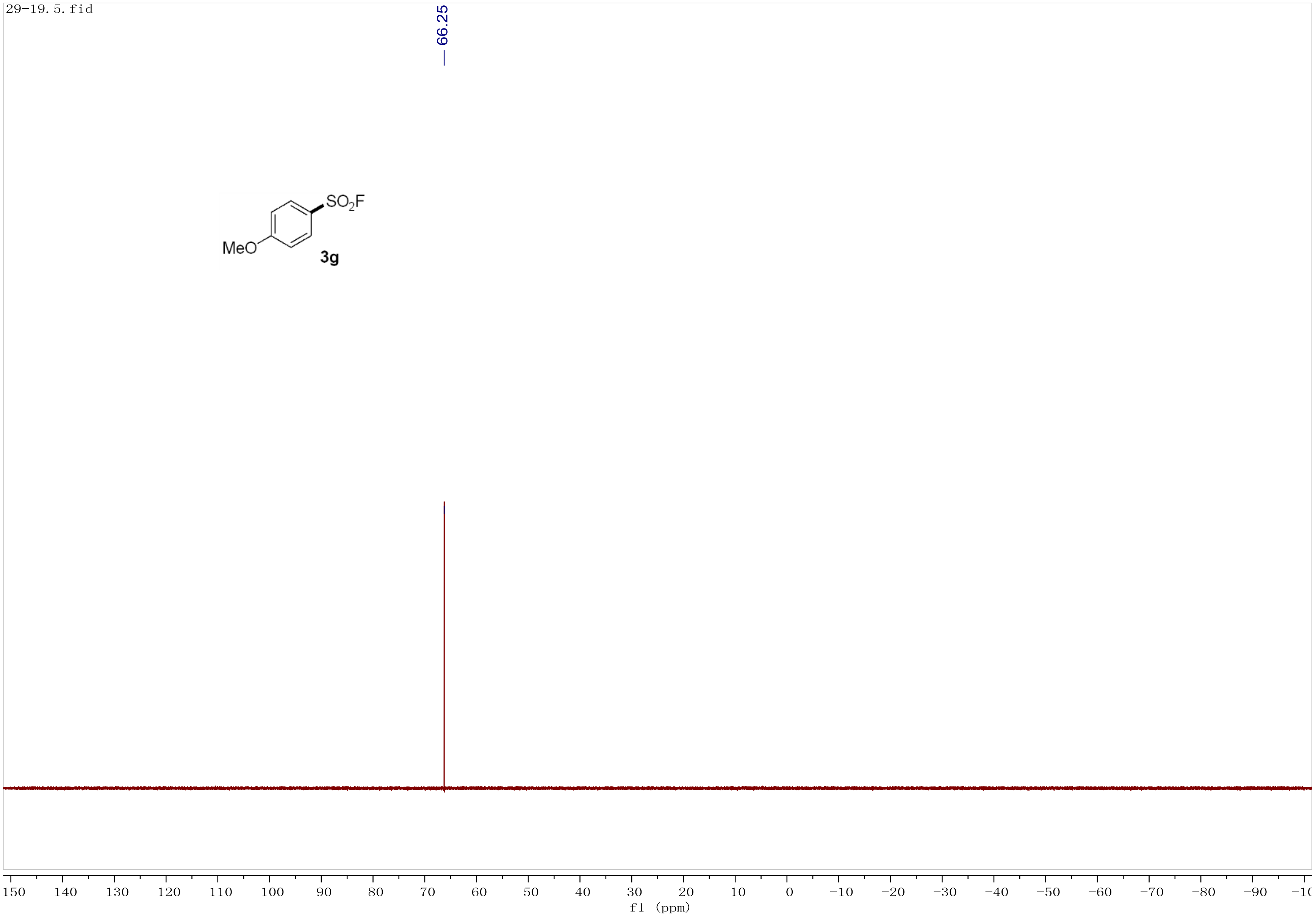

Supplementary Fig. 26 ^{19}F NMR spectrum of compound 3g ($CDCl_3$, 471 MHz, 298K)

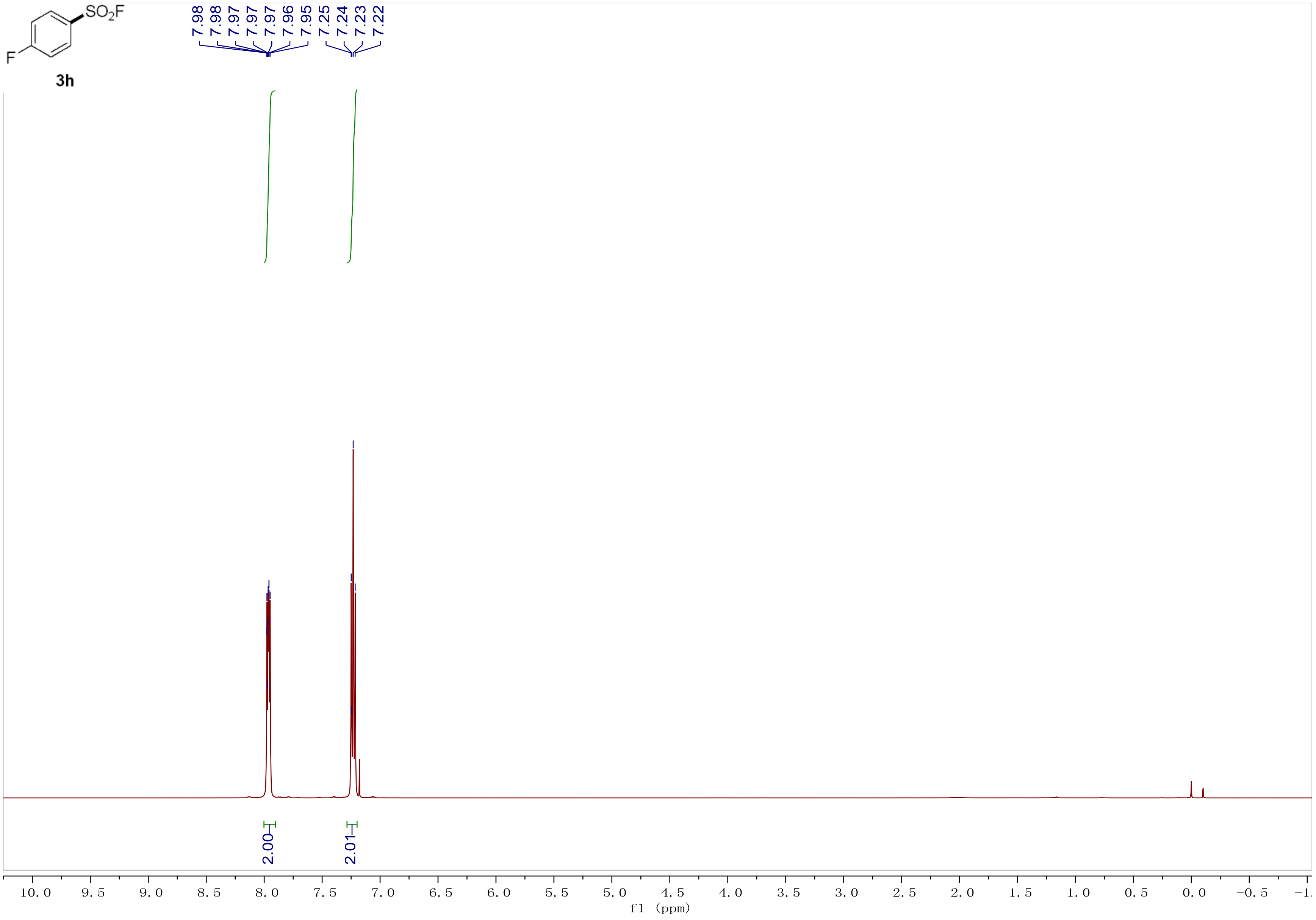

Supplementary Fig. 27 ^{1}H NMR spectrum of compound 3h ($CDCl_3$, 500 MHz, 298K)

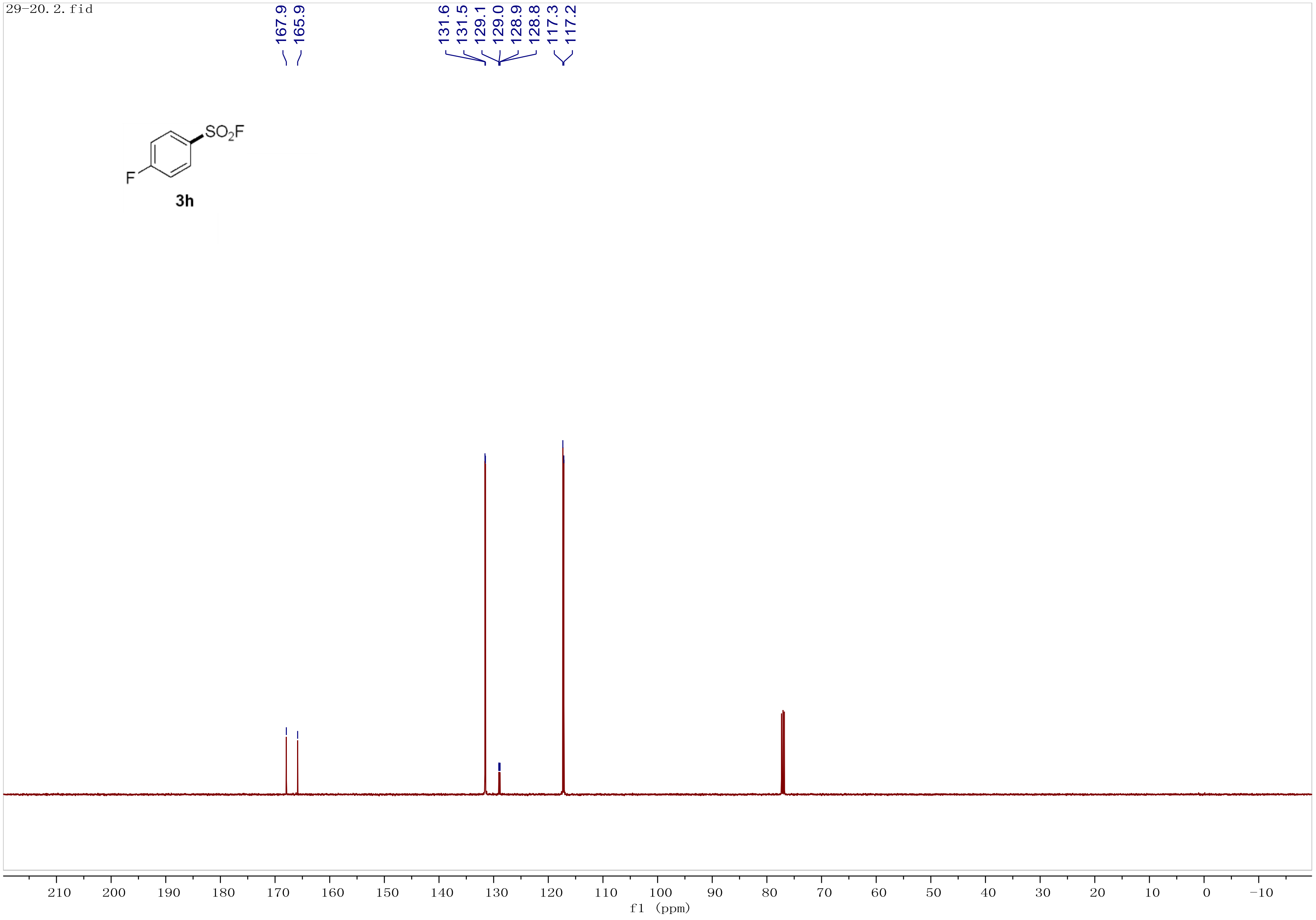

Supplementary Fig. 28 ^{13}C NMR spectrum of compound 3h ($CDCl_3$, 126 MHz, 298K)

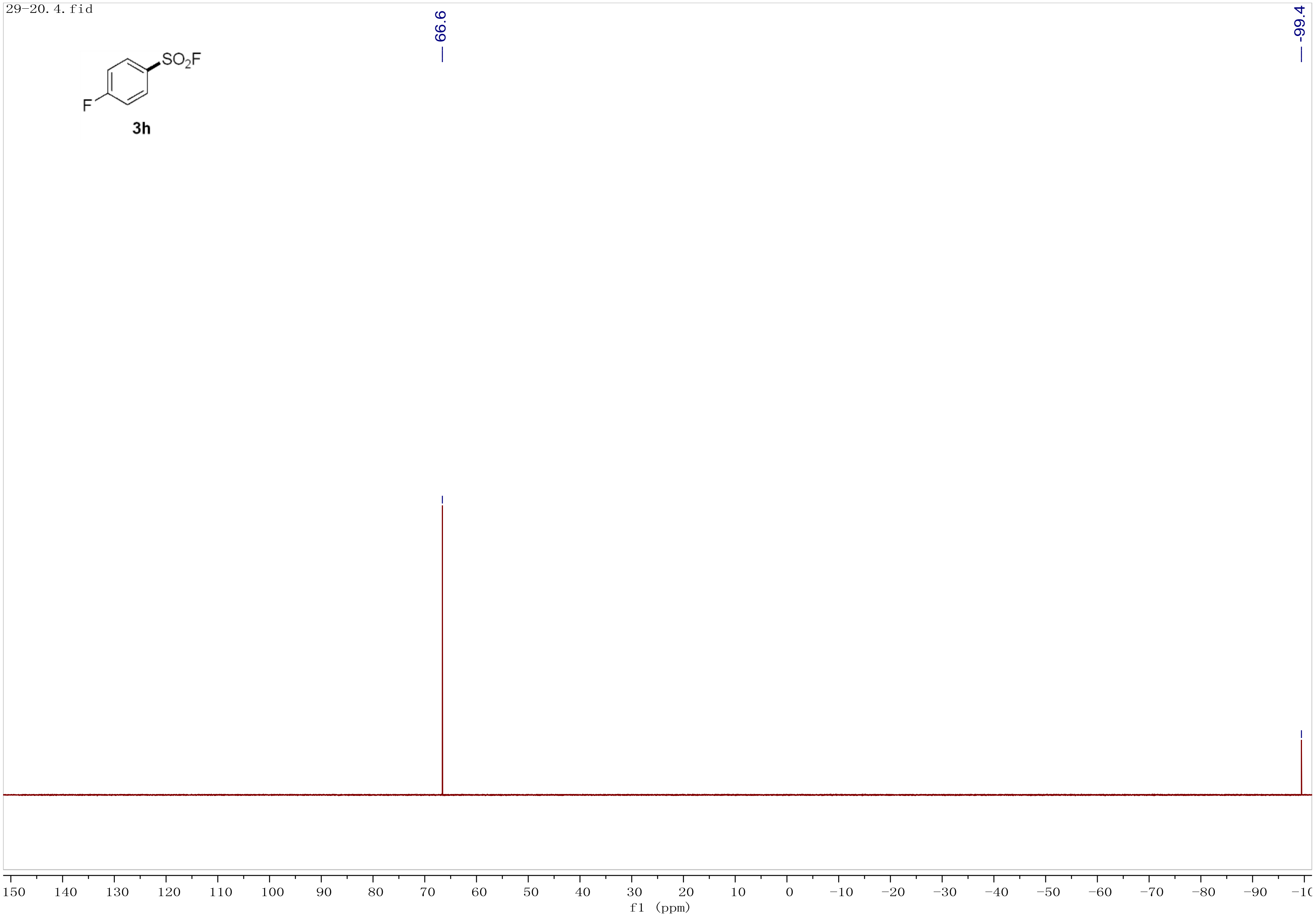

Supplementary Fig. 29 ^{19}F NMR spectrum of compound 3h ($CDCl_3$, 471 MHz, 298K)

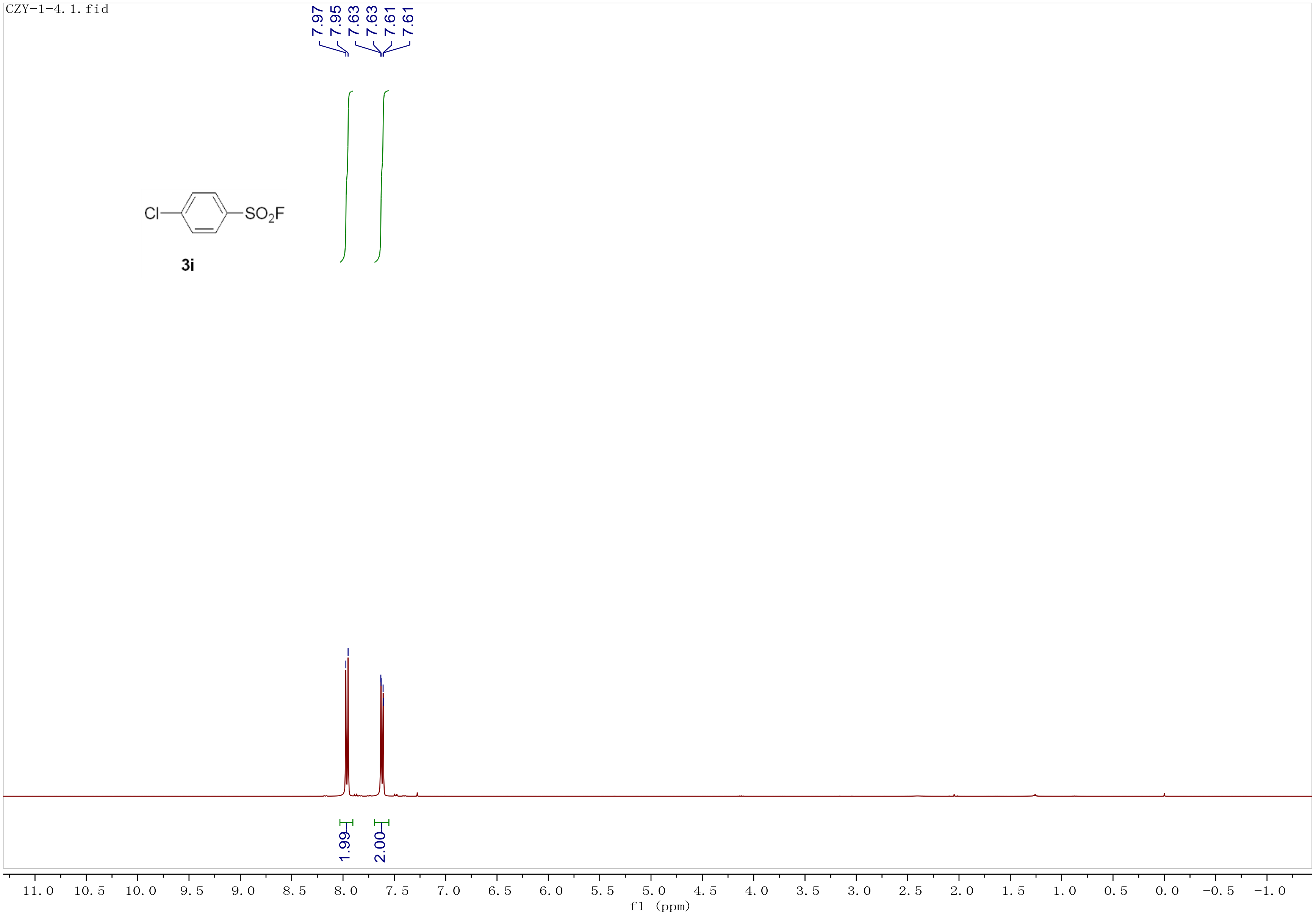

Supplementary Fig. 30 ^{1}H NMR spectrum of compound 3i ($CDCl_3$, 400 MHz, 298K)

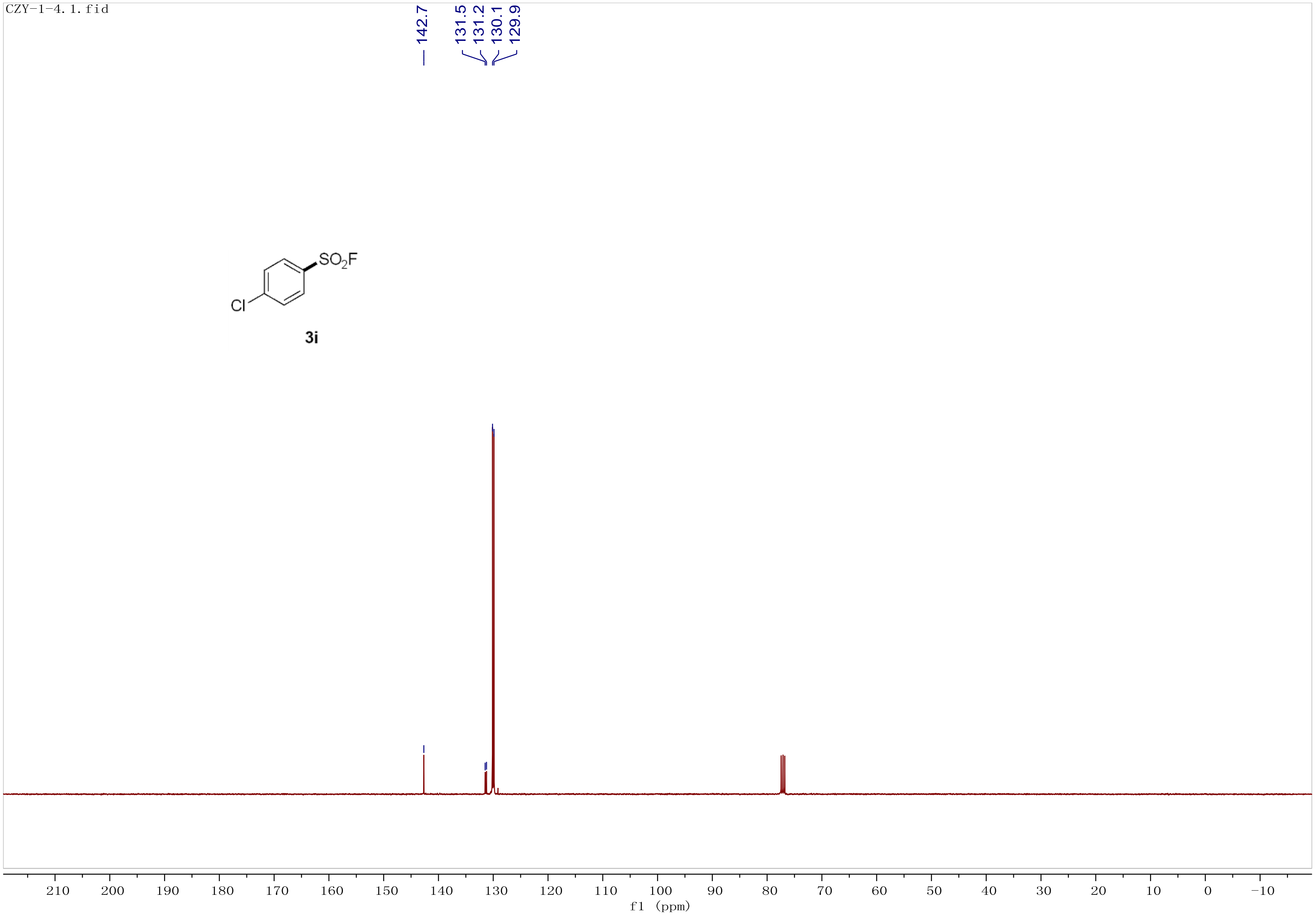

Supplementary Fig. 31 ^{13}C NMR spectrum of compound 3i ($CDCl_3$, 101 MHz, 298K)

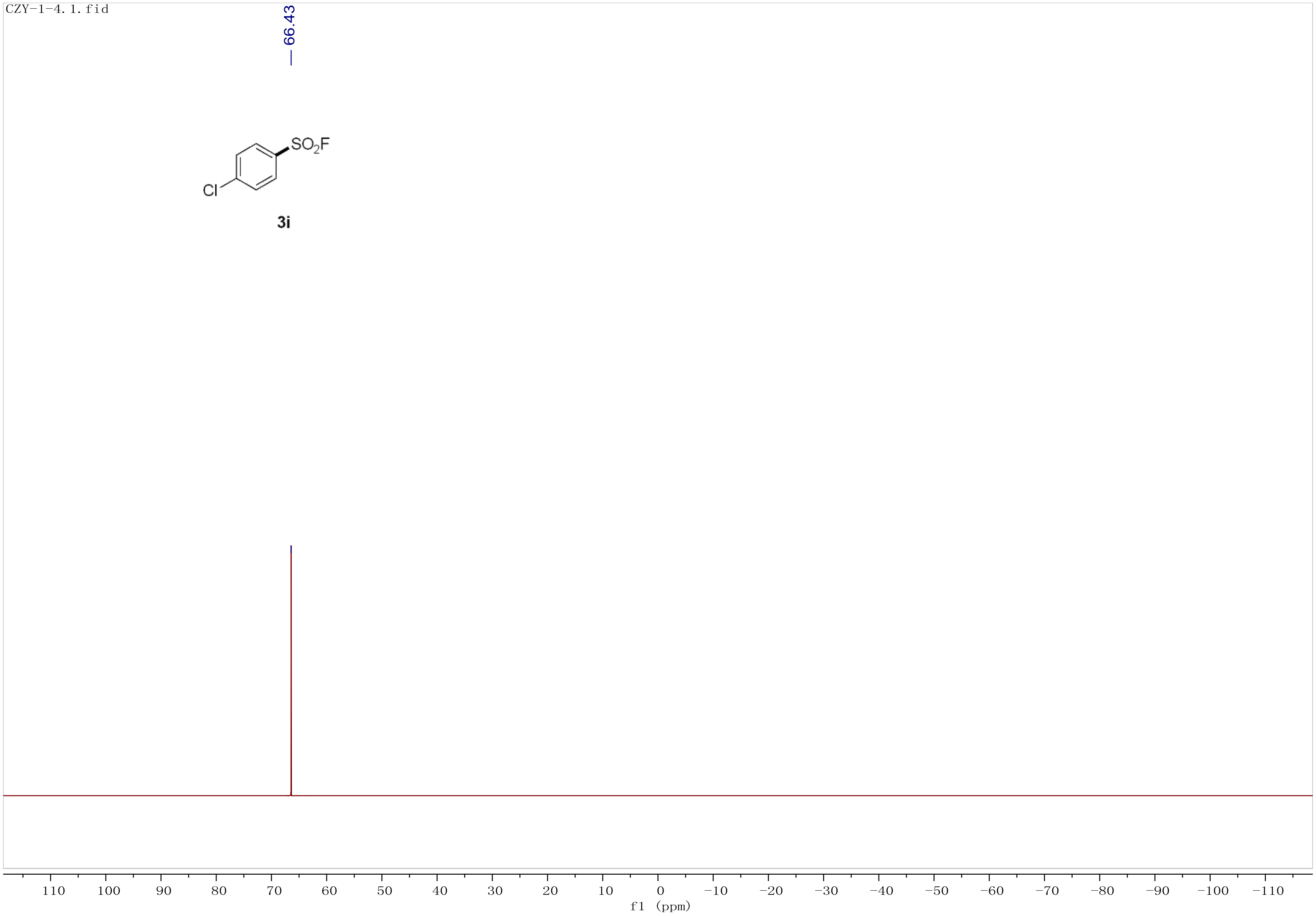

Supplementary Fig. 32 ^{19}F NMR spectrum of compound 3i ($CDCl_3$, 376 MHz, 298K)

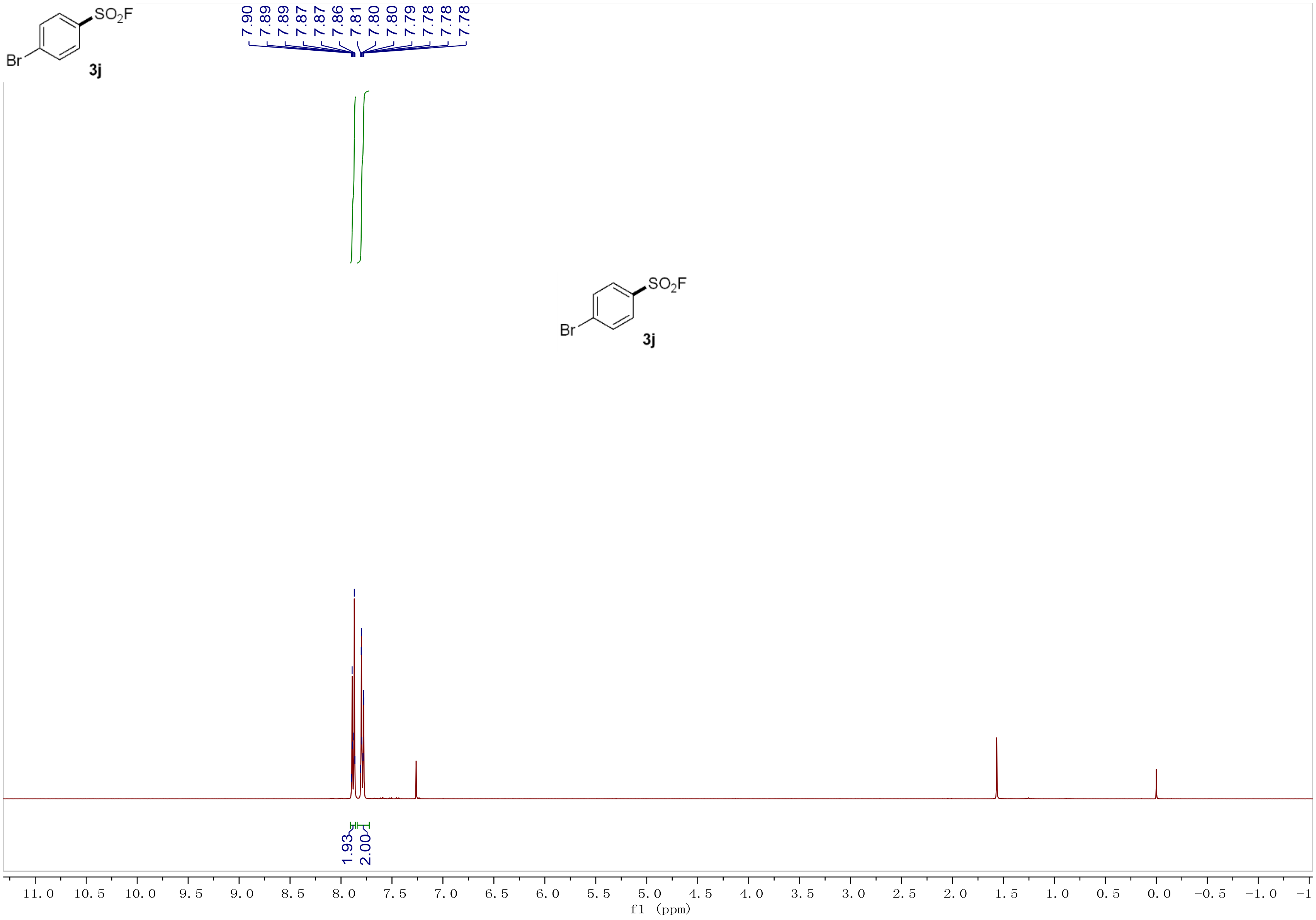

Supplementary Fig. 33 ^{1}H NMR spectrum of compound 3j ($CDCl_3$, 400 MHz, 298K)

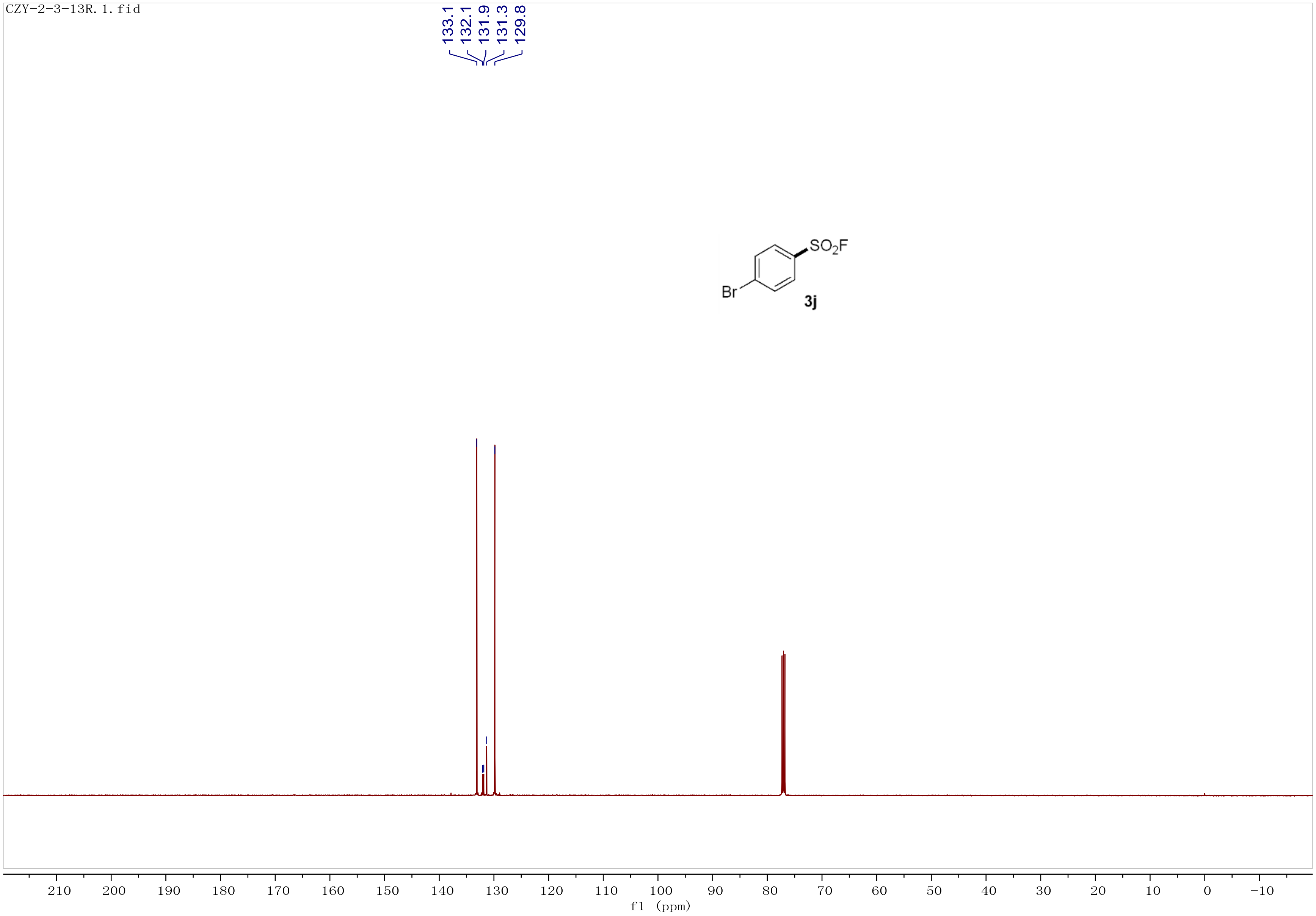

Supplementary Fig. 34 ^{13}C NMR spectrum of compound 3j ($CDCl_3$, 126 MHz, 298K)

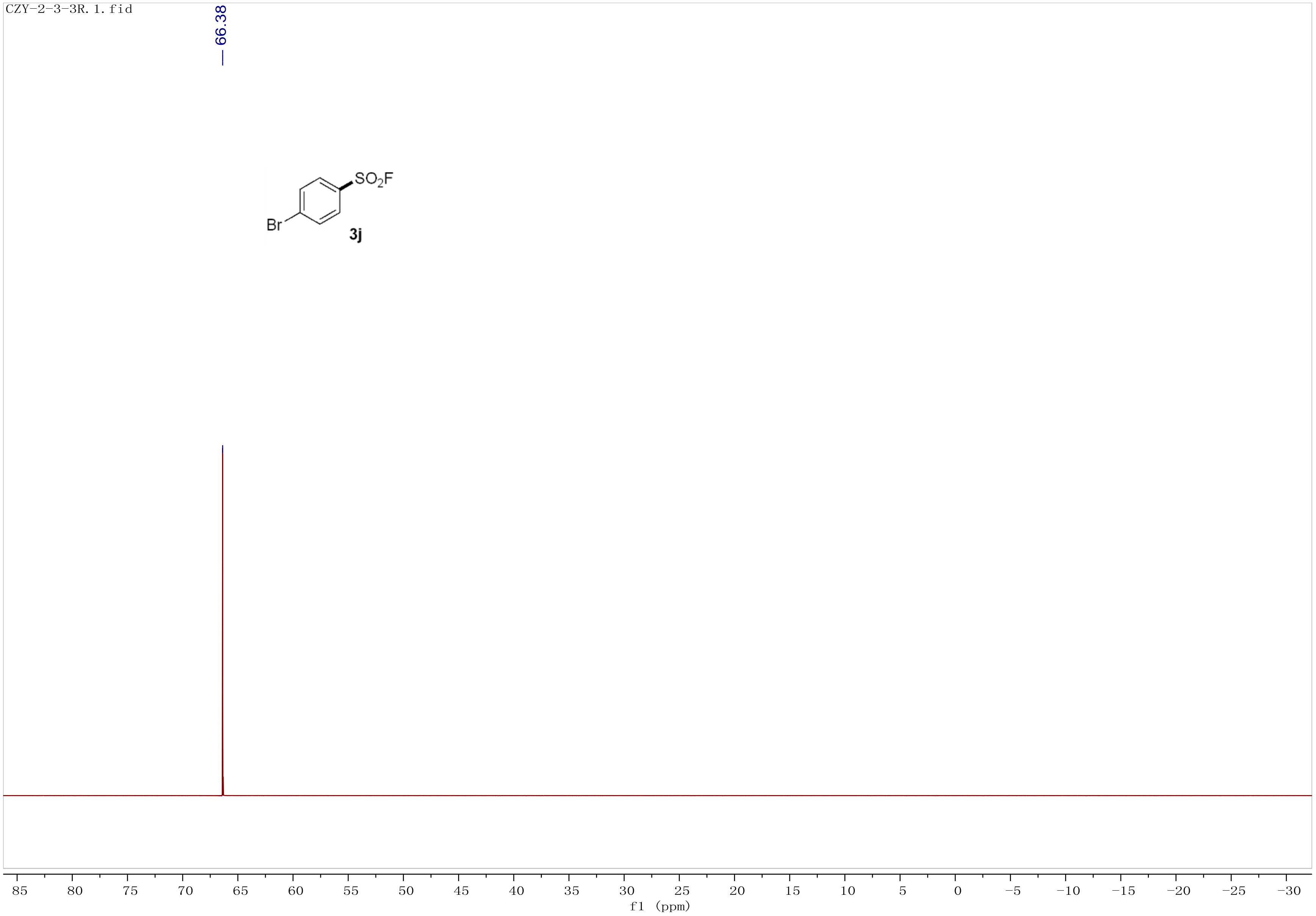

Supplementary Fig. 35 ^{19}F NMR spectrum of compound 3j ($CDCl_3$, 376 MHz, 298K)

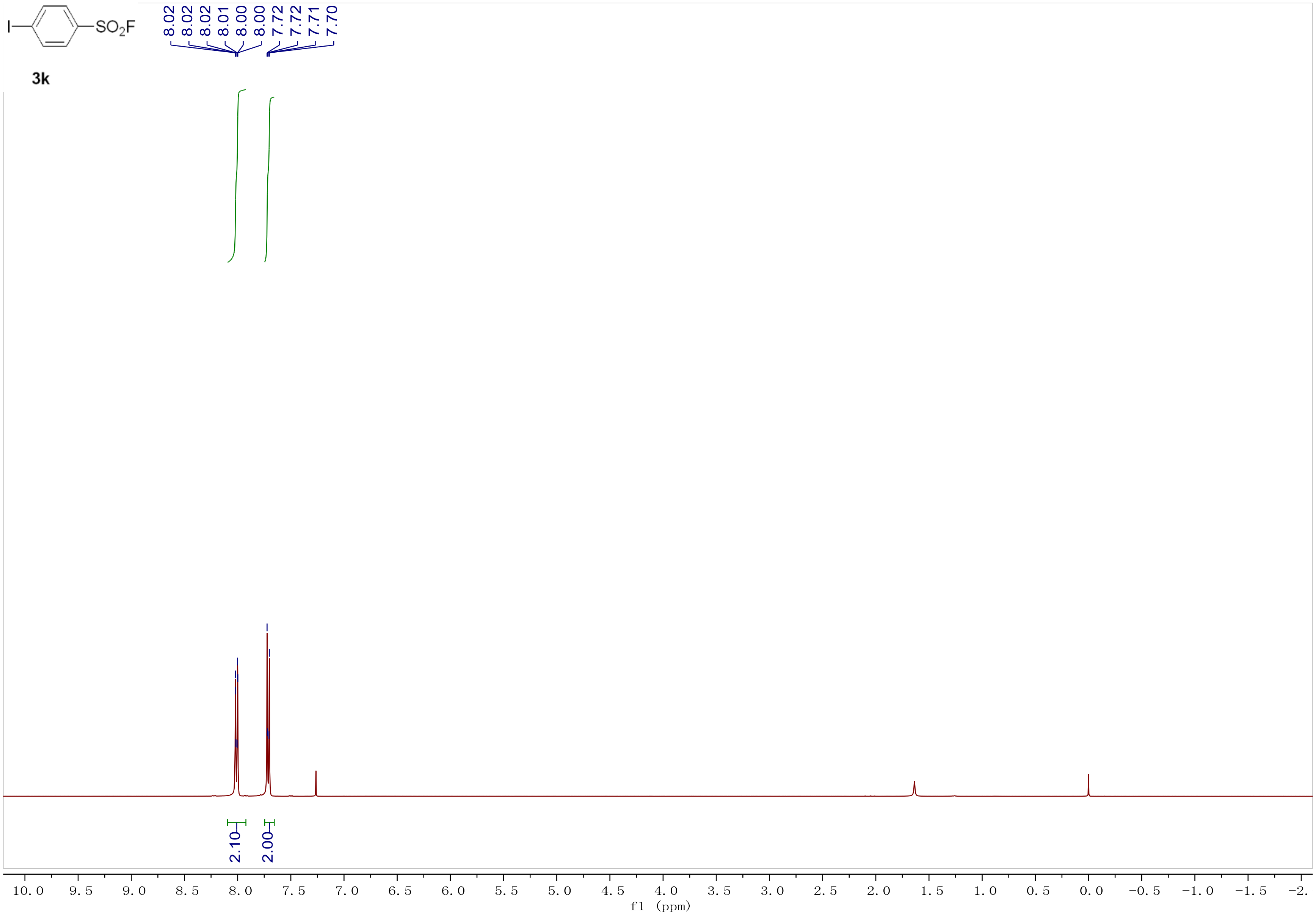

Supplementary Fig. 3k ^{1}H NMR spectrum of compound 3k ($CDCl_3$, 400 MHz, 298K)

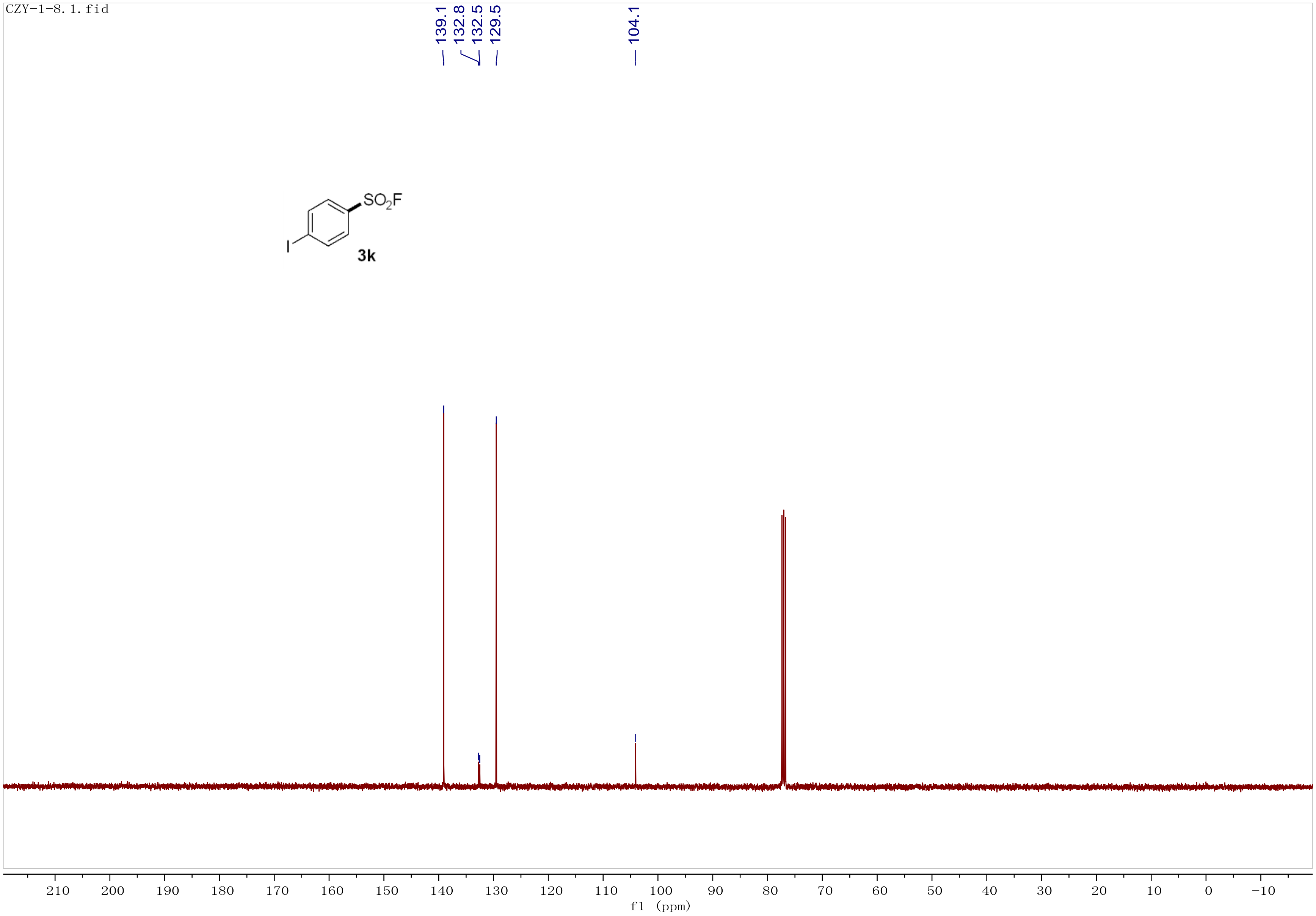

Supplementary Fig. 37 ^{13}C NMR spectrum of compound 3k ($CDCl_3$, 101 MHz, 298K)

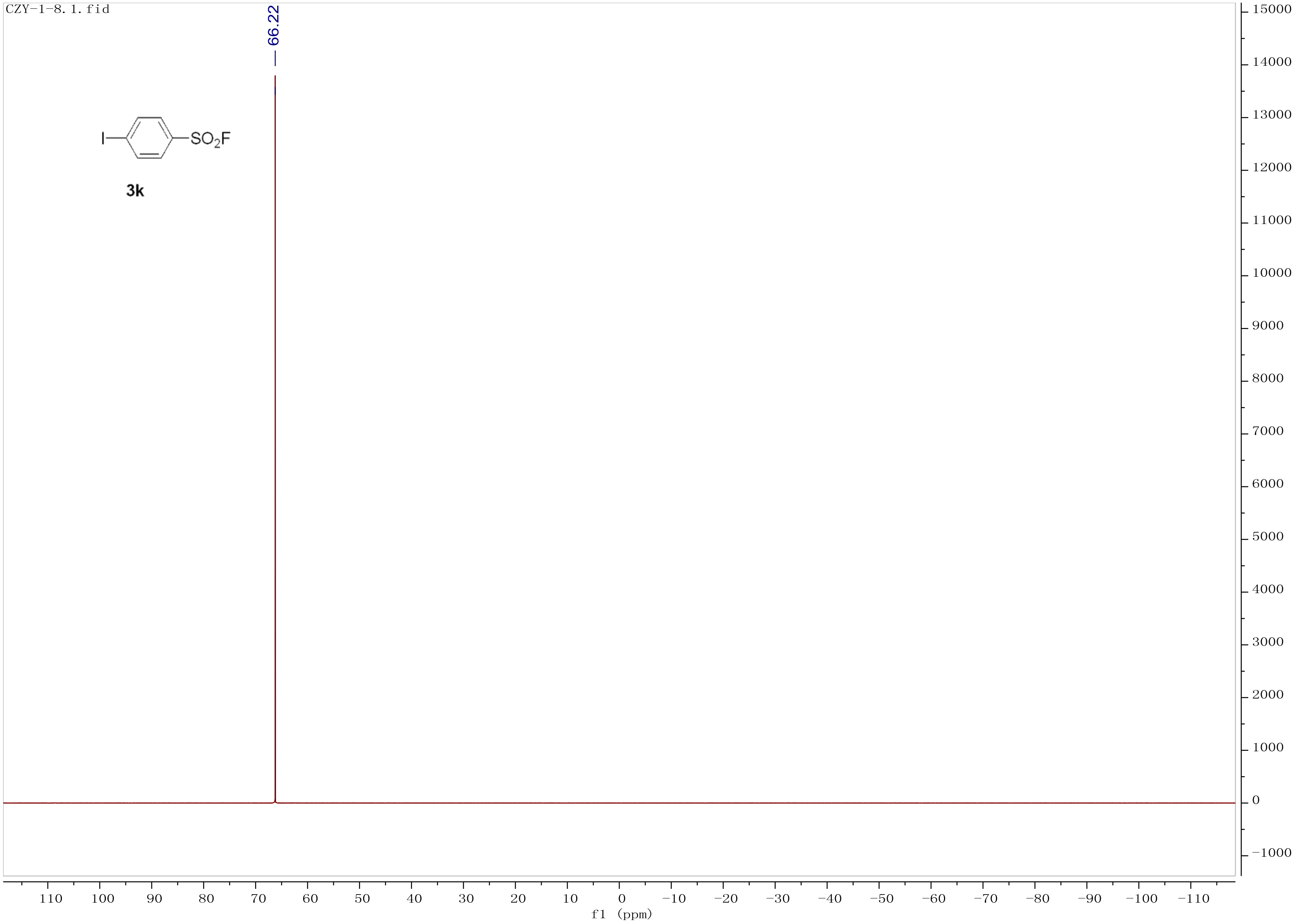

Supplementary Fig. 38 ^{19}F NMR spectrum of compound 3k ($CDCl_3$, 376 MHz, 298K)

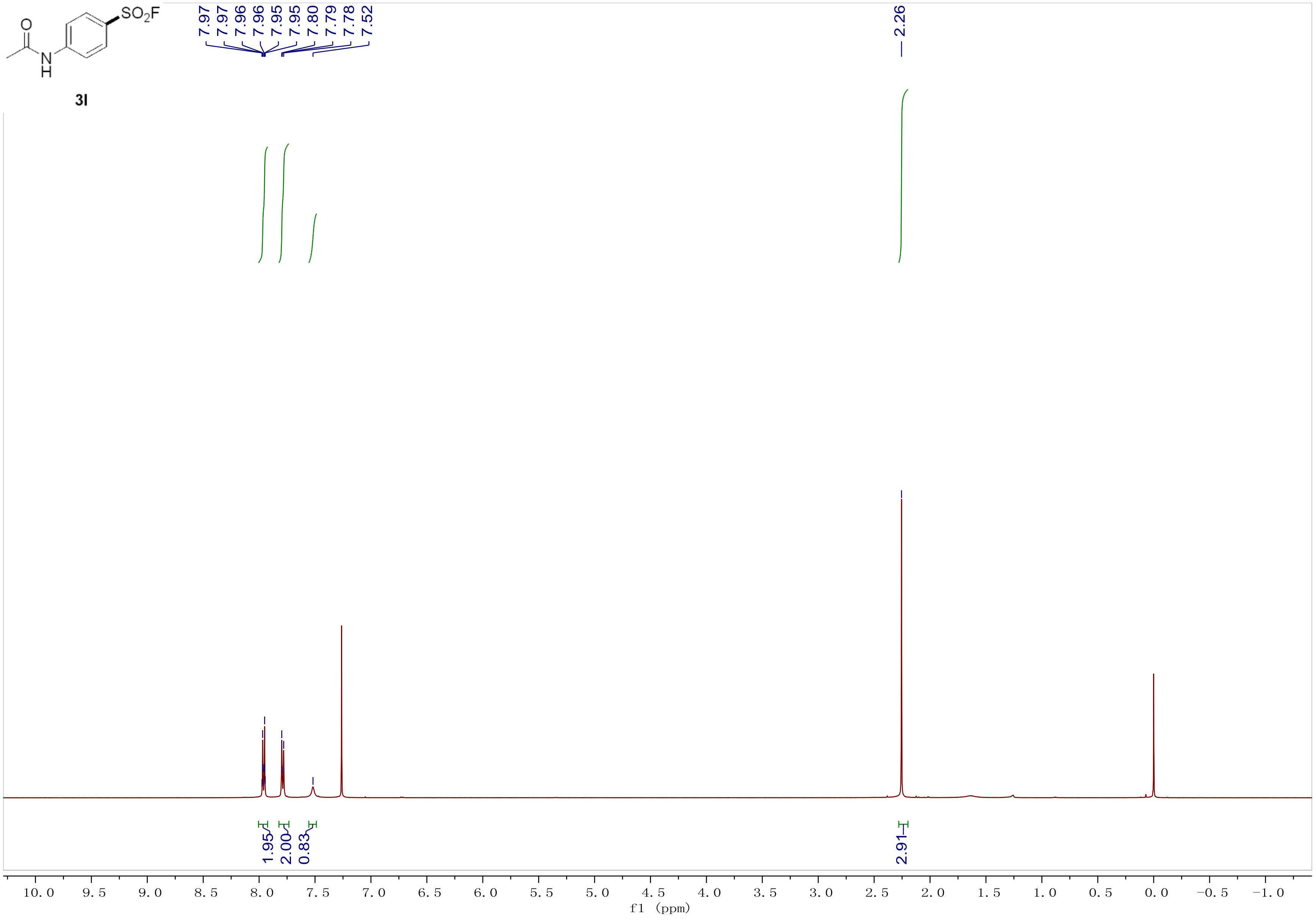

Supplementary Fig. 39 ^{1}H NMR spectrum of compound 3l ($CDCl_3$, 500 MHz, 298K)

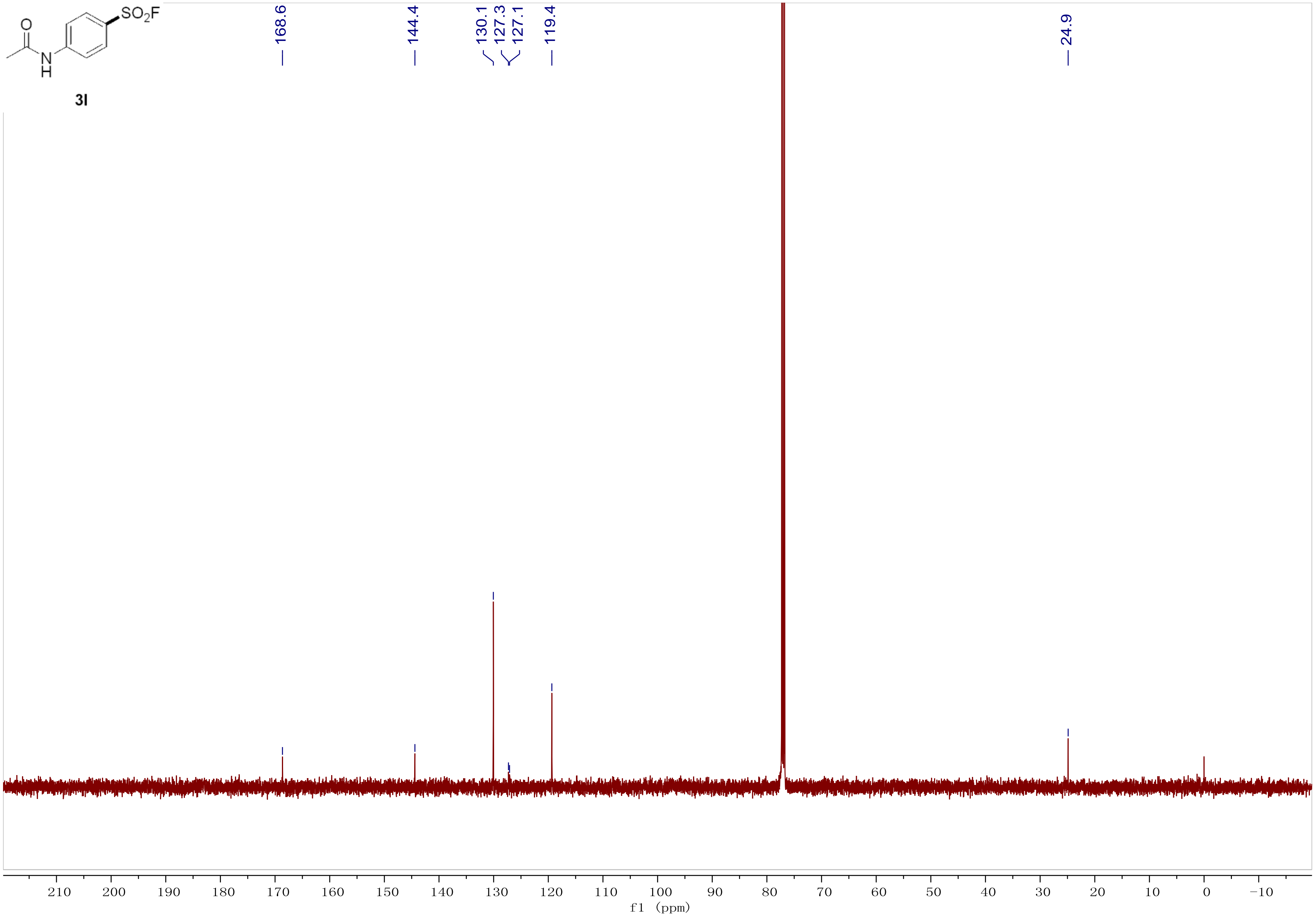

Supplementary Fig. 40 ^{13}C NMR spectrum of compound 3l ($CDCl_3$, 126 MHz, 298K)

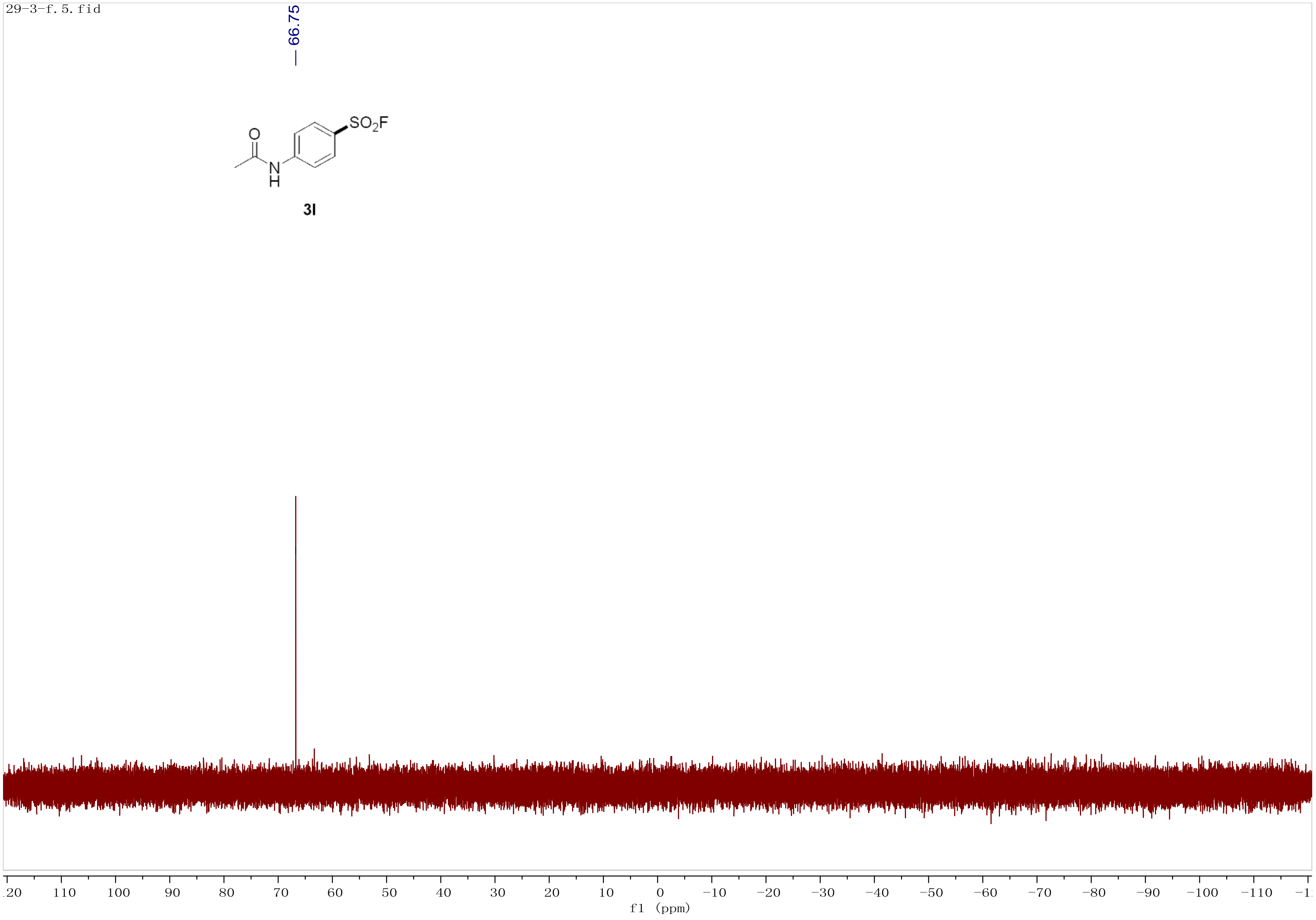

Supplementary Fig. 41 ^{19}**F NMR spectrum of compound 3l (CDCl$_3$, 471 MHz, 298K)**

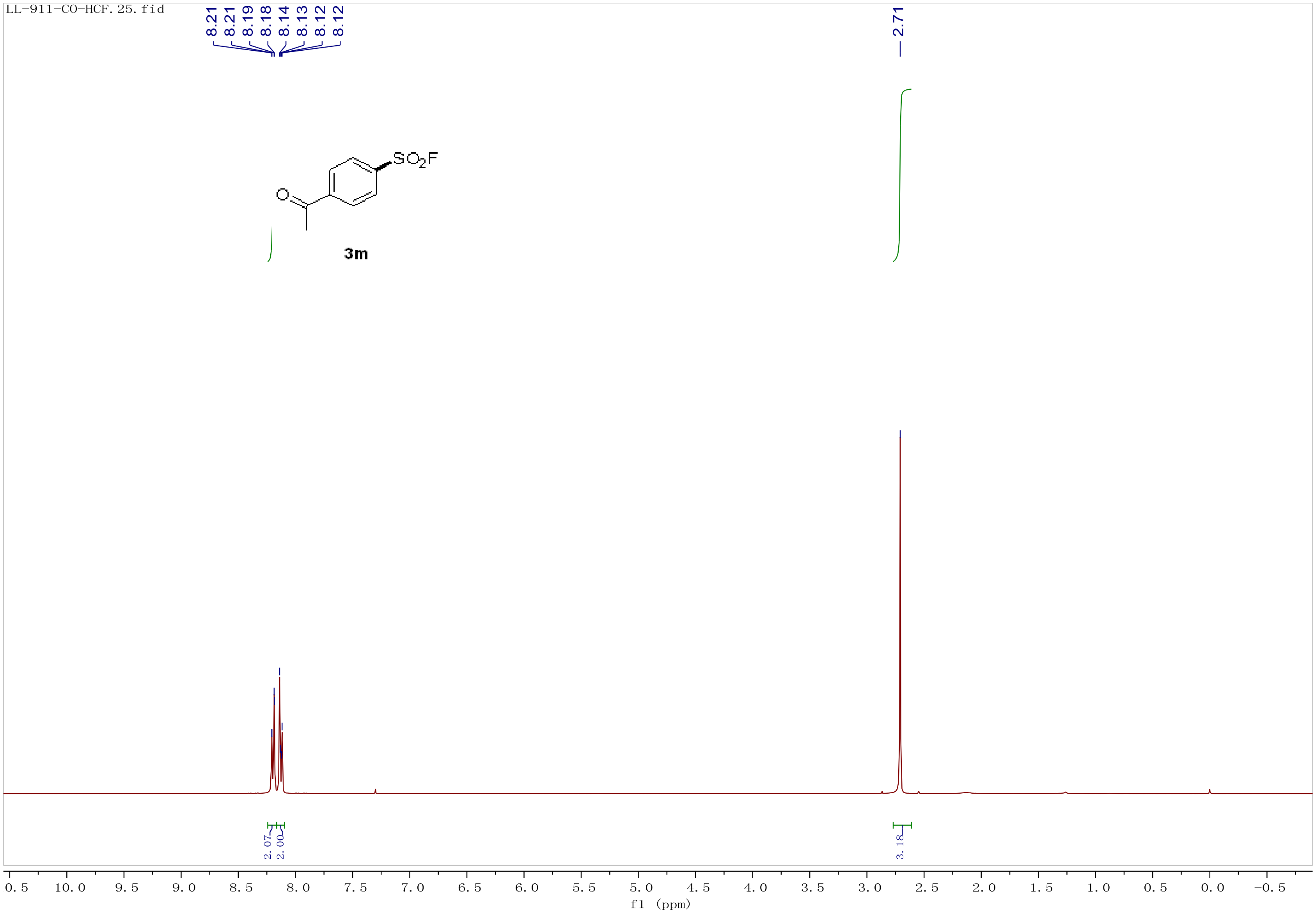

Supplementary Fig. 42 ^{1}H NMR spectrum of compound 3m ($CDCl_3$, 400 MHz, 298K)

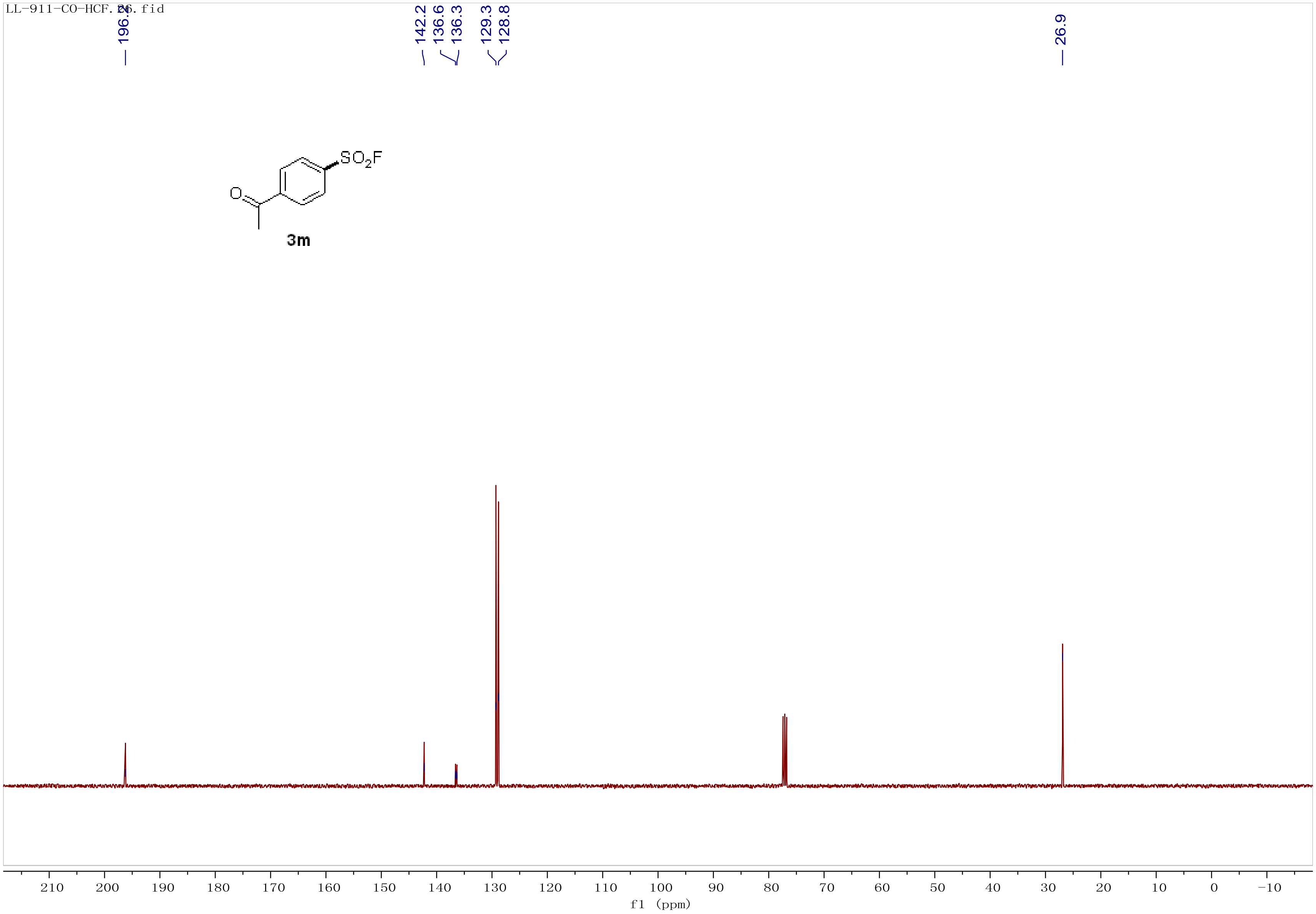

Supplementary Fig. 43 ^{13}C NMR spectrum of compound 3m ($CDCl_3$, 101 MHz, 298K)

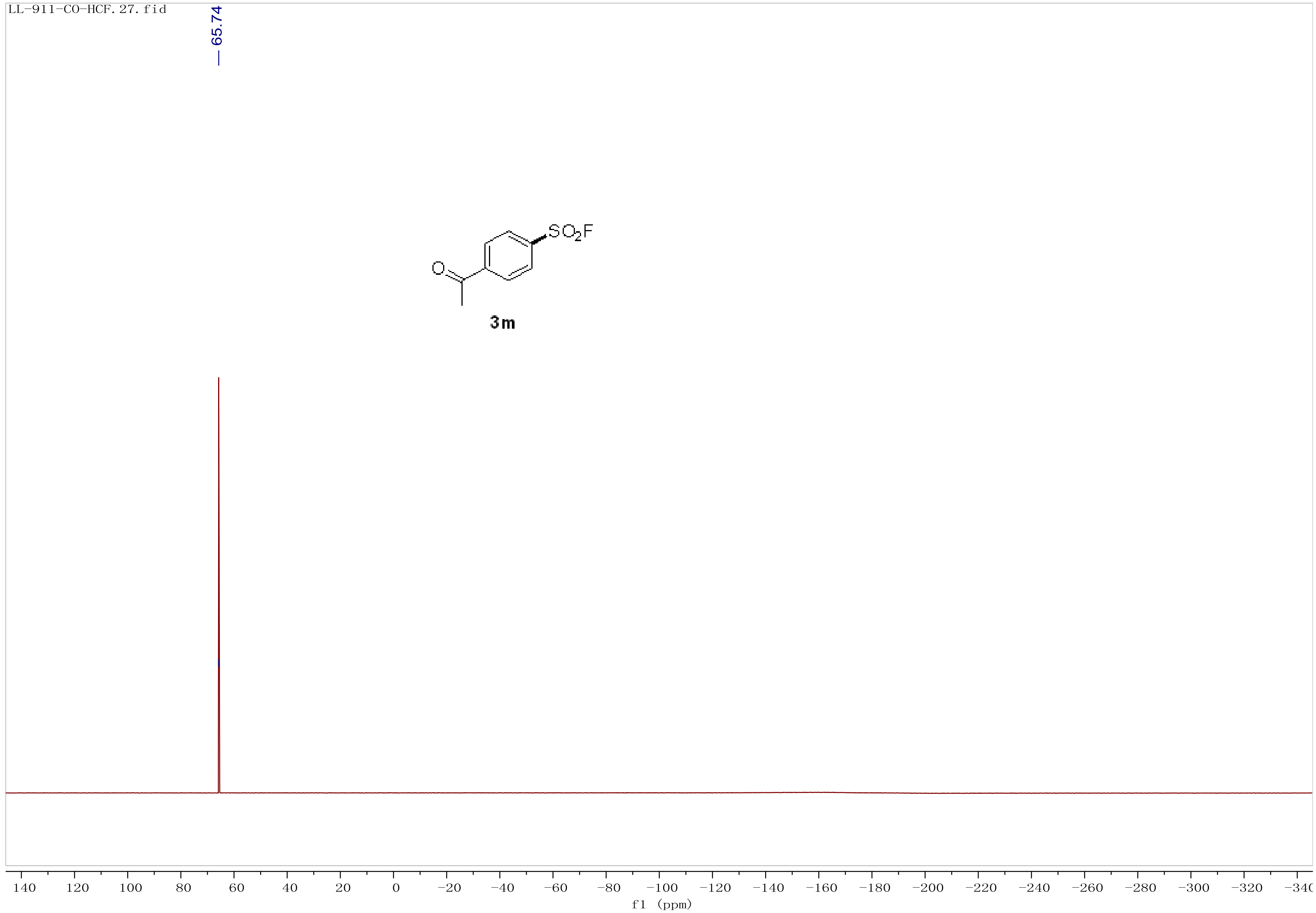

Supplementary Fig. 44 ^{19}F NMR spectrum of compound 3m ($CDCl_3$, 376 MHz, 298K)

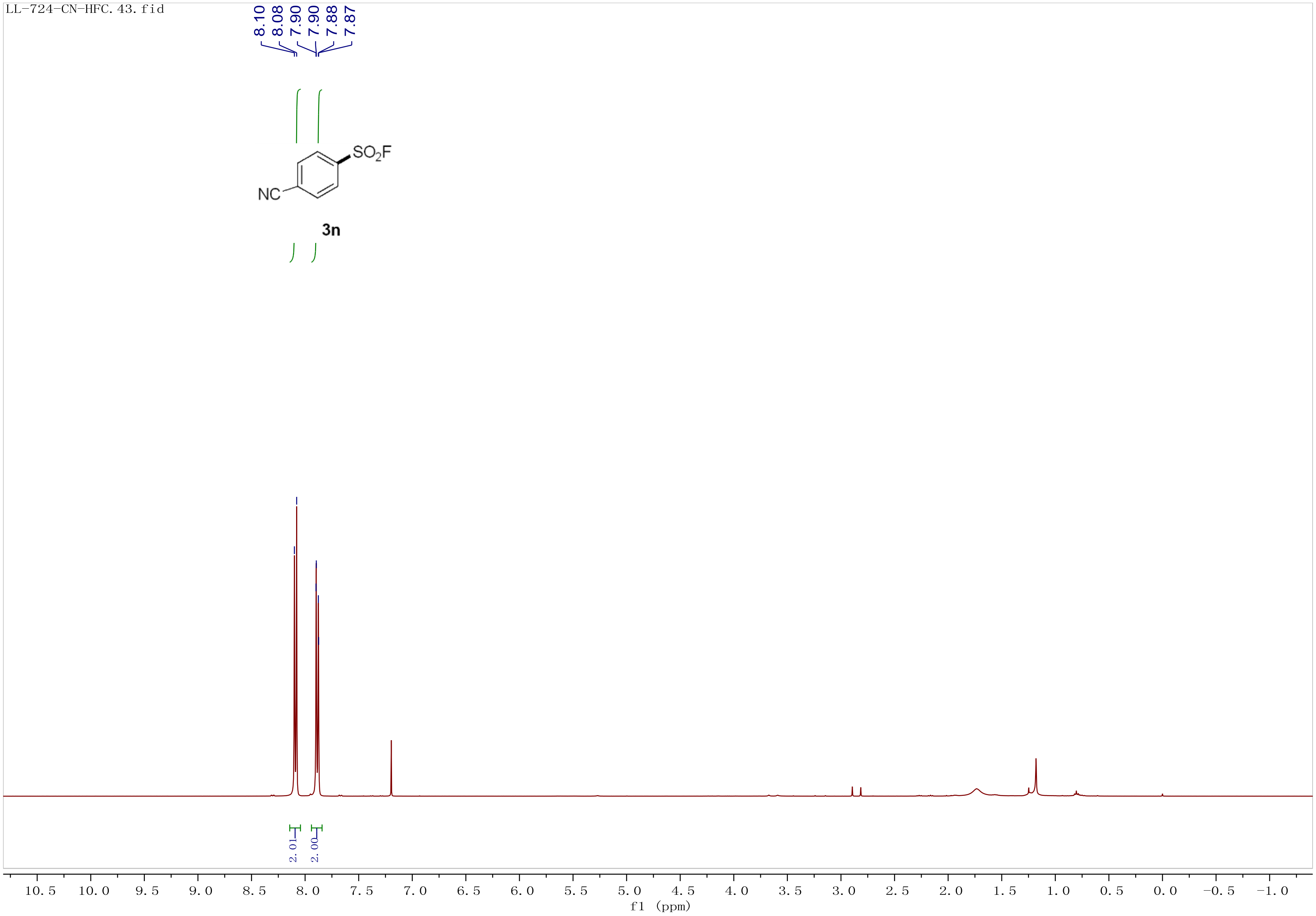

Supplementary Fig. 45 ^{1}H NMR spectrum of compound 3n ($CDCl_3$, 400 MHz, 298K)

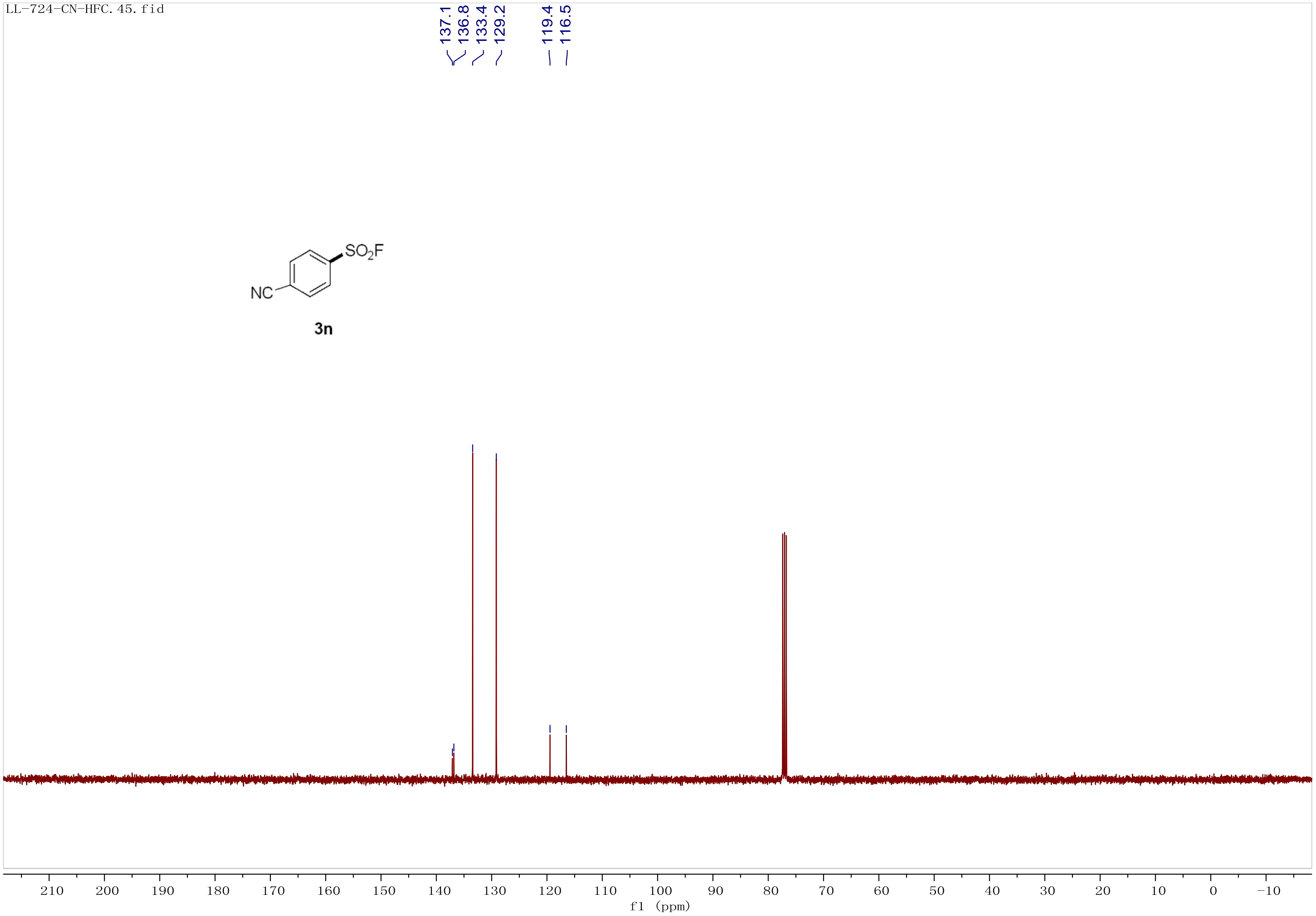

Supplementary Fig. 46 ^{13}C NMR spectrum of compound 3n ($CDCl_3$, 101 MHz, 298K)

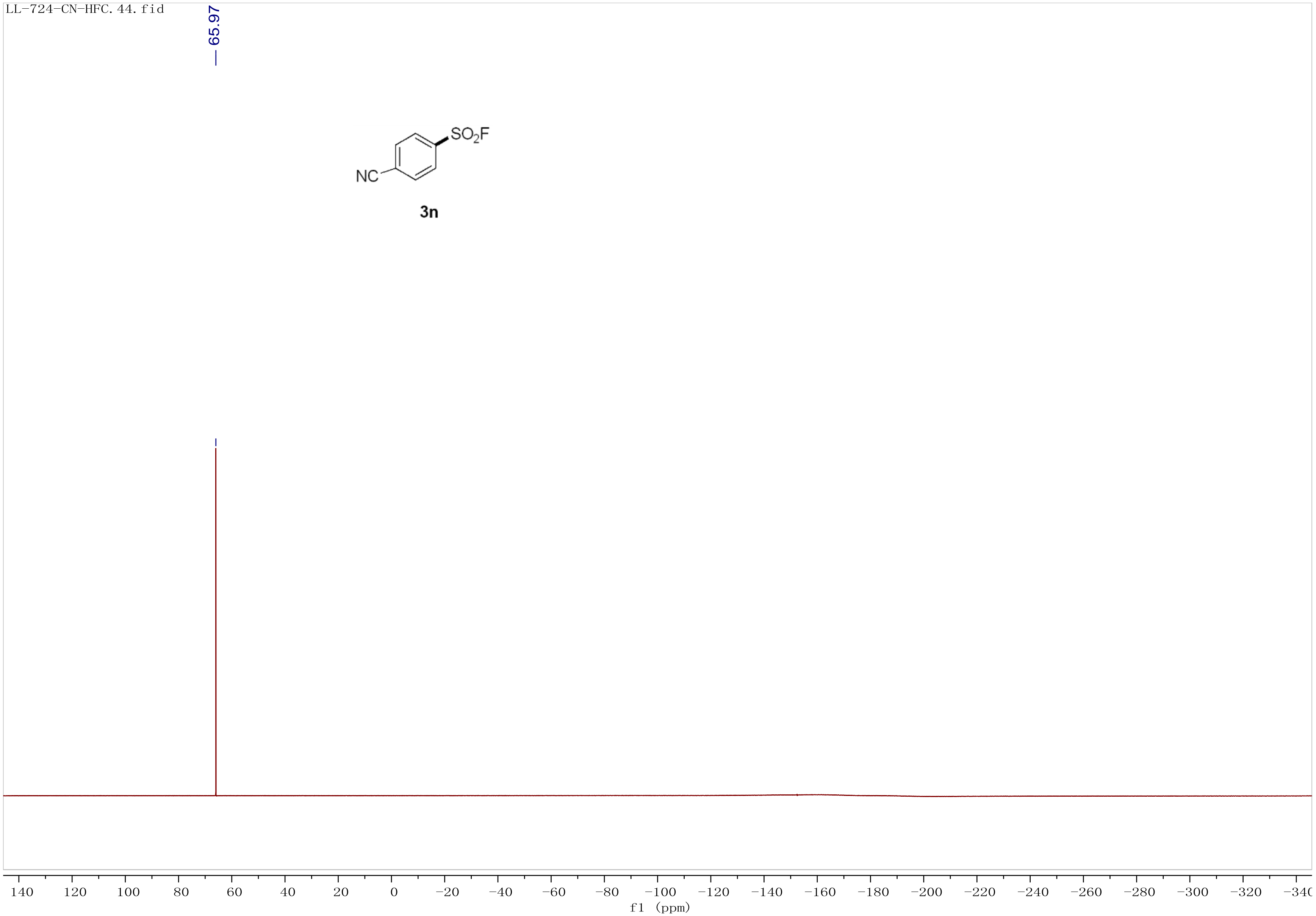

Supplementary Fig. 47 ^{19}F NMR spectrum of compound 3n ($CDCl_3$, 376 MHz, 298K)

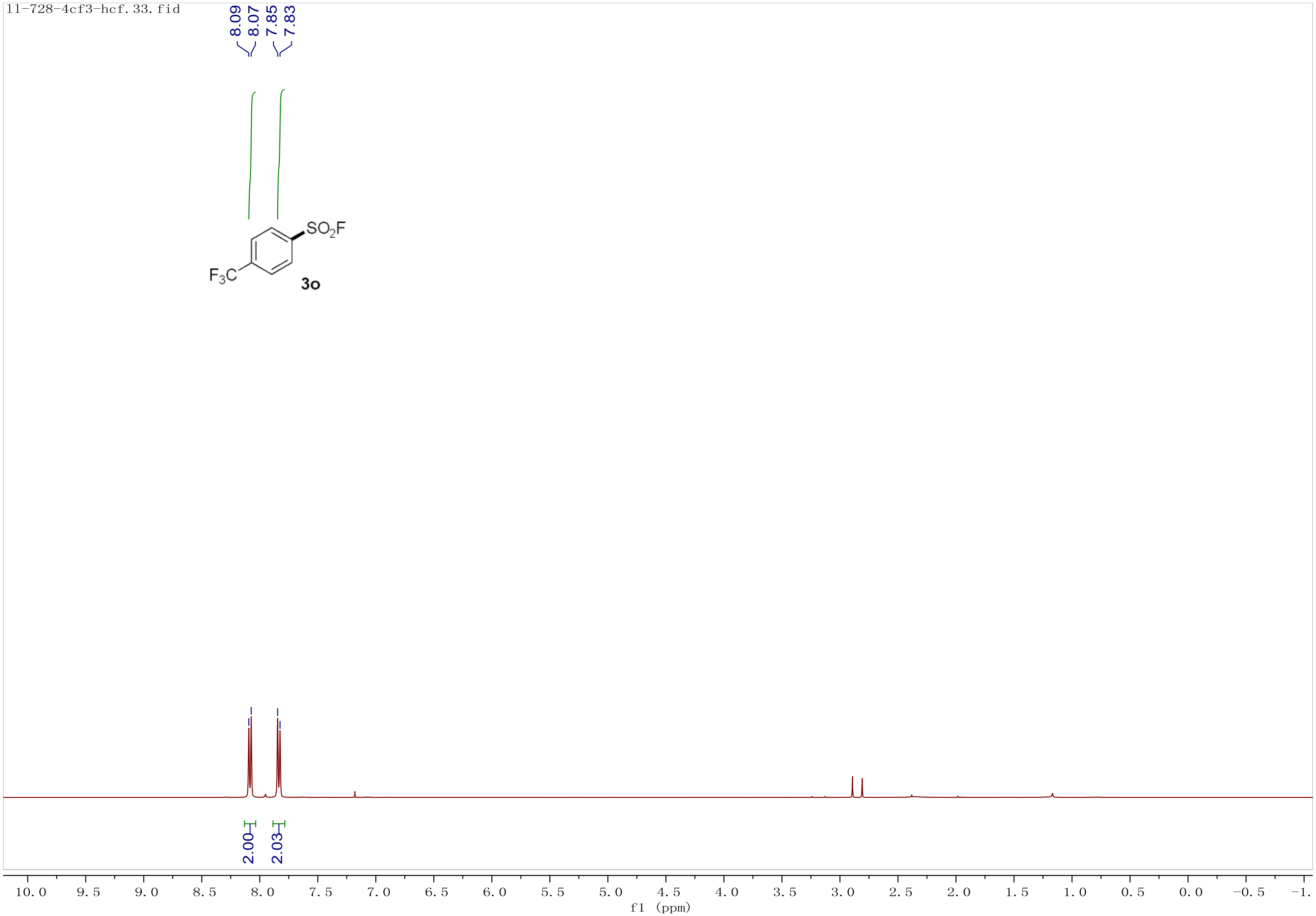

Supplementary Fig. 48 ^{1}H NMR spectrum of compound 3o ($CDCl_3$, 400 MHz, 298K)

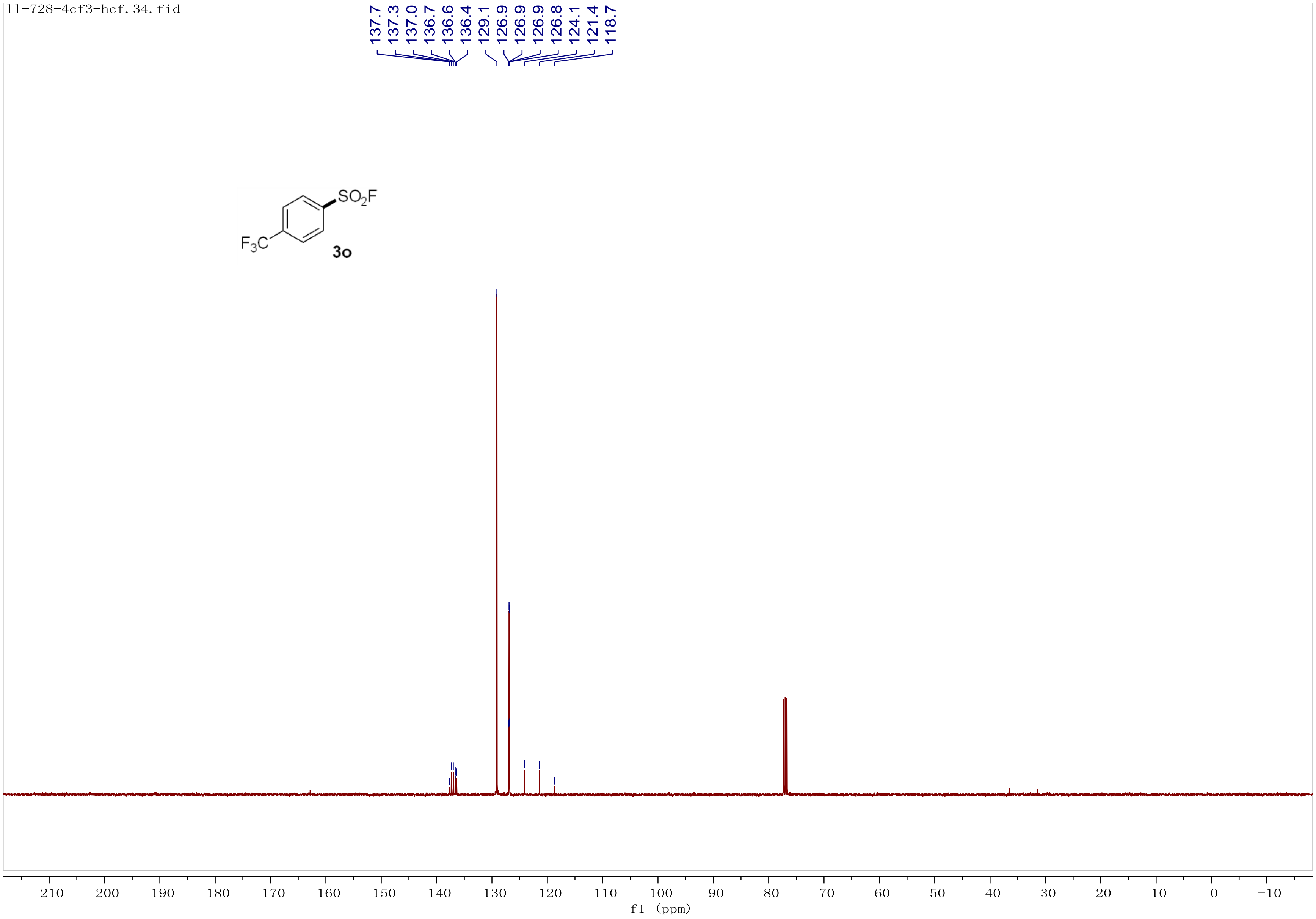

Supplementary Fig. 49 ^{13}C NMR spectrum of compound 3o ($CDCl_3$, 101 MHz, 298K)

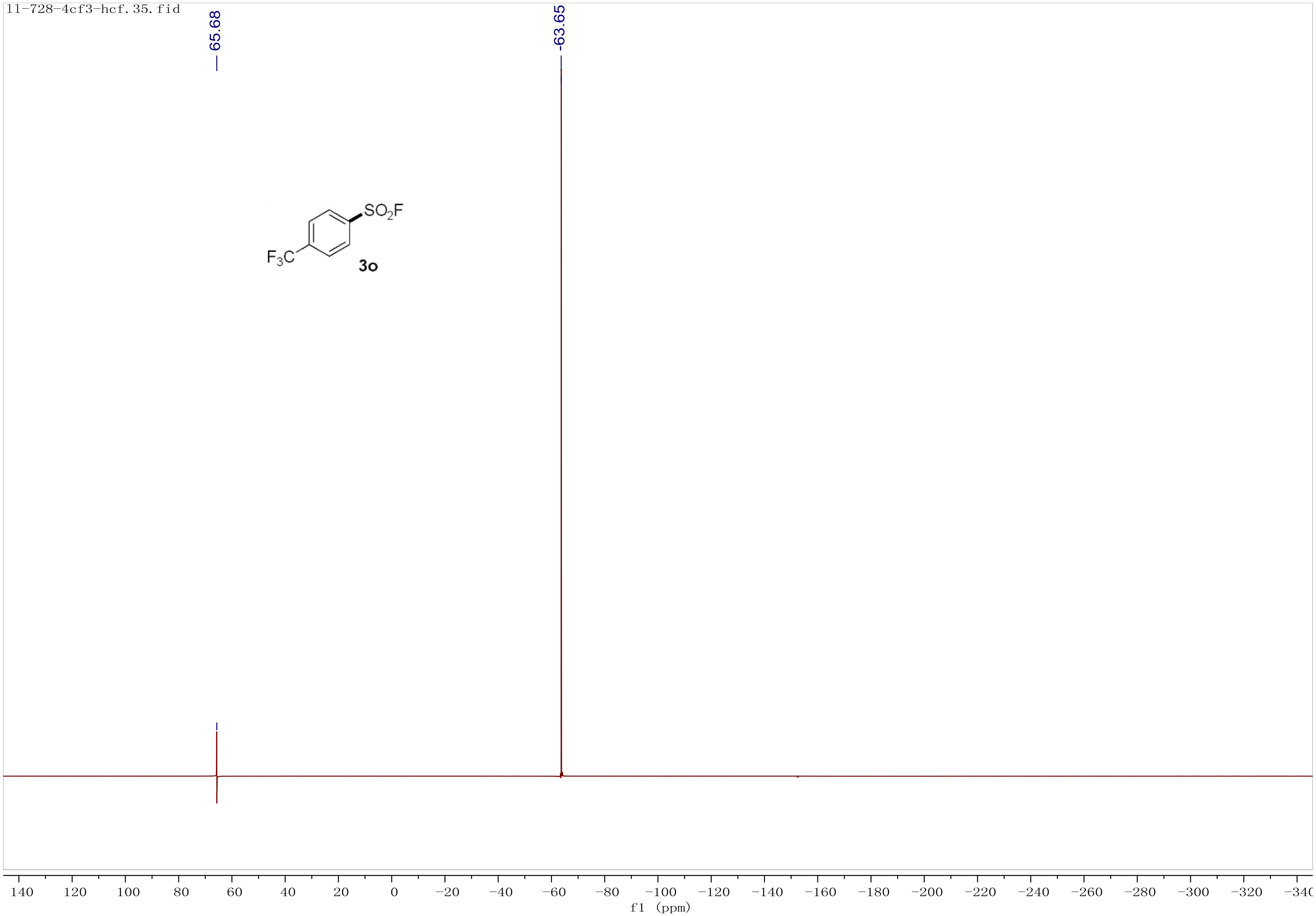

Supplementary Fig. 50 ^{19}F NMR spectrum of compound 3o ($CDCl_3$, 376 MHz, 298K)

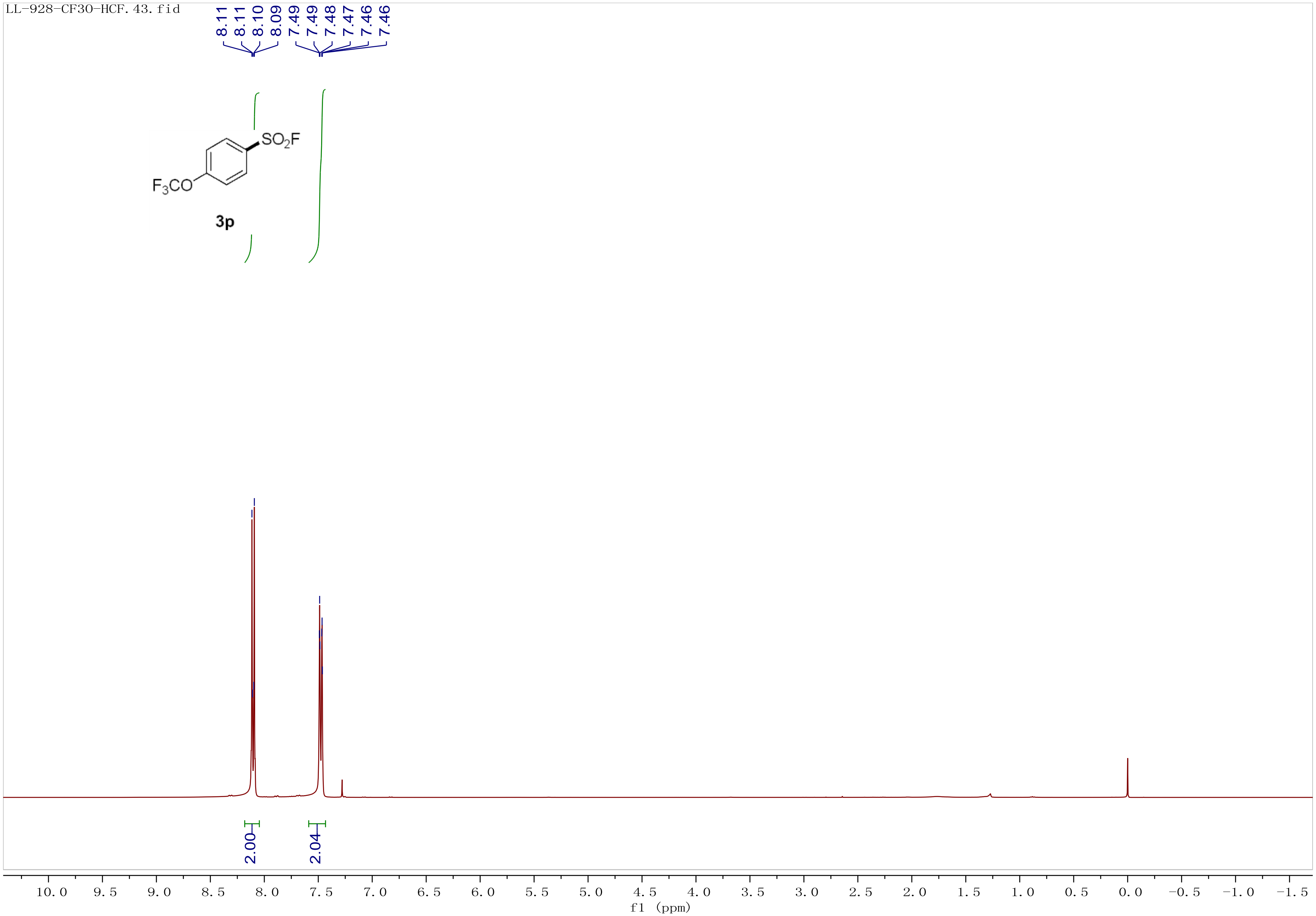

Supplementary Fig. 51 ^{1}H NMR spectrum of compound 3p ($CDCl_3$, 400 MHz, 298K)

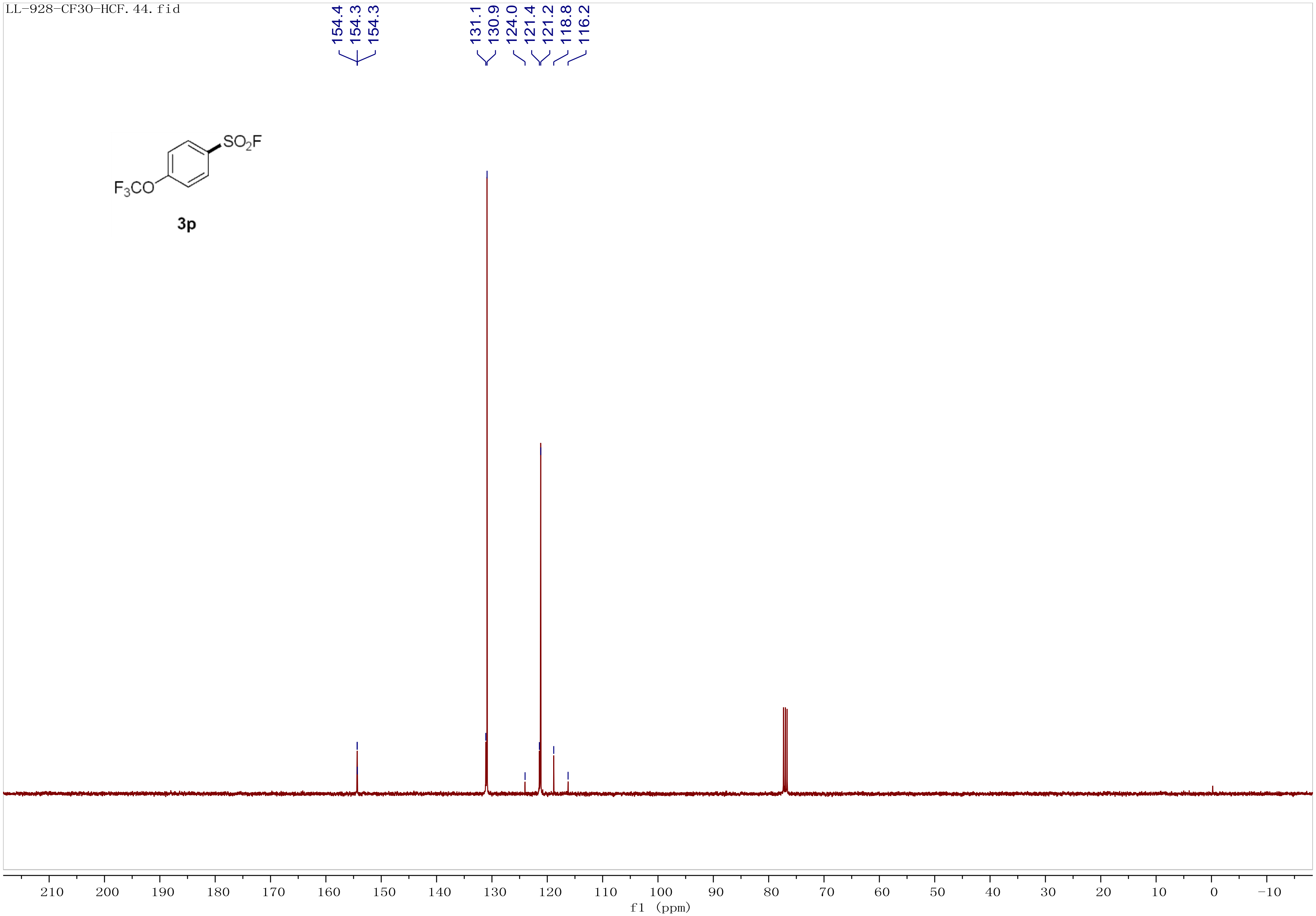

Supplementary Fig. 52 ^{13}C NMR spectrum of compound 3p ($CDCl_3$, 101 MHz, 298K)

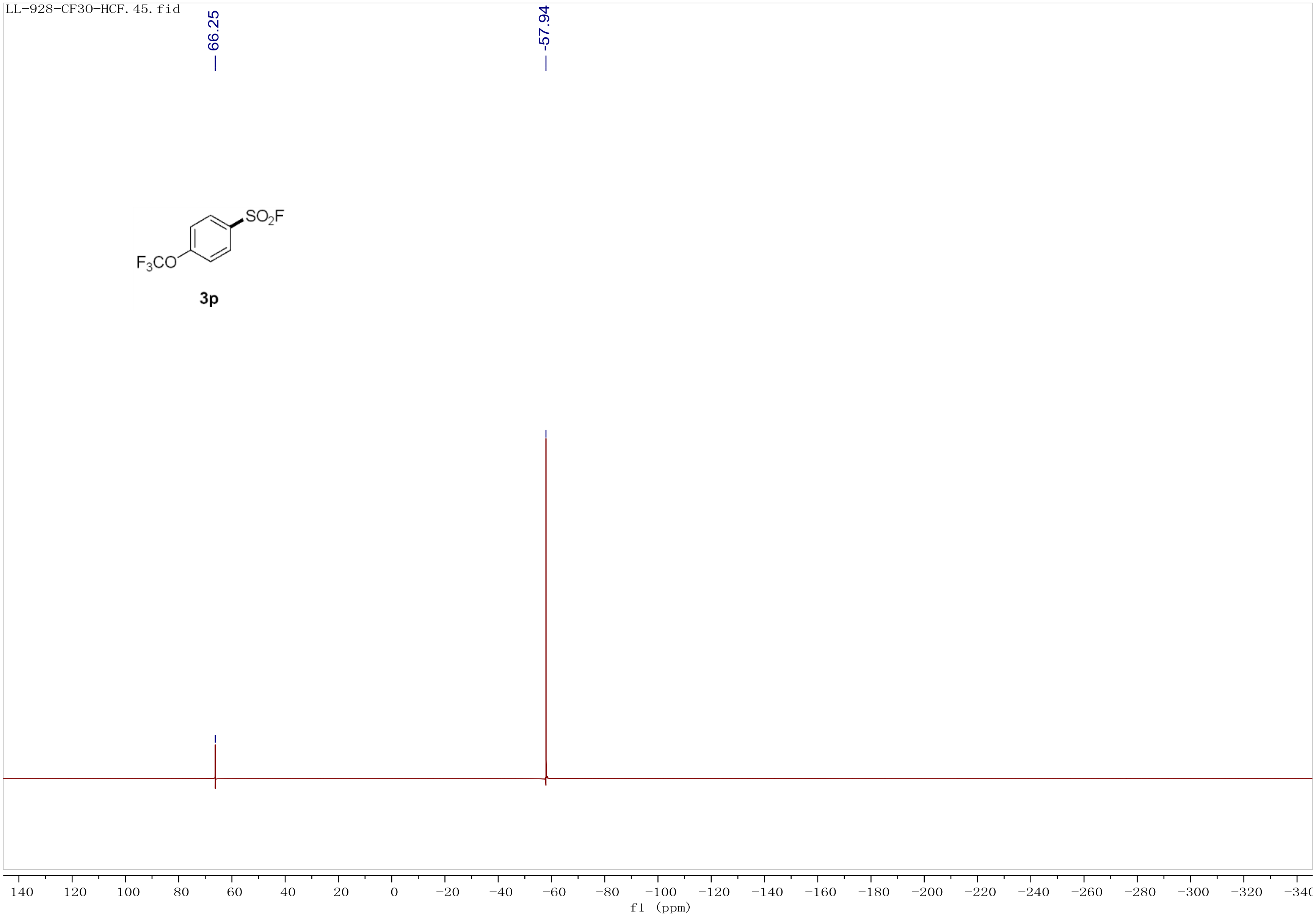

Supplementary Fig. 53 ^{19}F NMR spectrum of compound 3p ($CDCl_3$, 376 MHz, 298K)

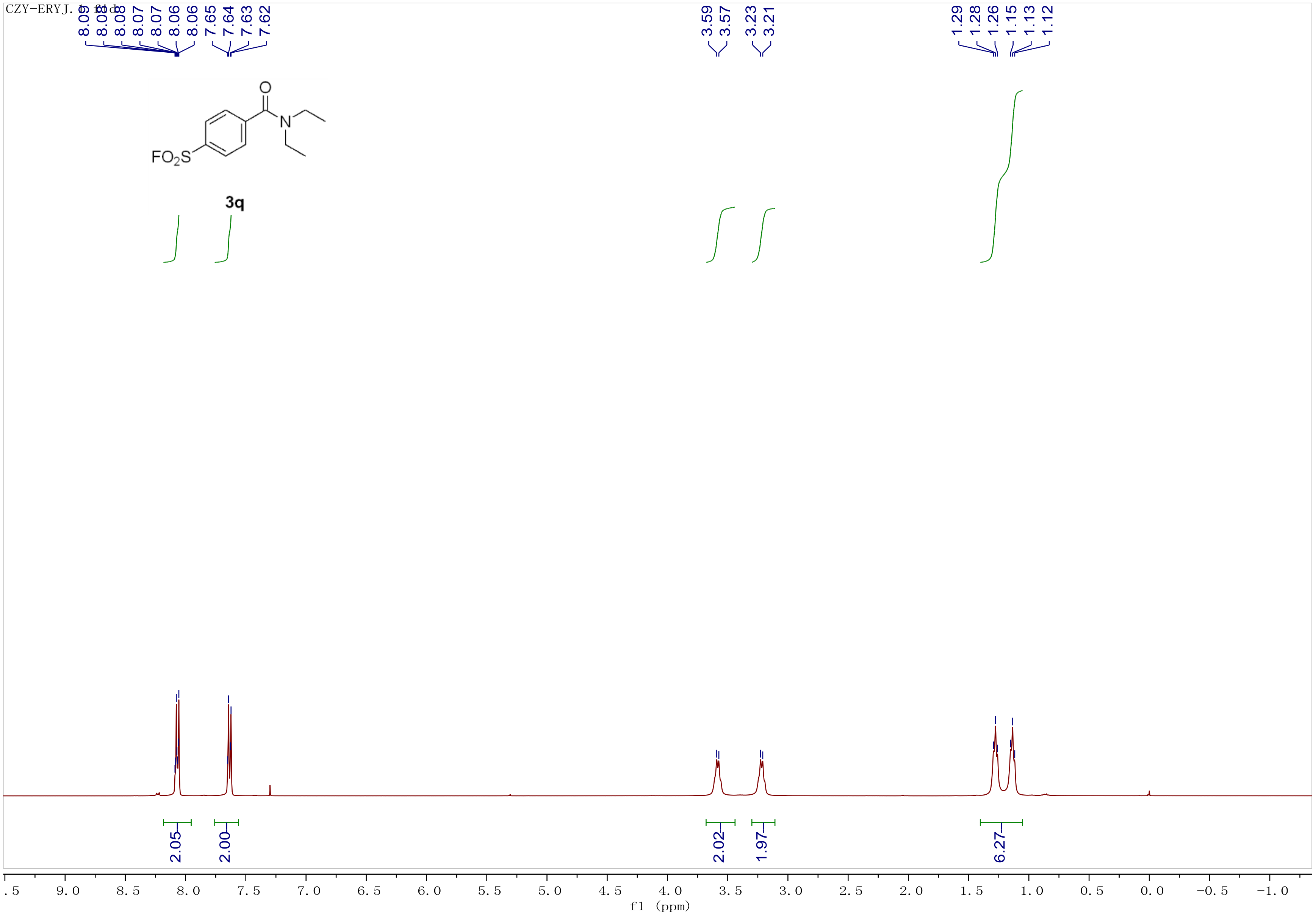

Supplementary Fig. 54 ^{1}H NMR spectrum of compound 3q ($CDCl_3$, 400 MHz, 298K)

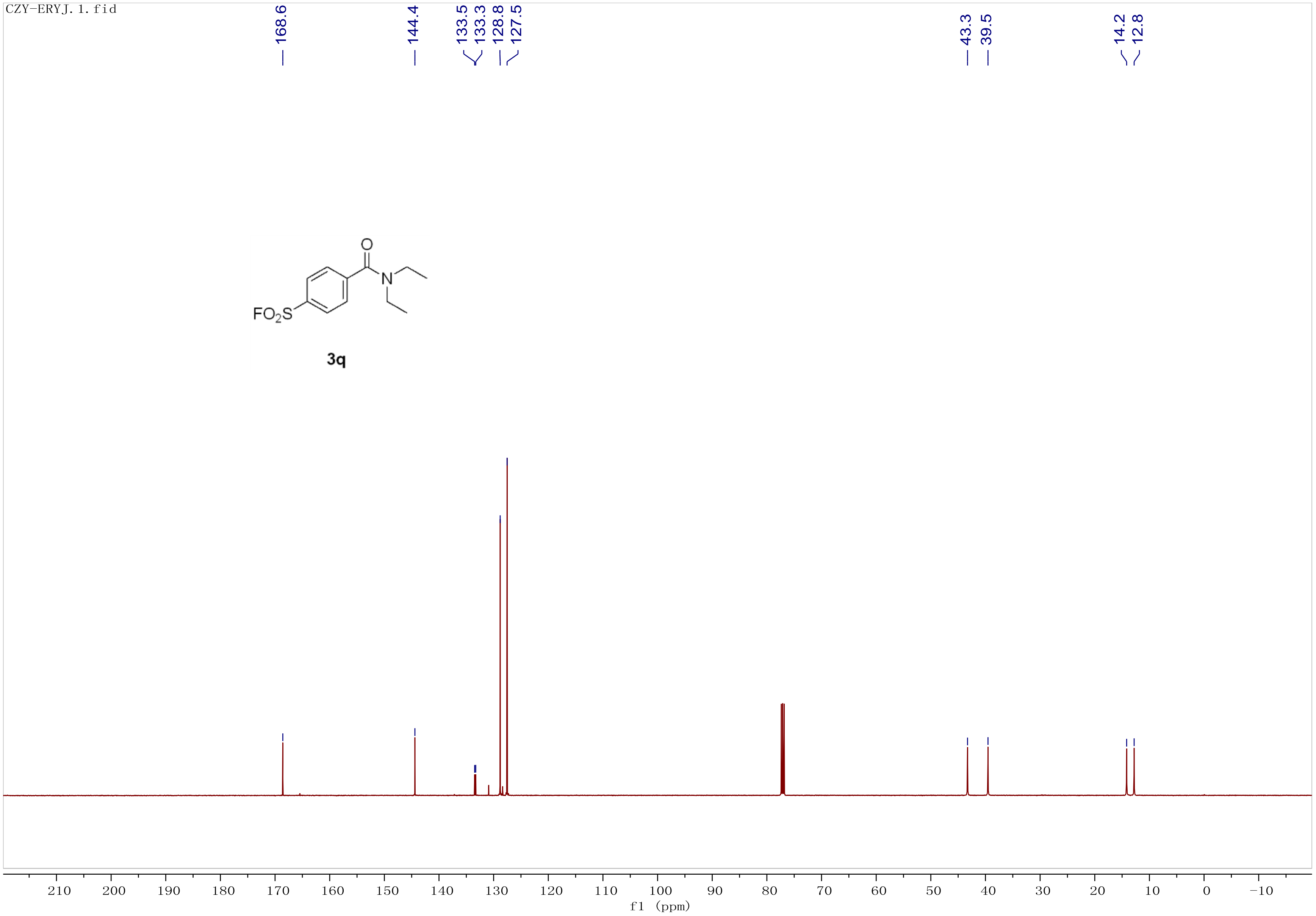

Supplementary Fig. 55 ^{13}C NMR spectrum of compound 3q ($CDCl_3$, 126 MHz, 298K)

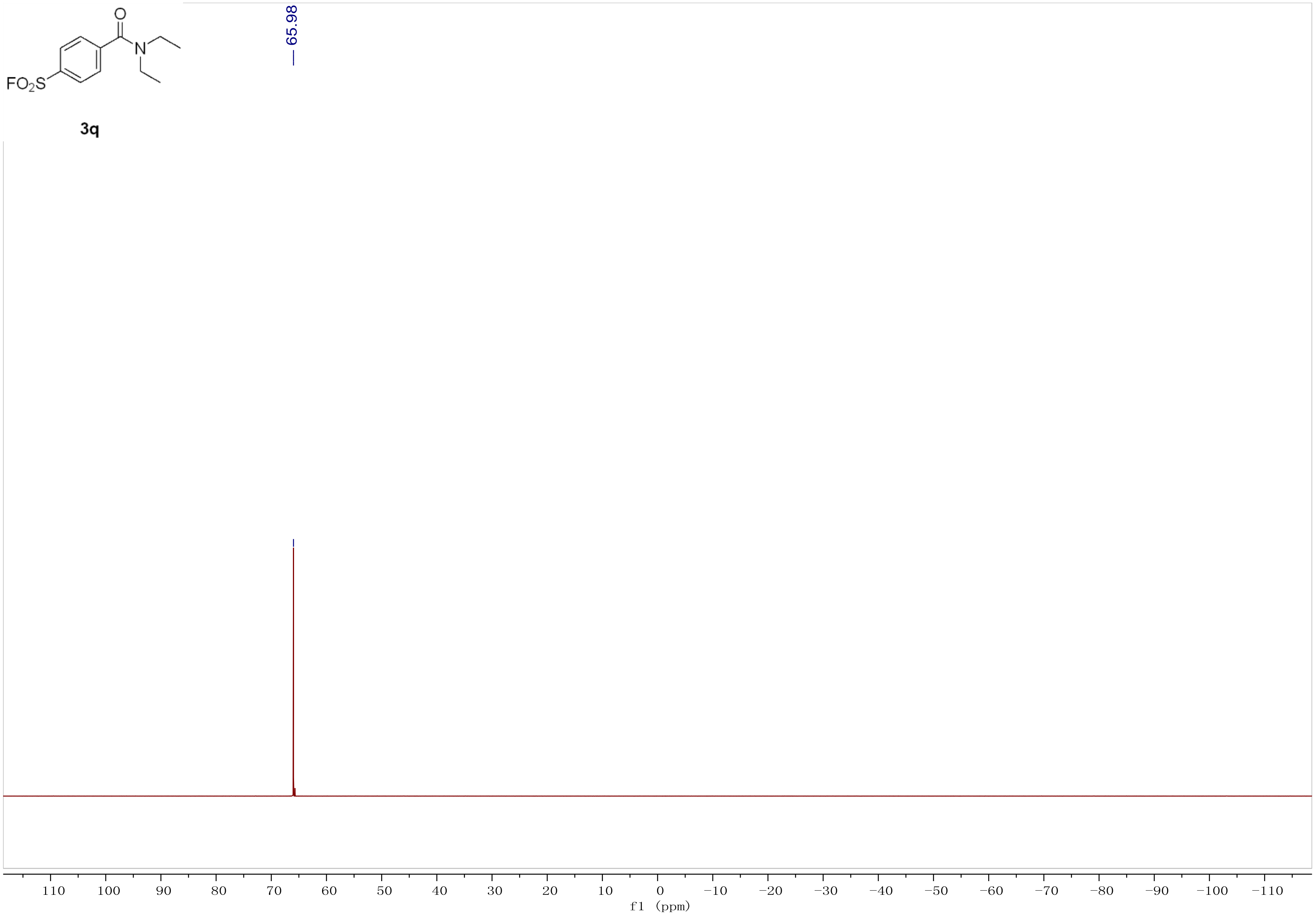

Supplementary Fig. 56 ^{19}F NMR spectrum of compound 3q ($CDCl_3$, 376 MHz, 298K)

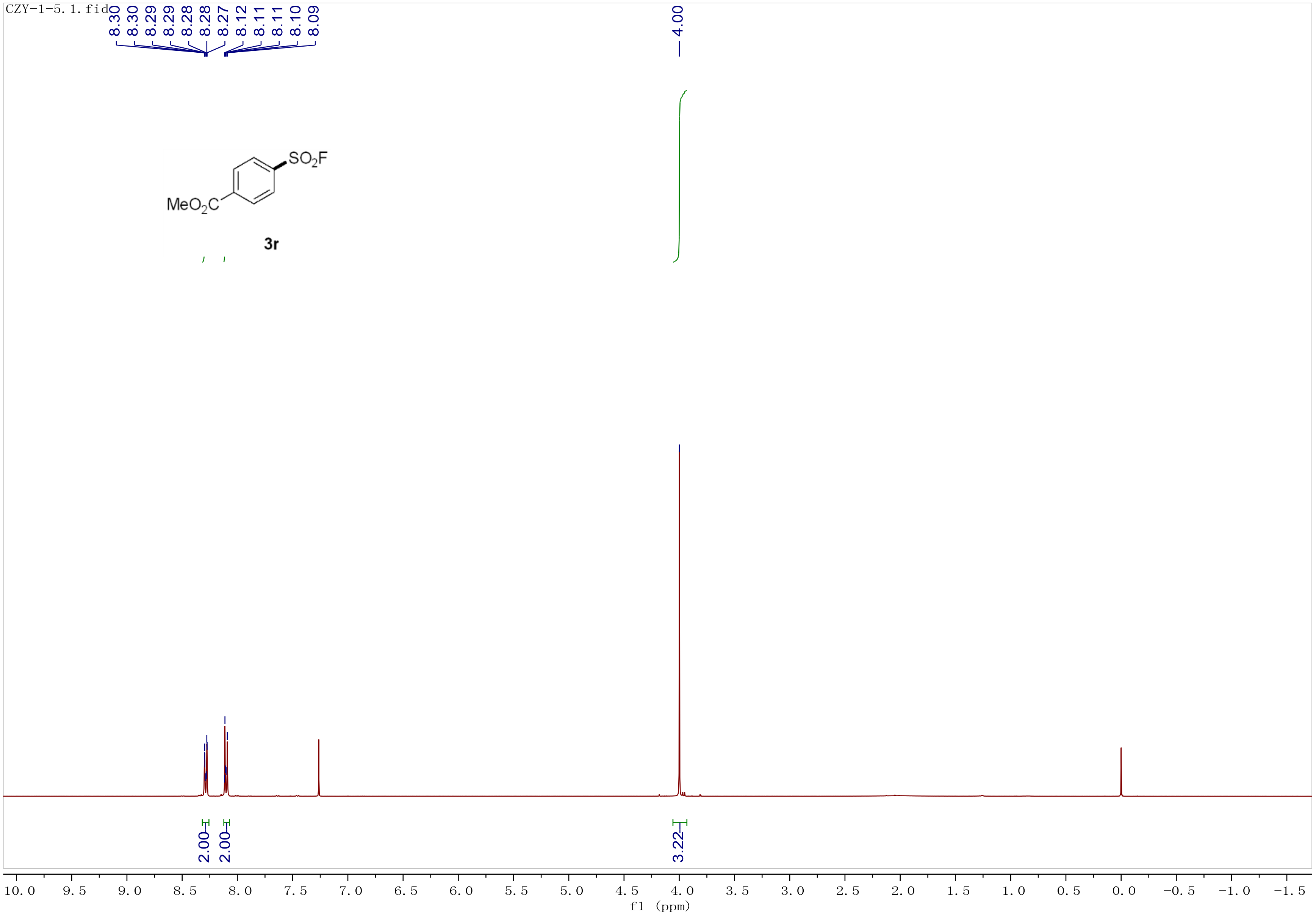

Supplementary Fig. 57 ^{1}H NMR spectrum of compound 3r ($CDCl_3$, 400 MHz, 298K)

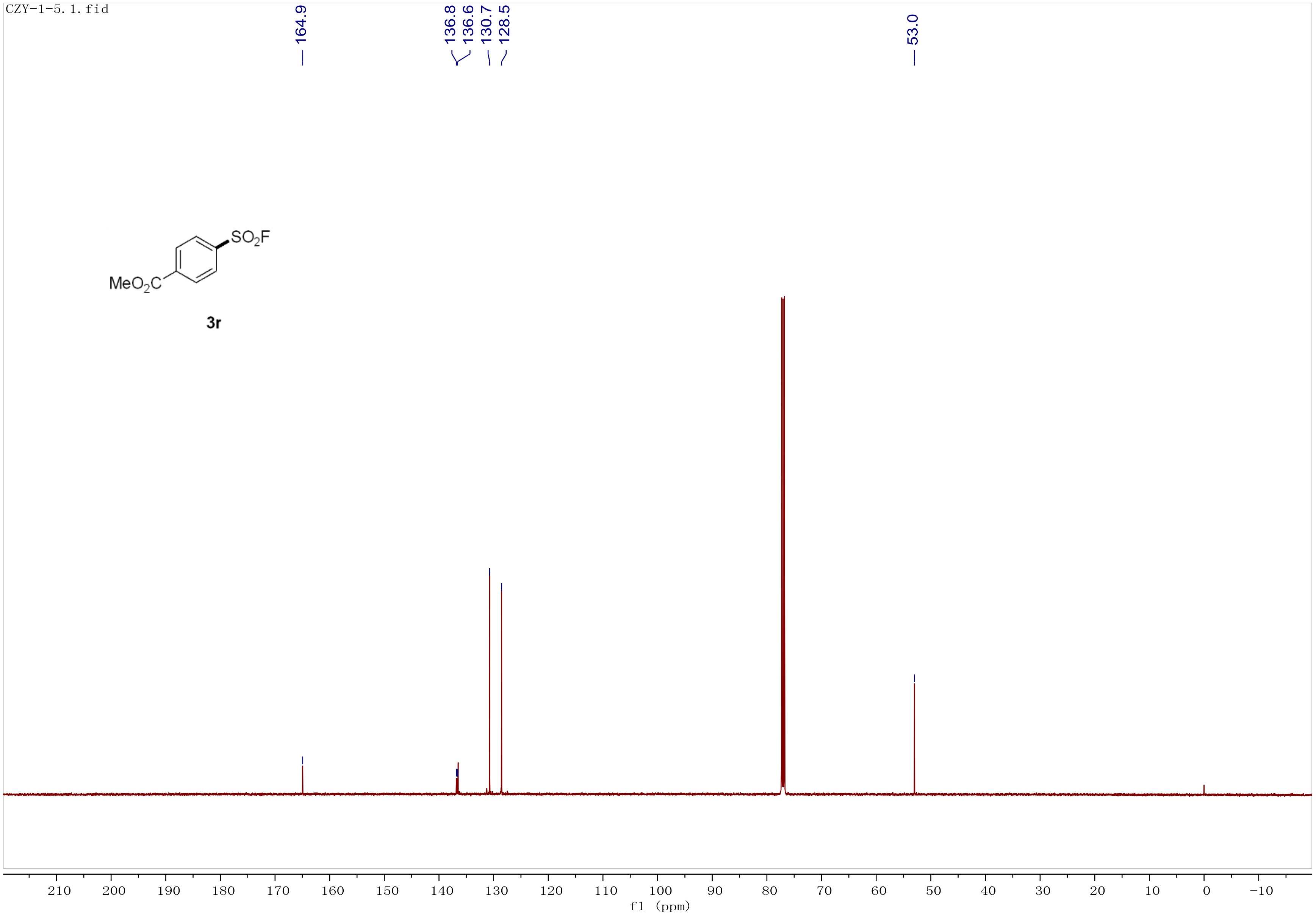

Supplementary Fig. 58 ^{13}C NMR spectrum of compound 3r ($CDCl_3$, 101 MHz, 298K)

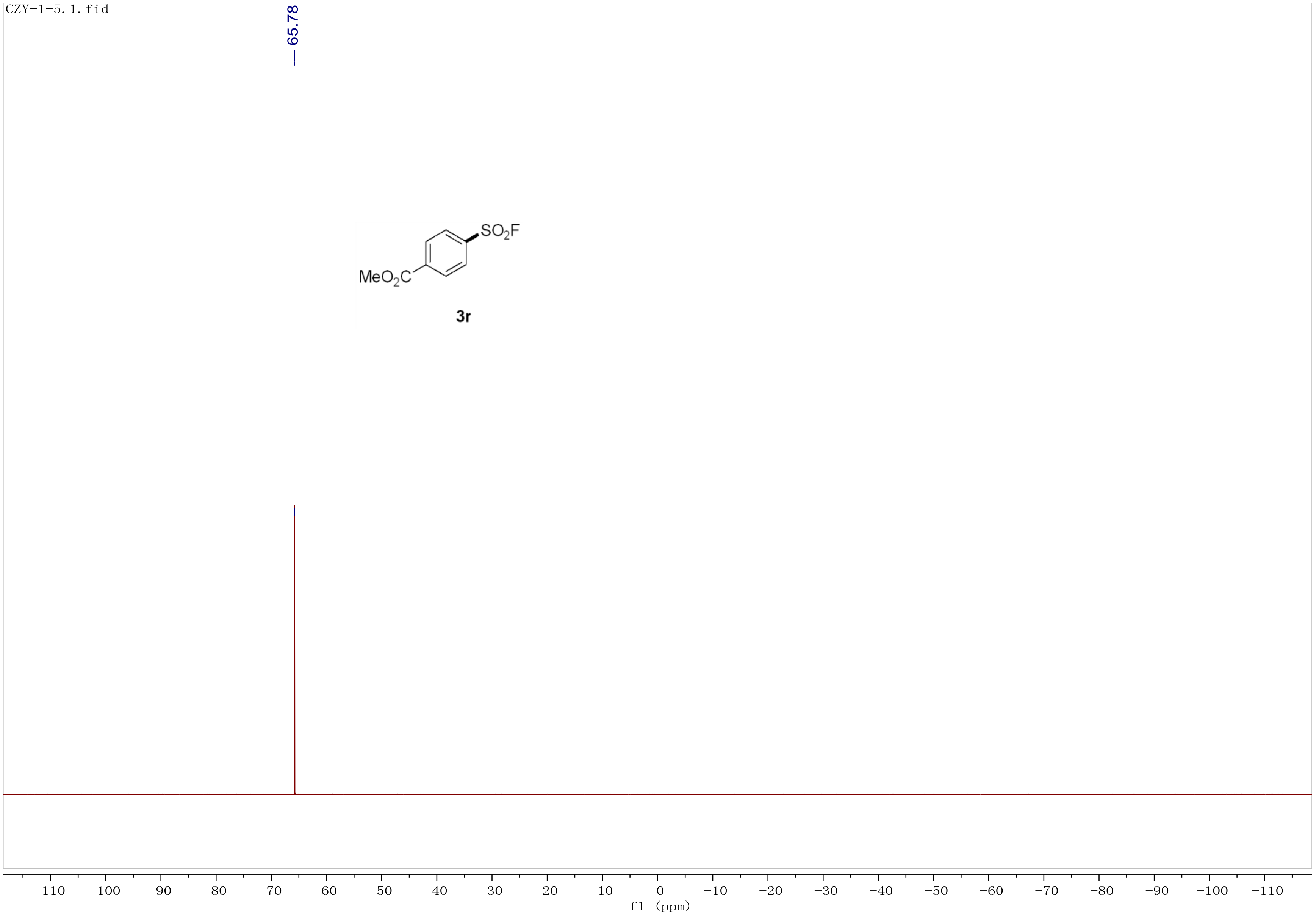

Supplementary Fig. 59 ^{19}F NMR spectrum of compound 3r ($CDCl_3$, 376 MHz, 298K)

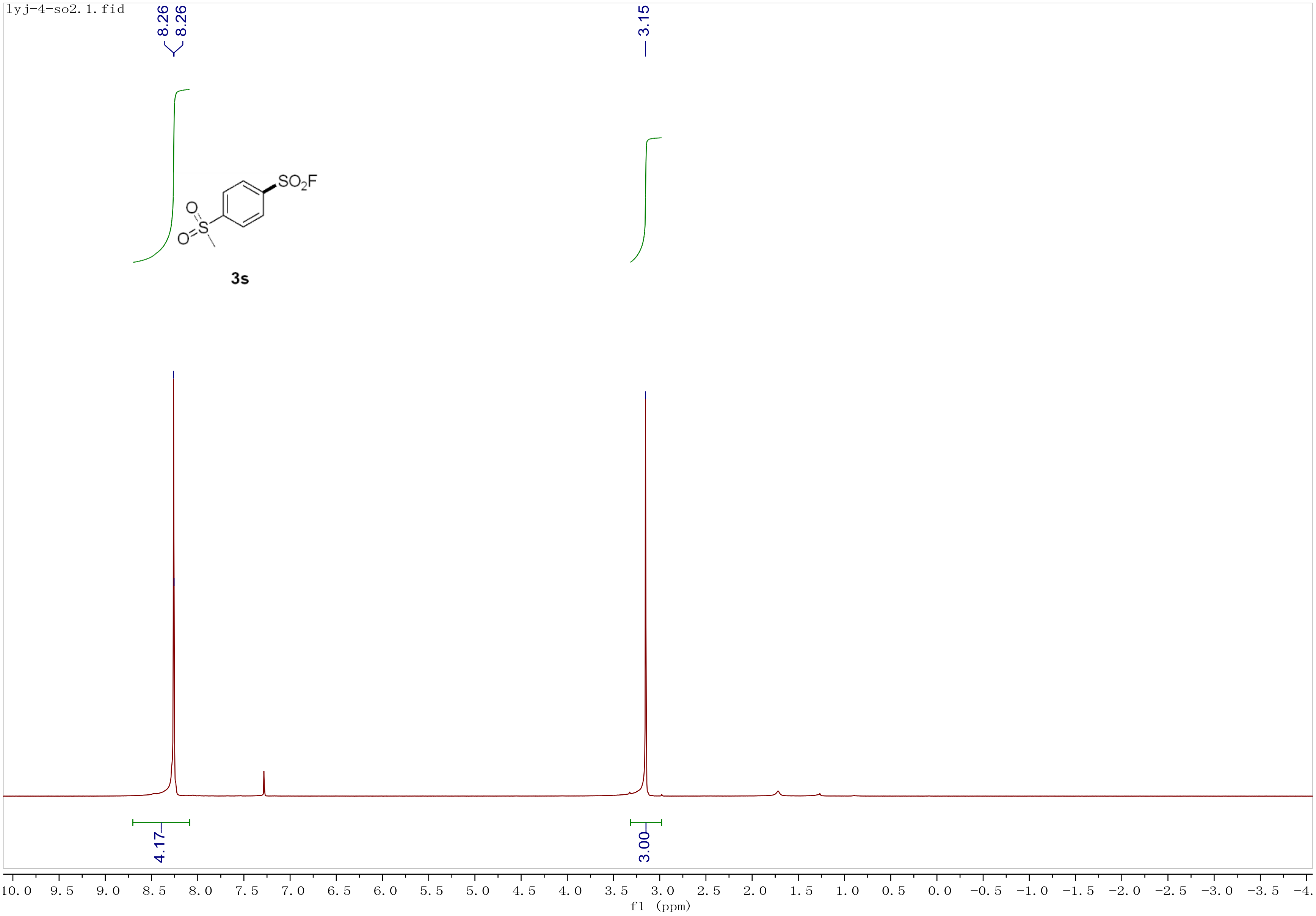

Supplementary Fig. 60 ^{1}H NMR spectrum of compound 3s ($CDCl_3$, 400 MHz, 298K)

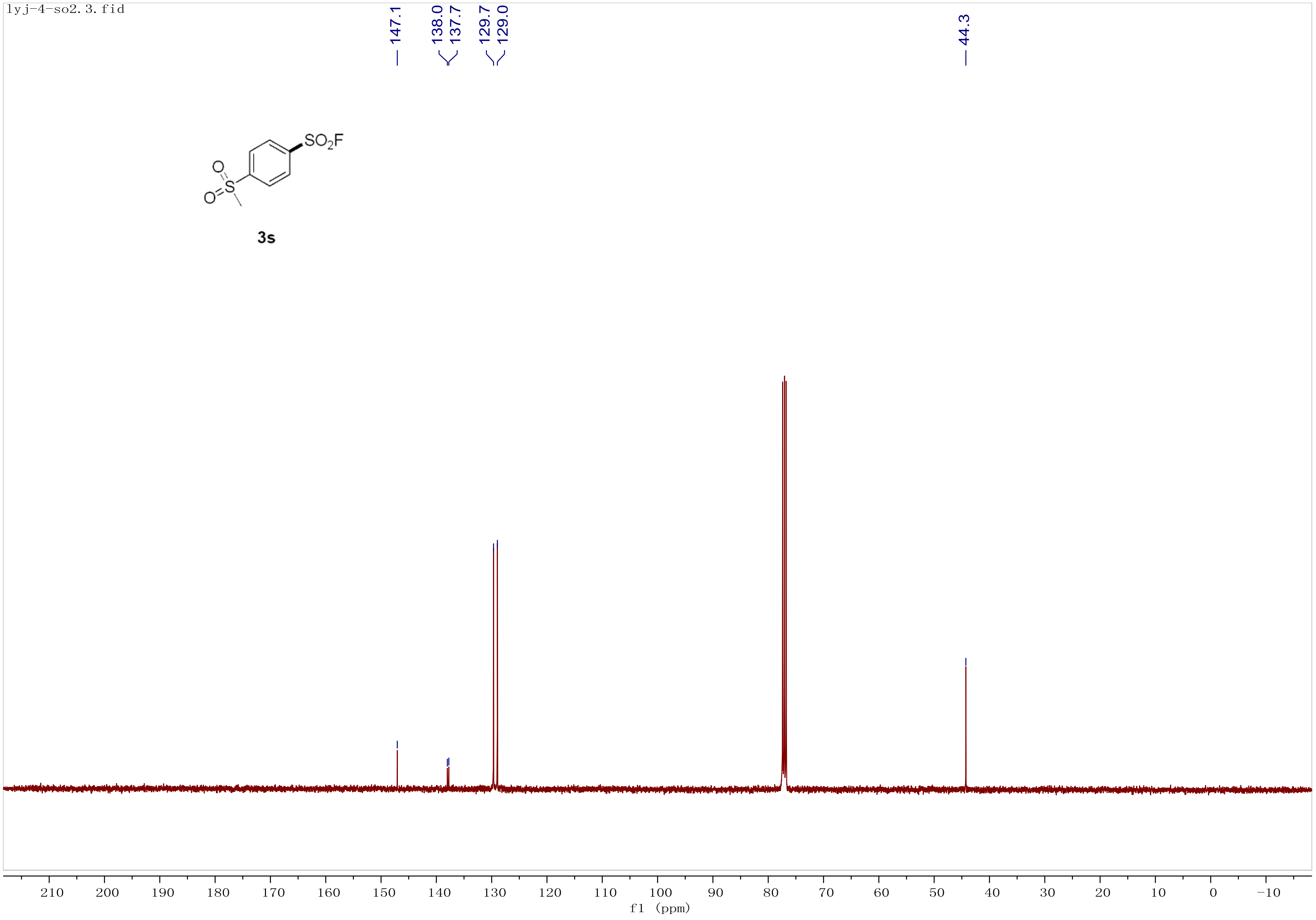

Supplementary Fig. 61 ^{13}C NMR spectrum of compound 3s ($CDCl_3$, 101 MHz, 298K)

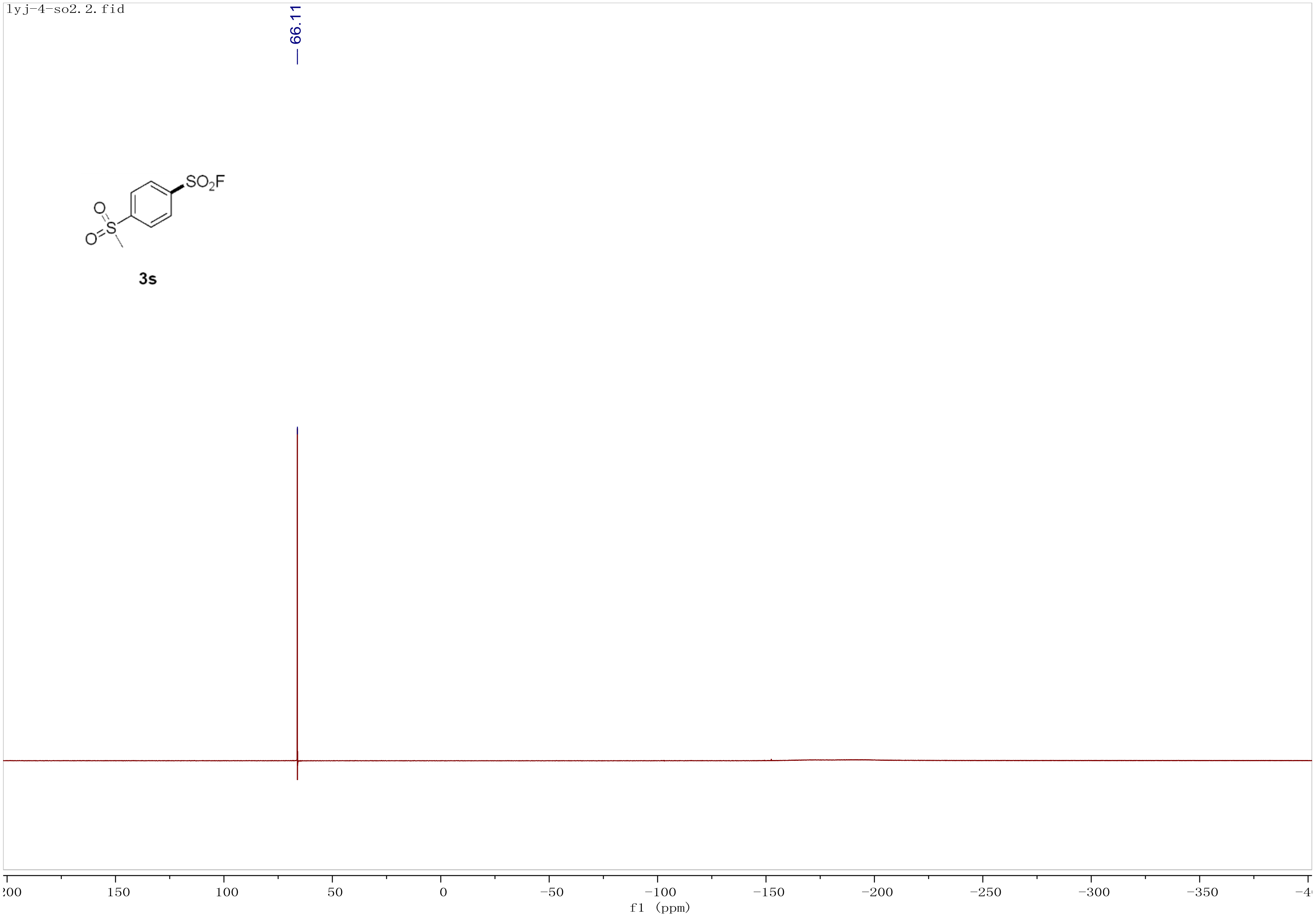

Supplementary Fig. 62 ^{19}F NMR spectrum of compound 3s ($CDCl_3$, 376 MHz, 298K)

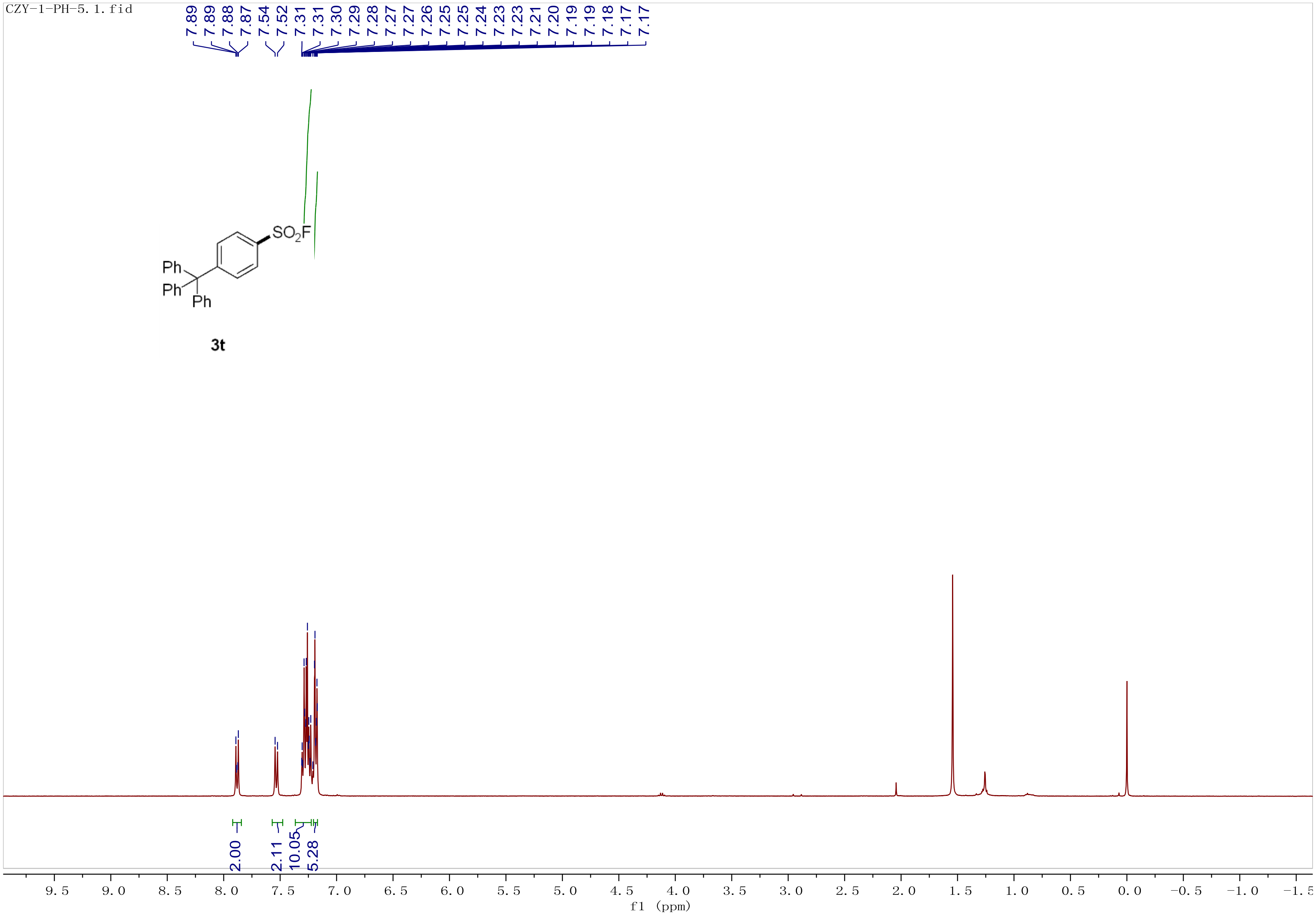

Supplementary Fig. 63 ^{1}H NMR spectrum of compound 3t ($CDCl_3$, 400 MHz, 298K)

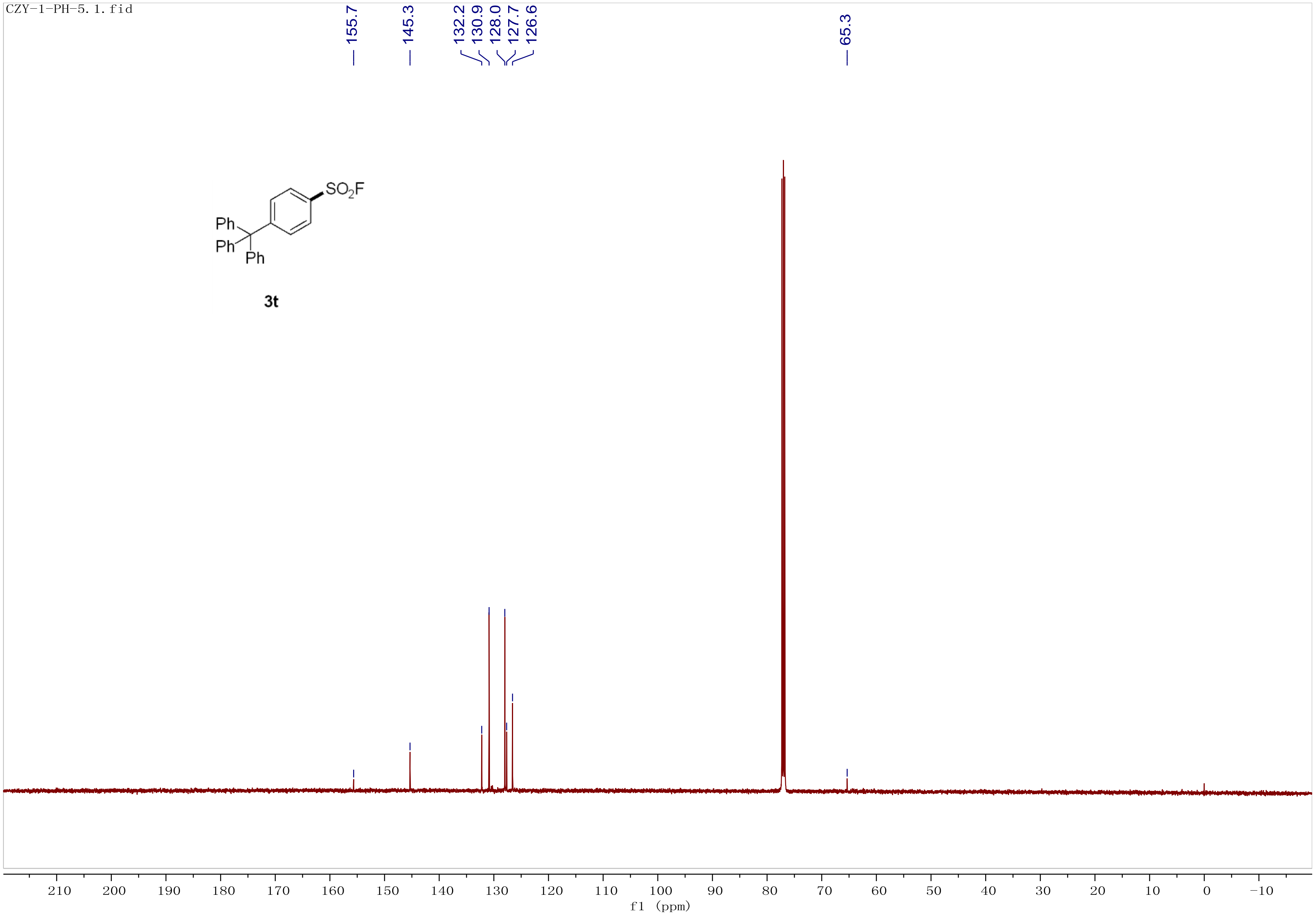

Supplementary Fig. 64 ^{13}C NMR spectrum of compound 3t ($CDCl_3$, 126 MHz, 298K)

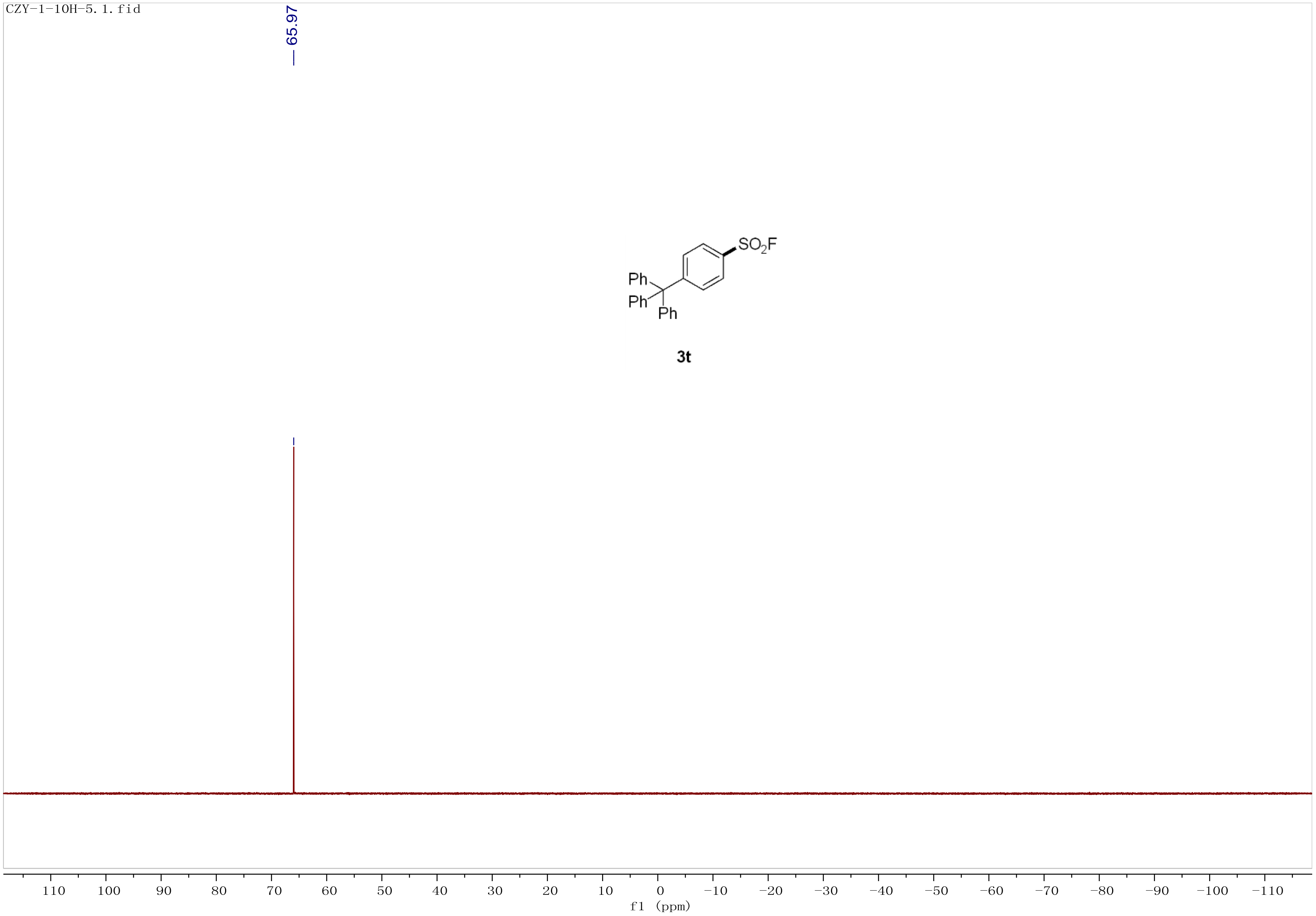

Supplementary Fig. 65 ^{19}F NMR spectrum of compound 3t ($CDCl_3$, 376 MHz, 298K)

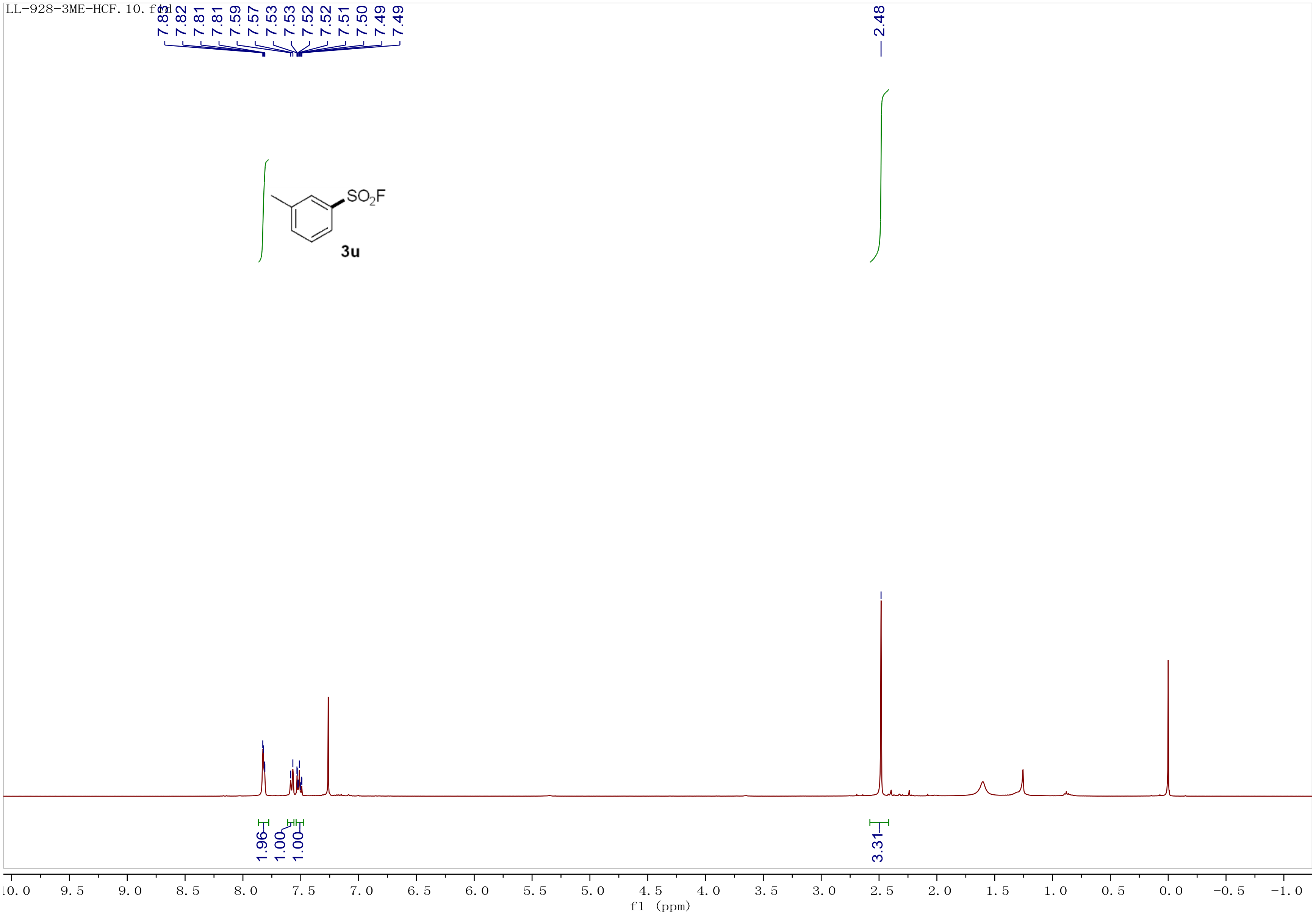

Supplementary Fig. 66 ^{1}H NMR spectrum of compound 3u ($CDCl_3$, 400 MHz, 298K)

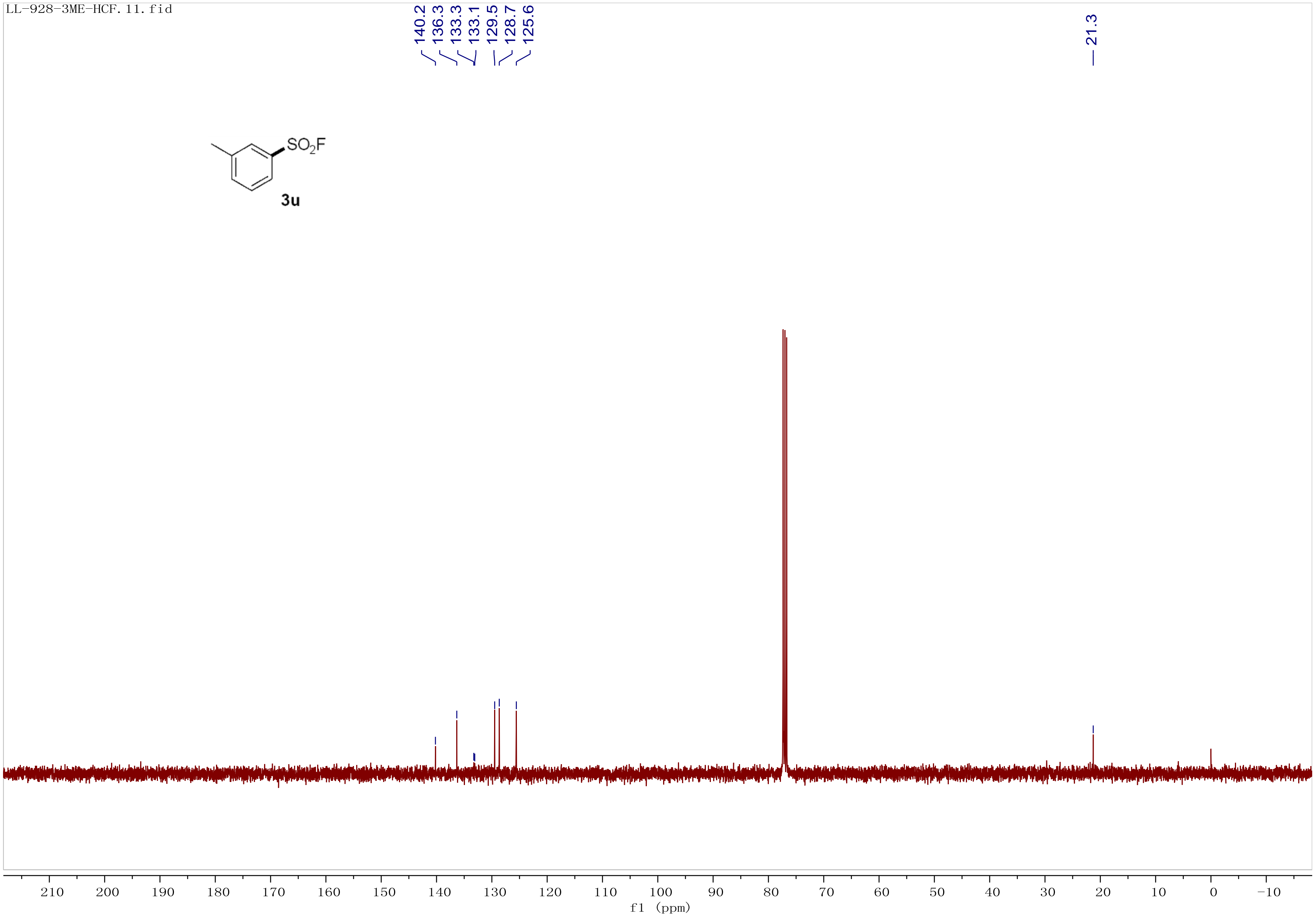

Supplementary Fig. 67 ^{13}C NMR spectrum of compound 3u ($CDCl_3$, 101 MHz, 298K)

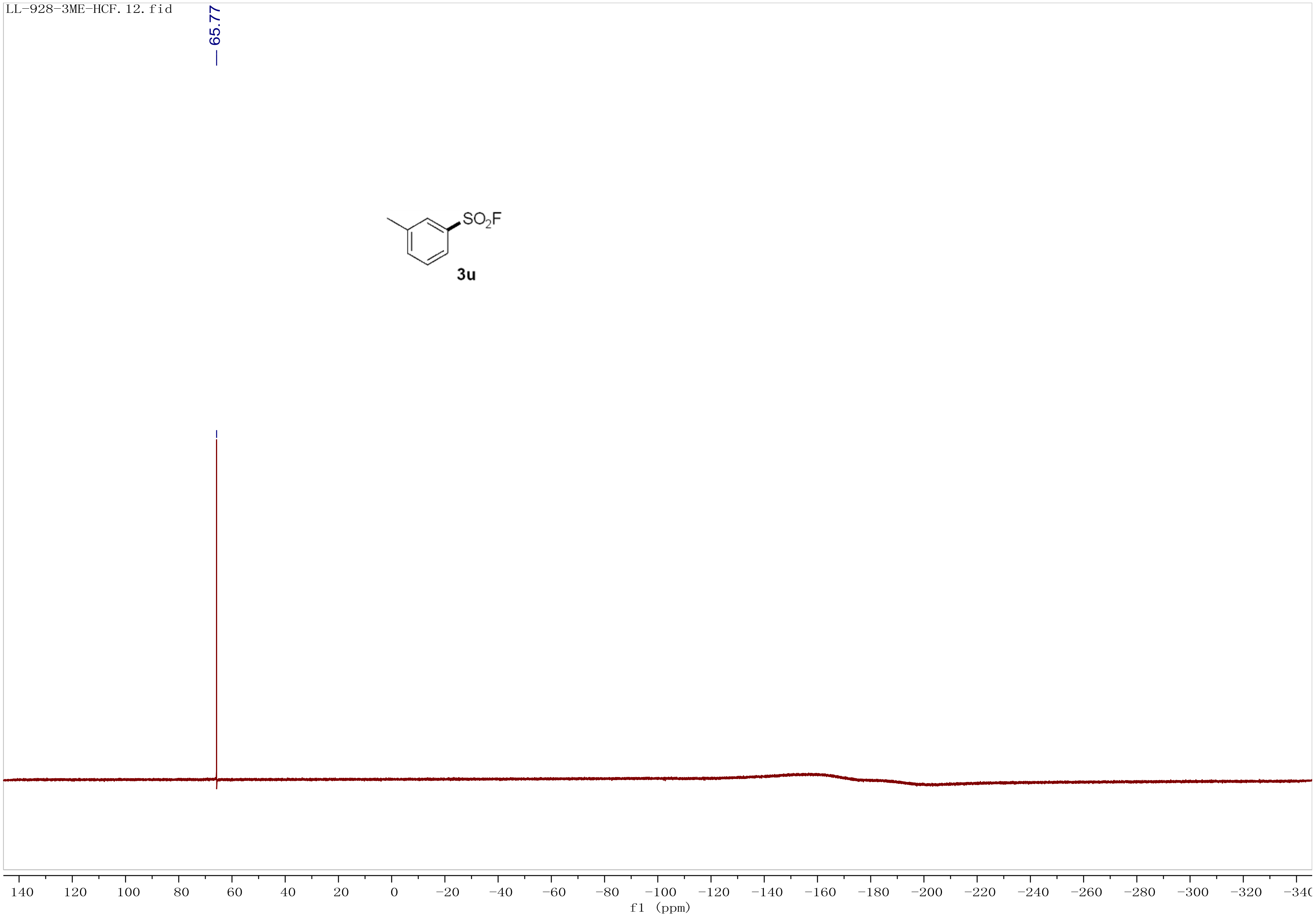

Supplementary Fig. 68 ^{19}F NMR spectrum of compound 3u ($CDCl_3$, 376 MHz, 298K)

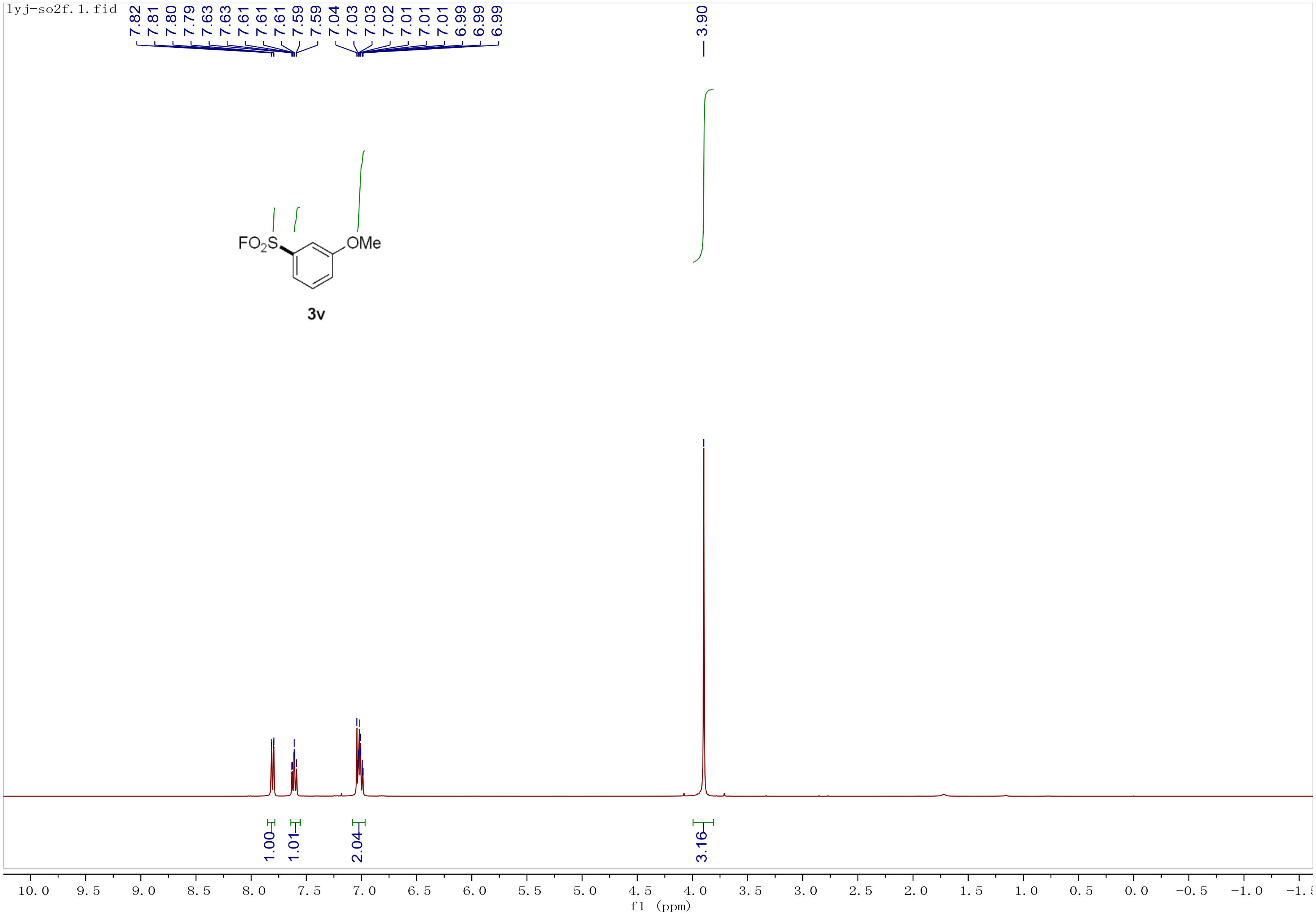

Supplementary Fig. 69 ^{1}H NMR spectrum of compound 3v ($CDCl_3$, 400 MHz, 298K)

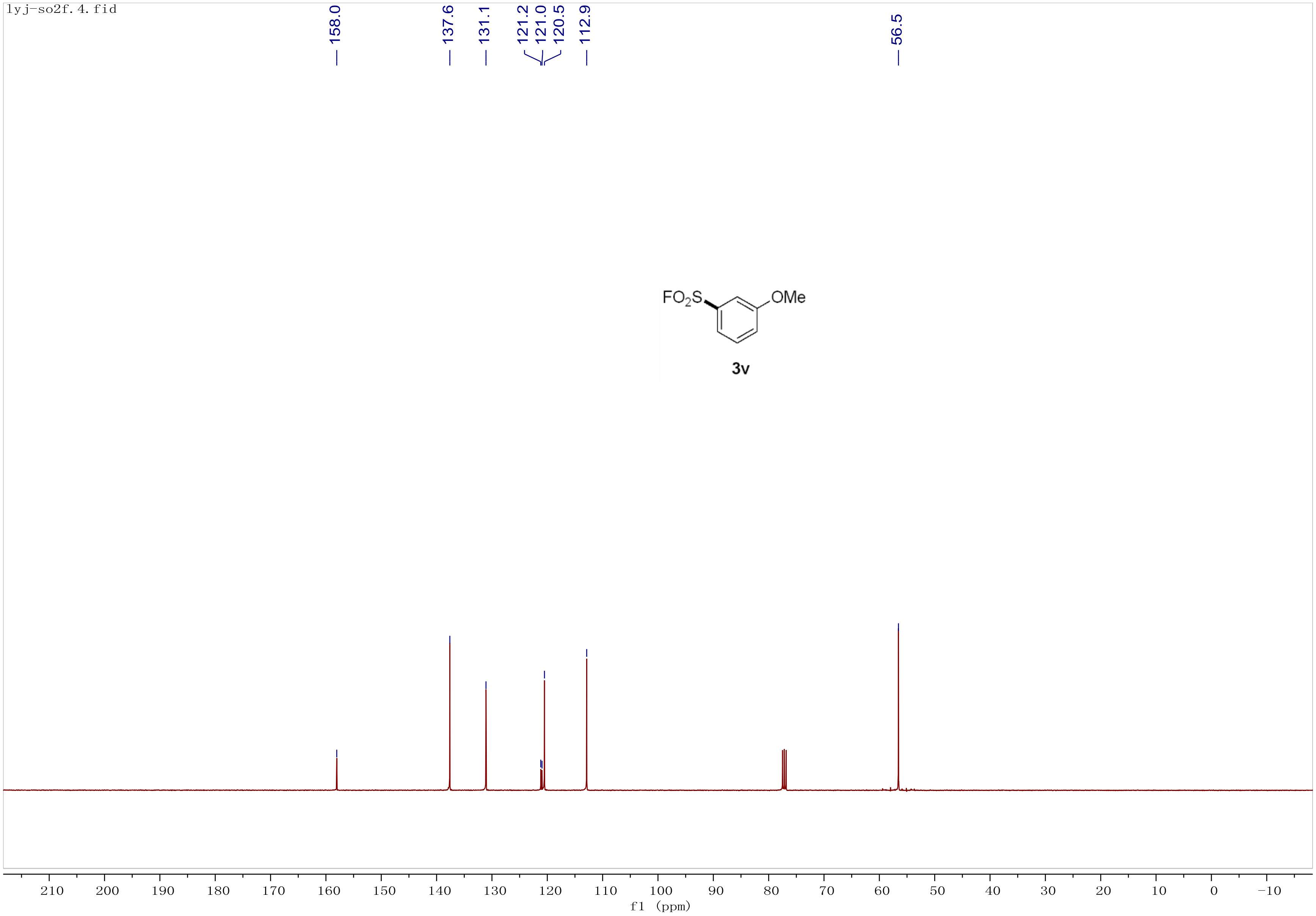

Supplementary Fig. 70 ^{13}C NMR spectrum of compound 3v ($CDCl_3$, 101 MHz, 298K)

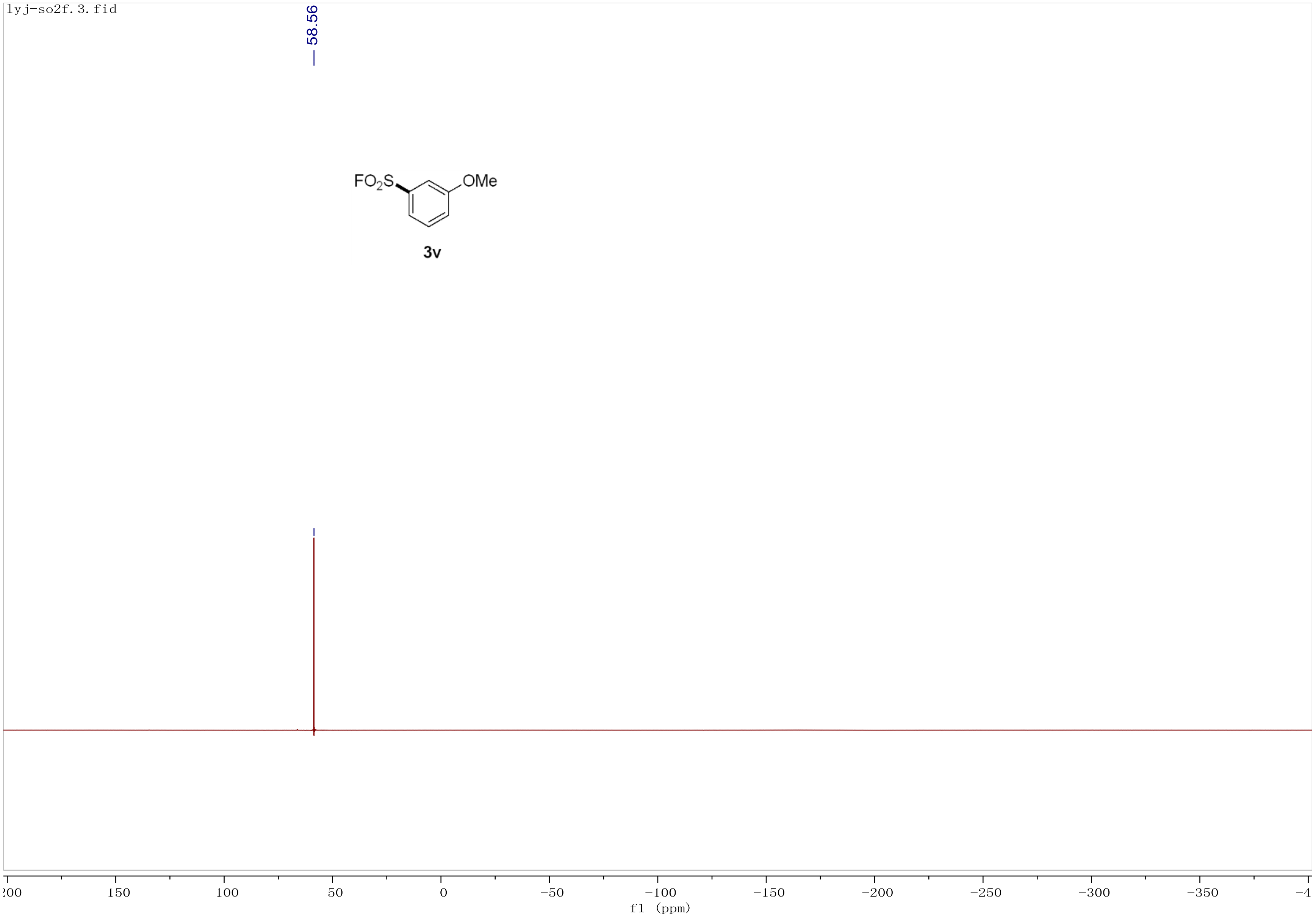

Supplementary Fig. 71 ^{19}F NMR spectrum of compound 3v ($CDCl_3$, 376 MHz, 298K)

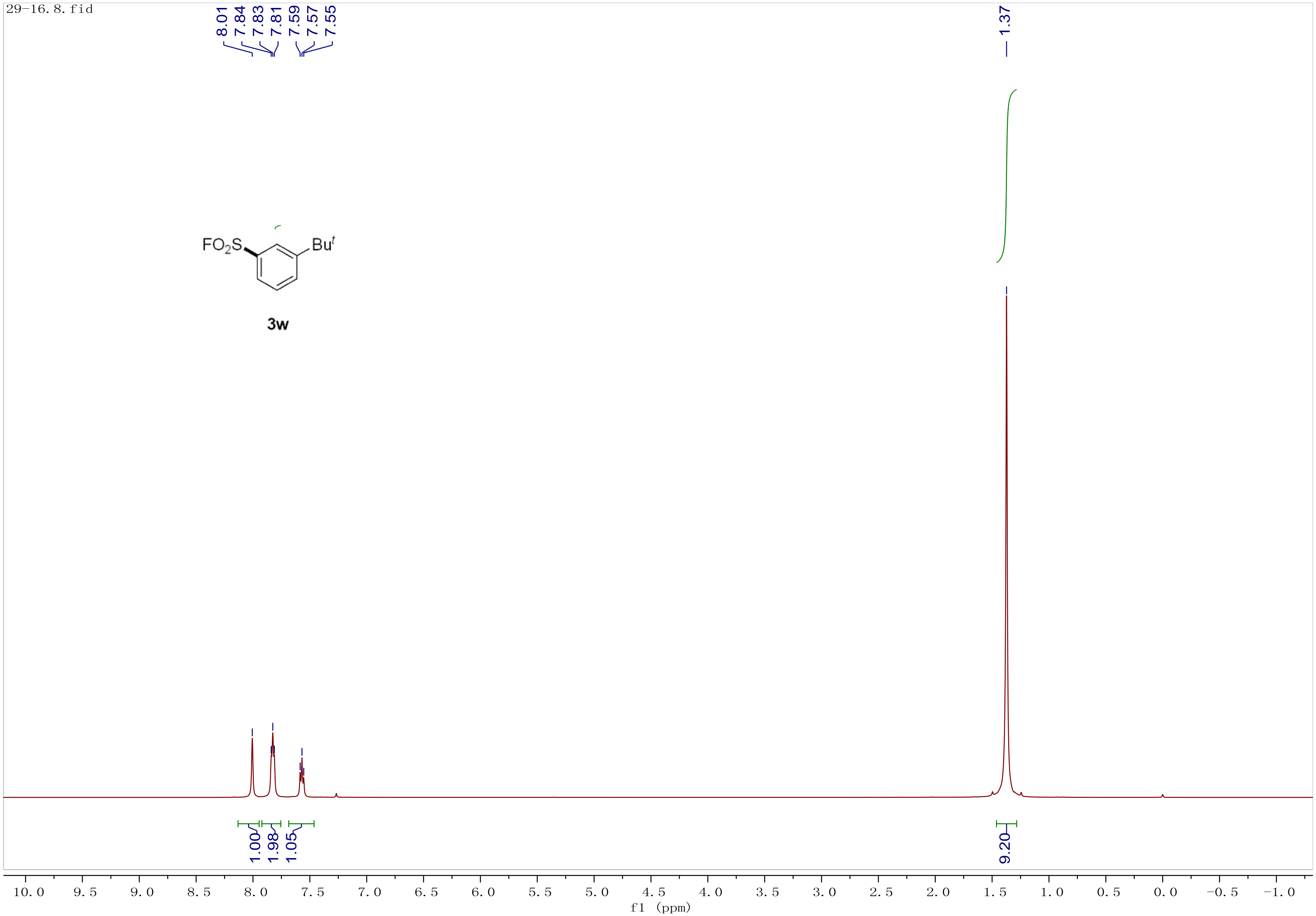

Supplementary Fig. 72 ^{1}H NMR spectrum of compound 3w ($CDCl_3$, 500 MHz, 298K)

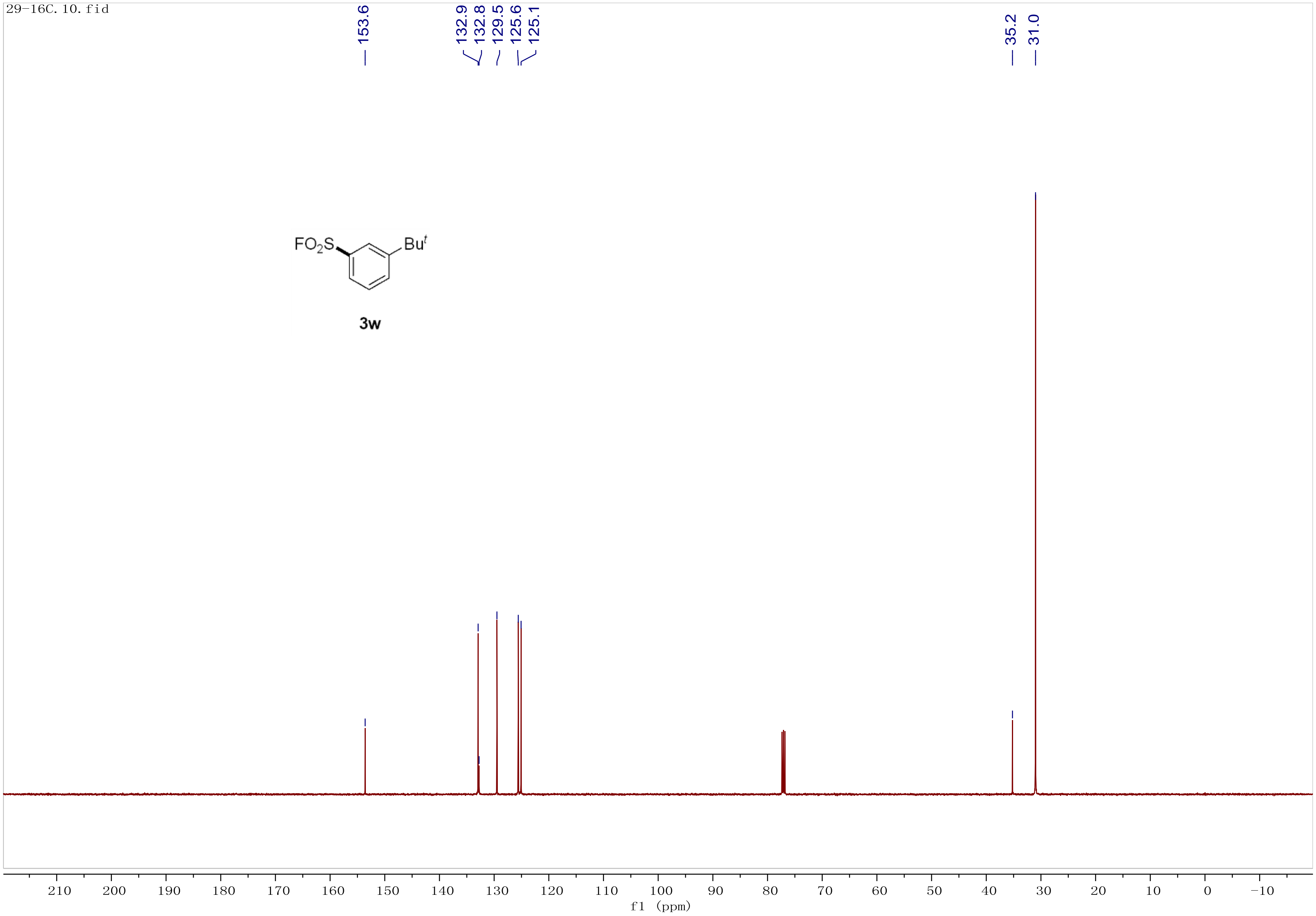

Supplementary Fig. 73 ^{13}C NMR spectrum of compound 3w ($CDCl_3$, 126 MHz, 298K)

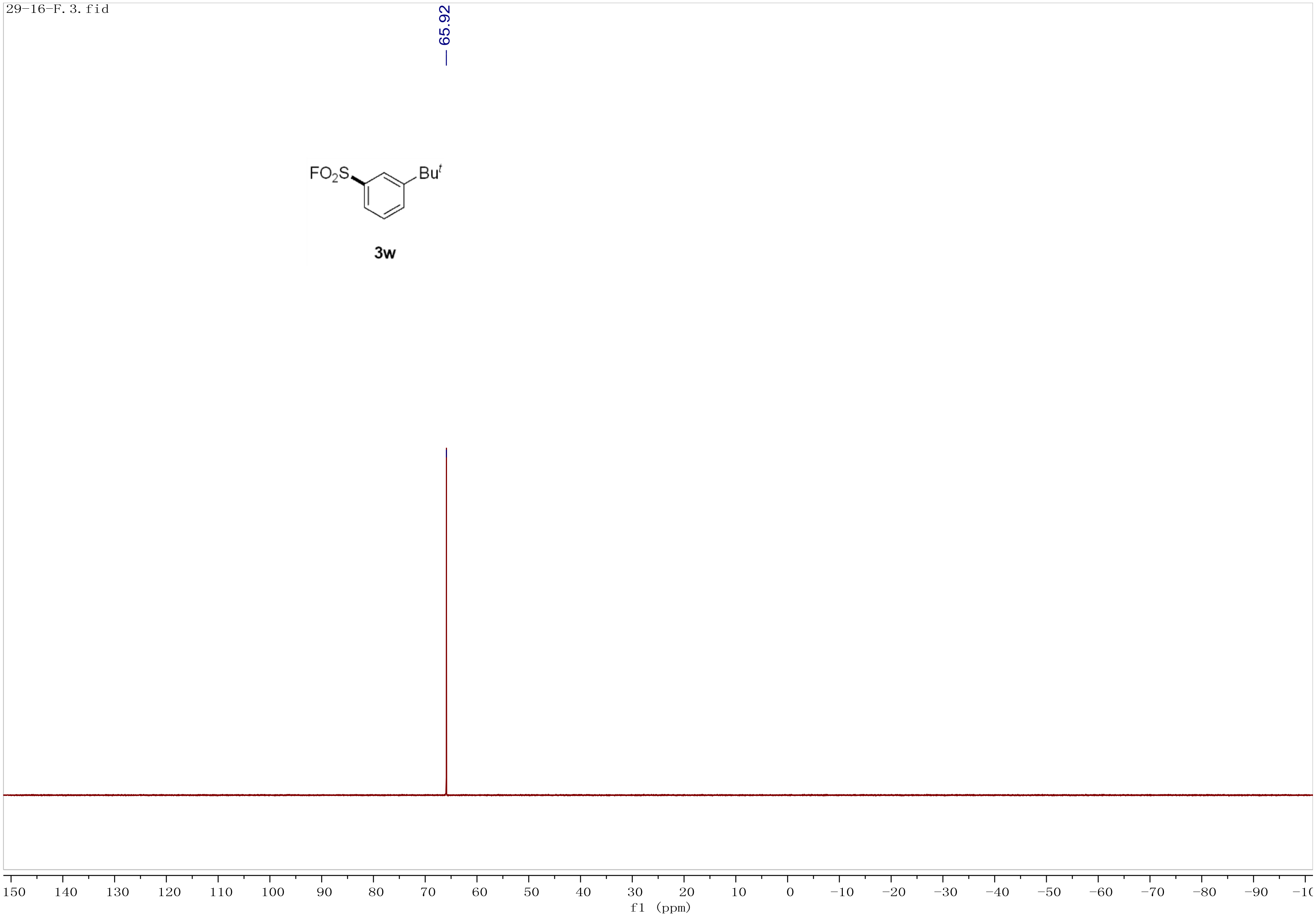

Supplementary Fig. 74 ^{19}F NMR spectrum of compound 3w ($CDCl_3$, 471 MHz, 298K)

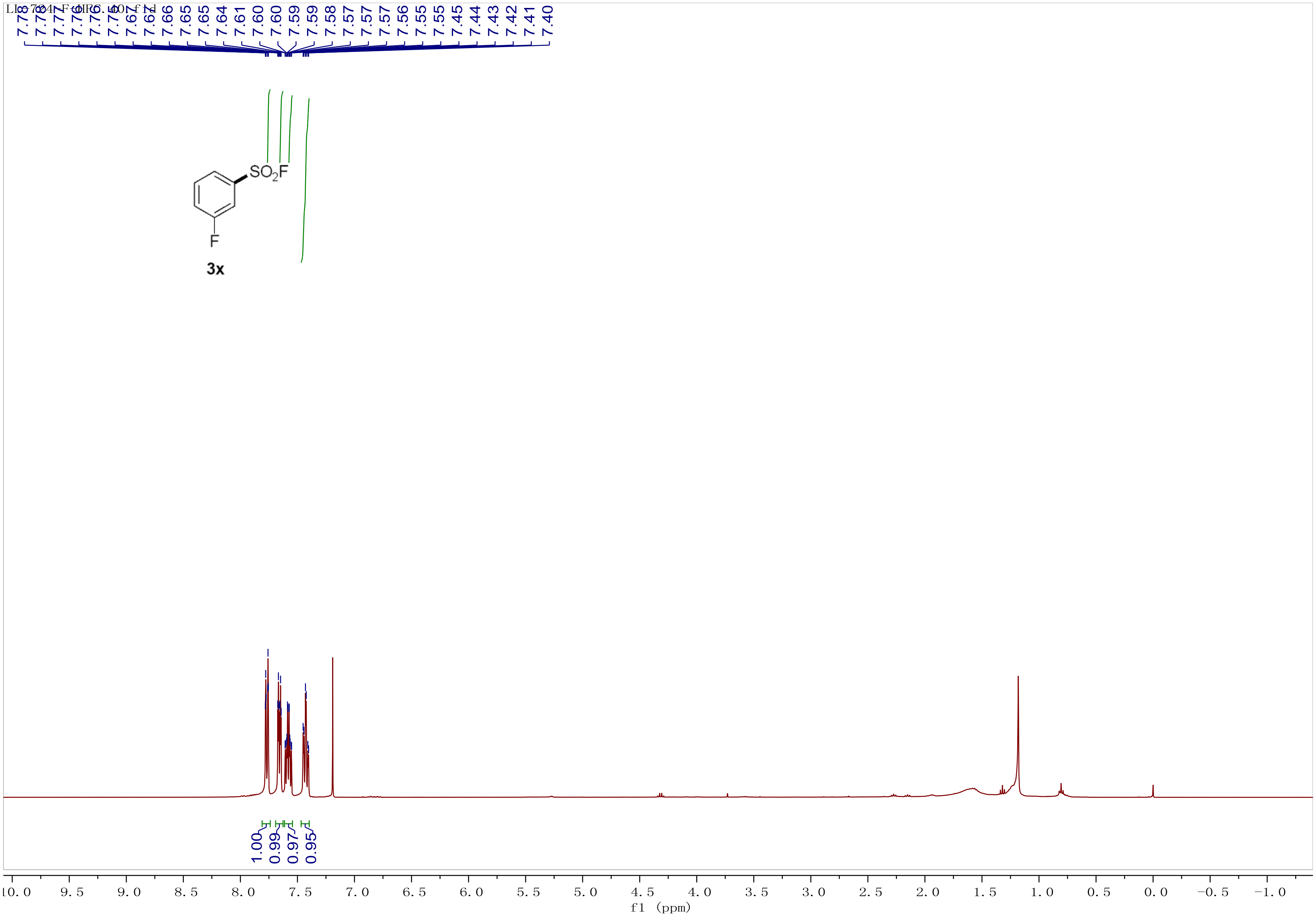

Supplementary Fig. 75 ^{1}H NMR spectrum of compound 3x ($CDCl_3$, 400 MHz, 298K)

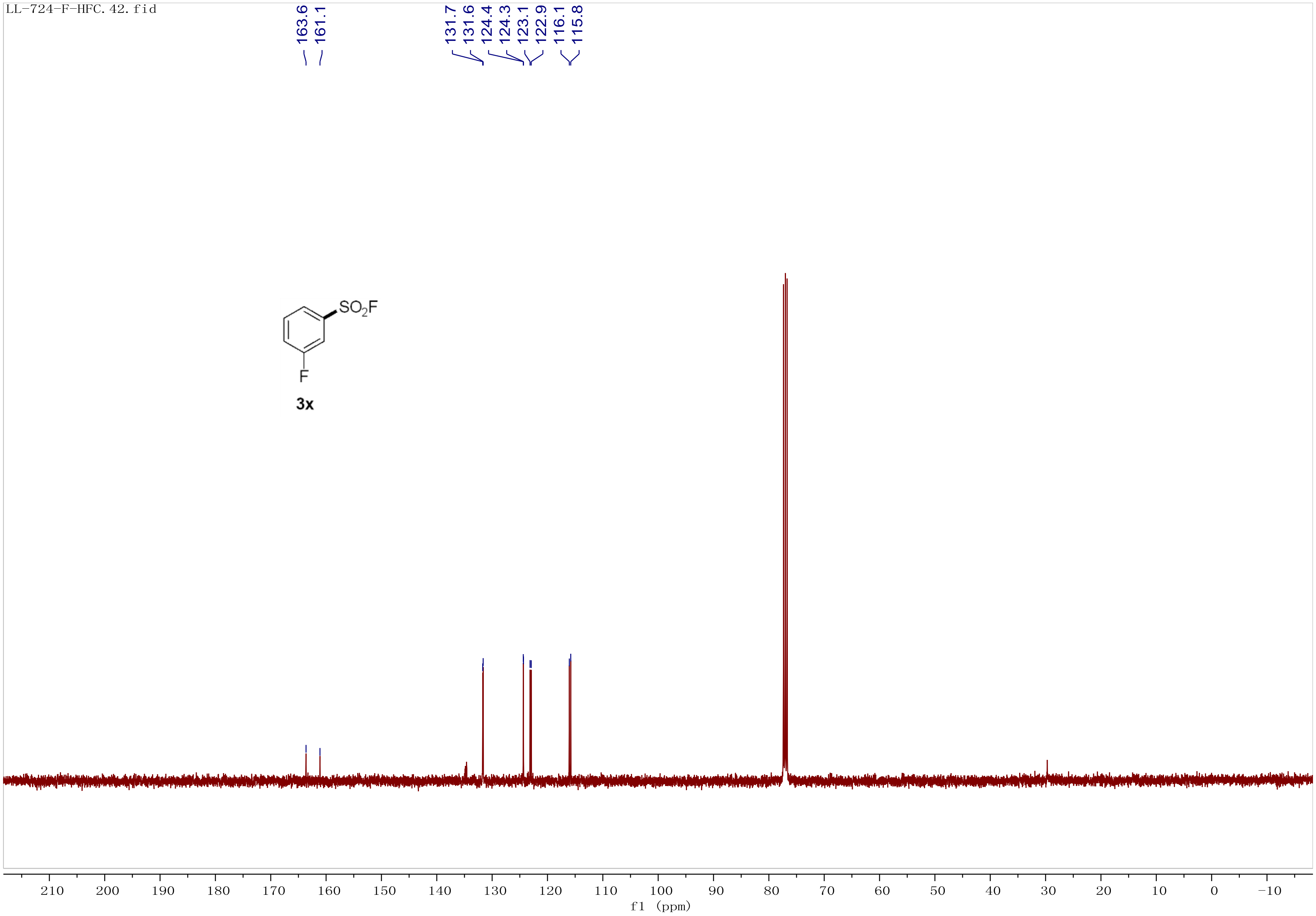

Supplementary Fig. 76 ^{13}C NMR spectrum of compound 3x ($CDCl_3$, 101 MHz, 298K)

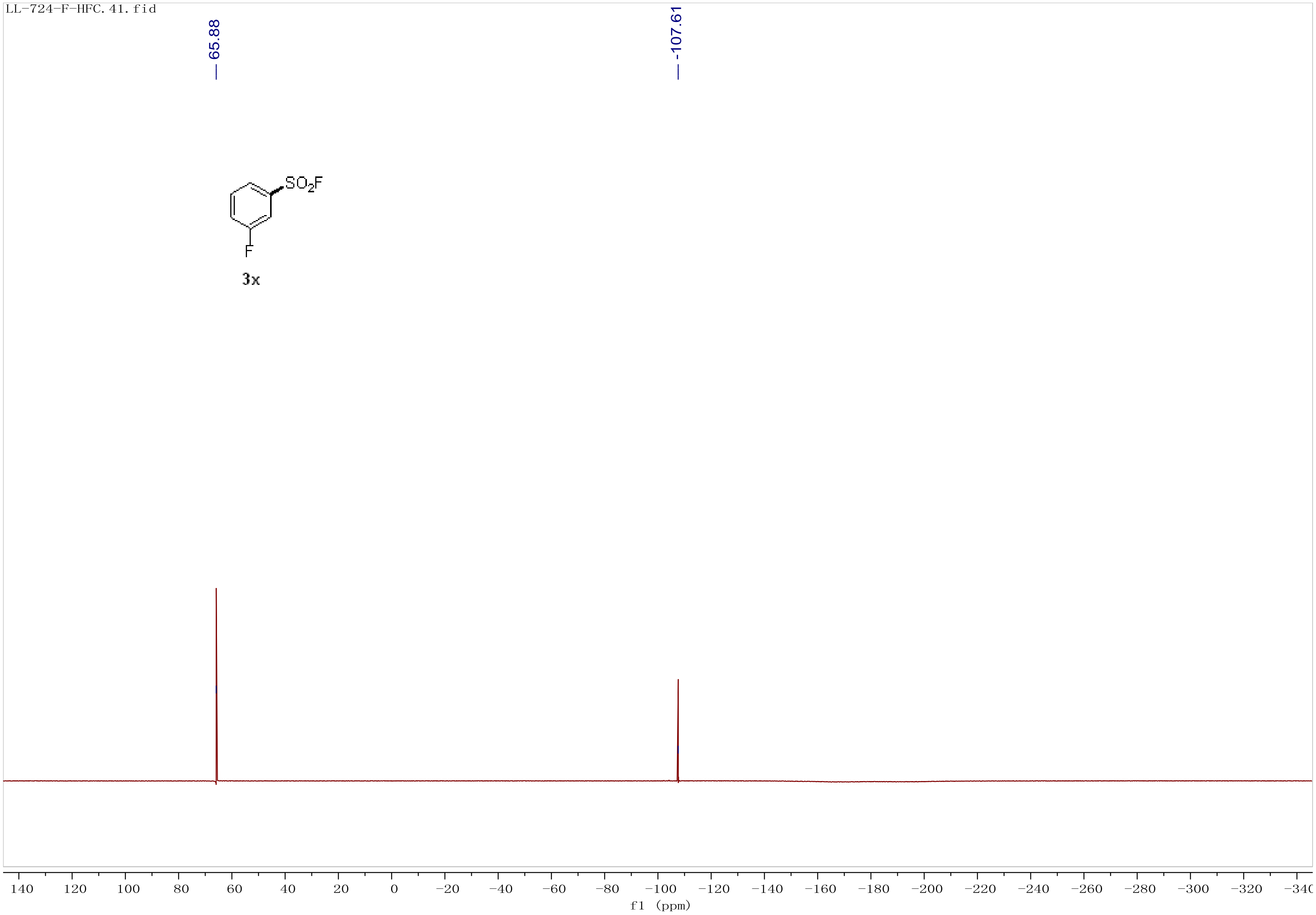

Supplementary Fig. 77 ^{19}F NMR spectrum of compound 3x ($CDCl_3$, 376 MHz, 298K)

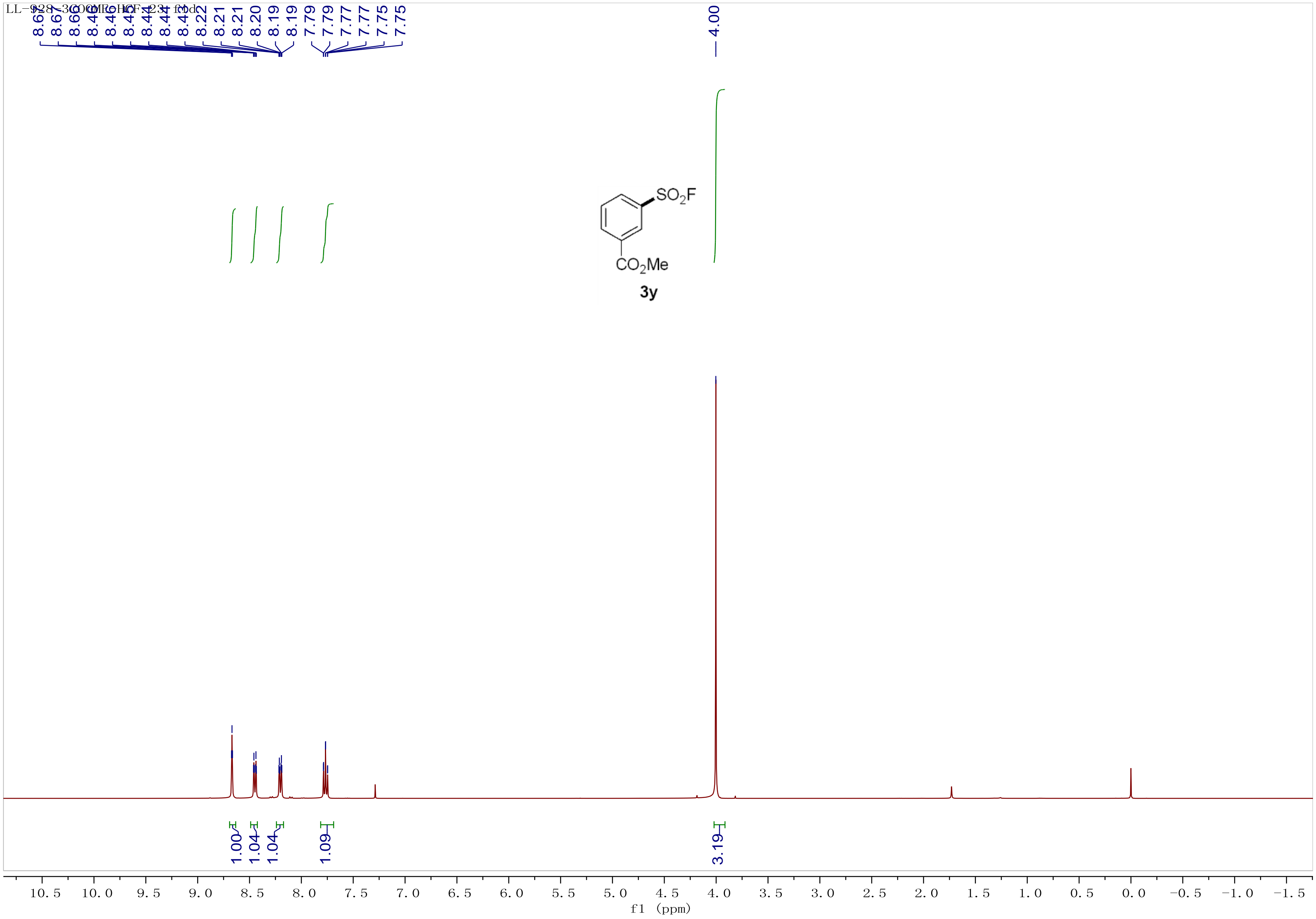

Supplementary Fig. 78 ^{1}H NMR spectrum of compound 3y ($CDCl_3$, 400 MHz, 298K)

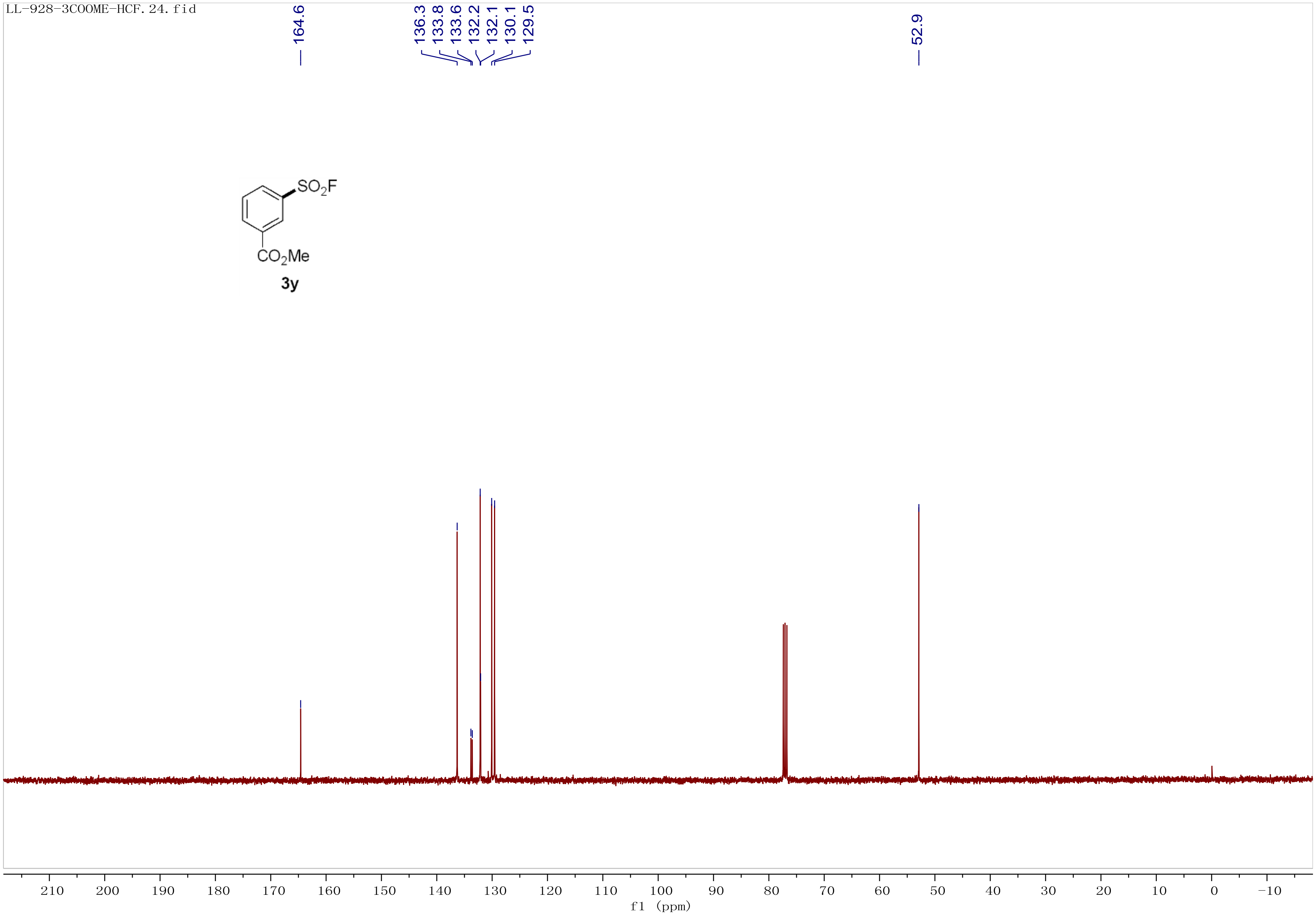

Supplementary Fig. 79 ^{13}C NMR spectrum of compound 3y ($CDCl_3$, 101 MHz, 298K)

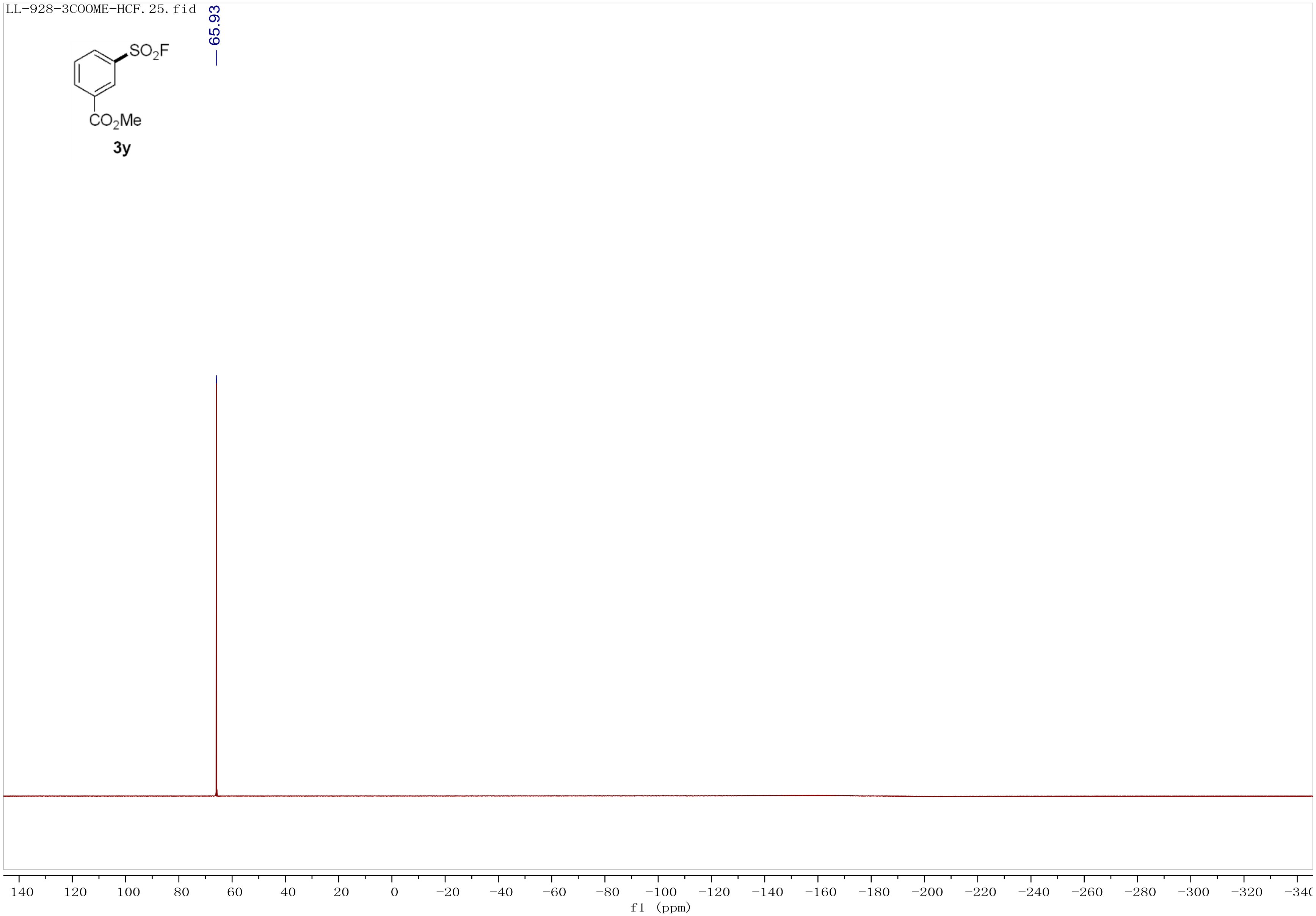

Supplementary Fig. 80 ^{19}F NMR spectrum of compound 3y ($CDCl_3$, 376 MHz, 298K)

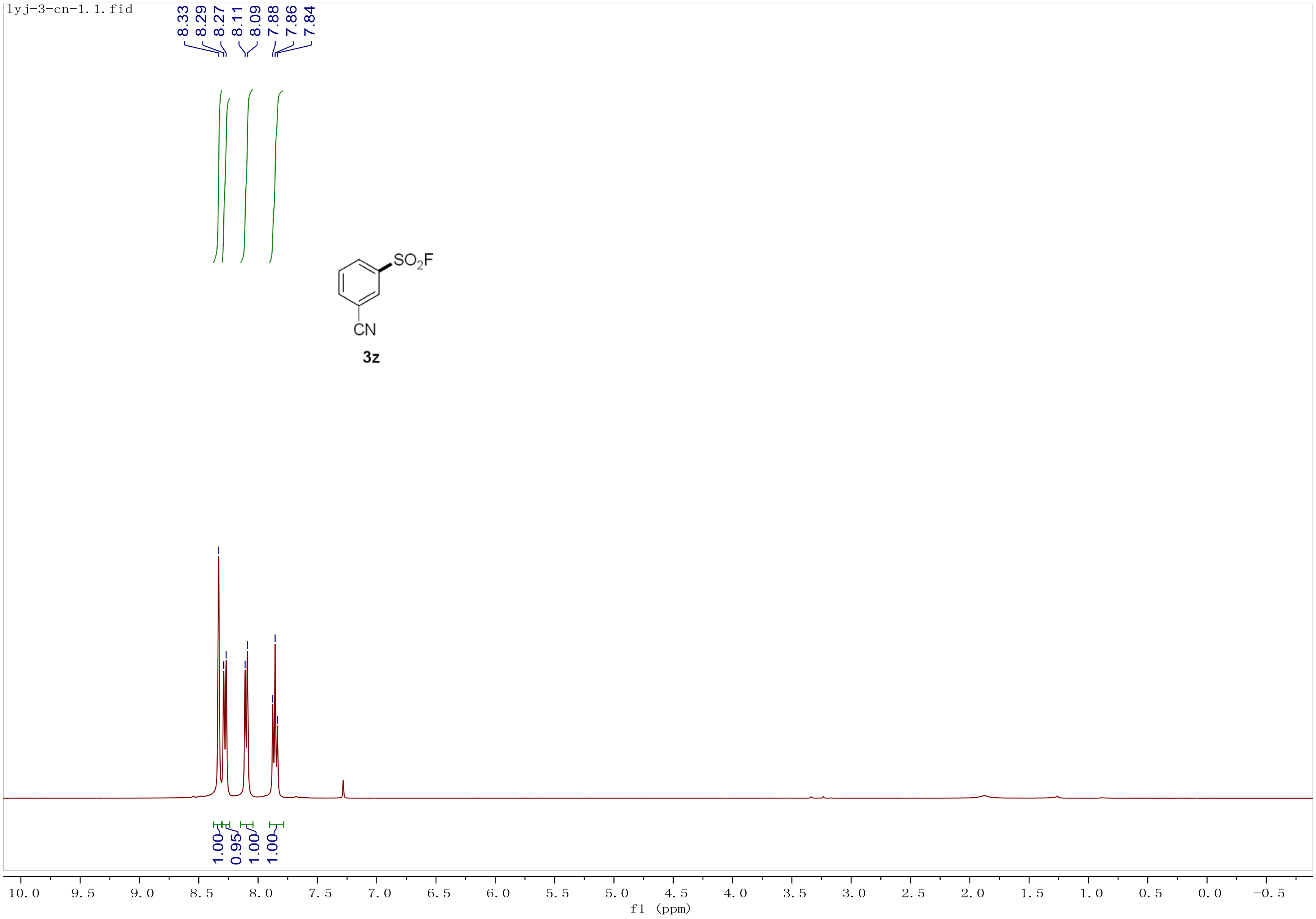

Supplementary Fig. 81 ^{1}H NMR spectrum of compound 3z ($CDCl_3$, 400 MHz, 298K)

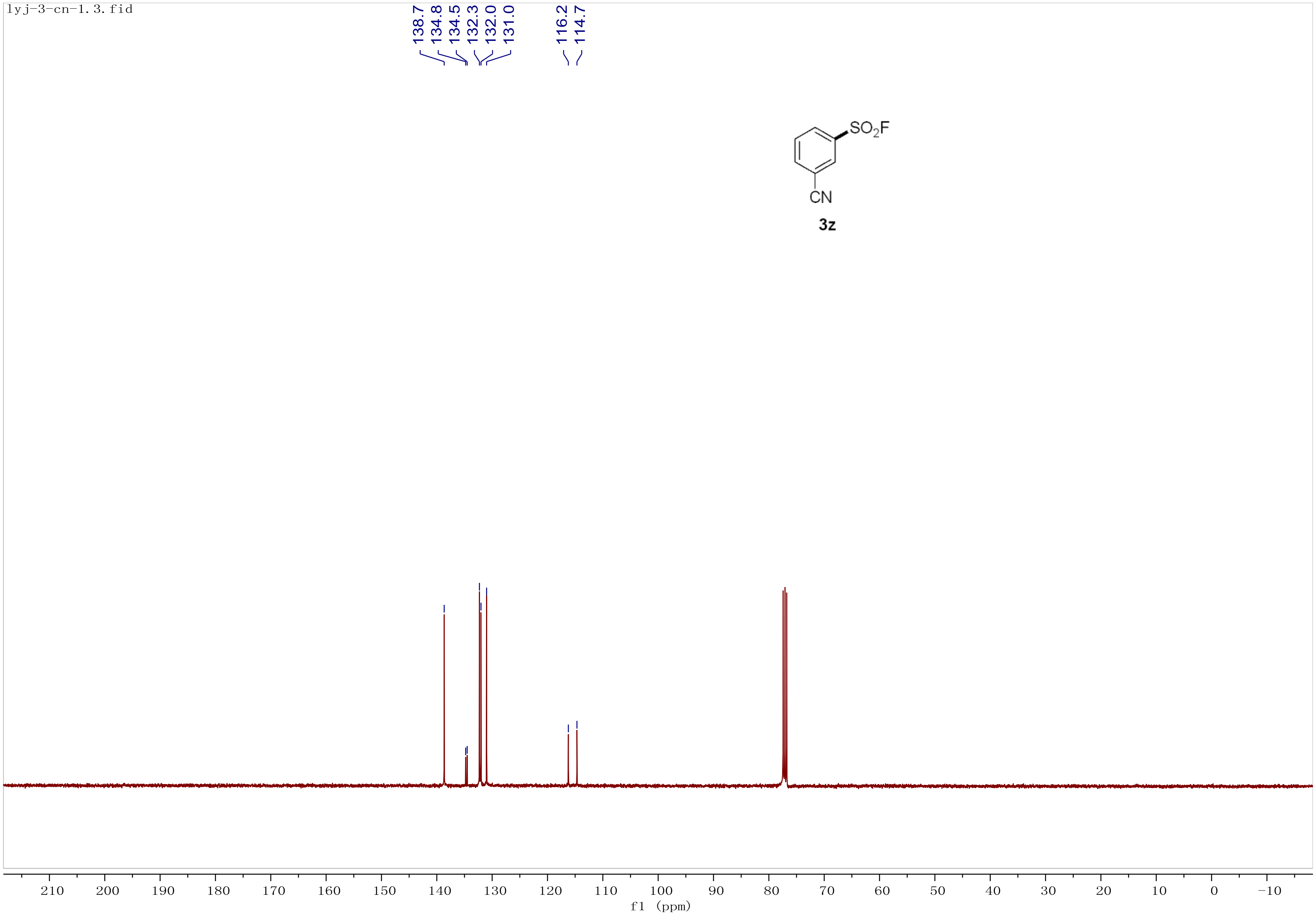

Supplementary Fig. 82 ^{13}C NMR spectrum of compound 3z ($CDCl_3$, 101 MHz, 298K)

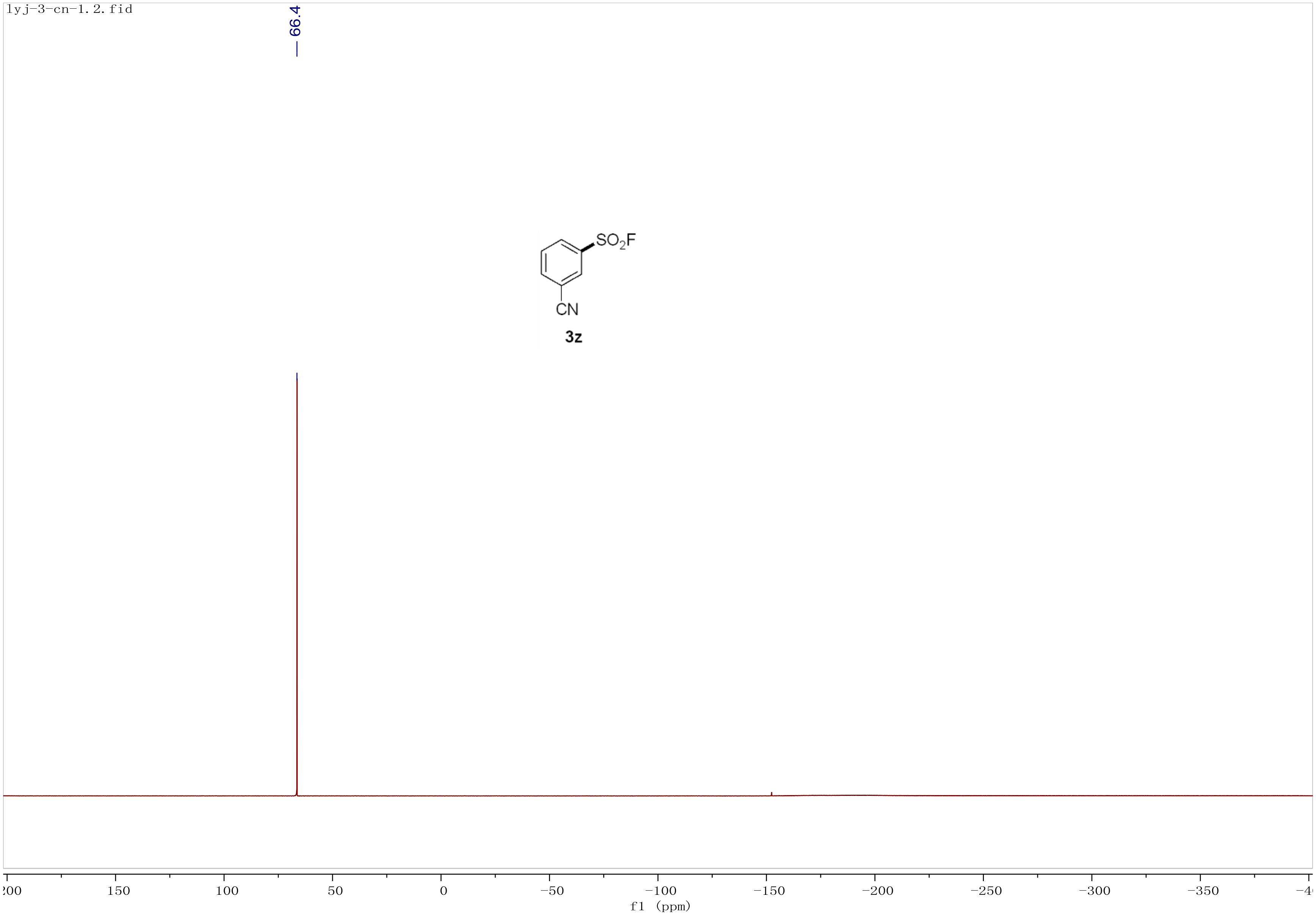

Supplementary Fig. 83 ^{19}F NMR spectrum of compound 3z ($CDCl_3$, 376 MHz, 298K)

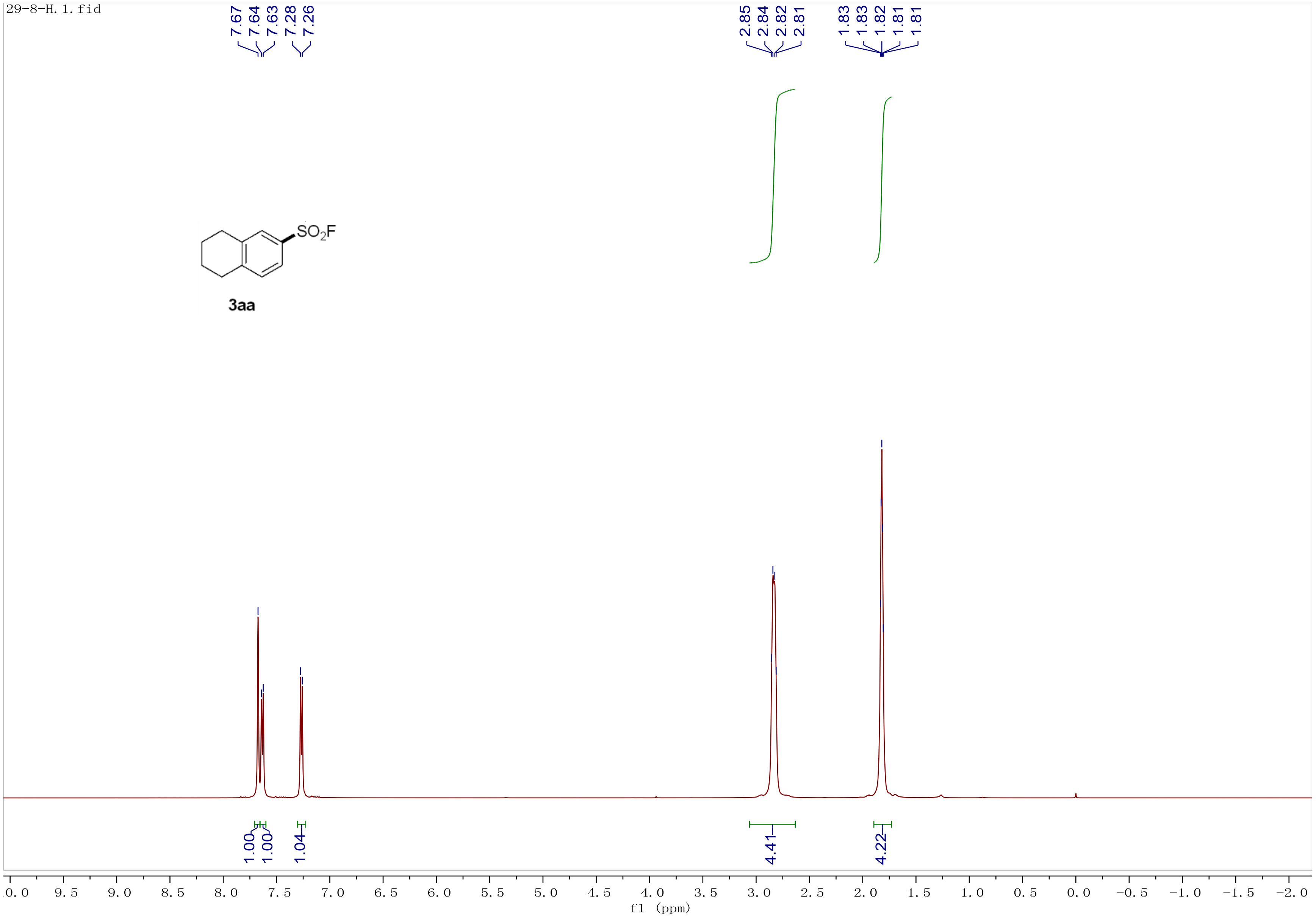

Supplementary Fig. 84 ^{1}H NMR spectrum of compound 3aa ($CDCl_3$, 500 MHz, 298K)

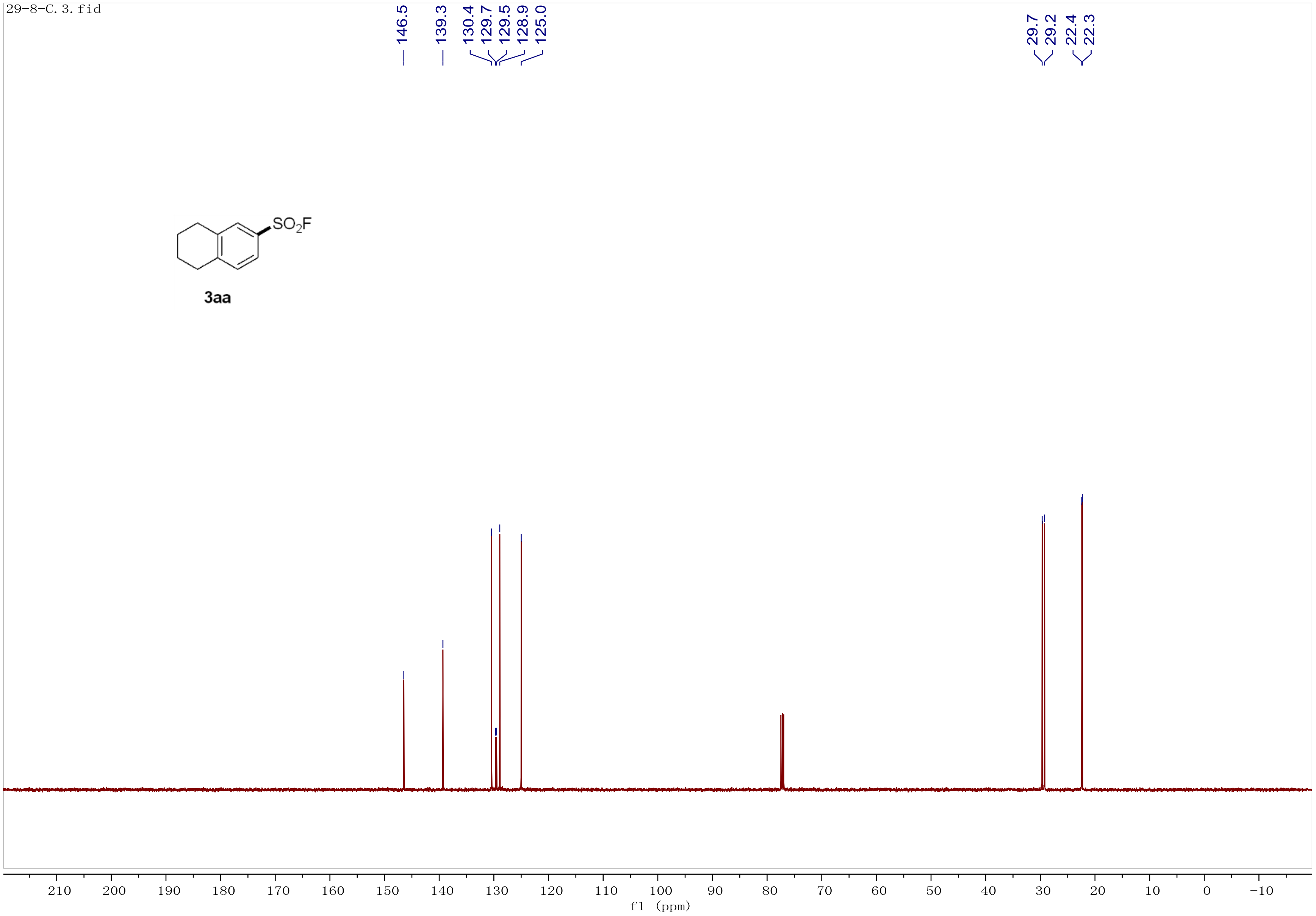

Supplementary Fig. 85 ^{13}C NMR spectrum of compound 3aa ($CDCl_3$, 126 MHz, 298K)

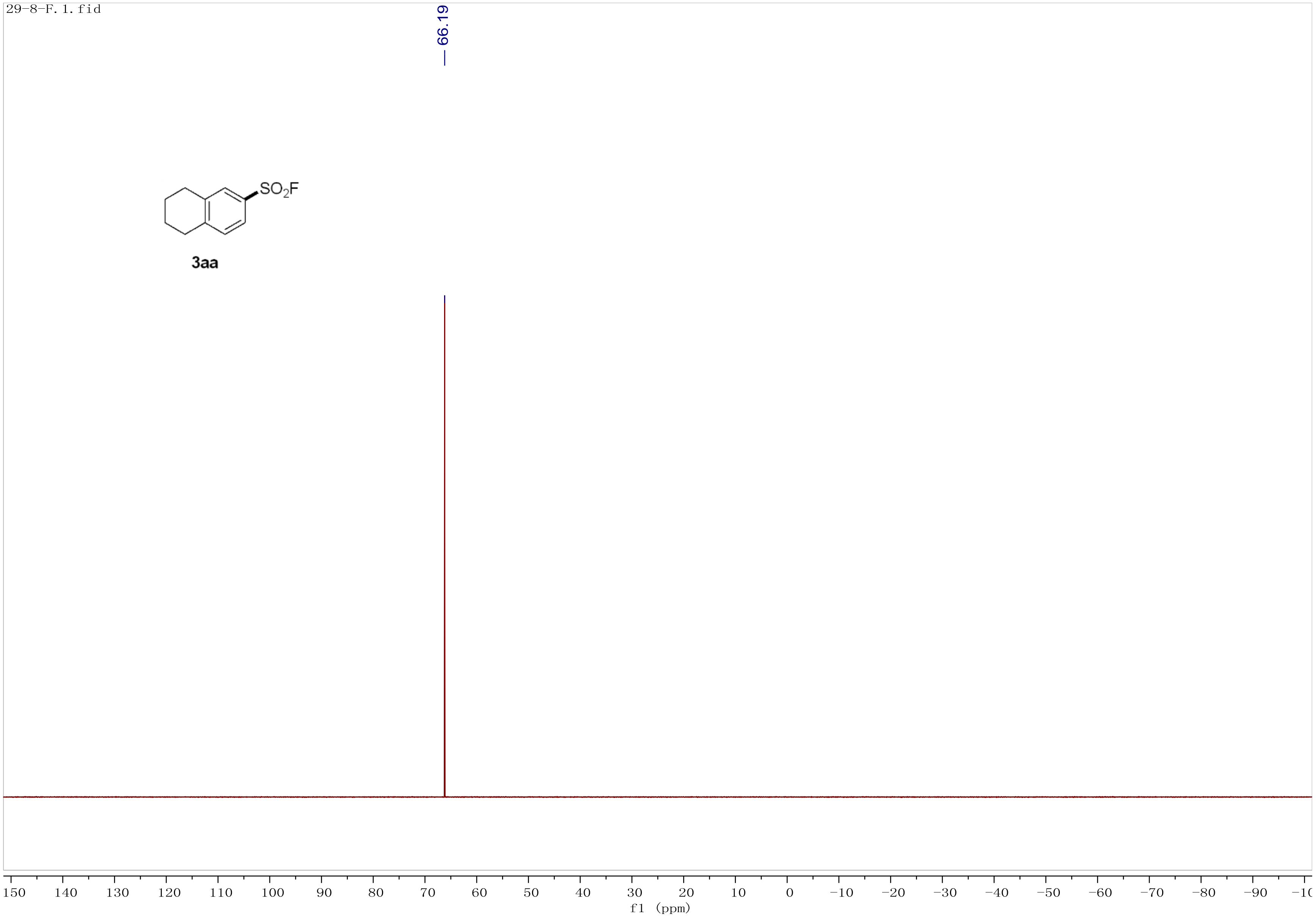

Supplementary Fig. 86 ^{19}F NMR spectrum of compound 3aa ($CDCl_3$, 471 MHz, 298K)

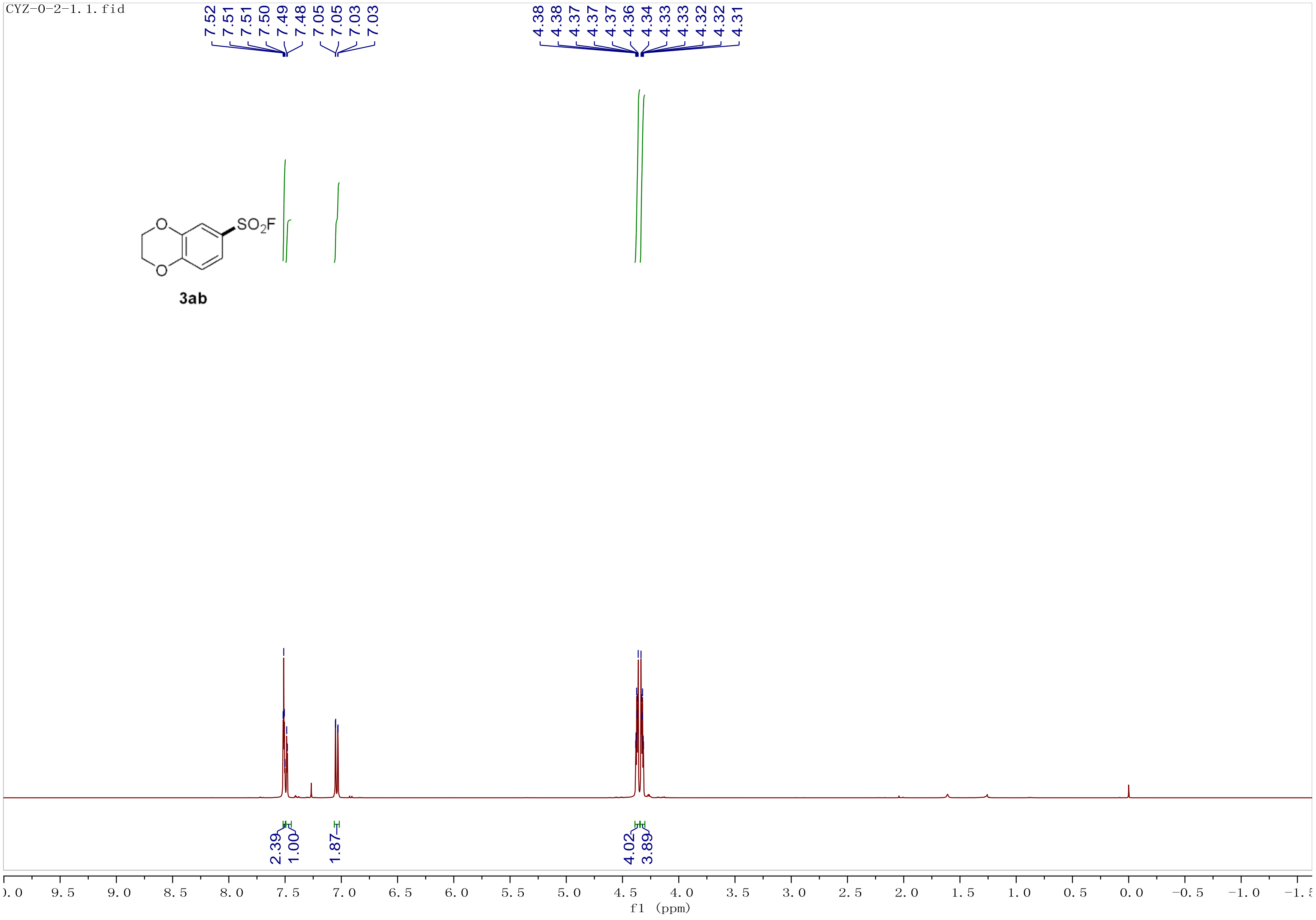

Supplementary Fig. 87 ^{1}H NMR spectrum of compound 3ab ($CDCl_3$, 400 MHz, 298K)

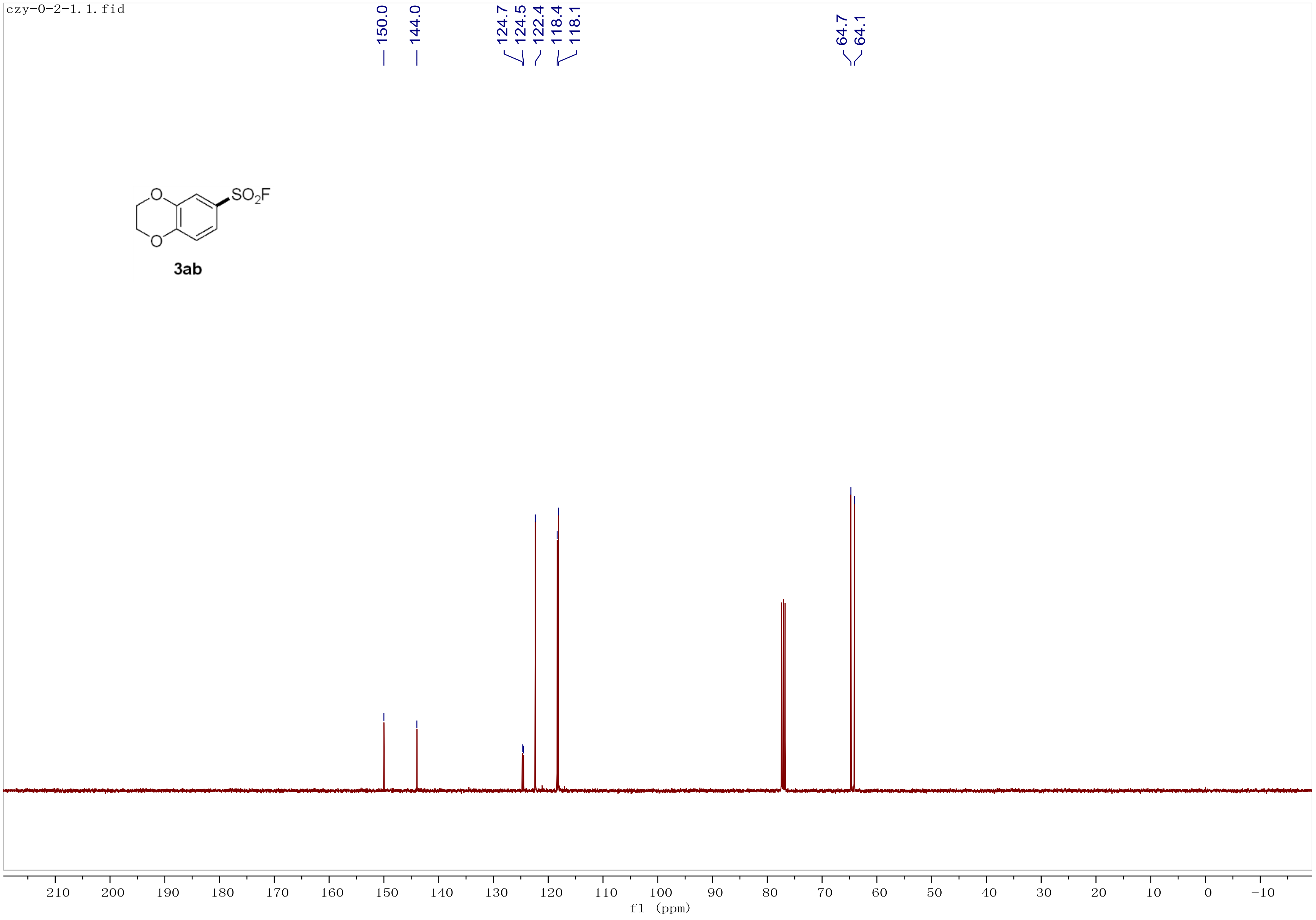

Supplementary Fig. 88 ^{13}C NMR spectrum of compound 3ab ($CDCl_3$, 101 MHz, 298K)

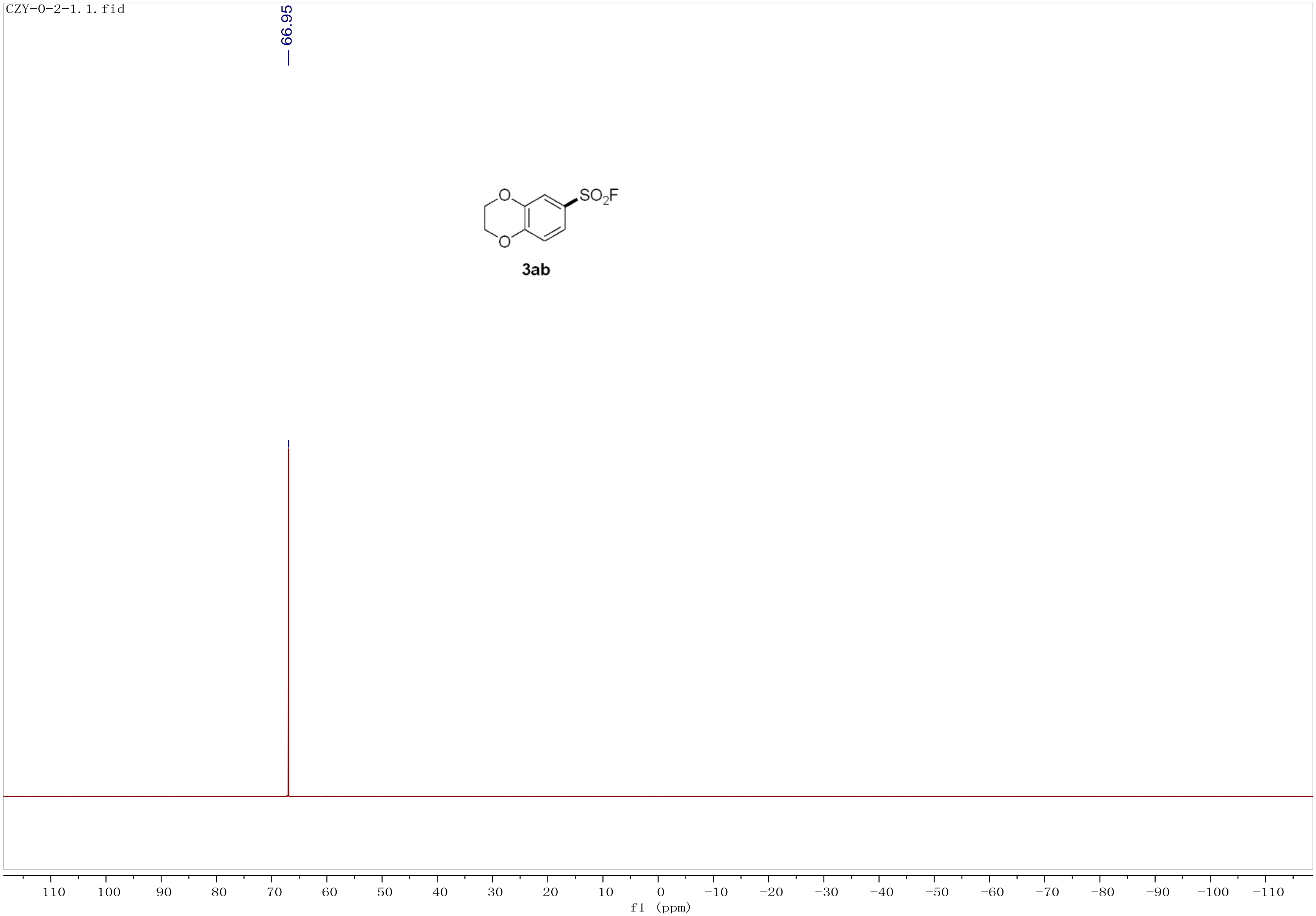

Supplementary Fig. 89 ^{19}F NMR spectrum of compound 3ab ($CDCl_3$, 376 MHz, 298K)

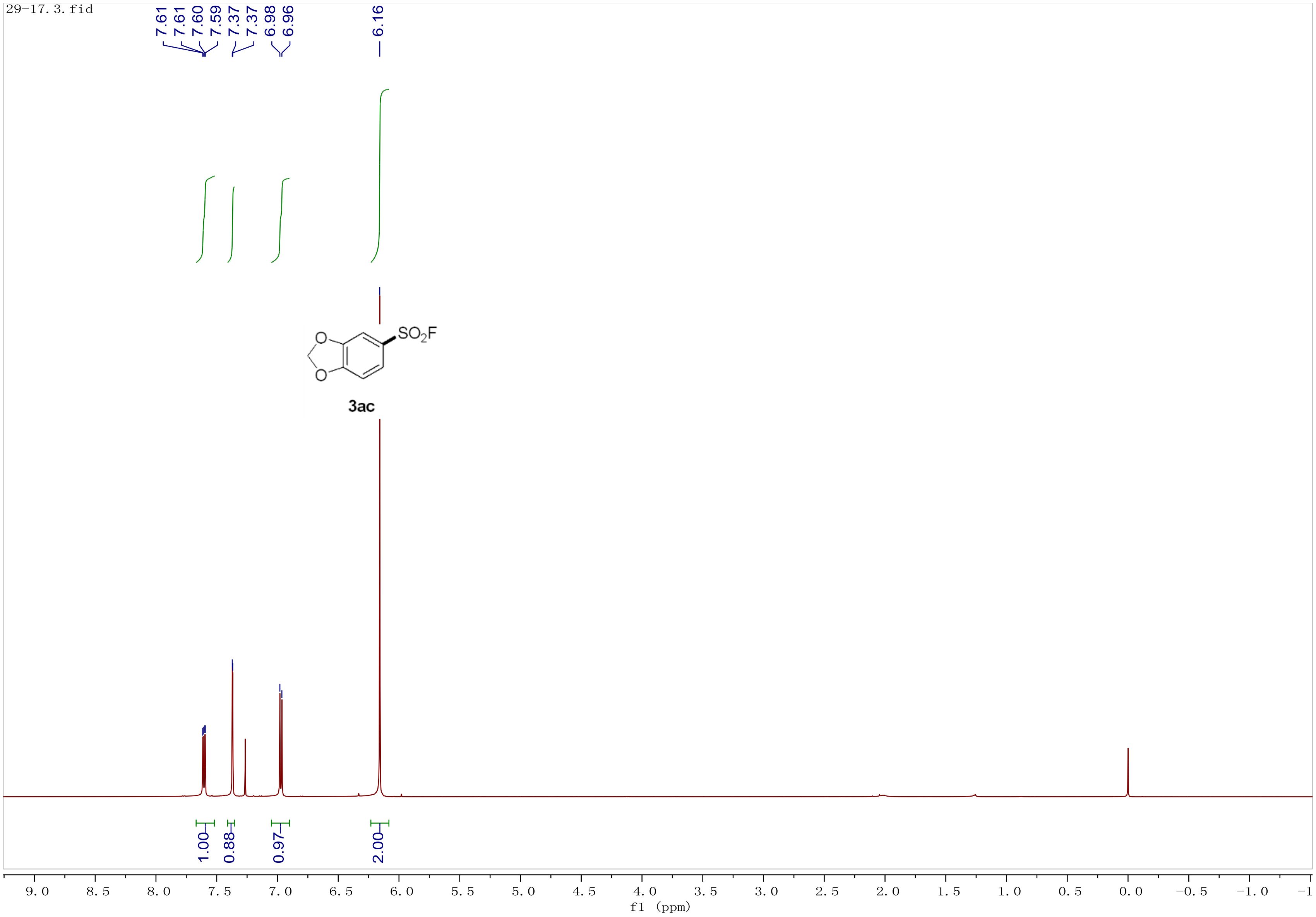

Supplementary Fig. 90 ^{1}H NMR spectrum of compound 3ac ($CDCl_3$, 500 MHz, 298K)

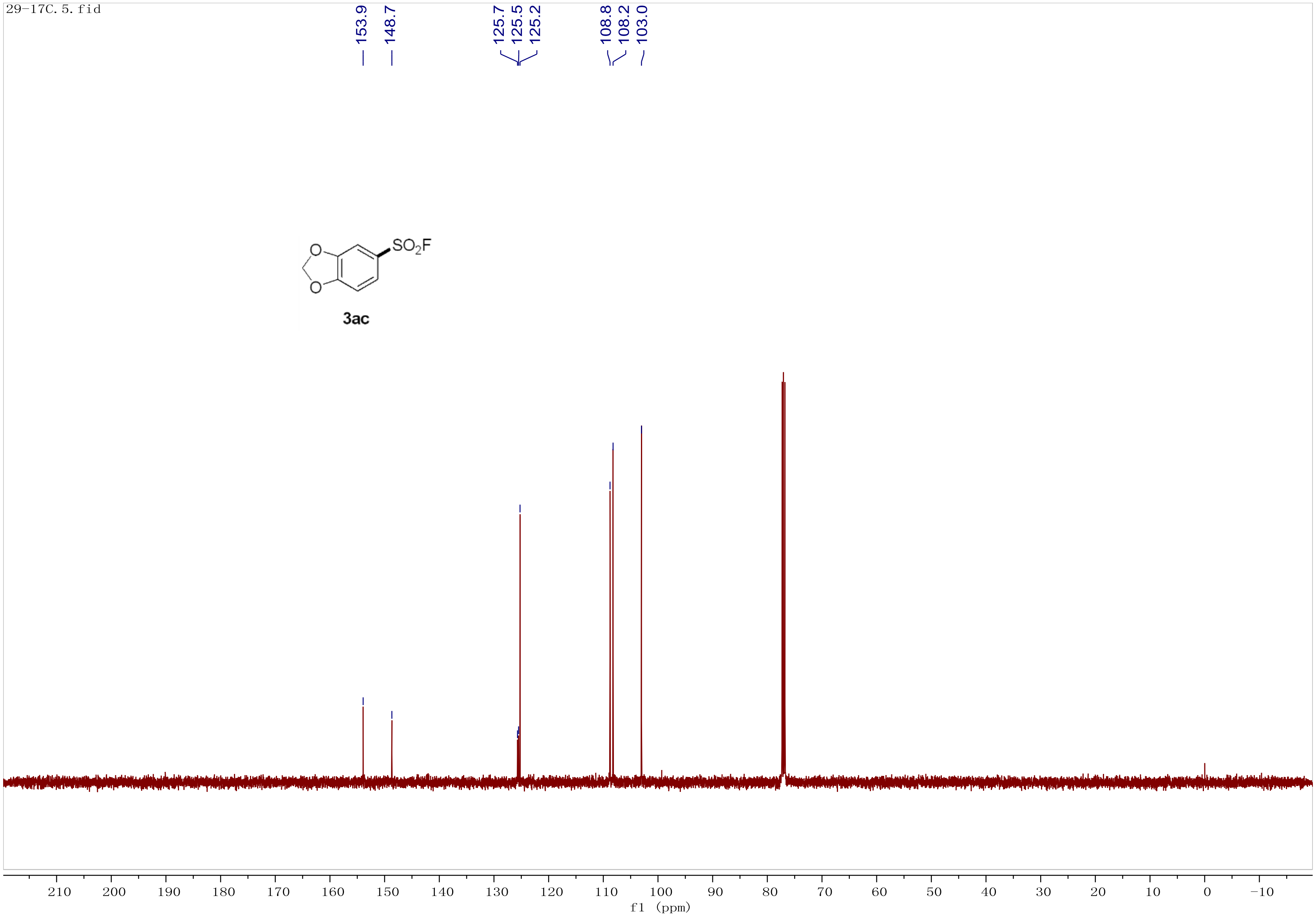

Supplementary Fig. 91 ^{13}**C NMR spectrum of compound 3ac (CDCl$_3$, 126 MHz, 298K)**

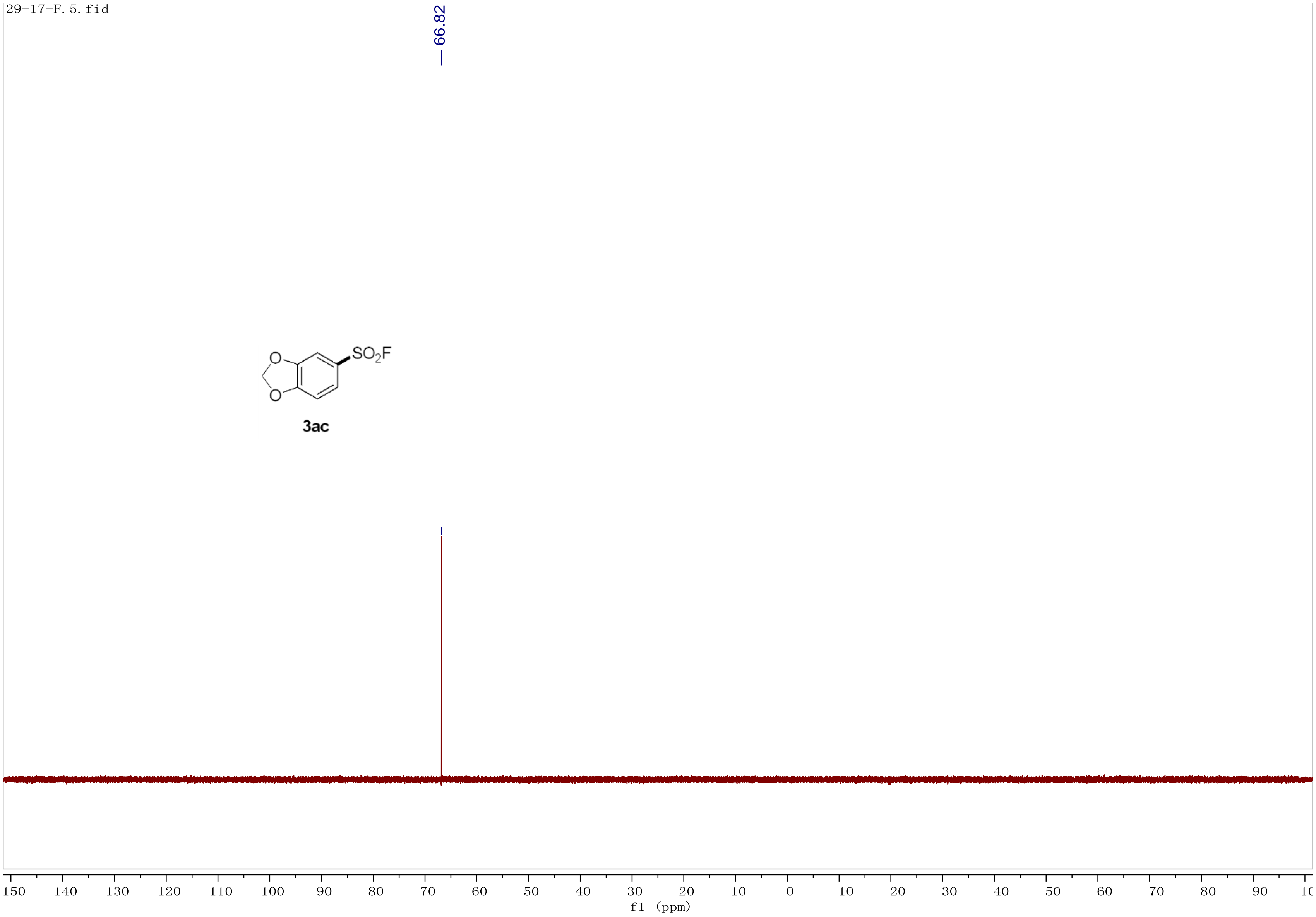

Supplementary Fig. 92 ^{19}F NMR spectrum of compound 3ac ($CDCl_3$, 471 MHz, 298K)

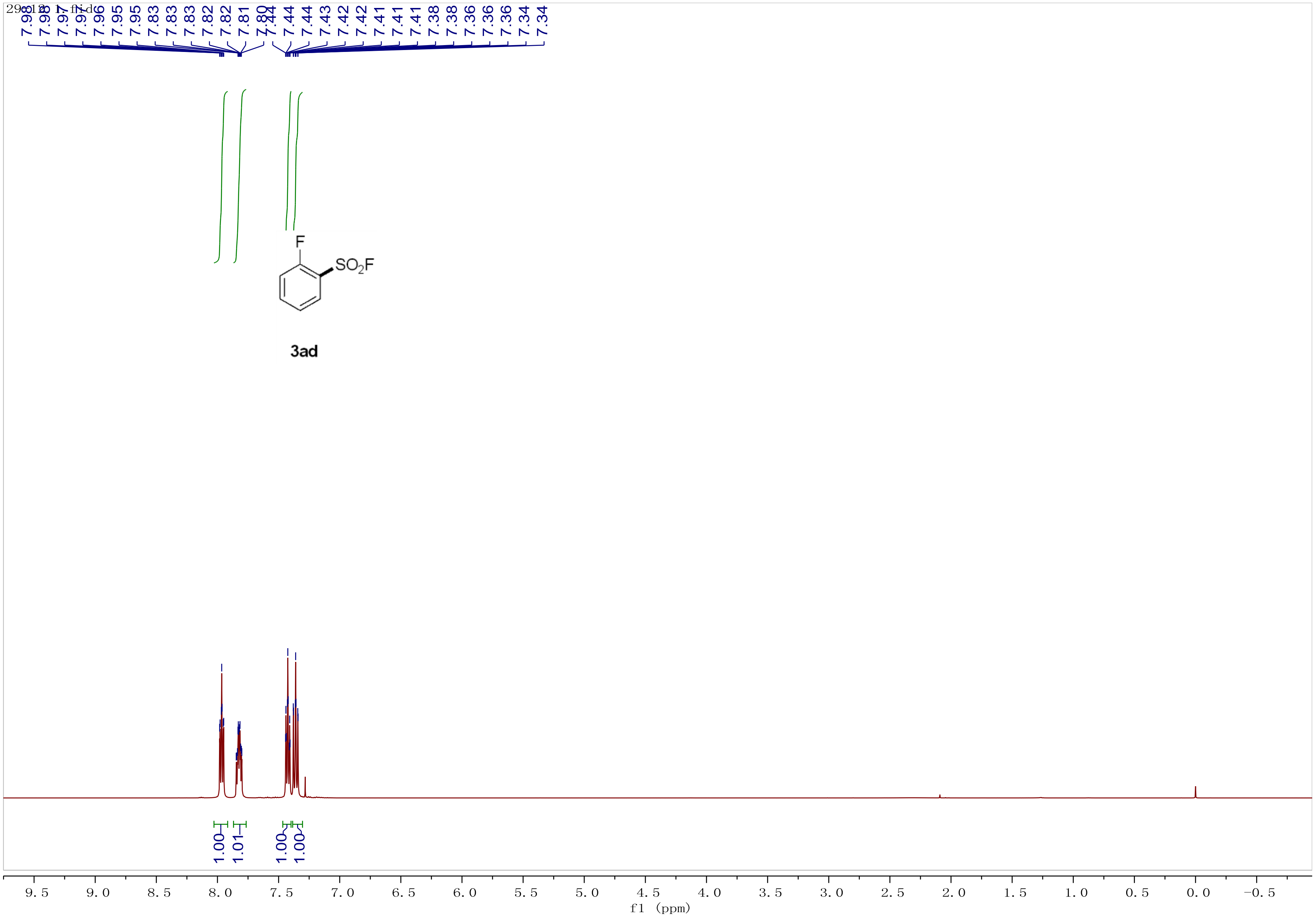

Supplementary Fig. 93 ^{1}H NMR spectrum of compound 3ad ($CDCl_3$, 500 MHz, 298K)

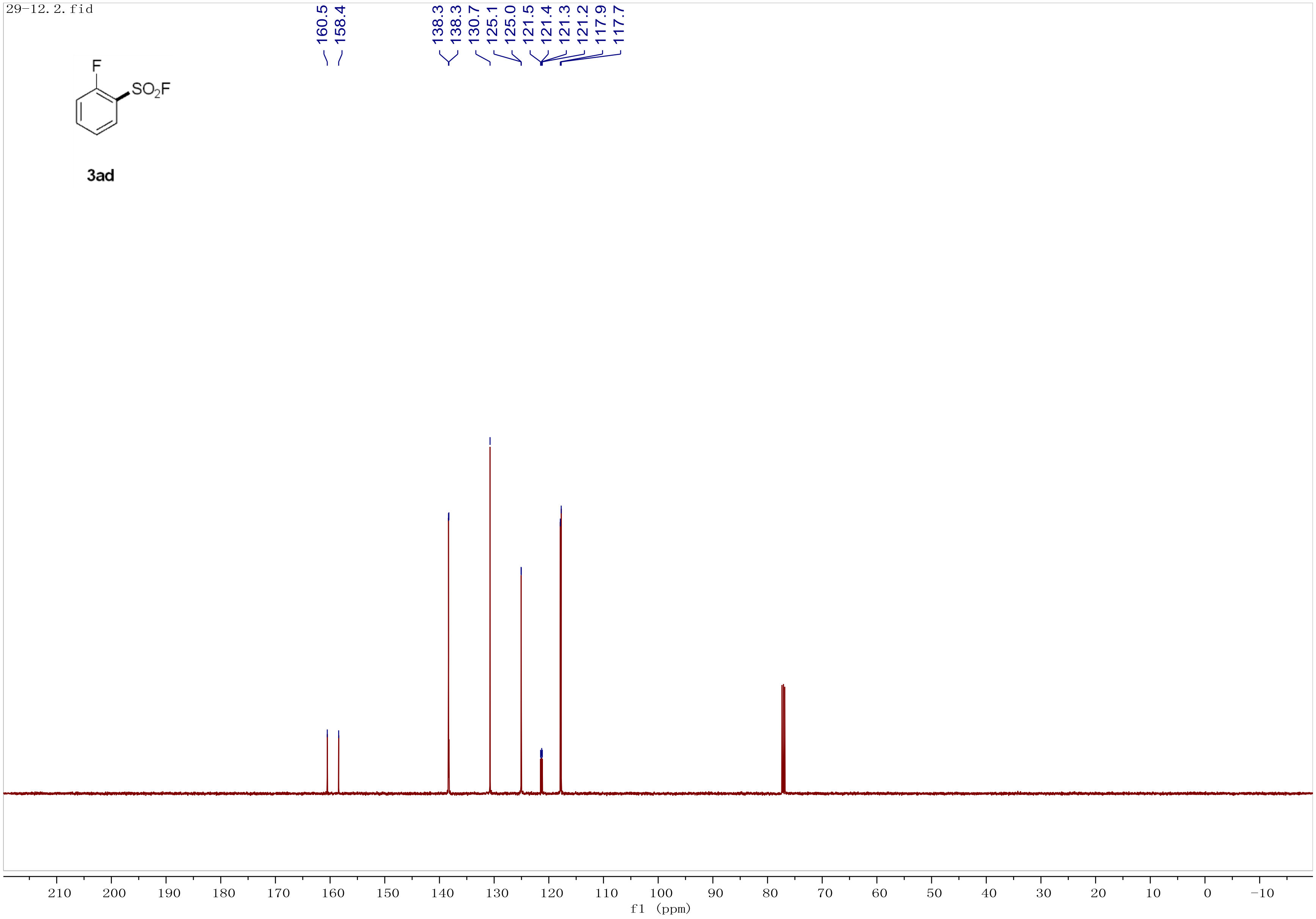

Supplementary Fig. 94 ^{13}C NMR spectrum of compound 3ad ($CDCl_3$, 126 MHz, 298K)

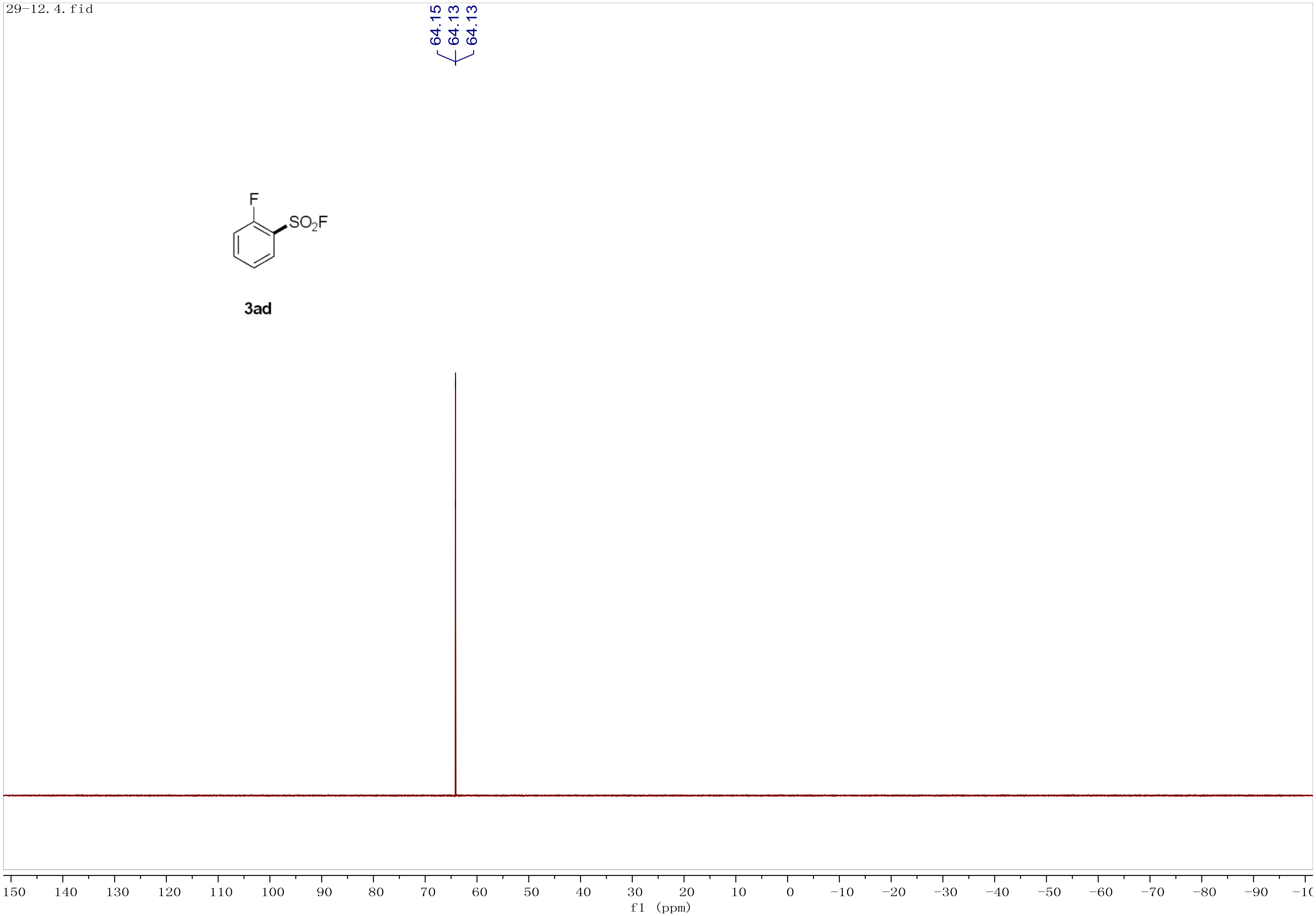

Supplementary Fig. 95 ^{19}F NMR spectrum of compound 3ad ($CDCl_3$, 376 MHz, 298K)

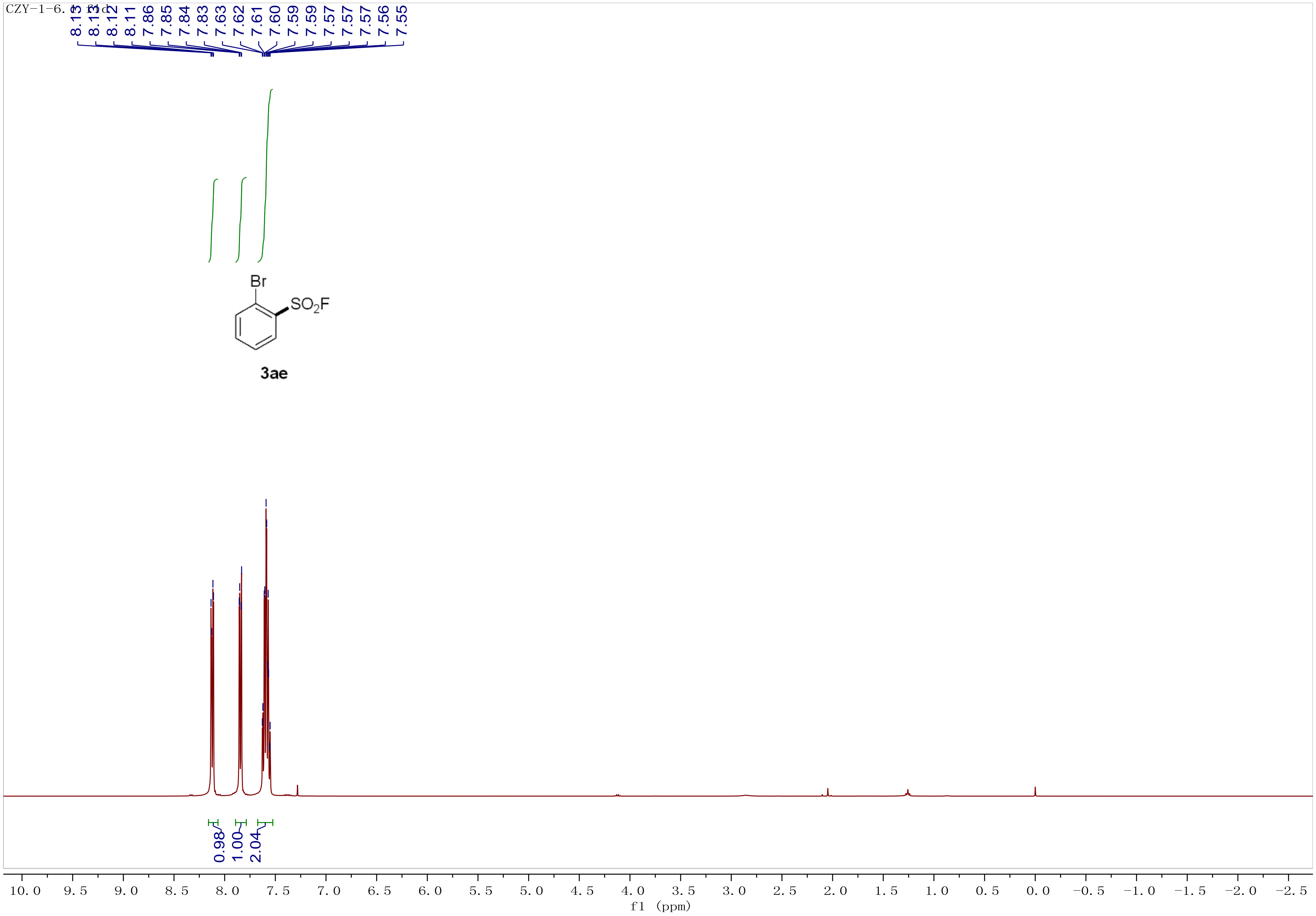

Supplementary Fig. 96 ^{1}H NMR spectrum of compound 3ae ($CDCl_3$, 400 MHz, 298K)

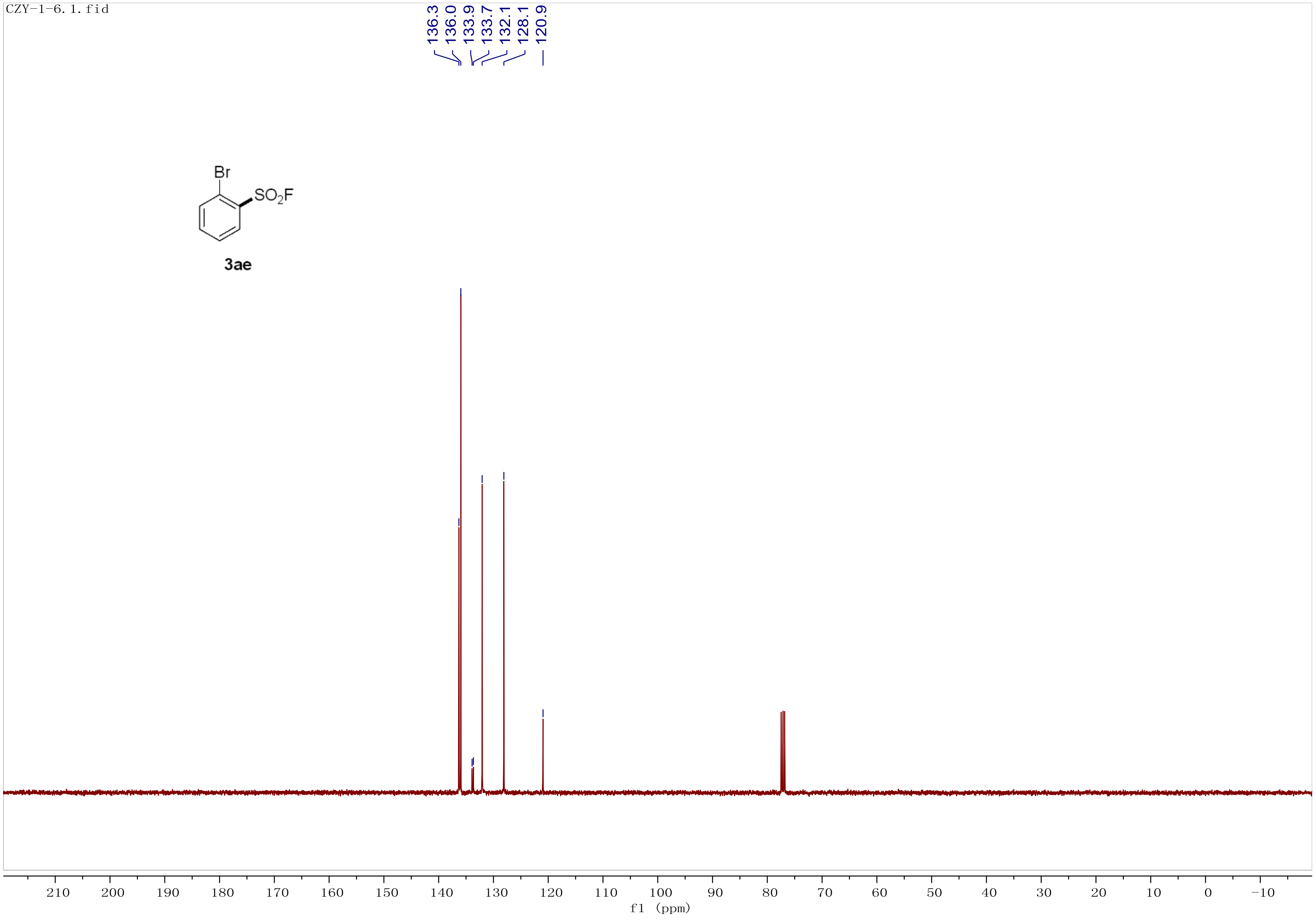

Supplementary Fig. 97 ^{13}C NMR spectrum of compound 3ae ($CDCl_3$, 101 MHz, 298K)

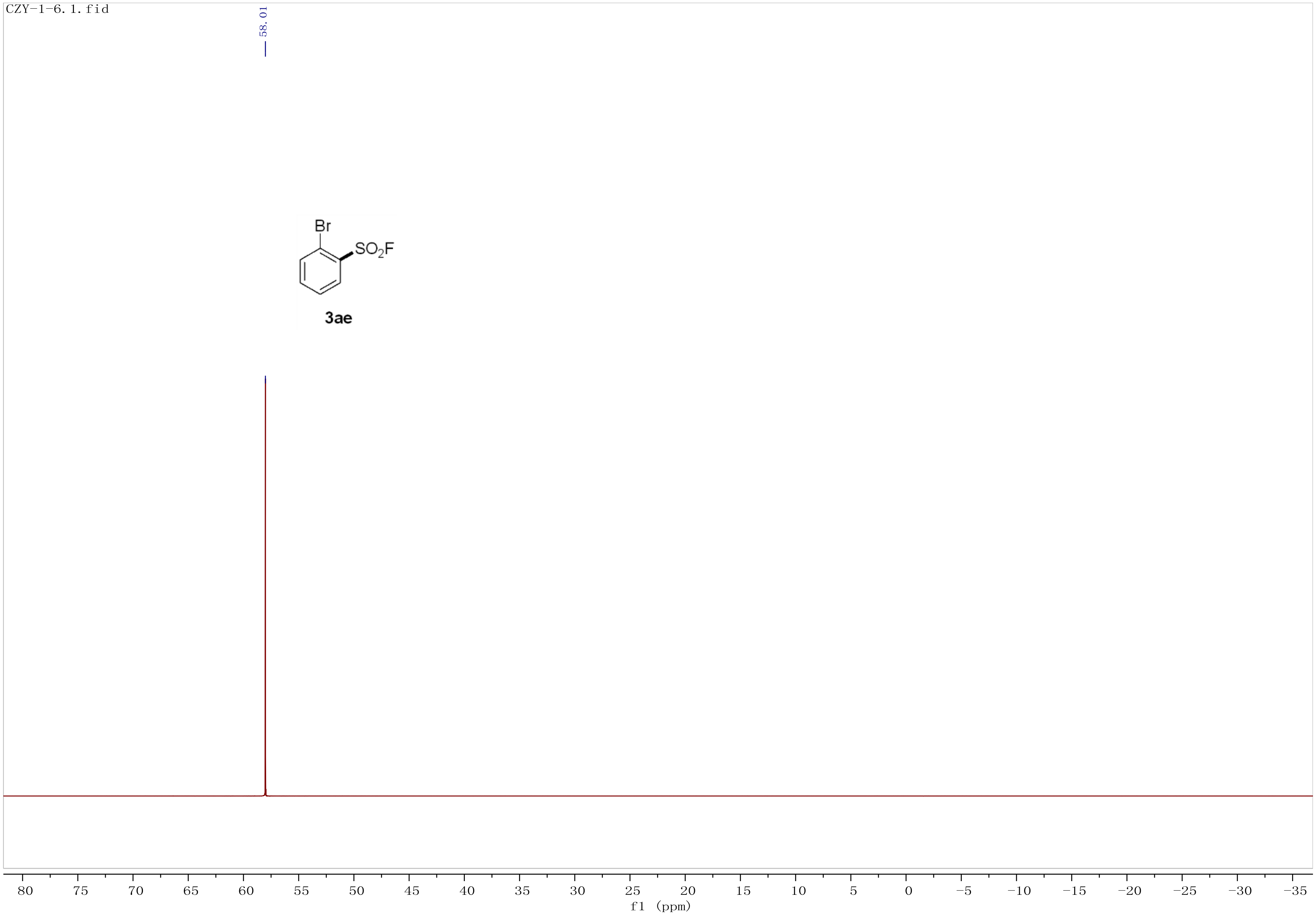

Supplementary Fig. 98 ^{19}F NMR spectrum of compound 3ae ($CDCl_3$, 376 MHz, 298K)

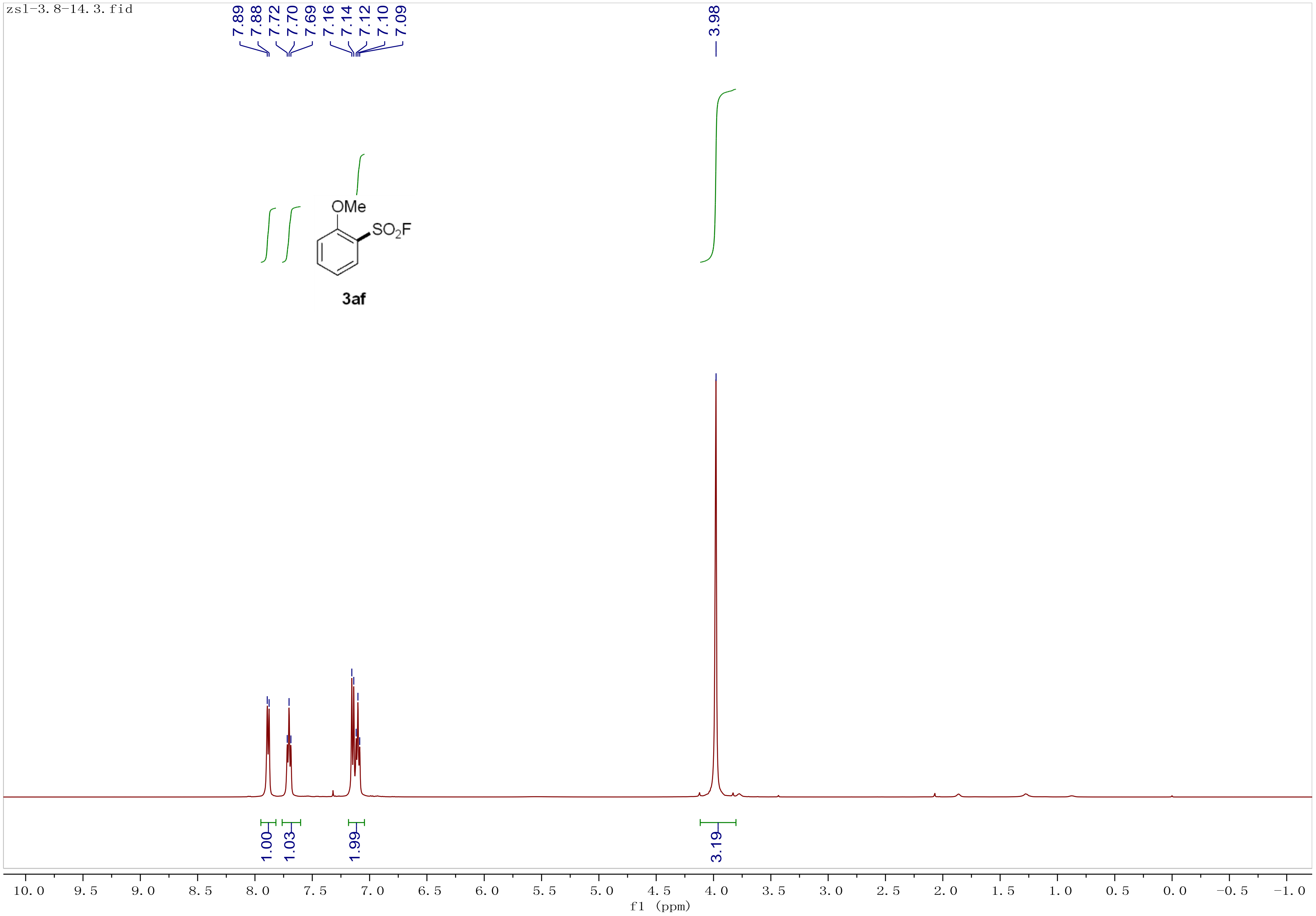

Supplementary Fig. 99 ^{1}H NMR spectrum of compound 3af ($CDCl_3$, 500 MHz, 298K)

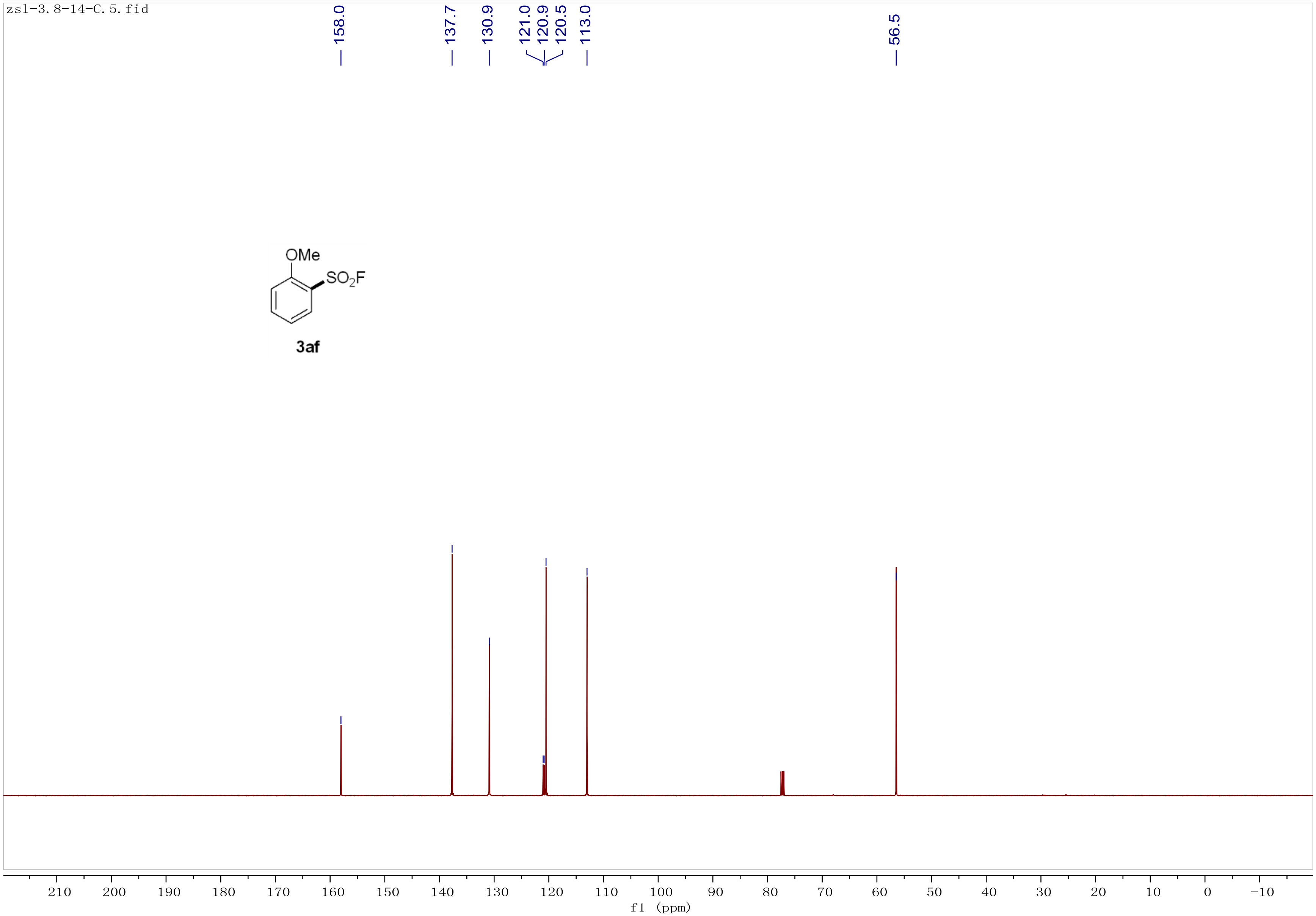

Supplementary Fig. 100 ^{13}C NMR spectrum of compound 3af ($CDCl_3$, 126 MHz, 298K)

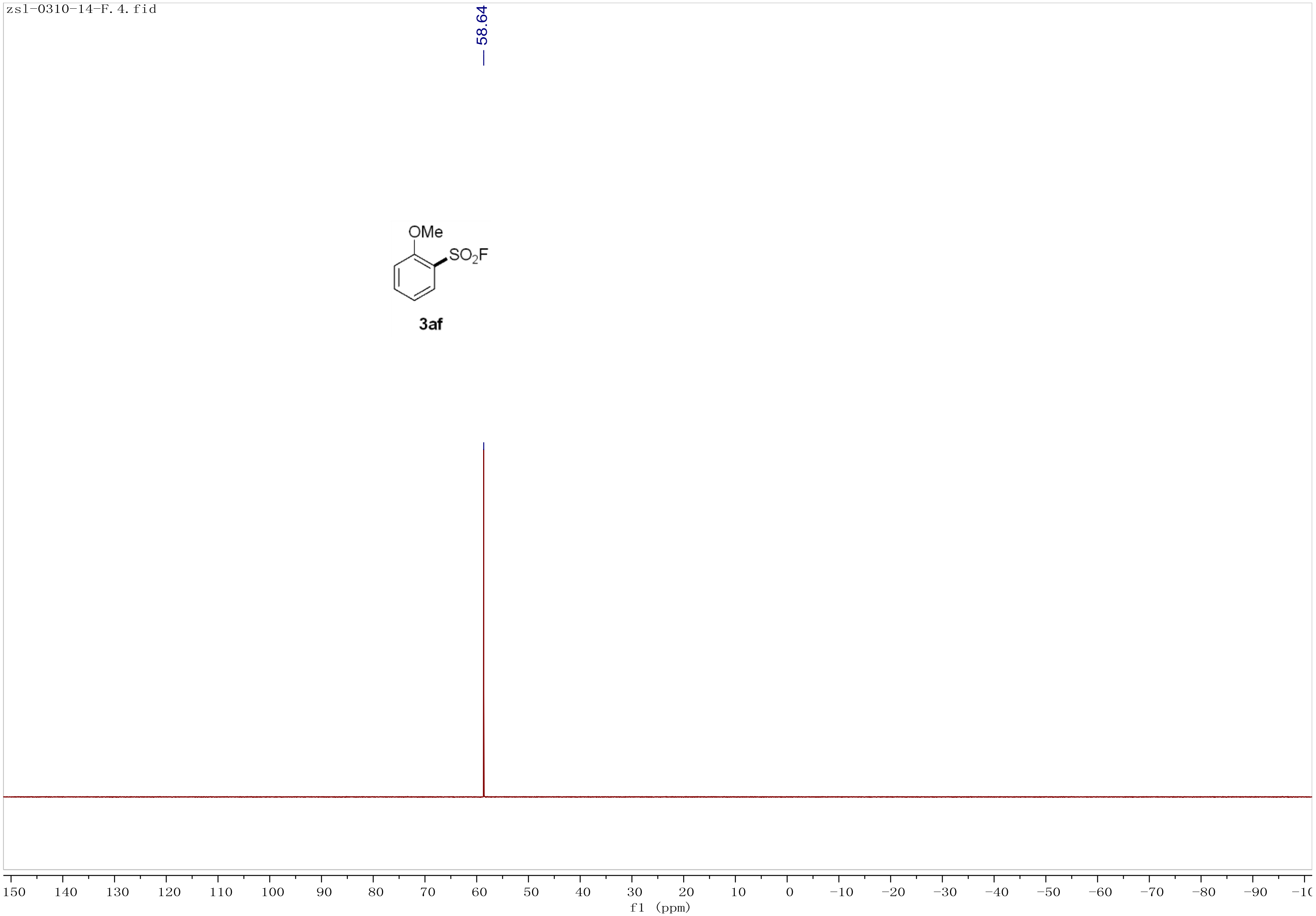

Supplementary Fig. 101 ^{19}F NMR spectrum of compound 3af ($CDCl_3$, 471 MHz, 298K)

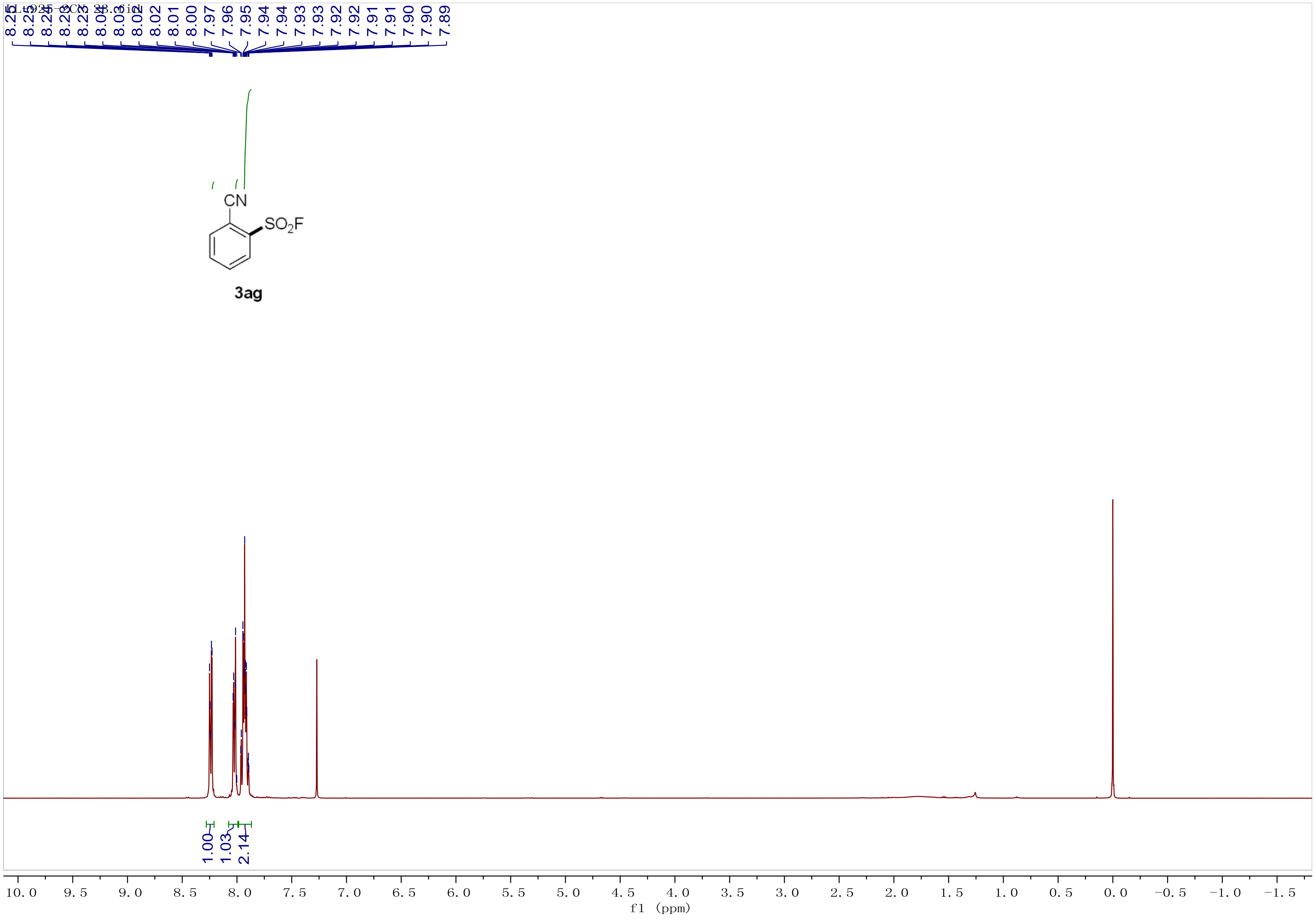

Supplementary Fig. 102 ^{1}H NMR spectrum of compound 3ag ($CDCl_3$, 400 MHz, 298K)

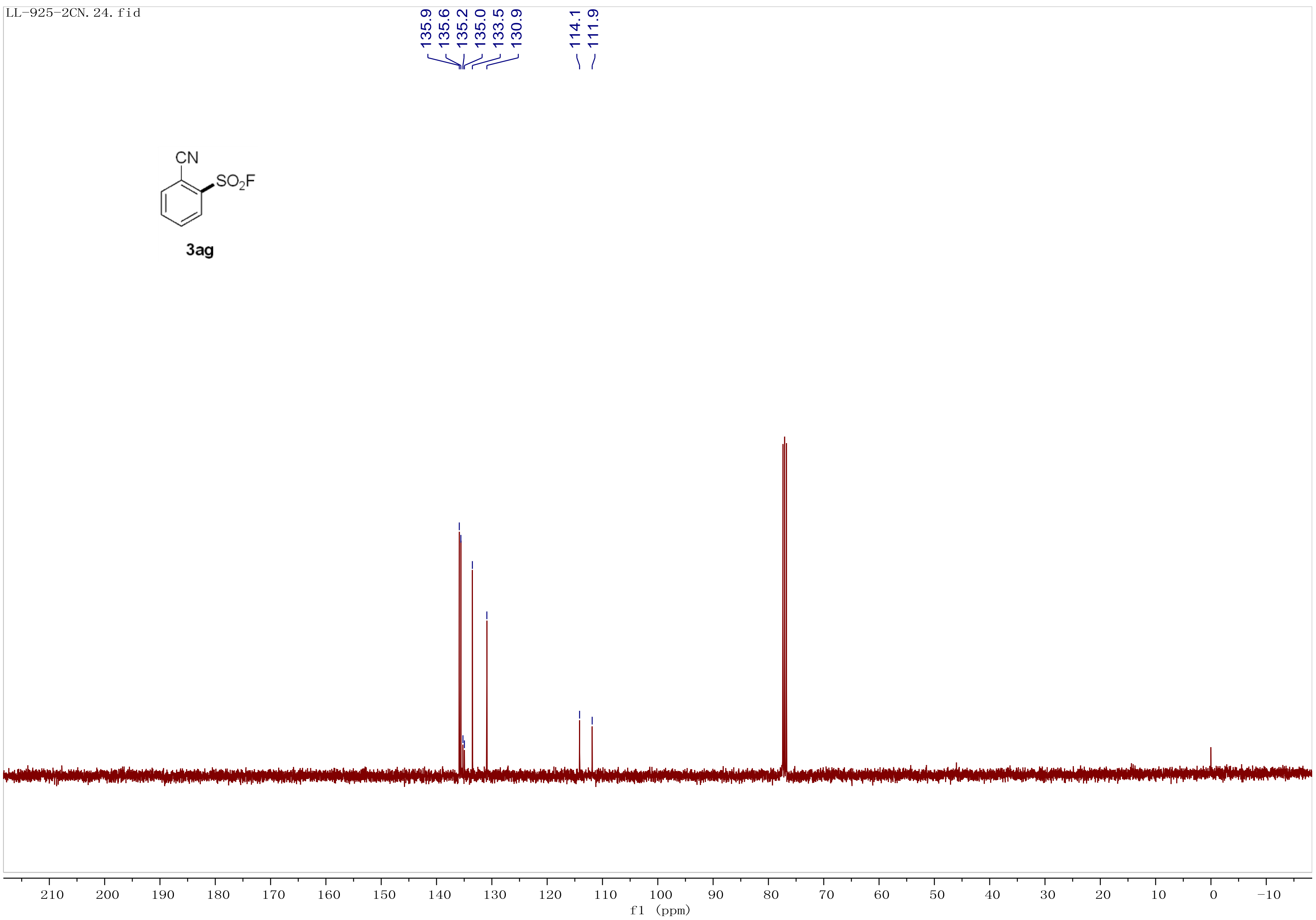

Supplementary Fig. 103 ^{13}C NMR spectrum of compound 3ag ($CDCl_3$, 101 MHz, 298K)

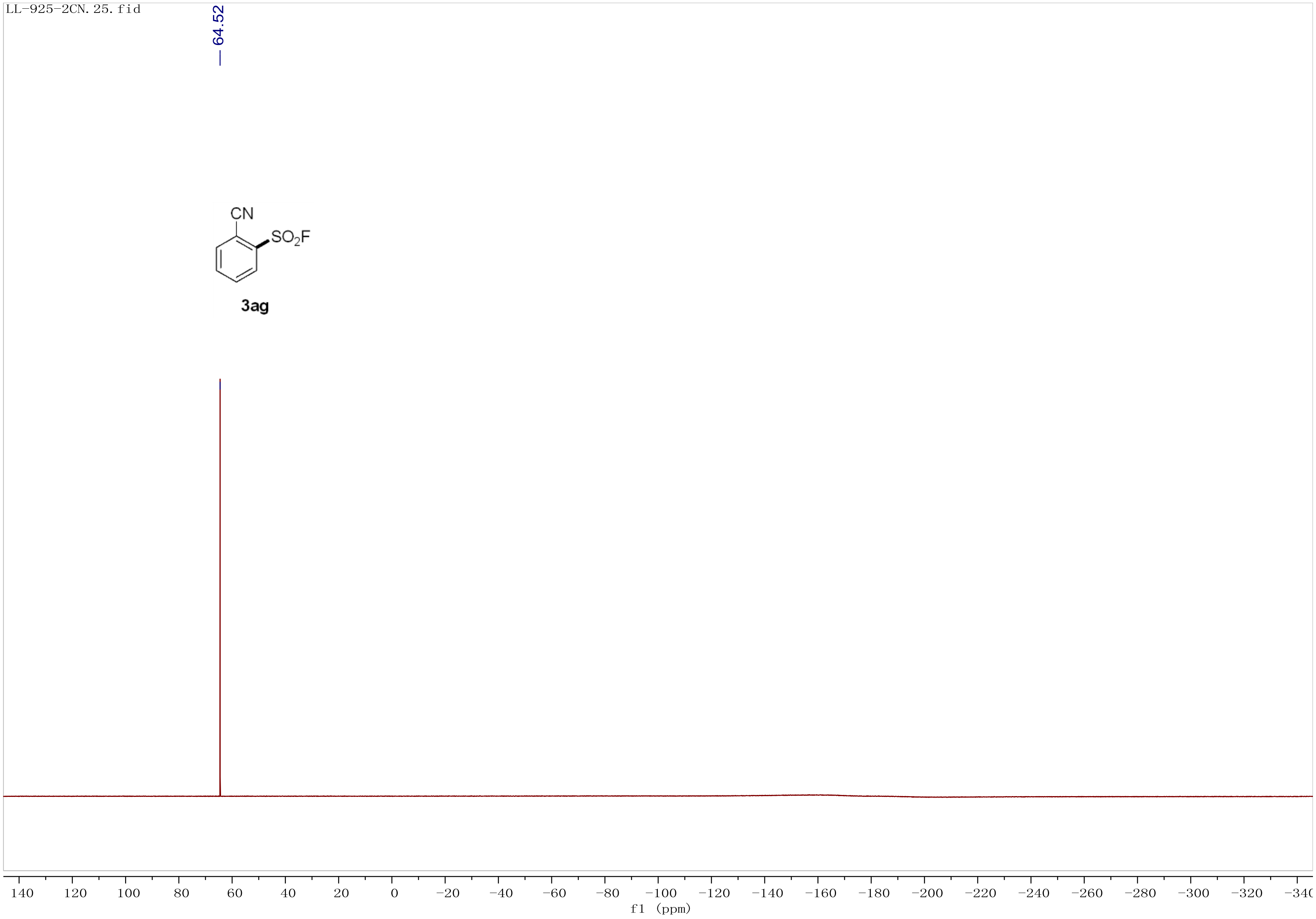

Supplementary Fig. 104 ^{19}F NMR spectrum of compound 3ag ($CDCl_3$, 376 MHz, 298K)

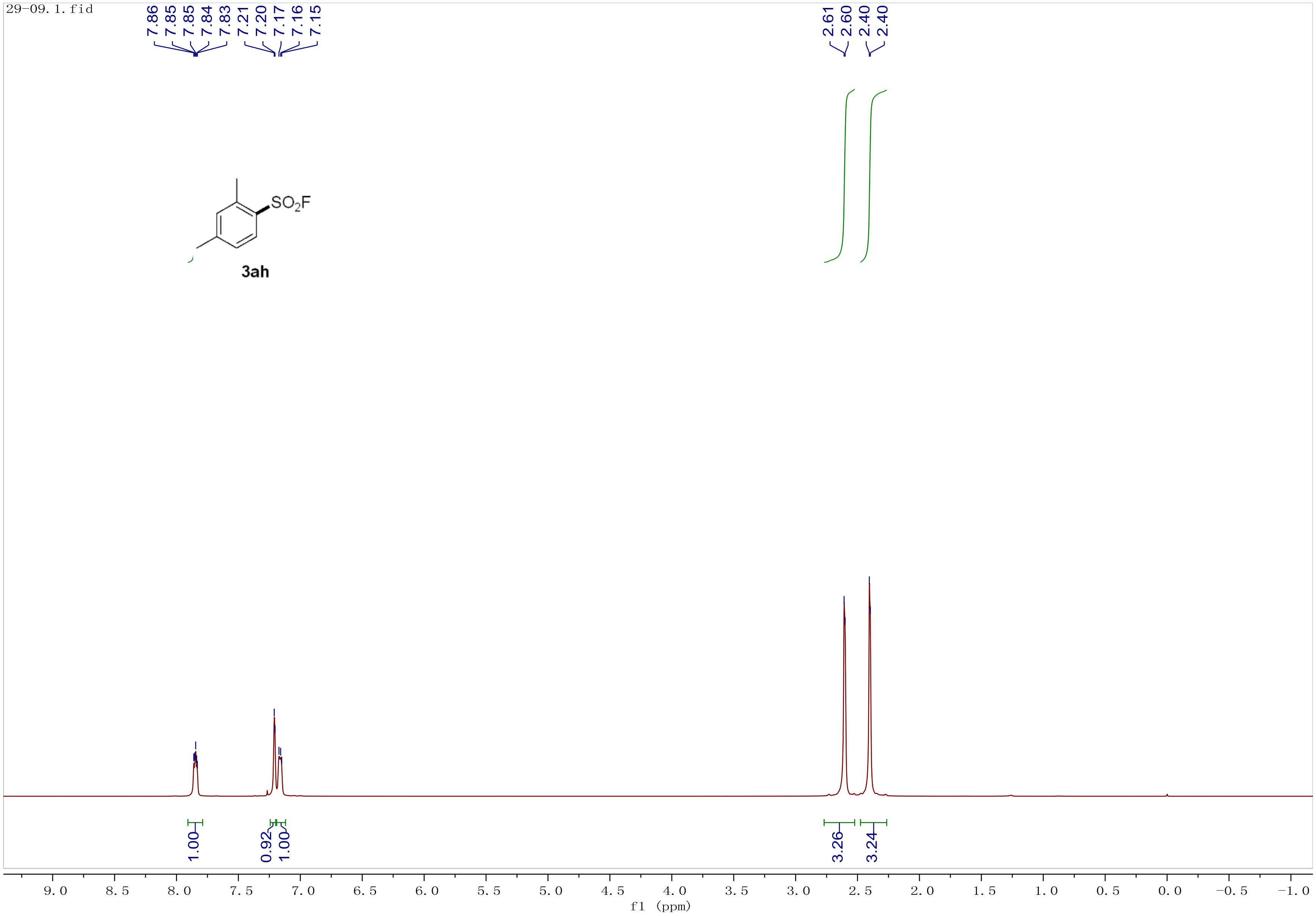

Supplementary Fig. 105 ^{1}H NMR spectrum of compound 3ah ($CDCl_3$, 500 MHz, 298K)

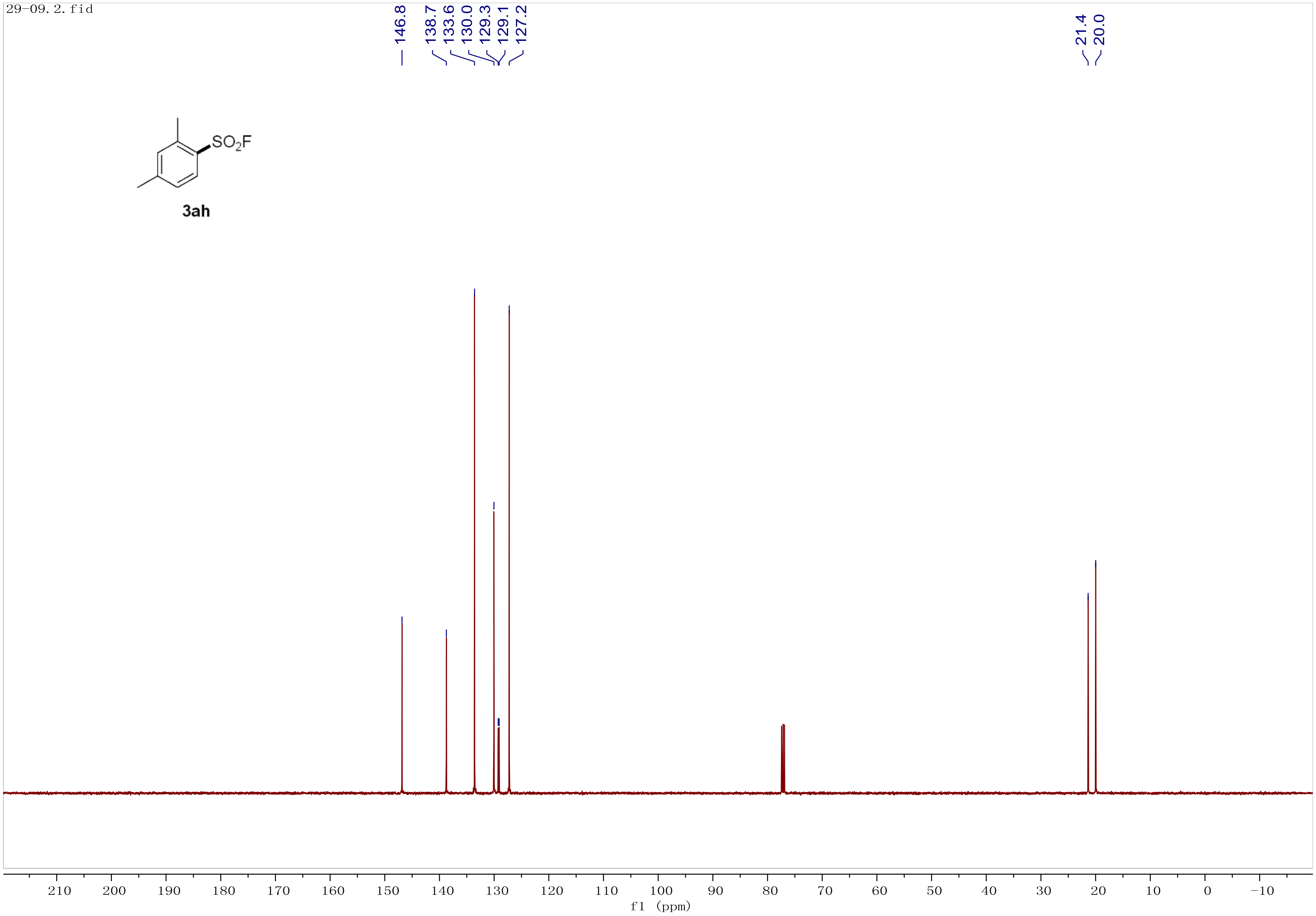

Supplementary Fig. 106 ^{13}C NMR spectrum of compound 3ah ($CDCl_3$, 126 MHz, 298K)

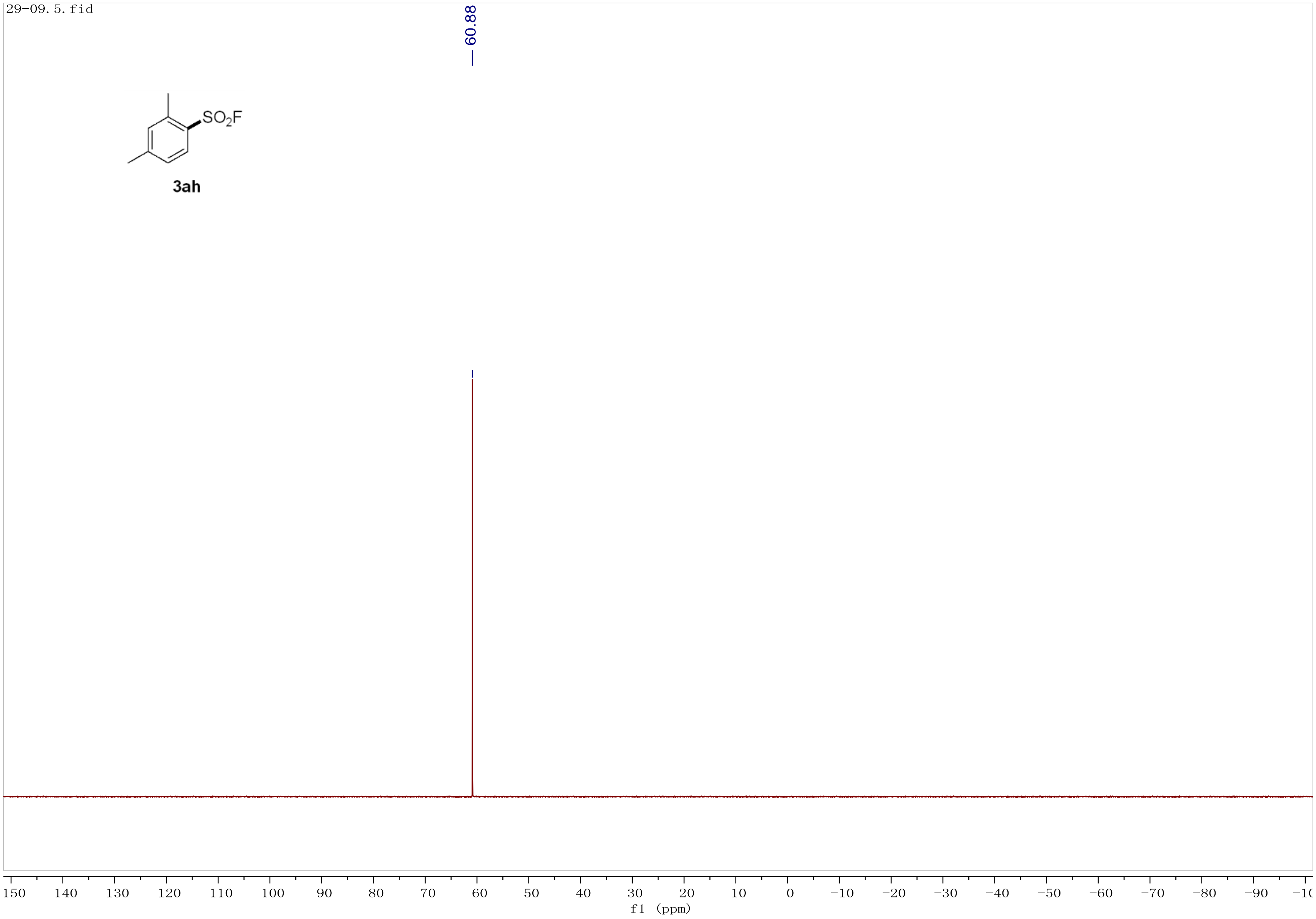

Supplementary Fig. 107 ^{19}F NMR spectrum of compound 3ah ($CDCl_3$, 471 MHz, 298K)

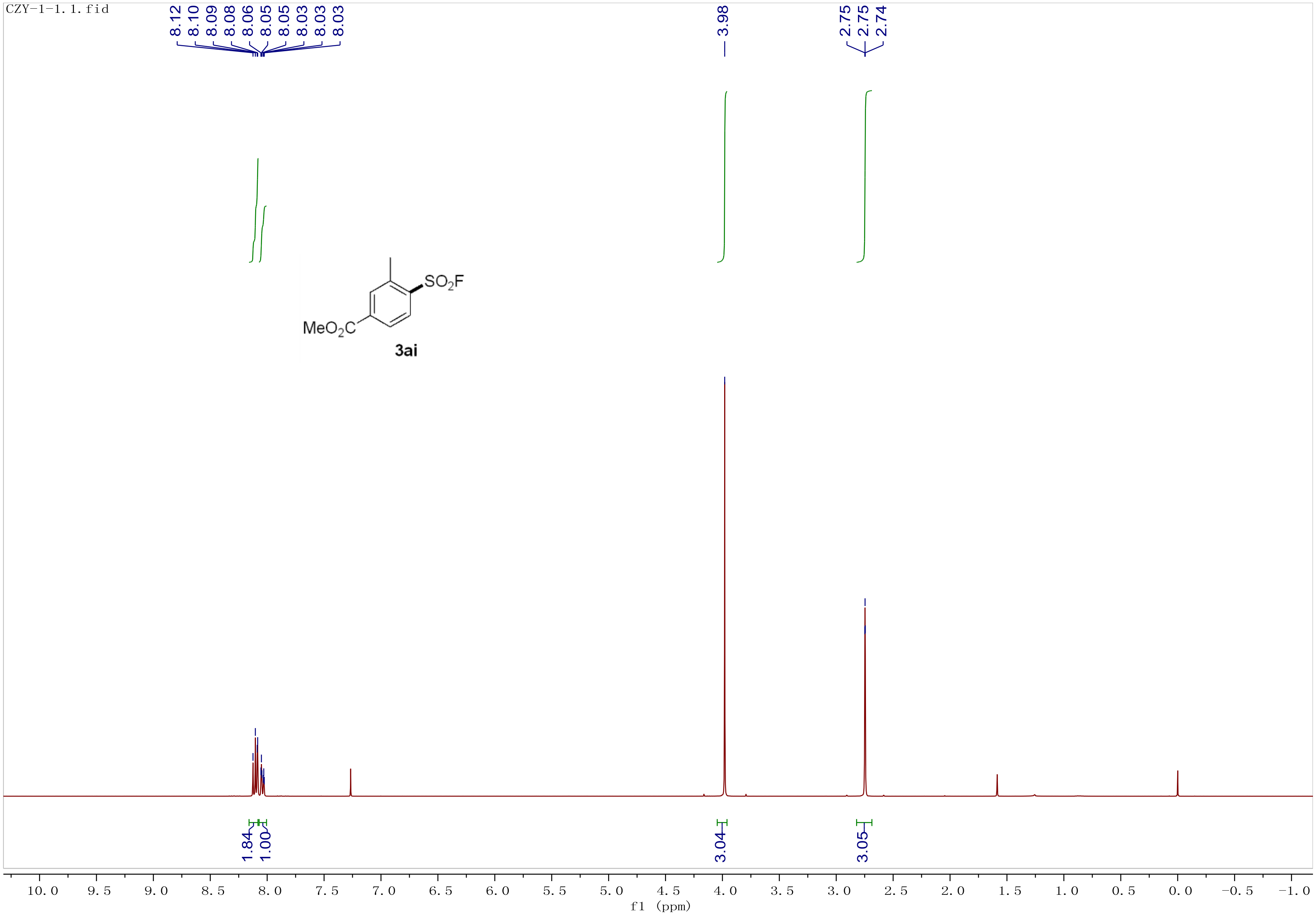

Supplementary Fig. 108 ^{1}H NMR spectrum of compound 3ai ($CDCl_3$, 400 MHz, 298K)

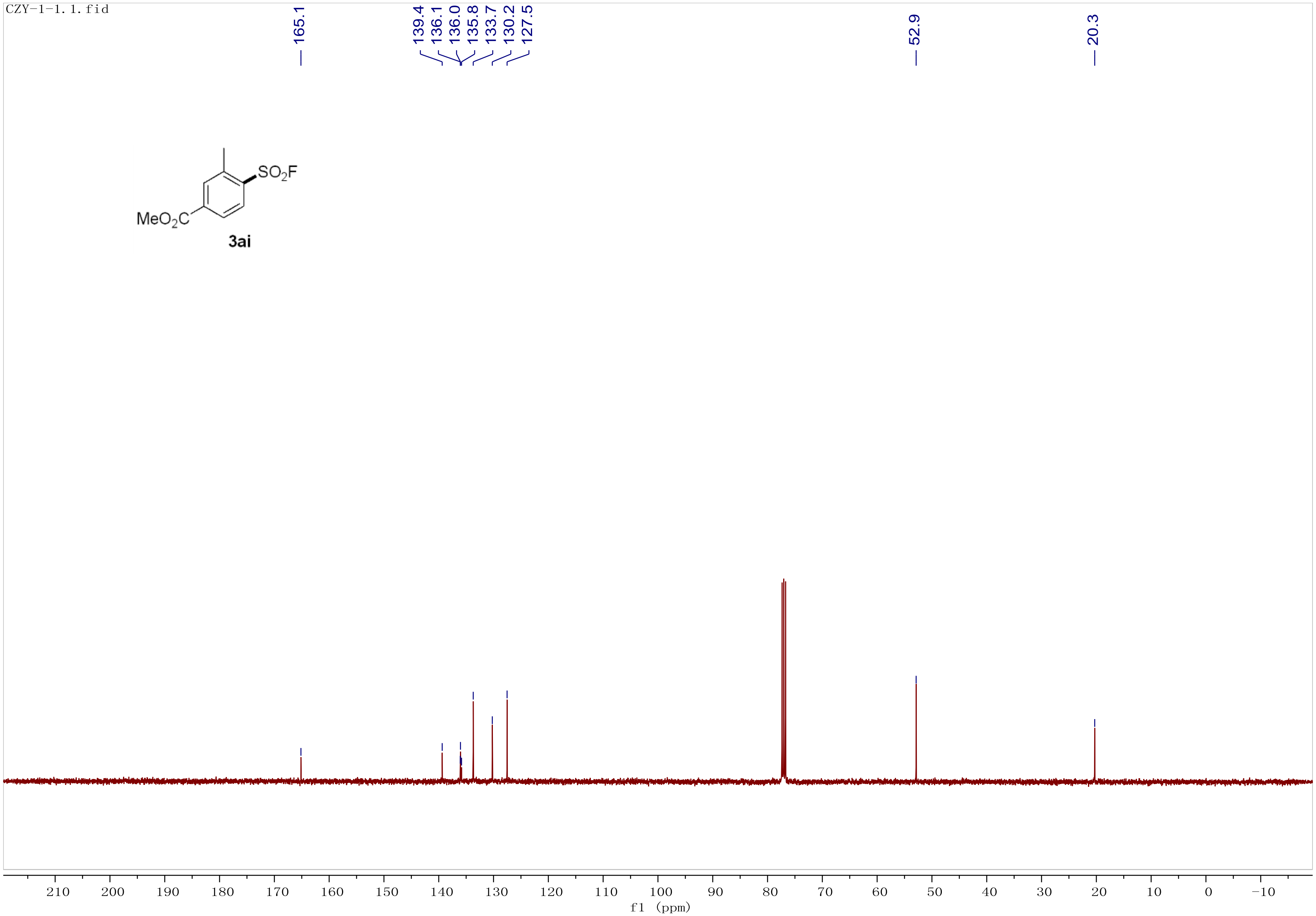

Supplementary Fig. 109 ^{13}C NMR spectrum of compound 3ai ($CDCl_3$, 101 MHz, 298K)

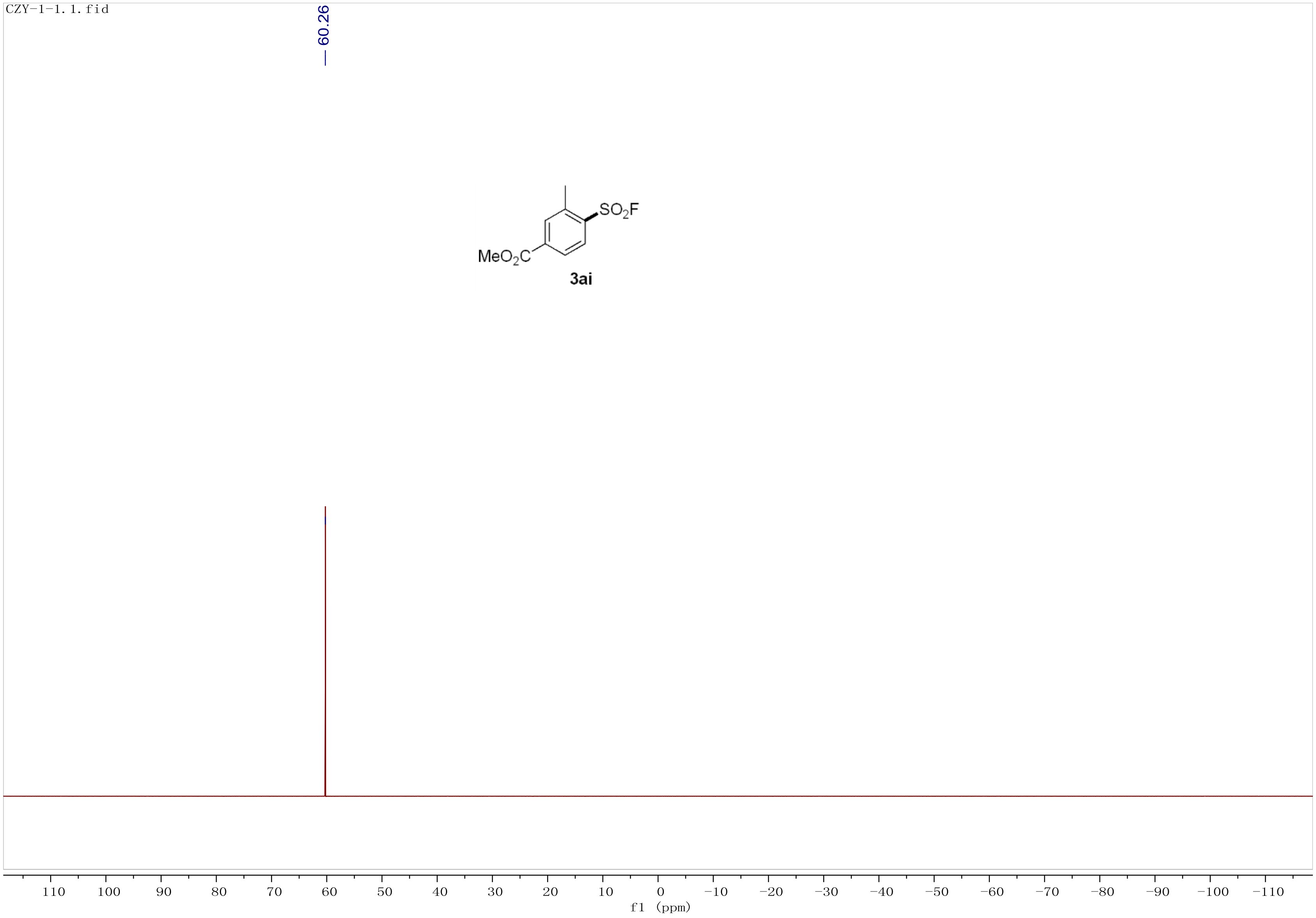

Supplementary Fig. 110 ^{19}F NMR spectrum of compound 3ai ($CDCl_3$, 376 MHz, 298K)

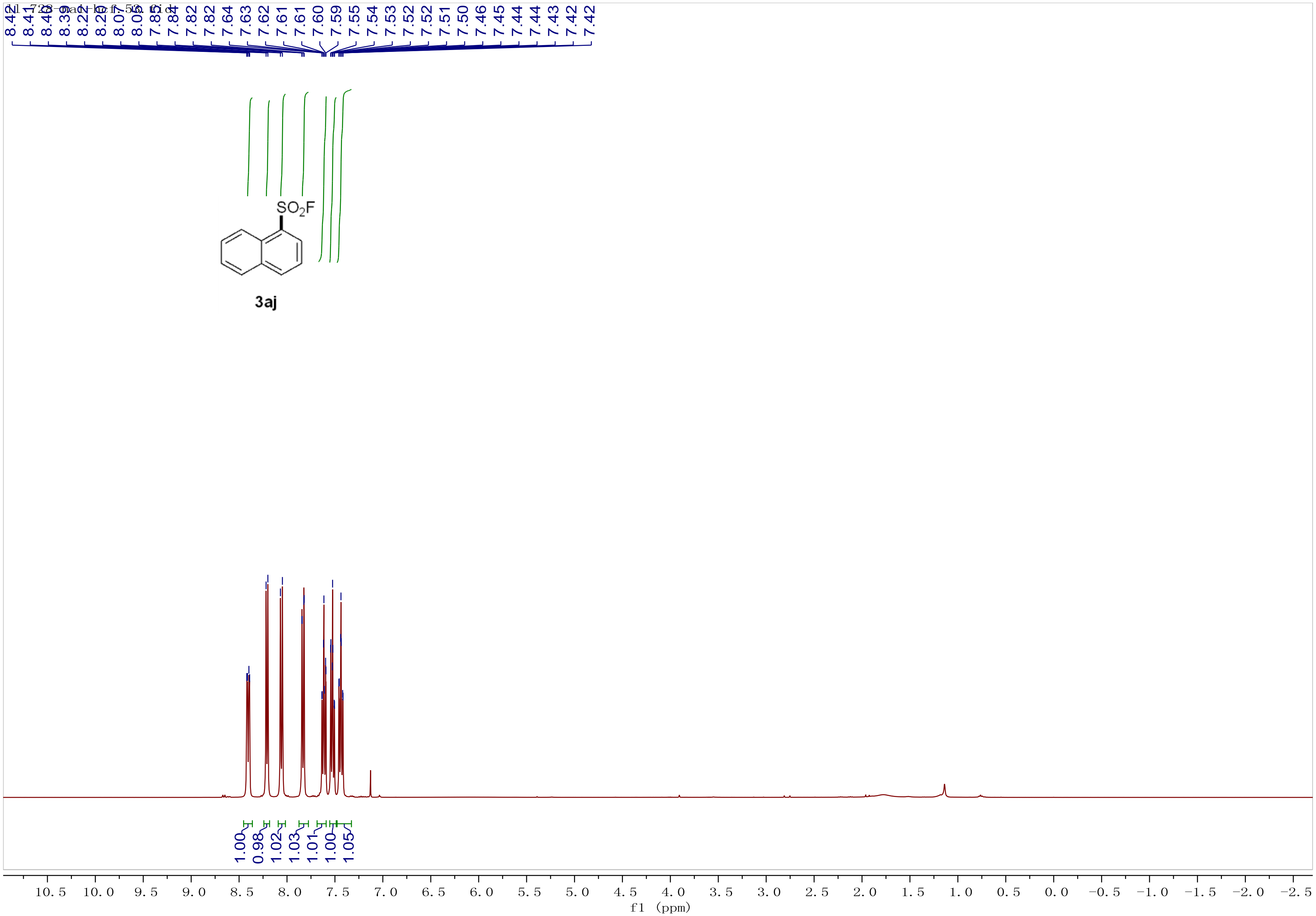

Supplementary Fig. 111 ^{1}H NMR spectrum of compound 3aj ($CDCl_3$, 400 MHz, 298K)

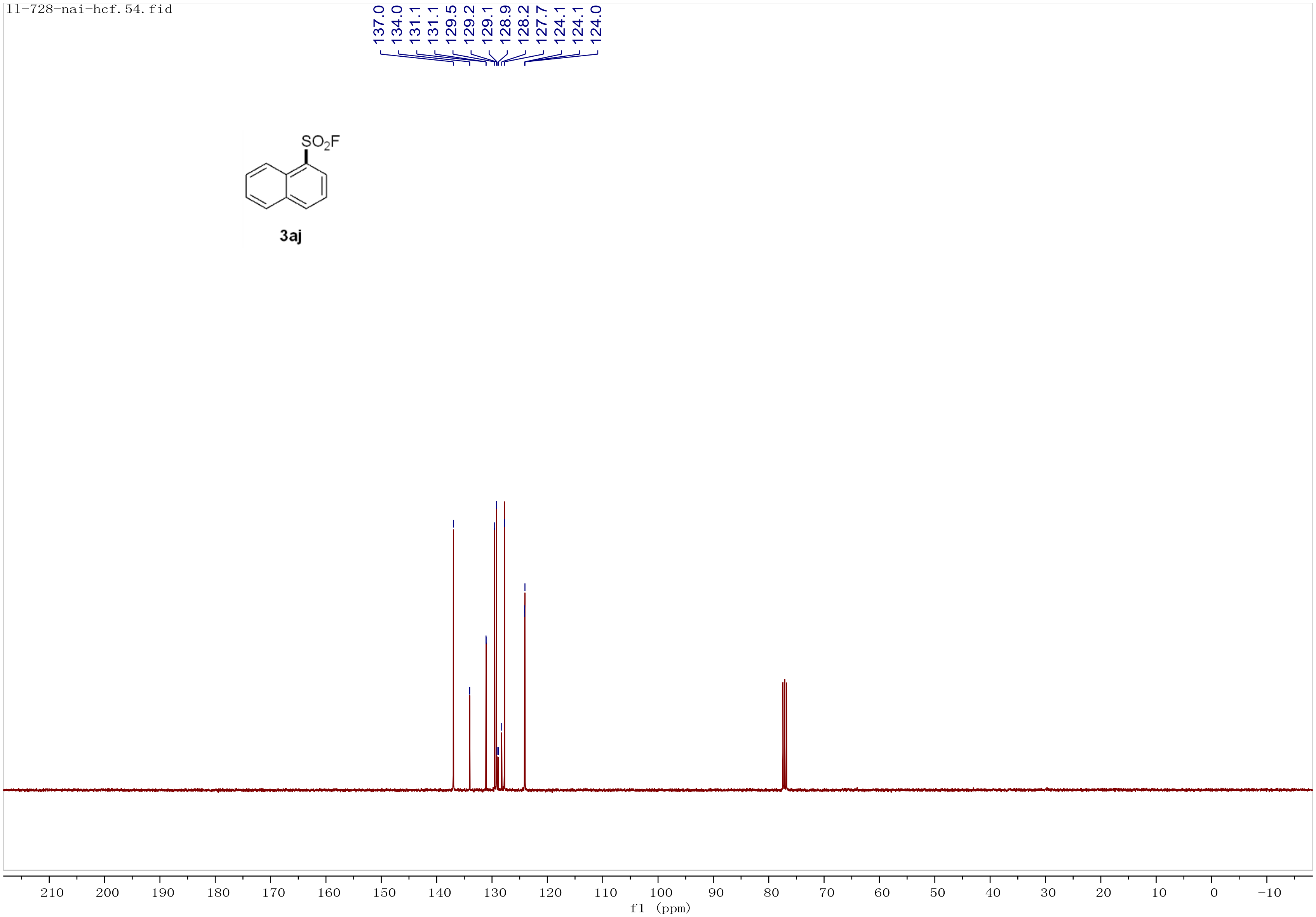

Supplementary Fig. 112 ^{13}C NMR spectrum of compound 3aj ($CDCl_3$, 101 MHz, 298K)

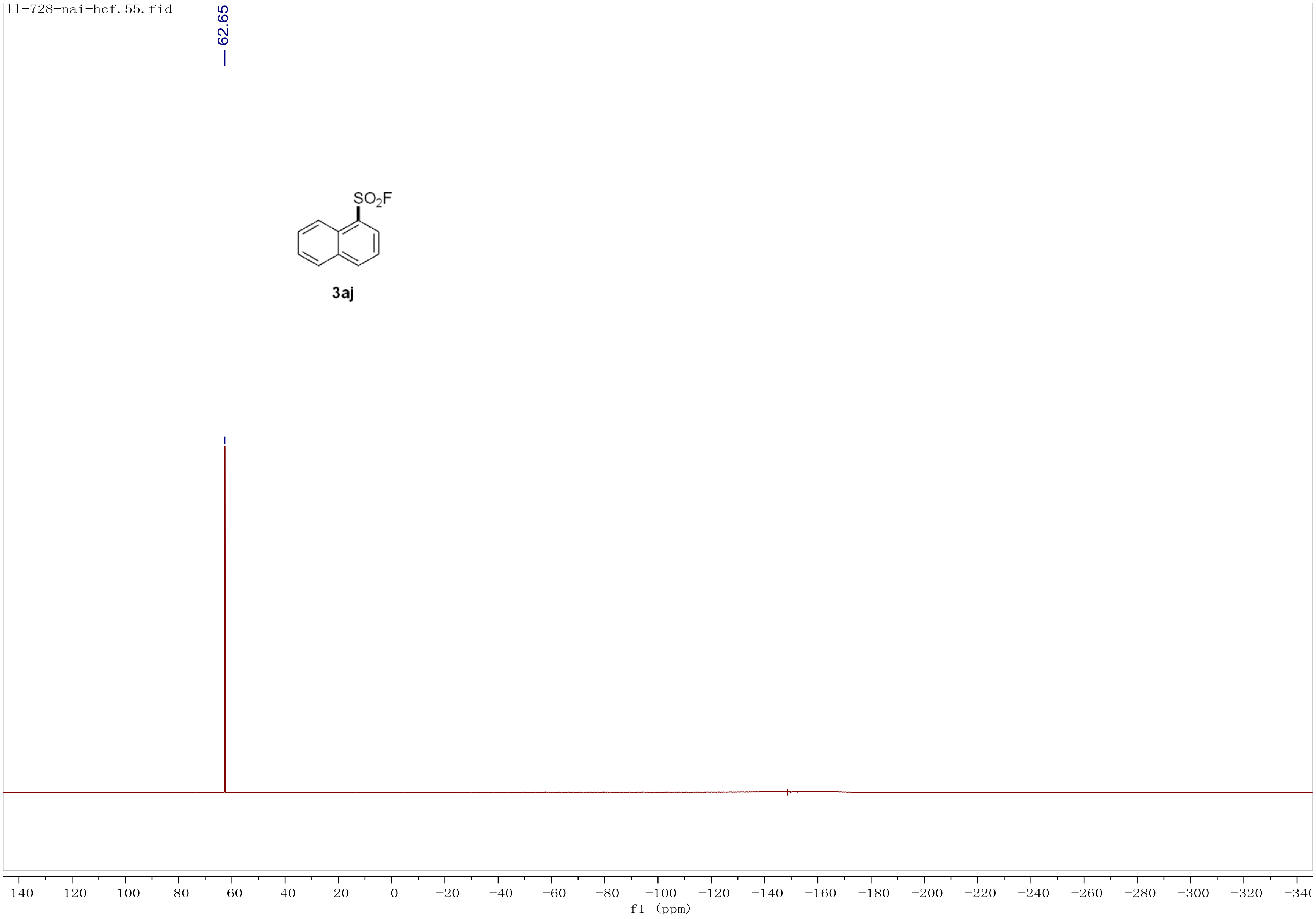

Supplementary Fig. 113 ^{19}F NMR spectrum of compound 3aj ($CDCl_3$, 376 MHz, 298K)

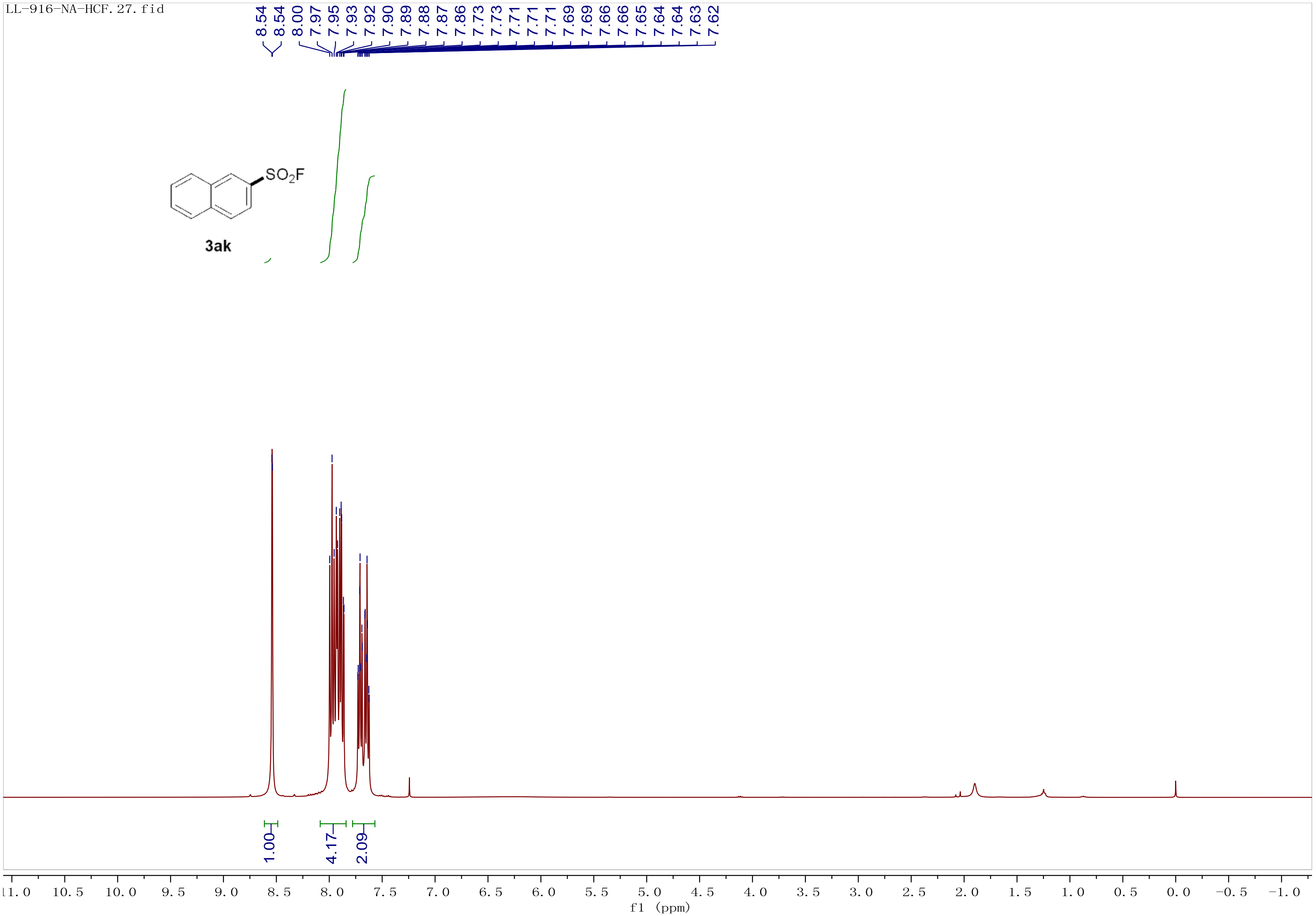

Supplementary Fig. 114 ^{1}H NMR spectrum of compound 3ak ($CDCl_3$, 400 MHz, 298K)

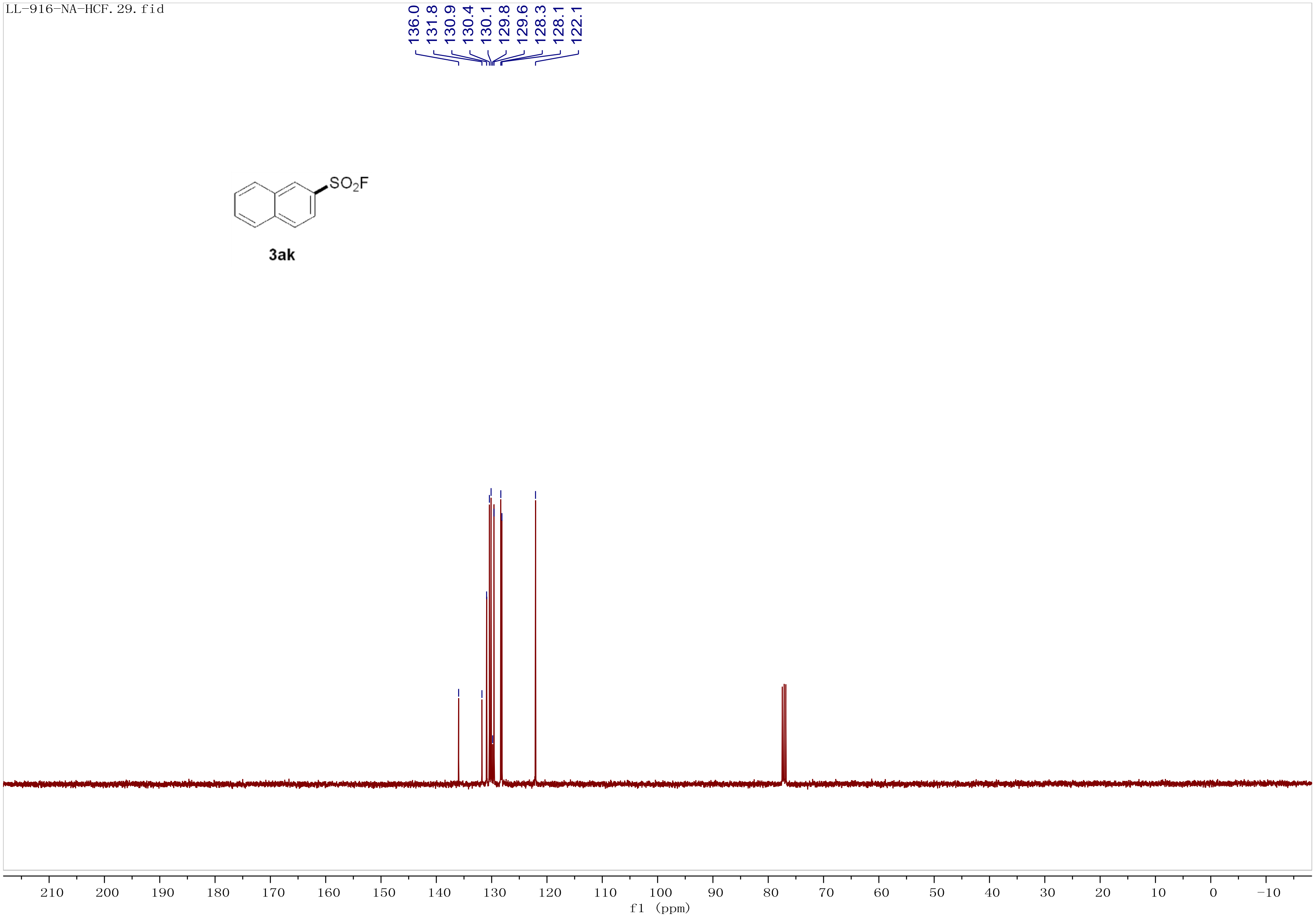

Supplementary Fig. 115 ^{13}C NMR spectrum of compound 3ak ($CDCl_3$, 101 MHz, 298K)

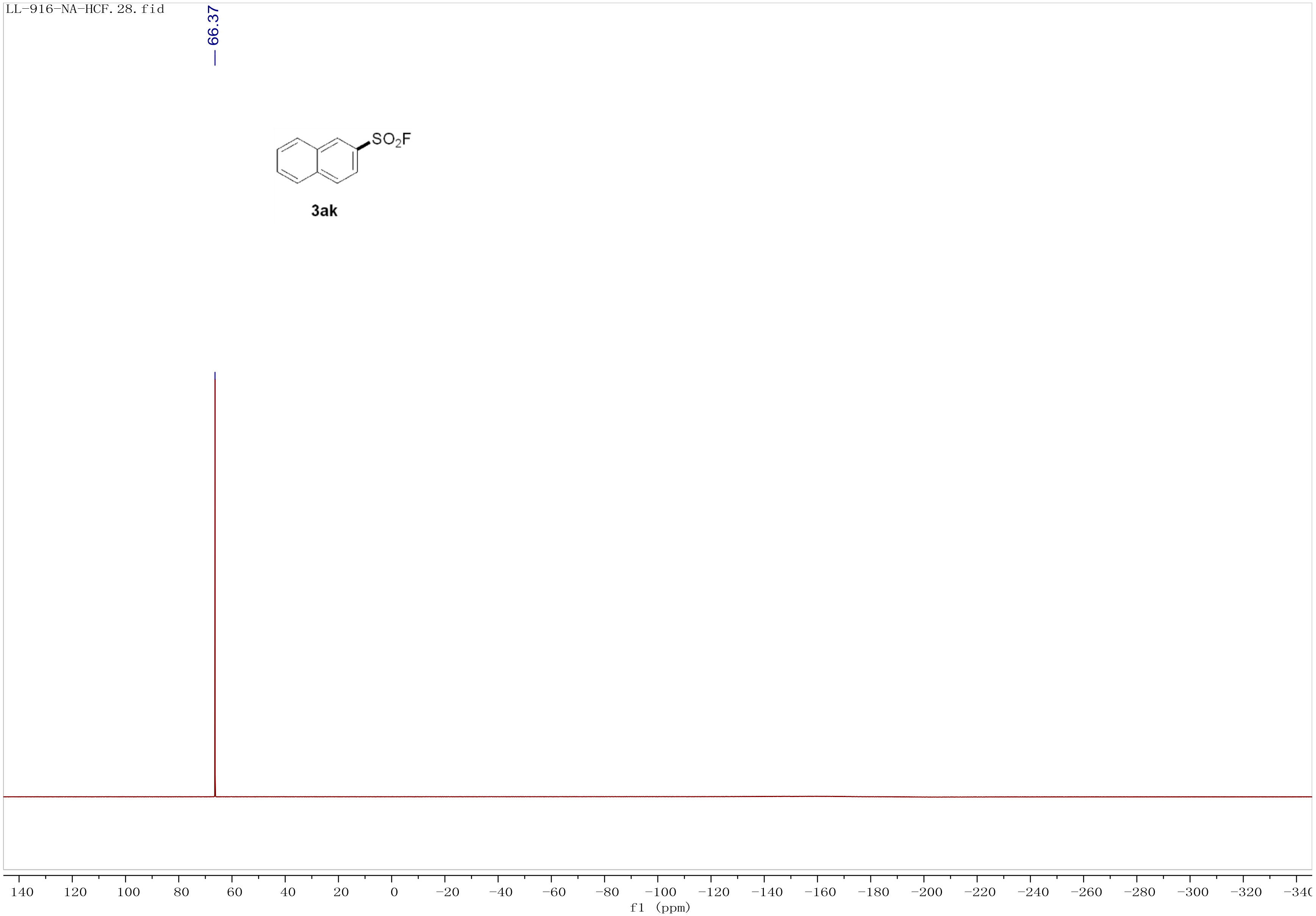

Supplementary Fig. 116 ^{19}F NMR spectrum of compound 3ak ($CDCl_3$, 376 MHz, 298K)

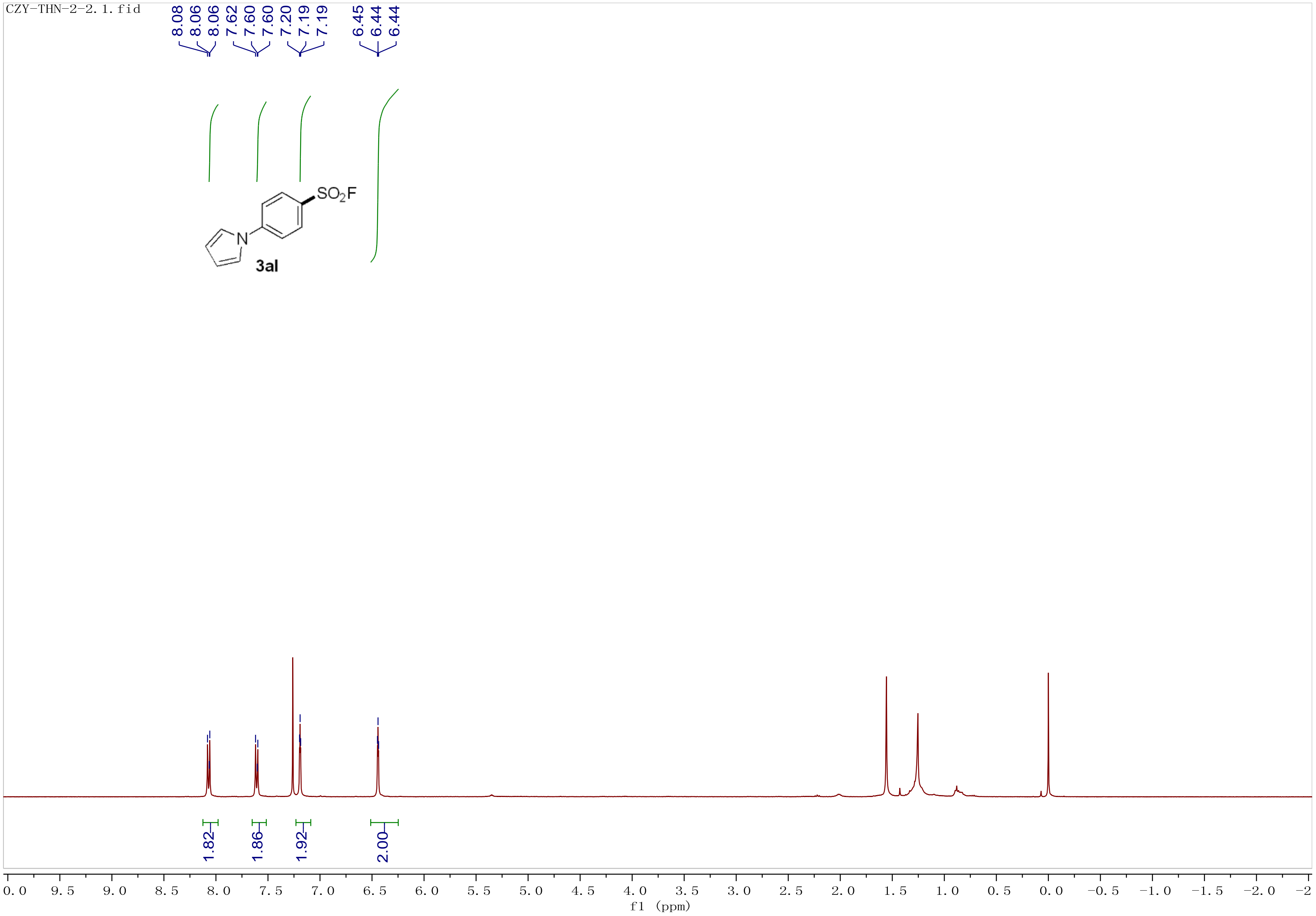

Supplementary Fig. 117 ^{1}H NMR spectrum of compound 3al ($CDCl_3$, 400 MHz, 298K)

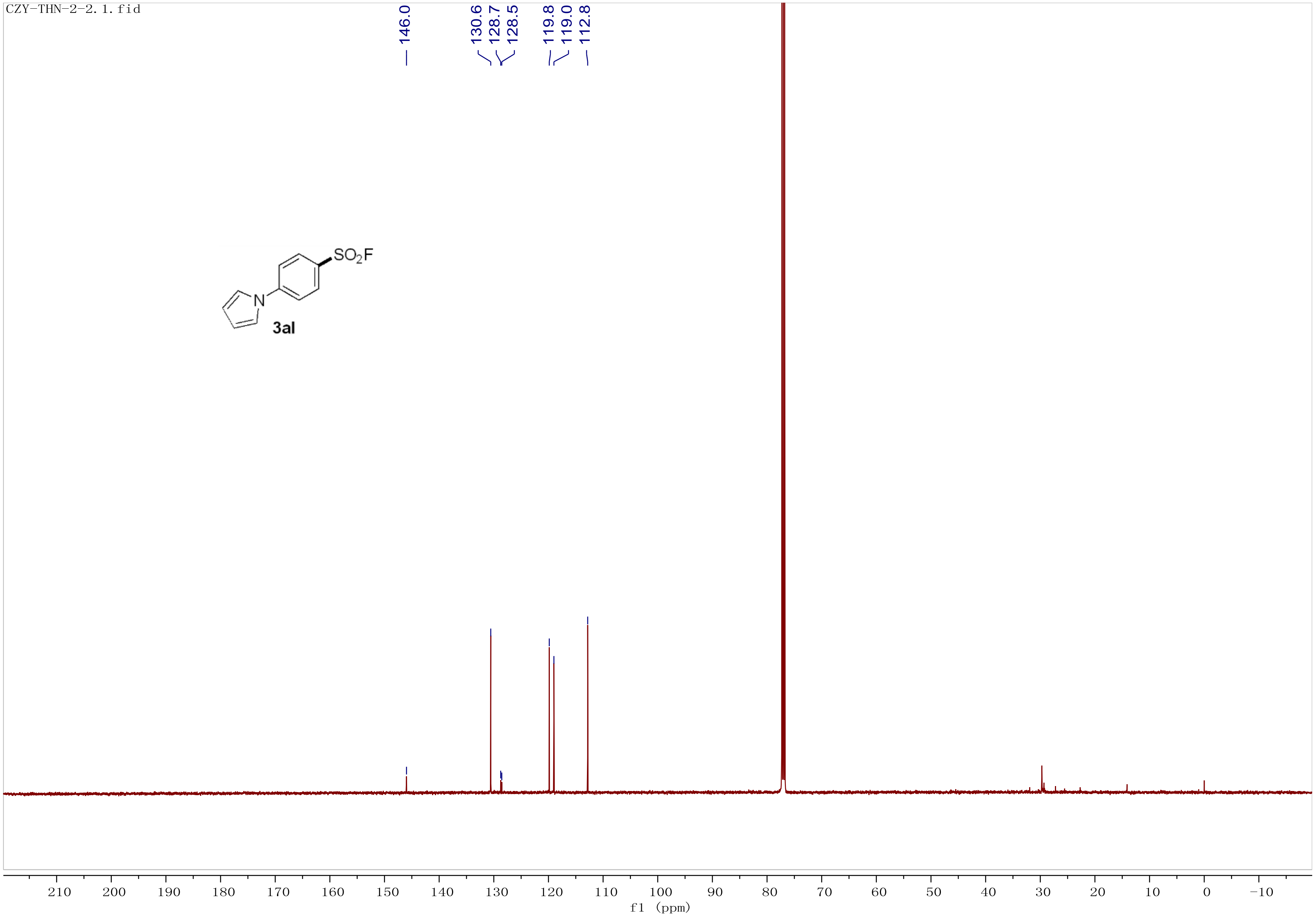

Supplementary Fig. 118 ^{13}C NMR spectrum of compound 3al ($CDCl_3$, 126 MHz, 298K)

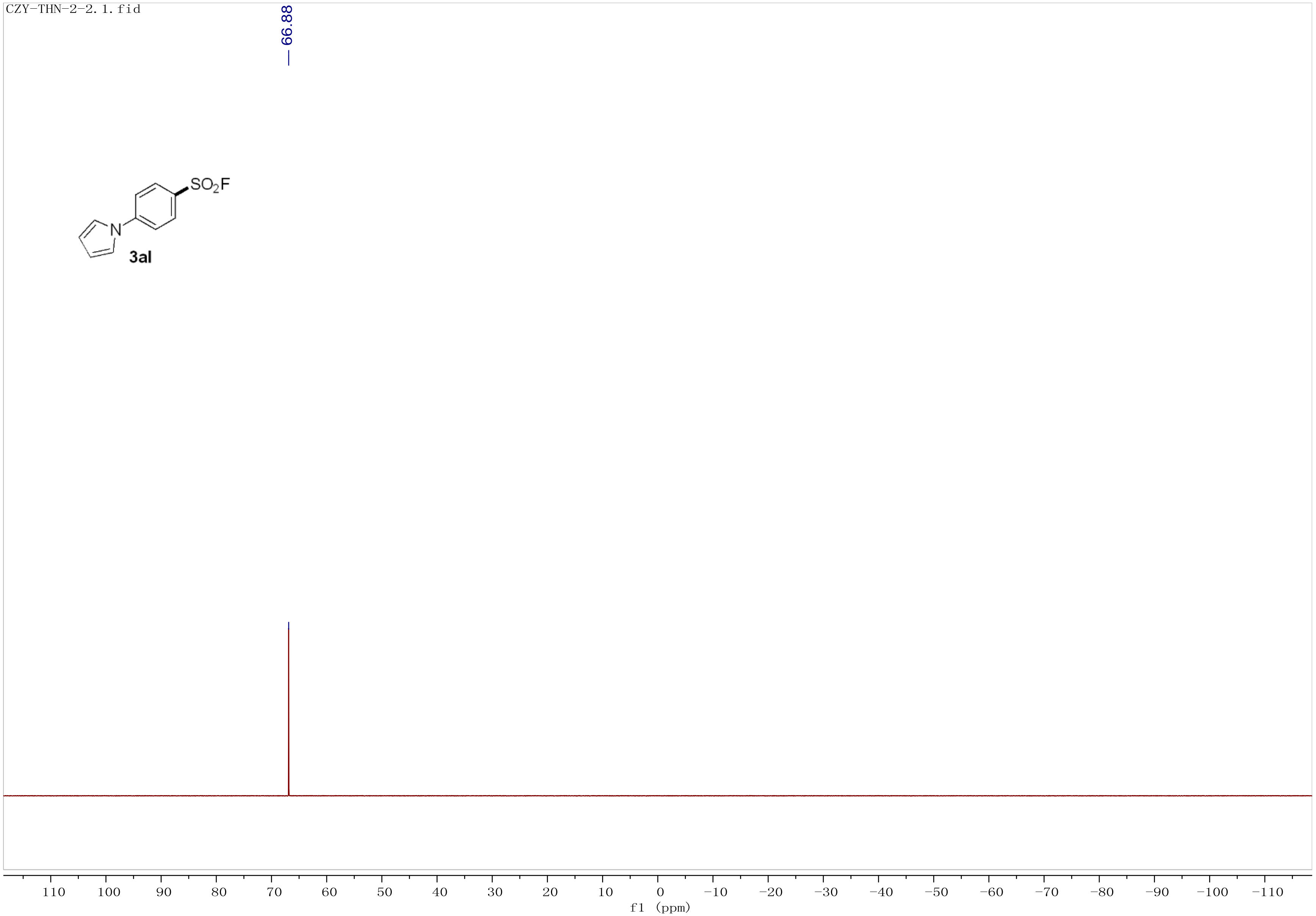

Supplementary Fig. 119 ^{19}F NMR spectrum of compound 3al ($CDCl_3$, 376 MHz, 298K)

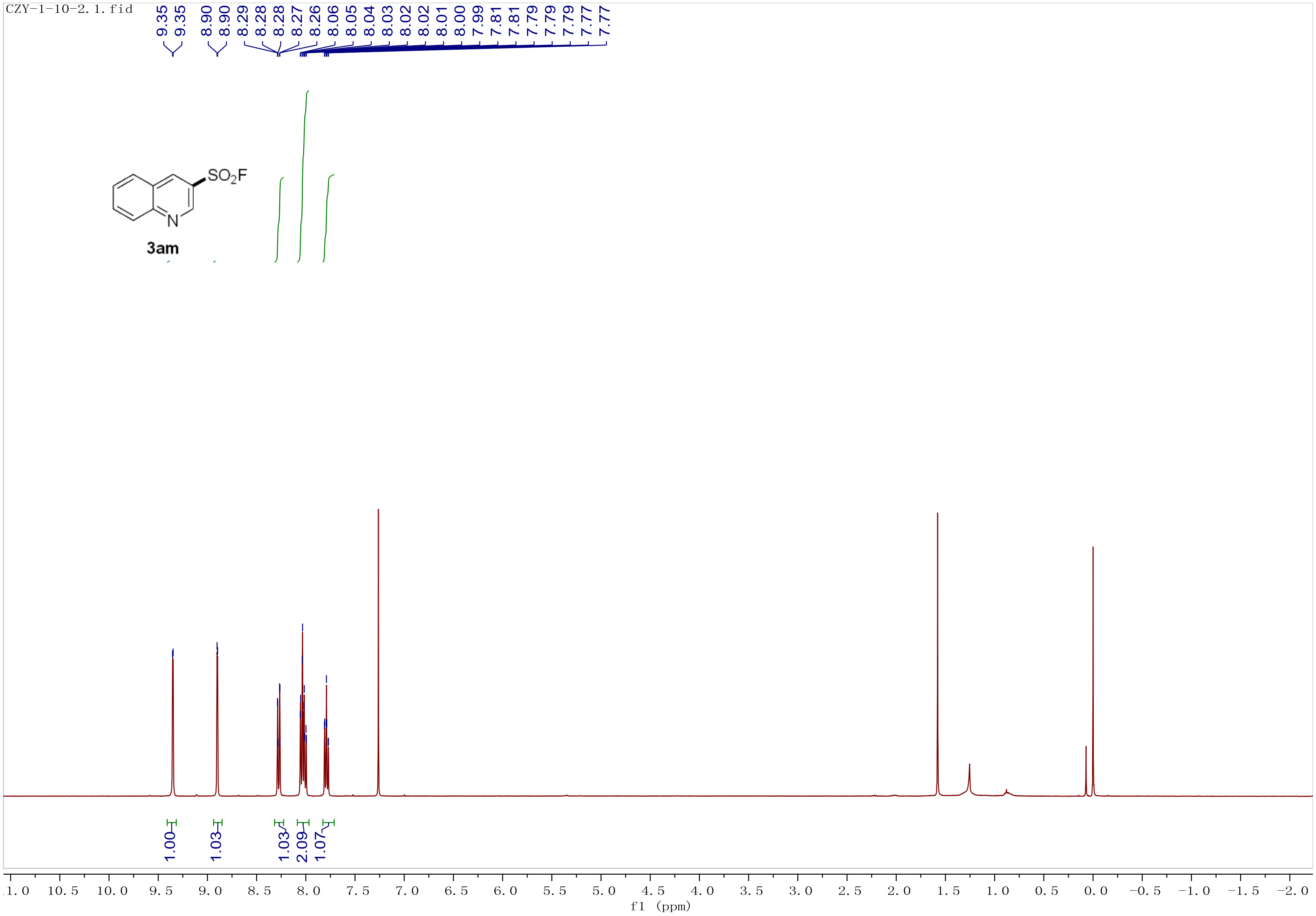

Supplementary Fig. 120 ^{1}H NMR spectrum of compound 3am ($CDCl_3$, 400 MHz, 298K)

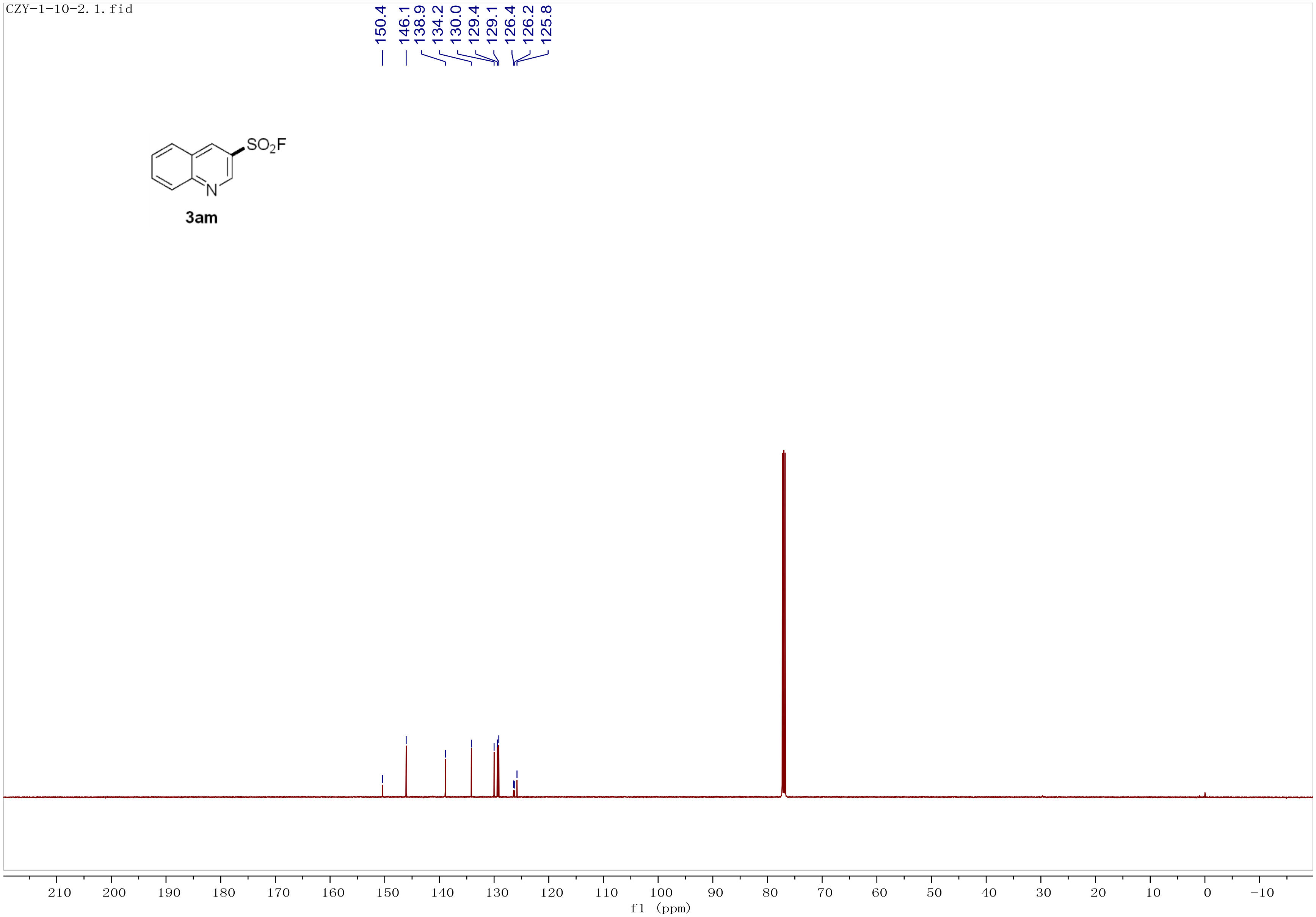

Supplementary Fig. 121 ^{13}C NMR spectrum of compound 3am ($CDCl_3$, 101 MHz, 298K)

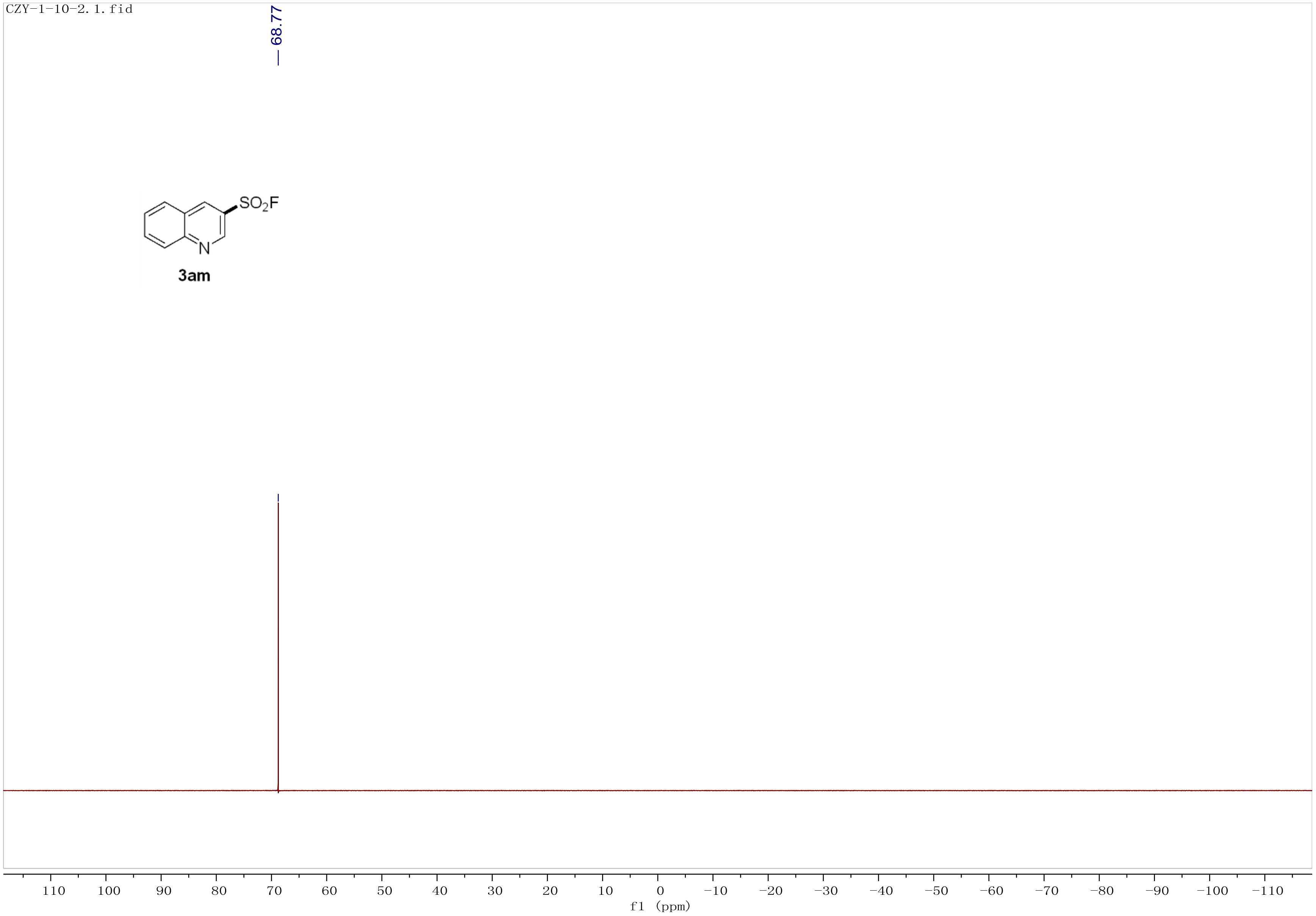

Supplementary Fig. 122 ^{19}F NMR spectrum of compound 3am ($CDCl_3$, 376 MHz, 298K)

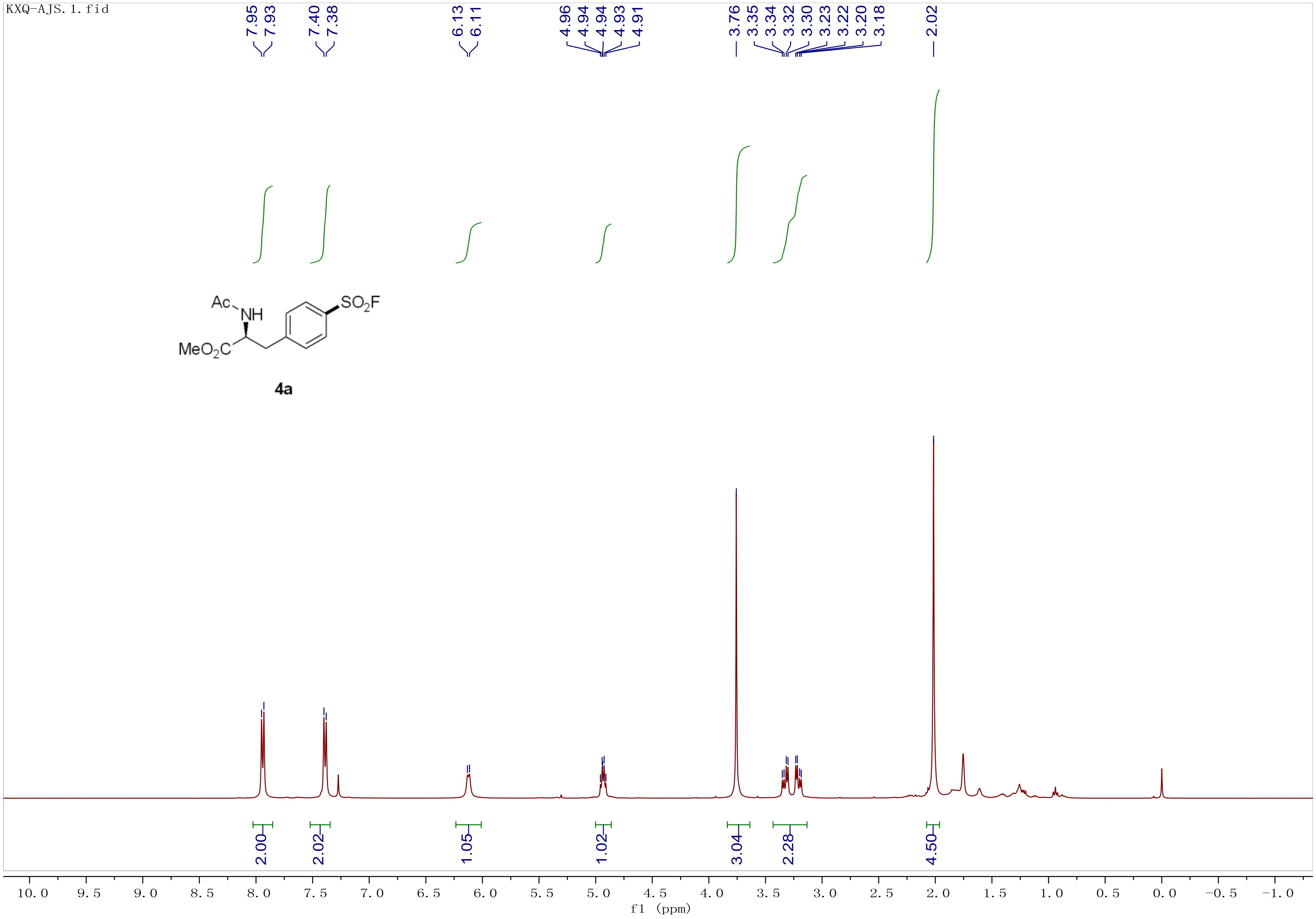

Supplementary Fig. 123 ^{1}H NMR spectrum of compound 4a ($CDCl_3$, 400 MHz, 298K)

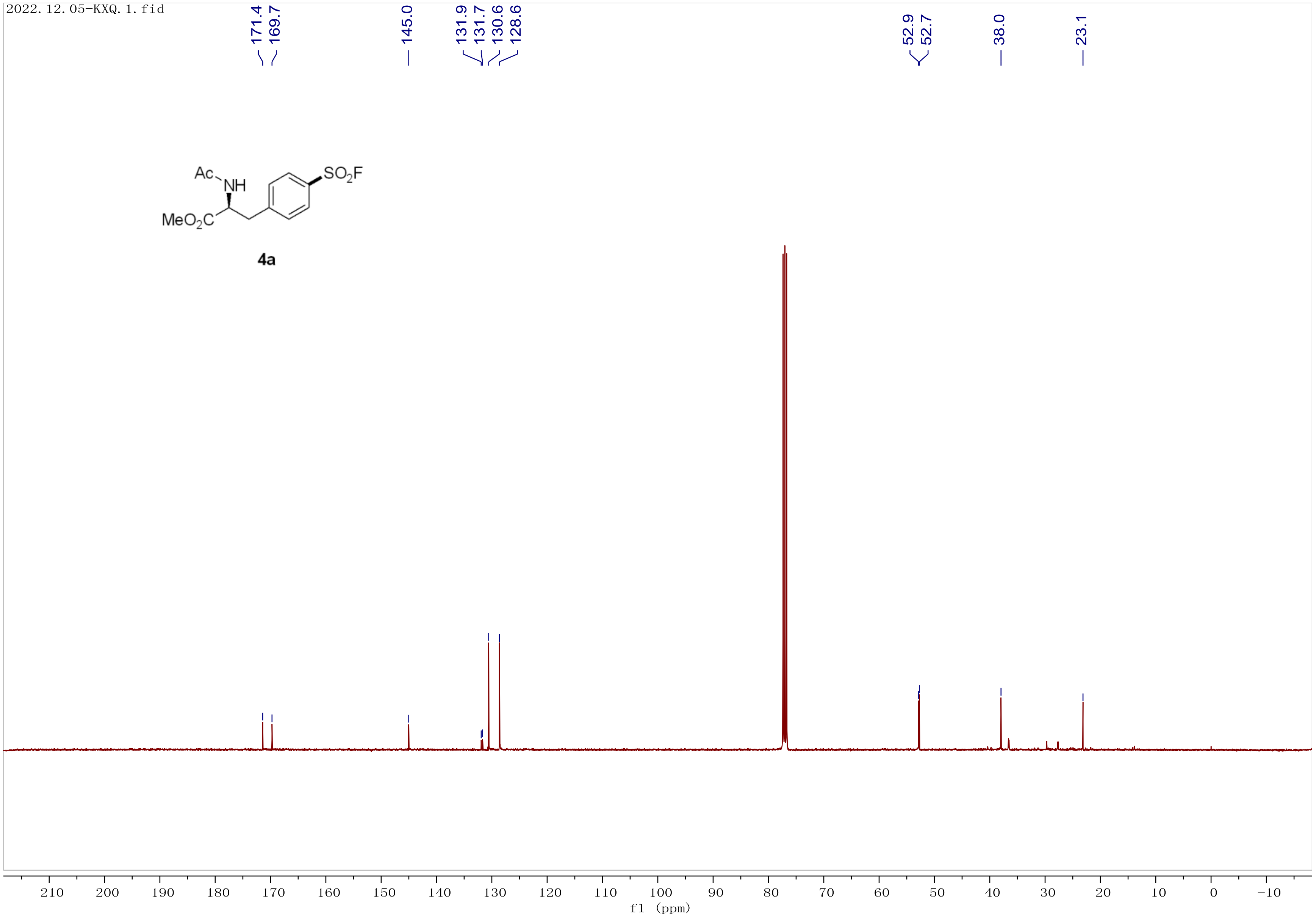

Supplementary Fig. 124 ^{13}C NMR spectrum of compound 4a ($CDCl_3$, 101 MHz, 298K)

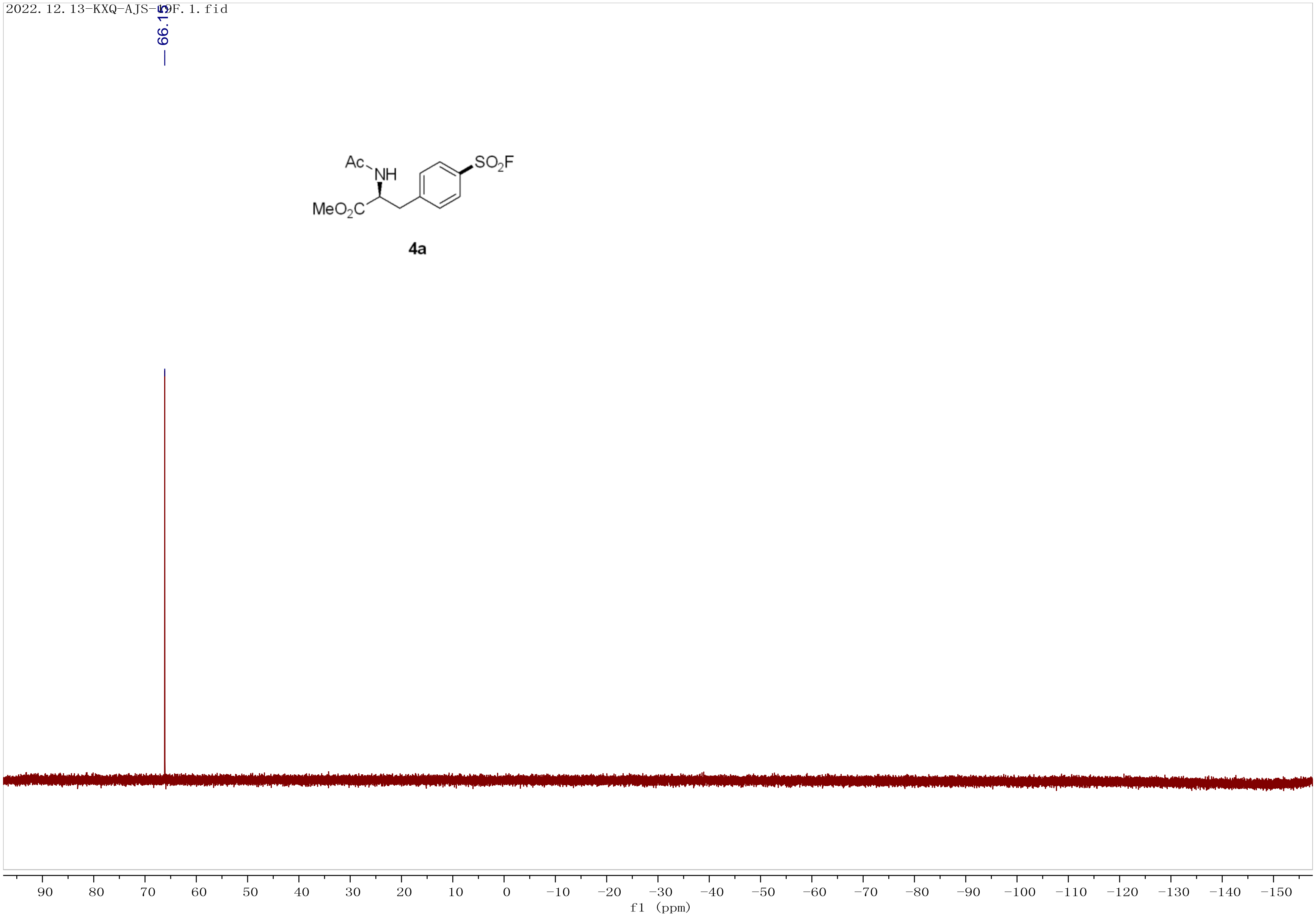

Supplementary Fig. 125 ^{19}F NMR spectrum of compound 4a ($CDCl_3$, 376 MHz, 298K)

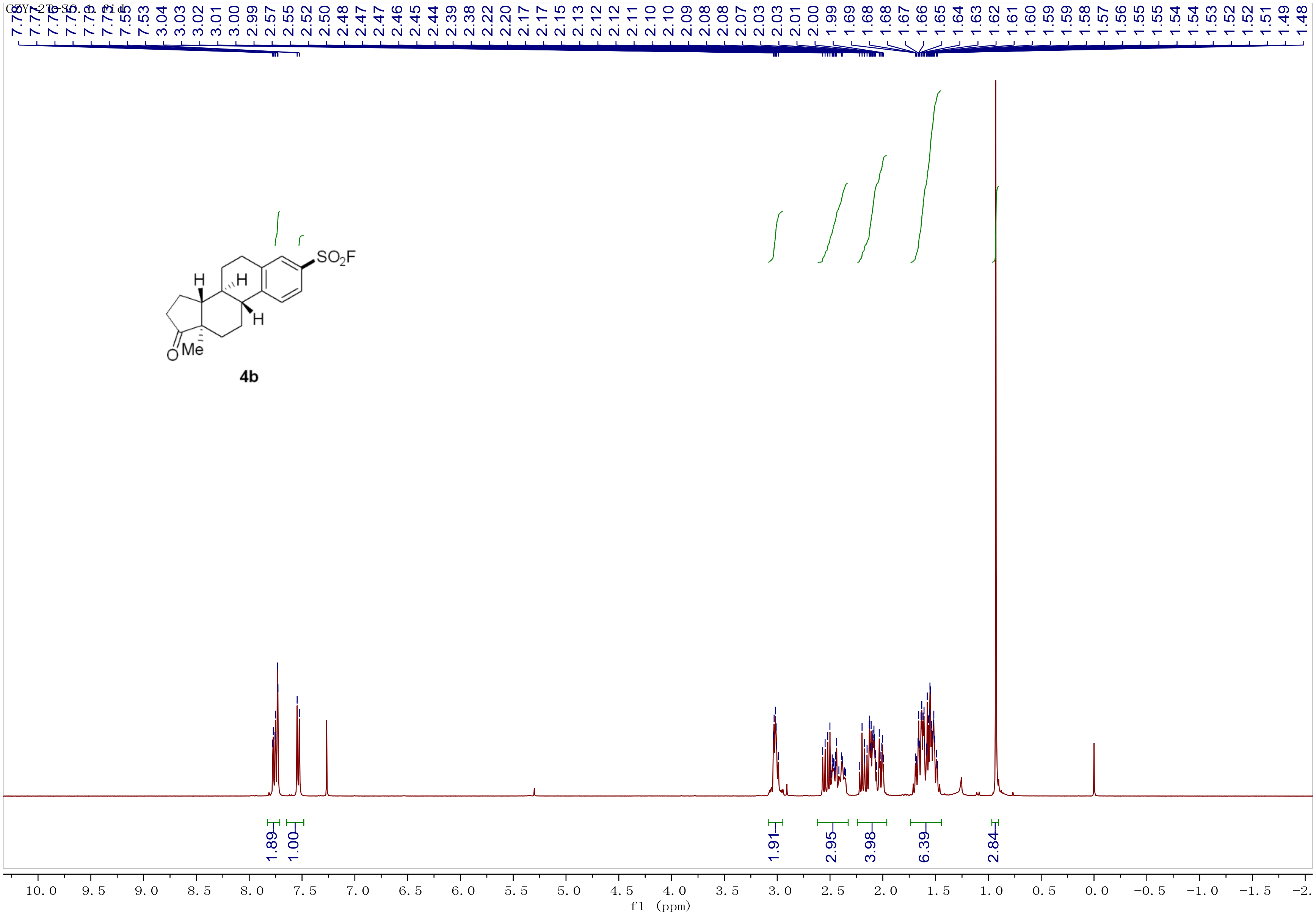

Supplementary Fig. 126 ^{1}H NMR spectrum of compound 4b ($CDCl_3$, 400 MHz, 298K)

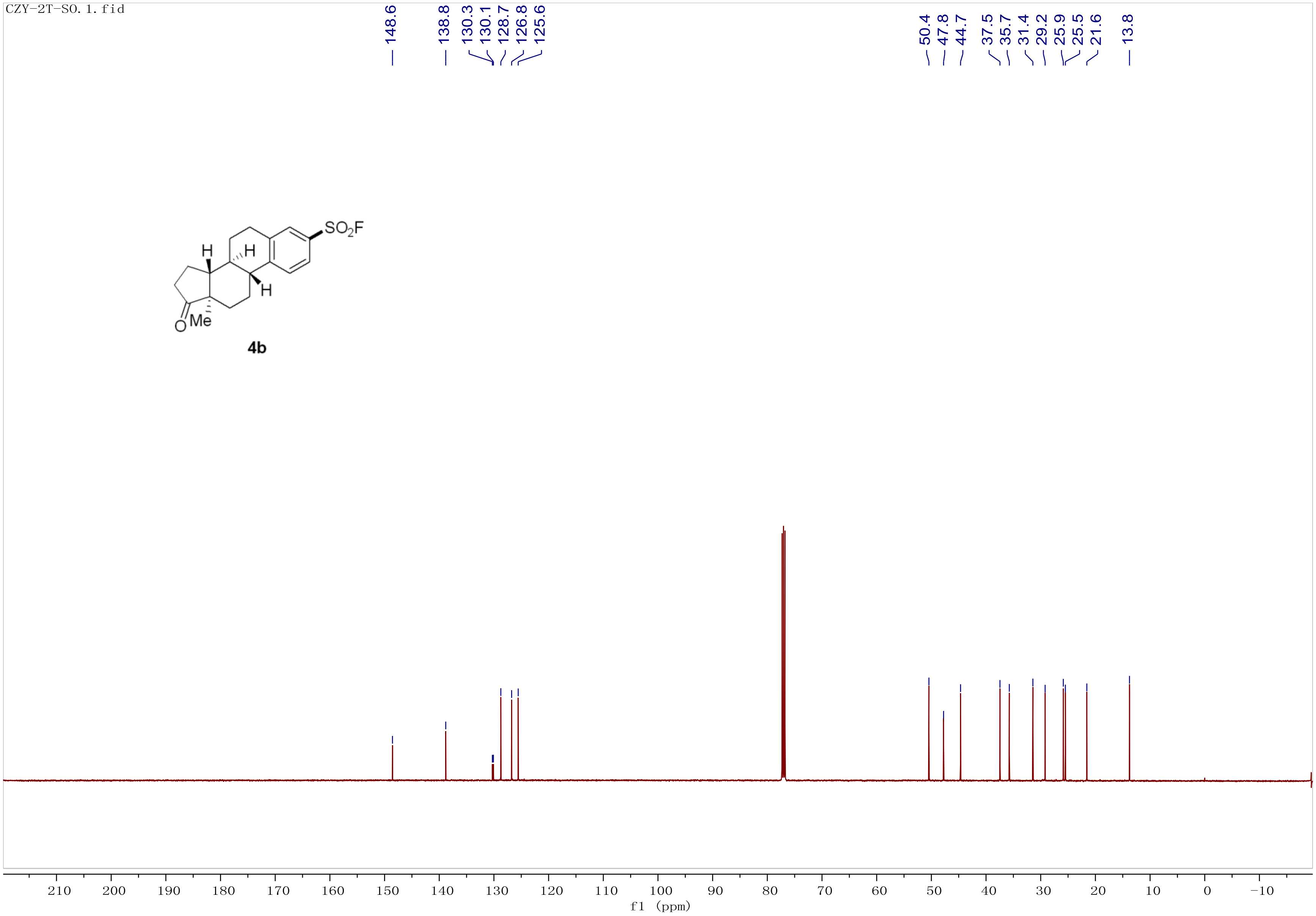

Supplementary Fig. 127 ^{13}C NMR spectrum of compound 4b ($CDCl_3$, 126 MHz, 298K)

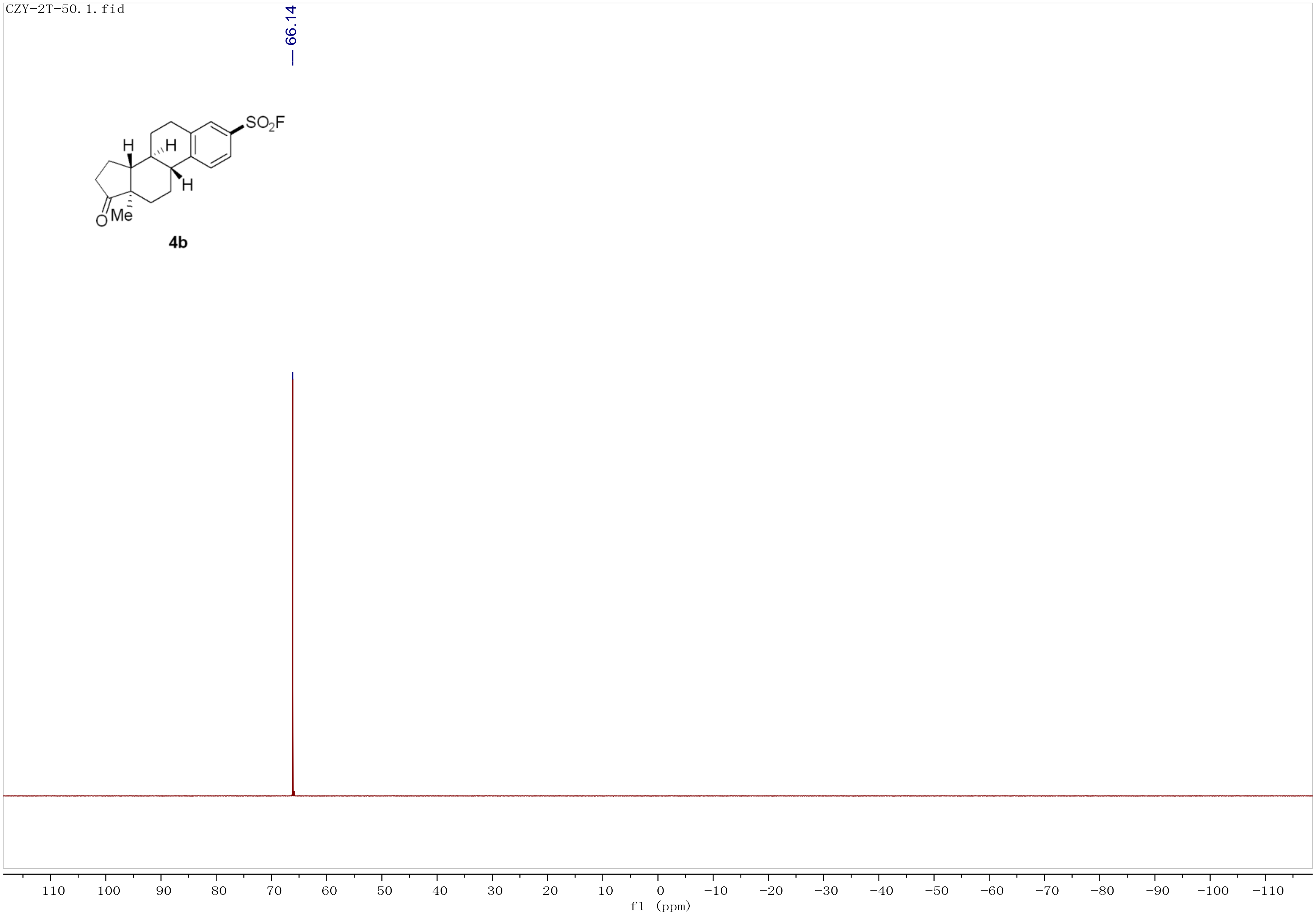

Supplementary Fig. 128 ^{19}F NMR spectrum of compound 4b ($CDCl_3$, 376 MHz, 298K)

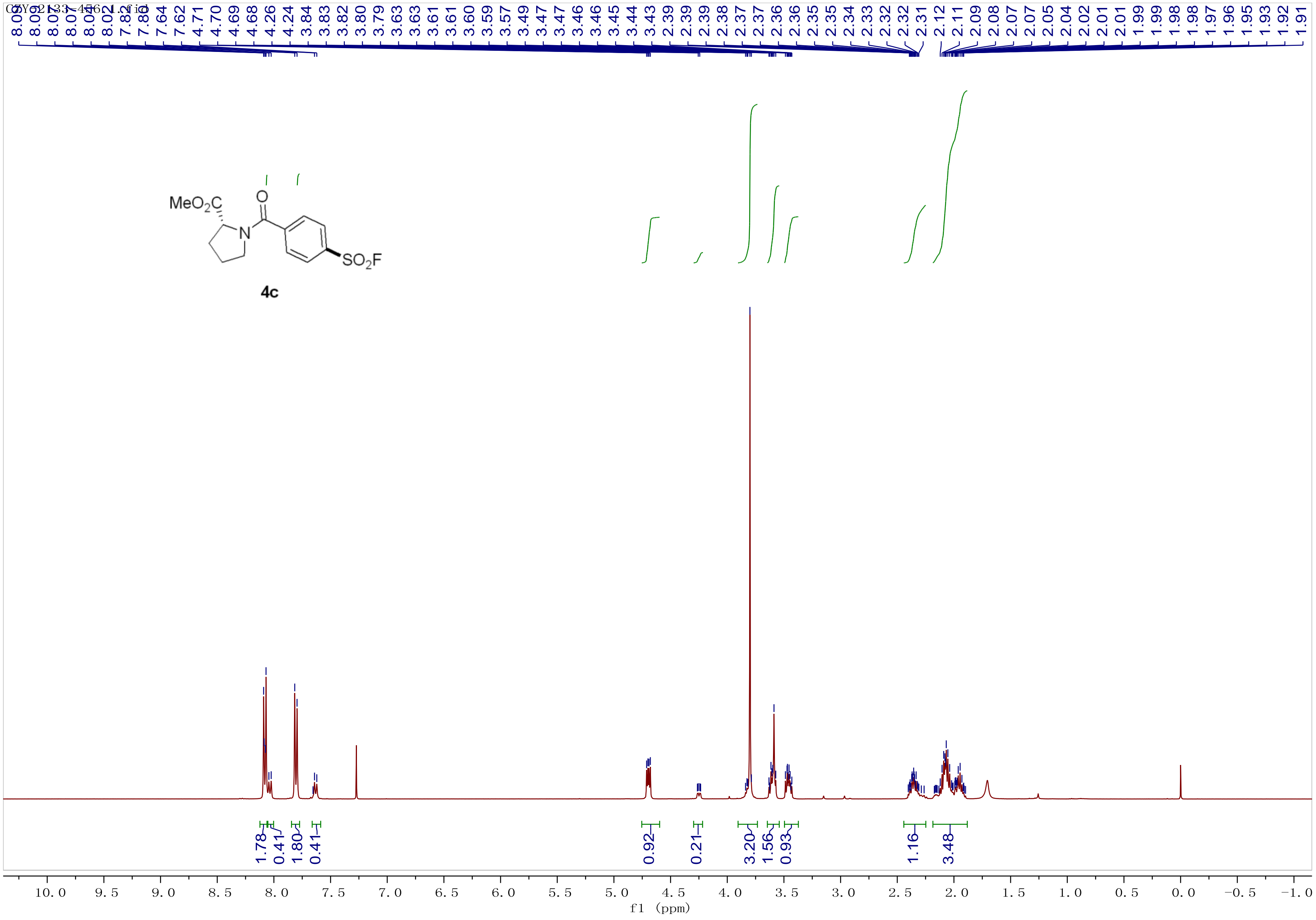

Supplementary Fig. 129 ^{1}H NMR spectrum of compound 4c ($CDCl_3$, 400 MHz, 298K)

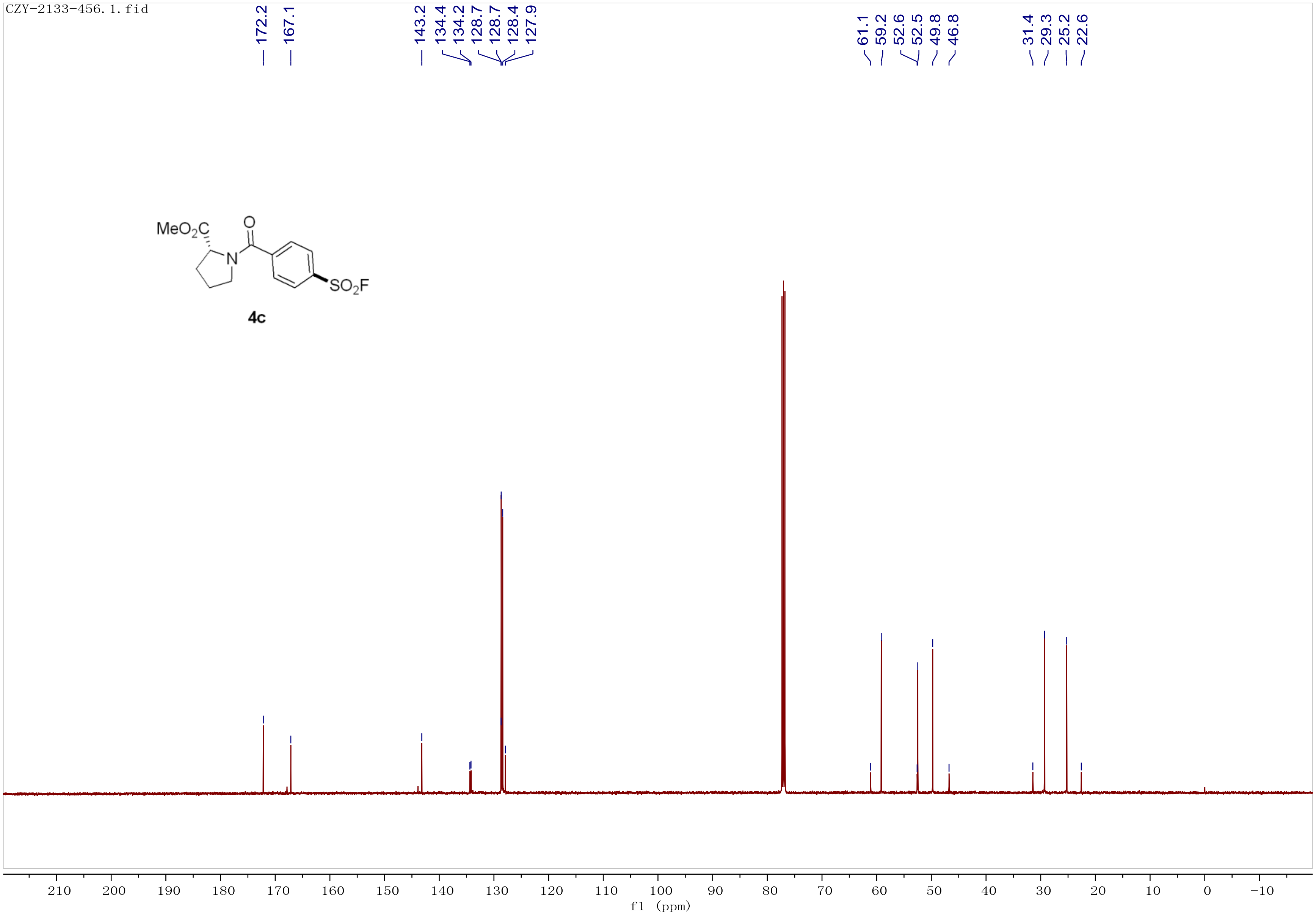

Supplementary Fig. 130 ^{13}C NMR spectrum of compound 4c ($CDCl_3$, 126 MHz, 298K)

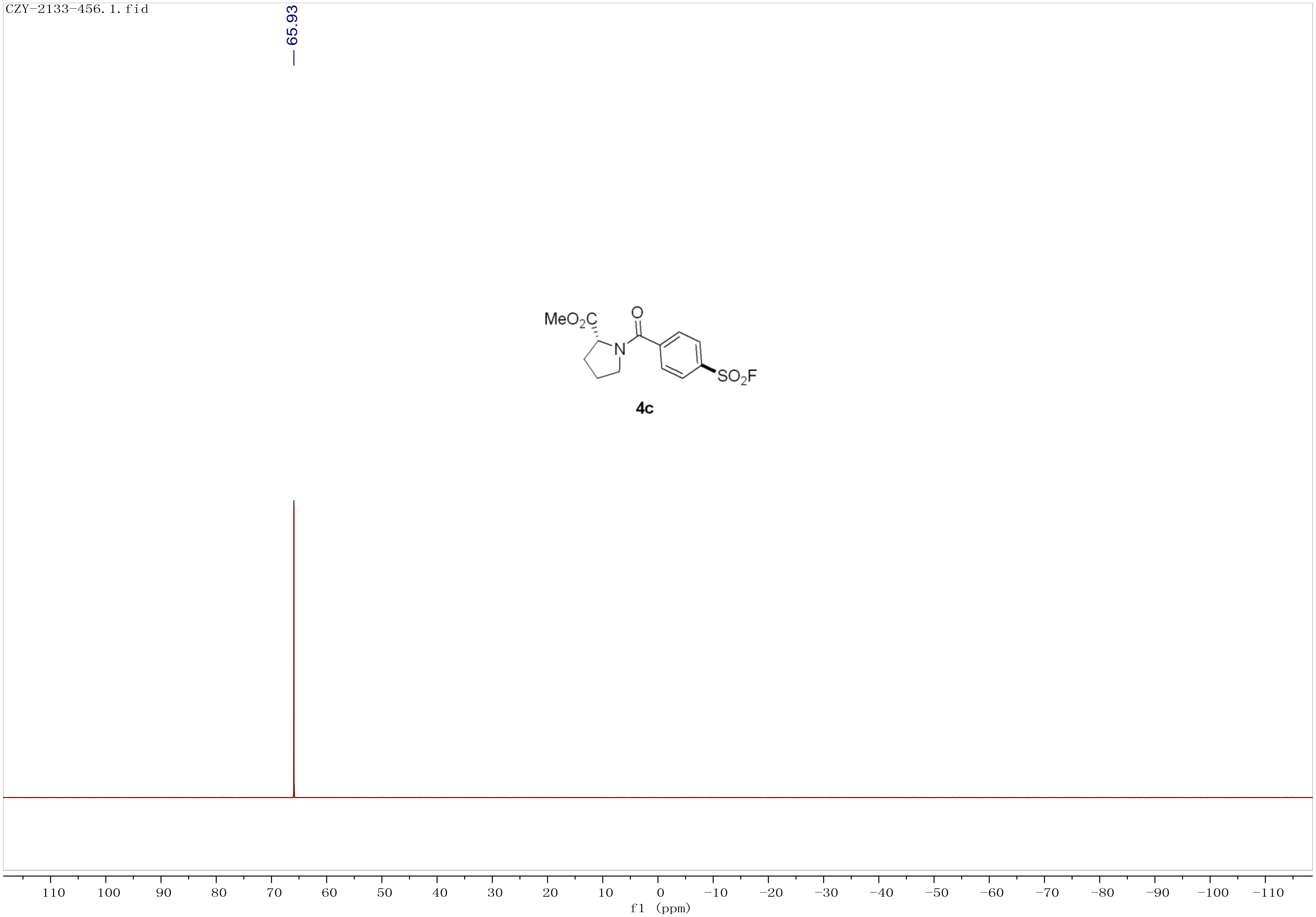

Supplementary Fig. 131 ^{19}F NMR spectrum of compound 4c ($CDCl_3$, 376 MHz, 298K)

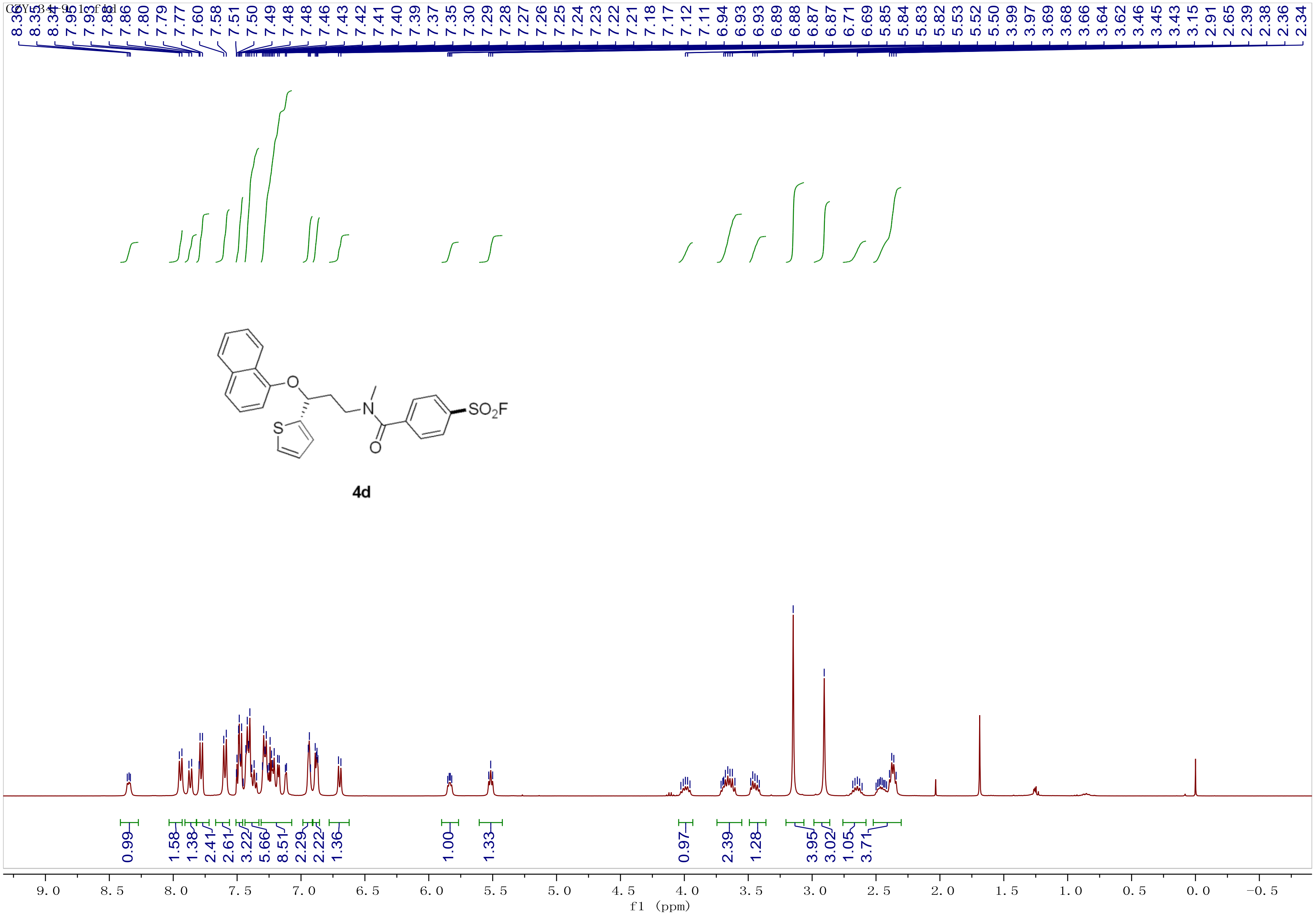

Supplementary Fig. 132 ^{1}H NMR spectrum of compound 4d ($CDCl_3$, 400 MHz, 298K)

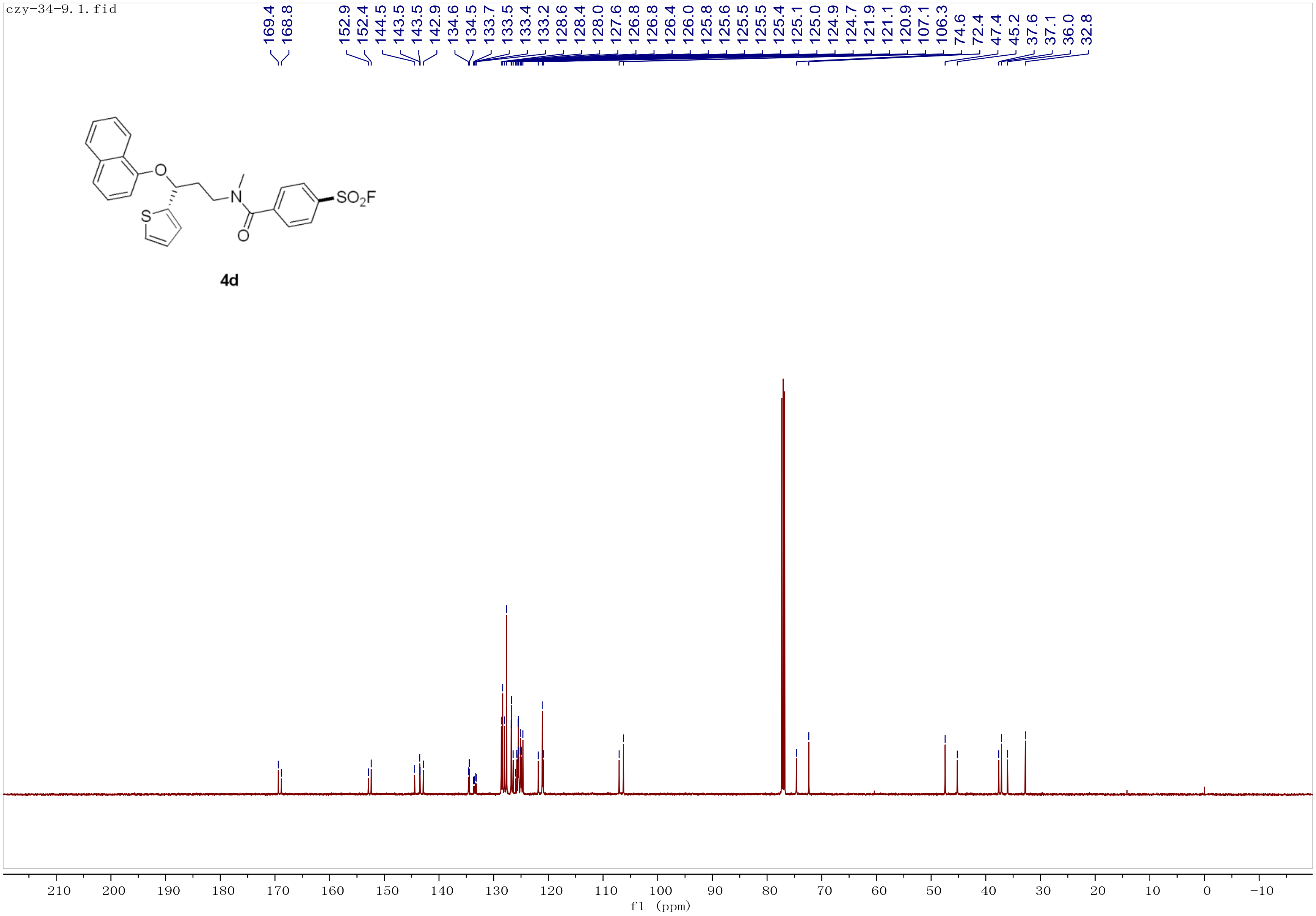

Supplementary Fig. 133 ^{13}C NMR spectrum of compound 4d ($CDCl_3$, 126 MHz, 298K)

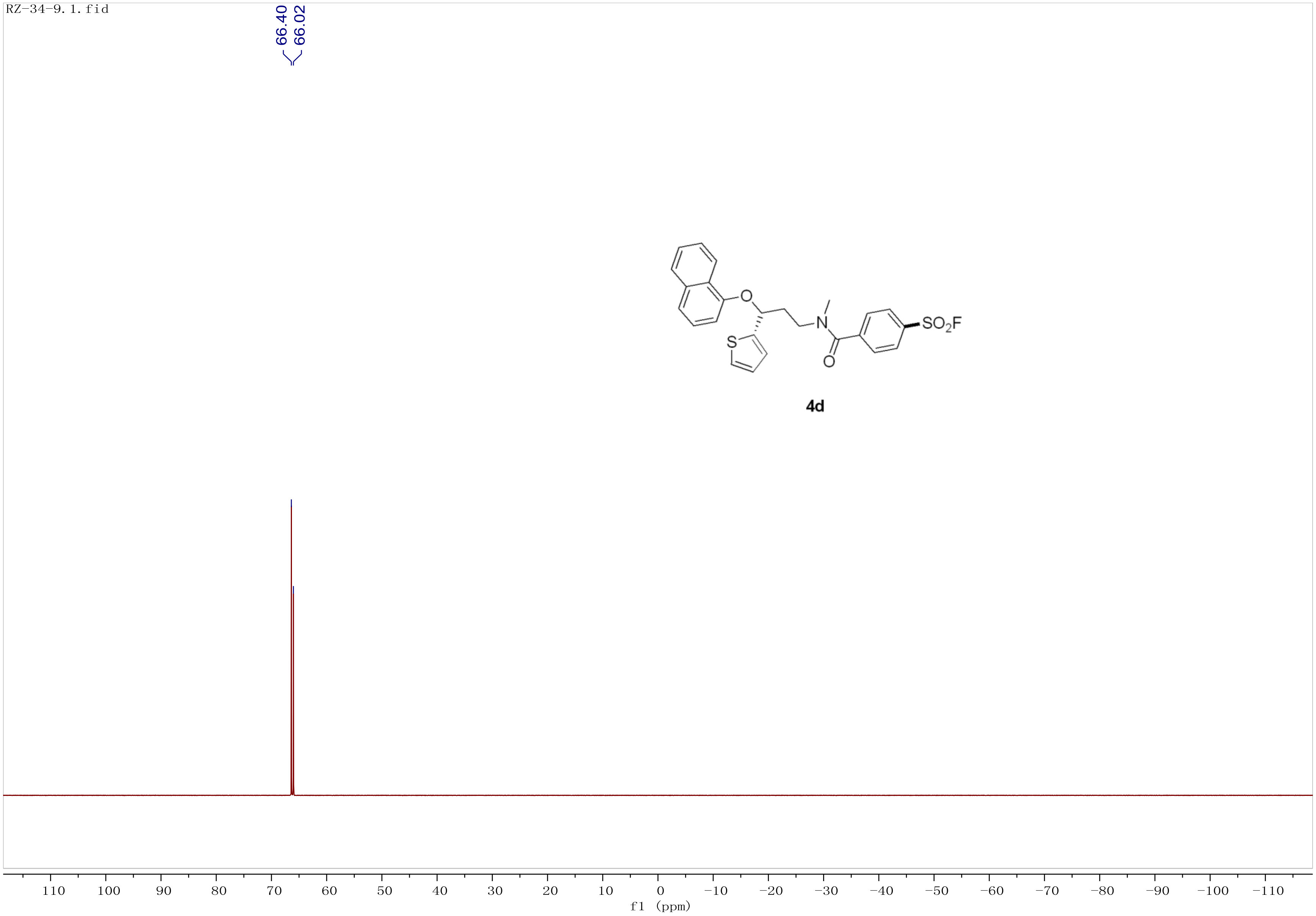

Supplementary Fig. 134 ^{19}F NMR spectrum of compound 4d ($CDCl_3$, 376 MHz, 298K)

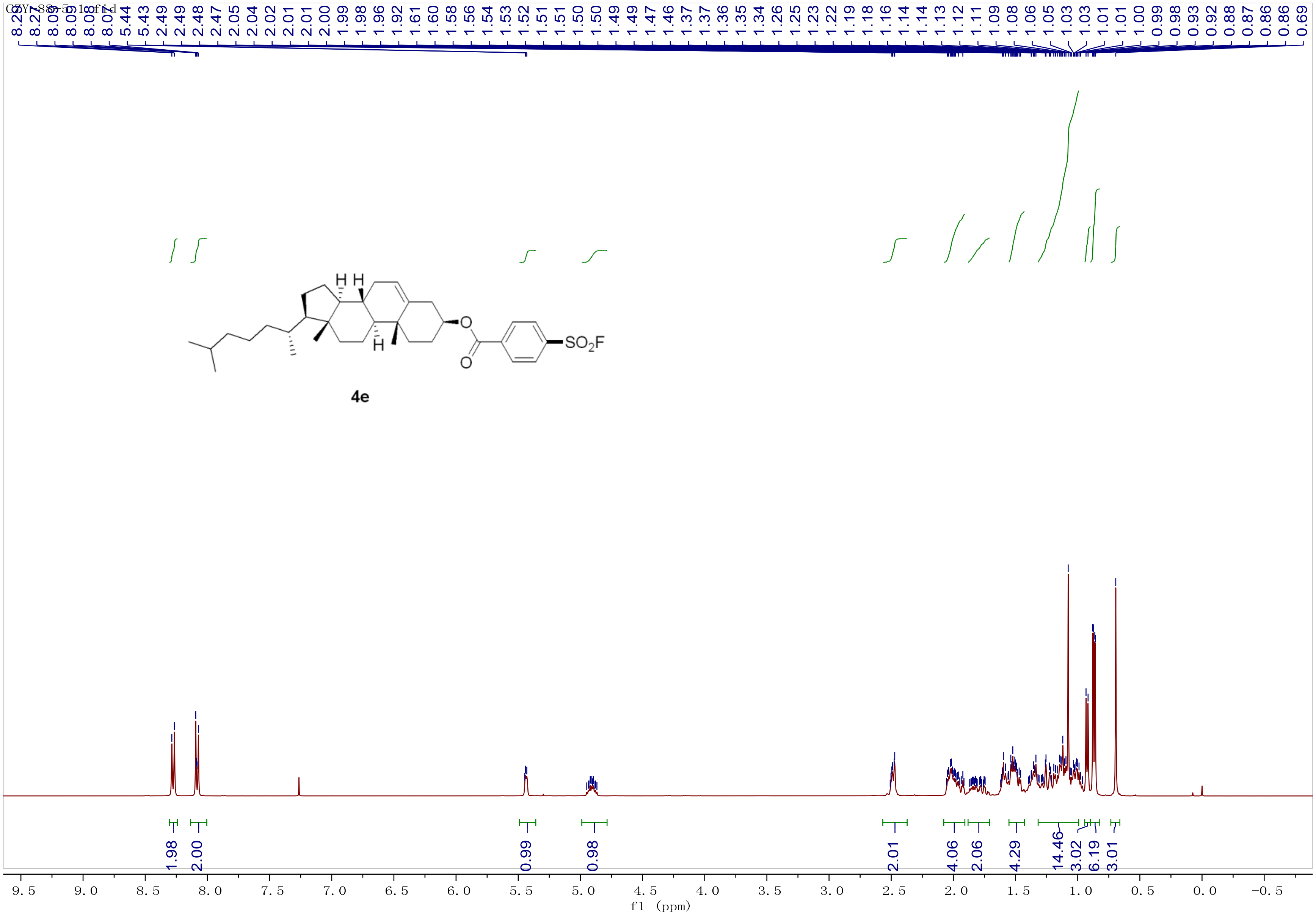

Supplementary Fig. 135 ^{1}H NMR spectrum of compound 4e ($CDCl_3$, 400 MHz, 298K)

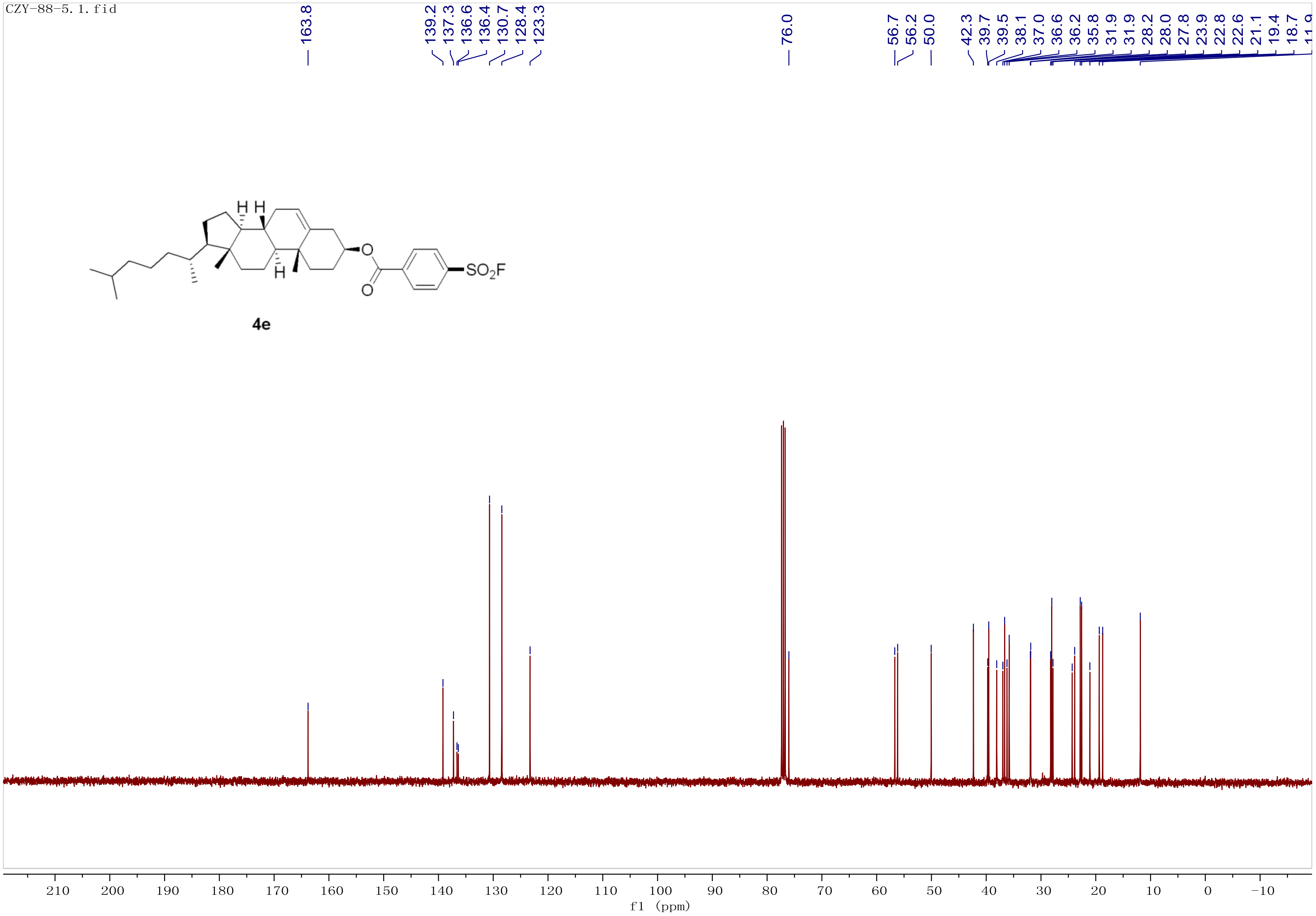

Supplementary Fig. 136 ^{13}C NMR spectrum of compound 4e ($CDCl_3$, 101 MHz, 298K)

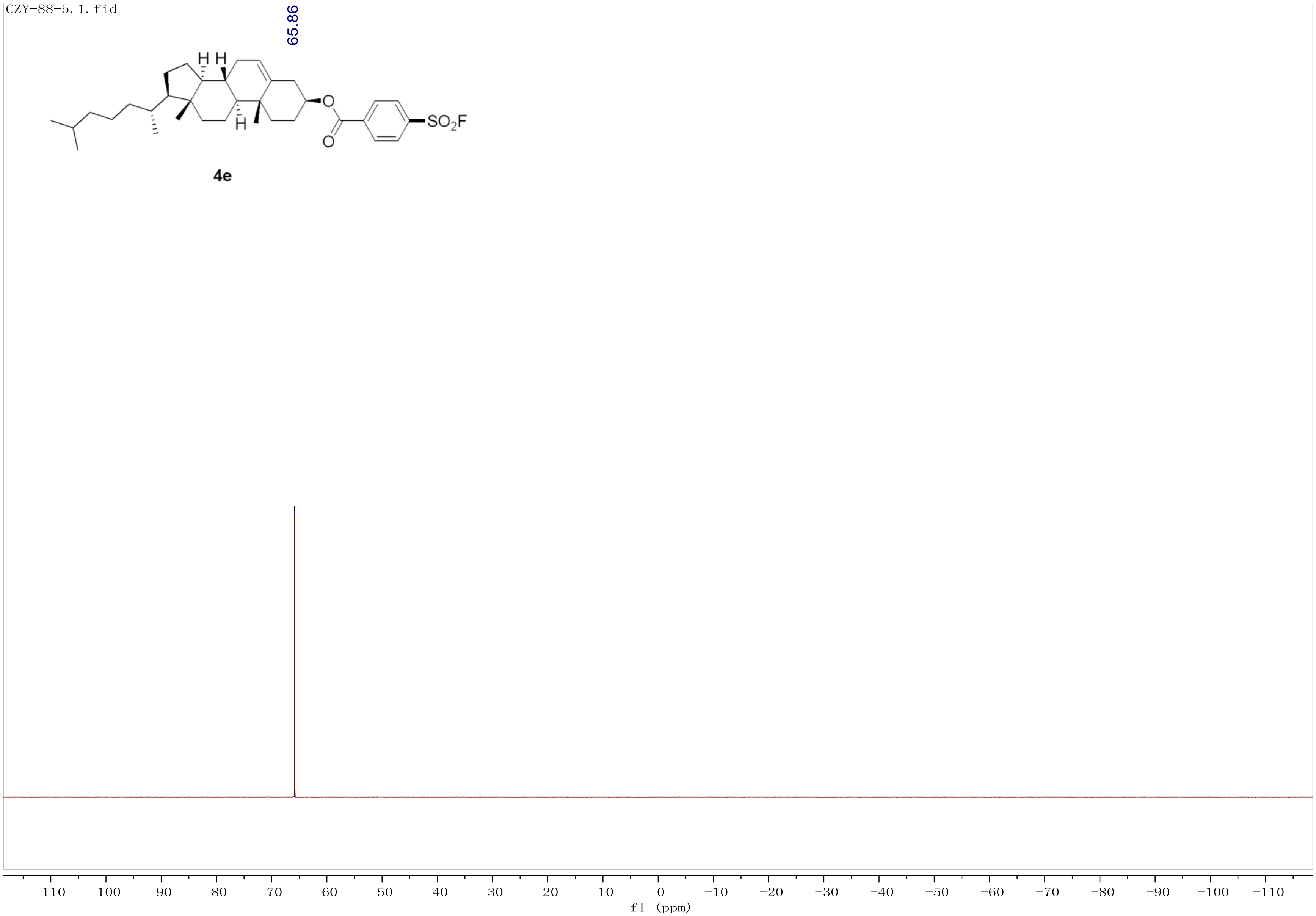

Supplementary Fig. 137 ^{19}F NMR spectrum of compound 4e ($CDCl_3$, 376 MHz, 298K)

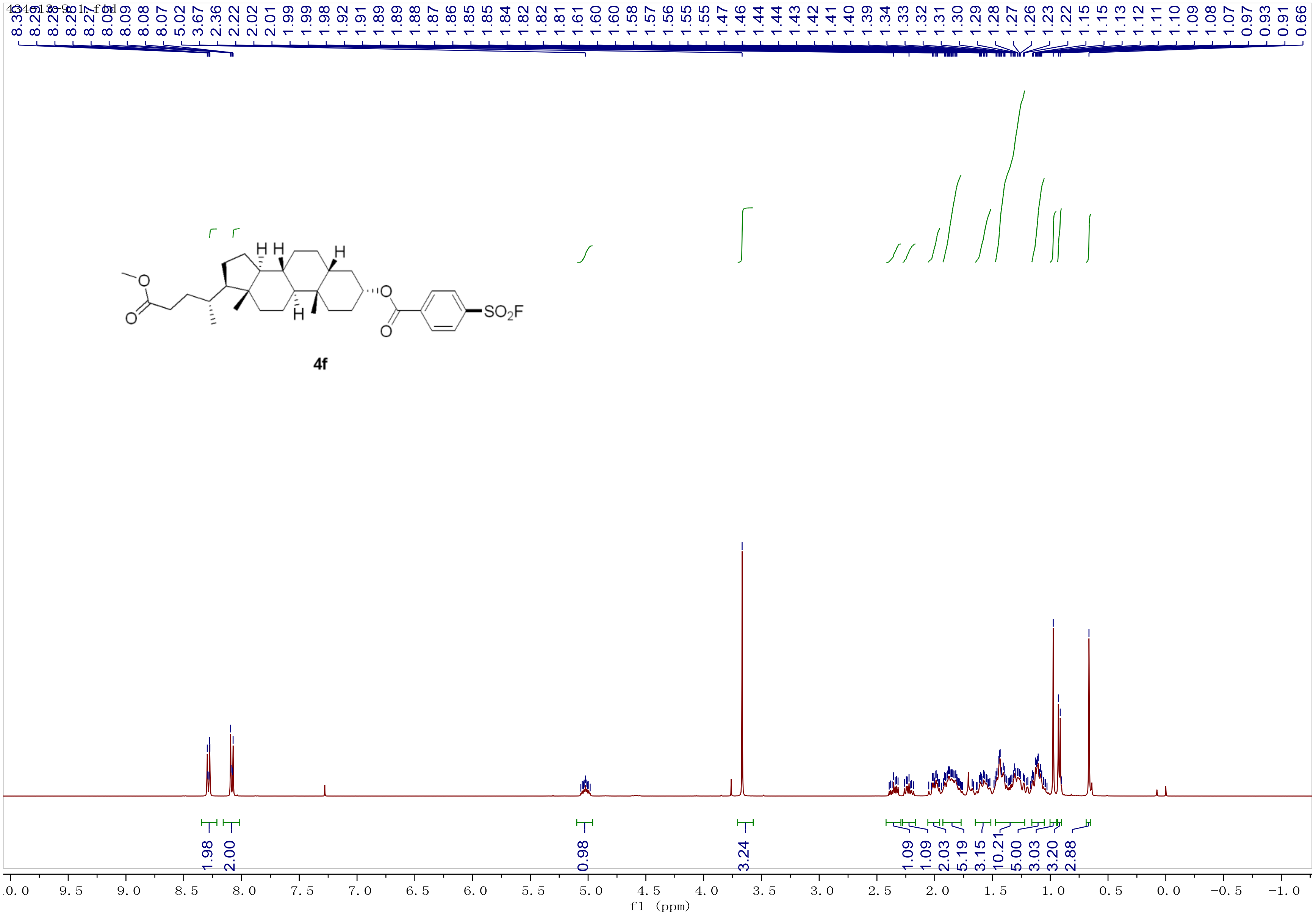

Supplementary Fig. 138 ^{1}H NMR spectrum of compound 4f ($CDCl_3$, 400 MHz, 298K)

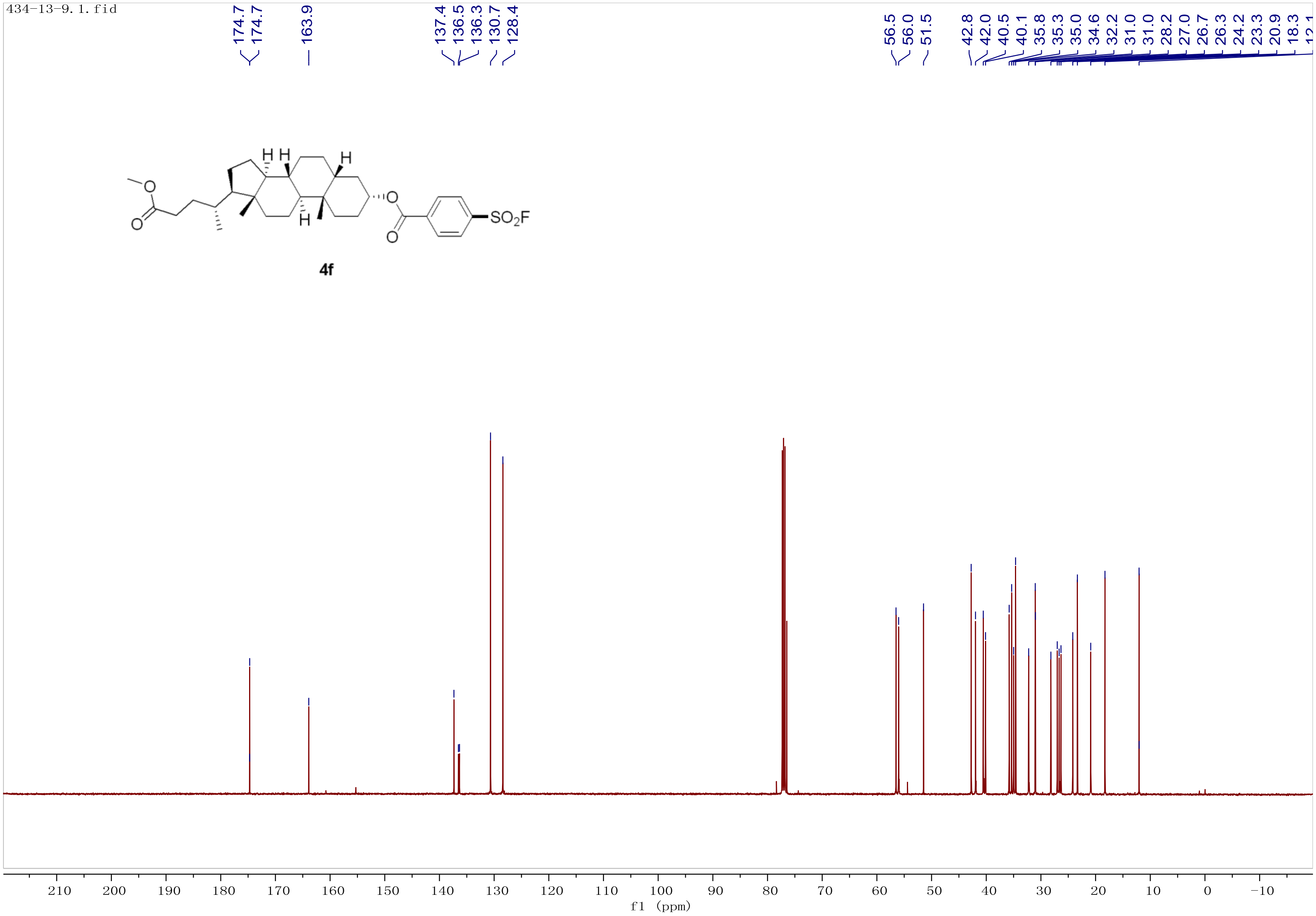

Supplementary Fig. 139 ^{13}C NMR spectrum of compound 4f ($CDCl_3$, 126 MHz, 298K)

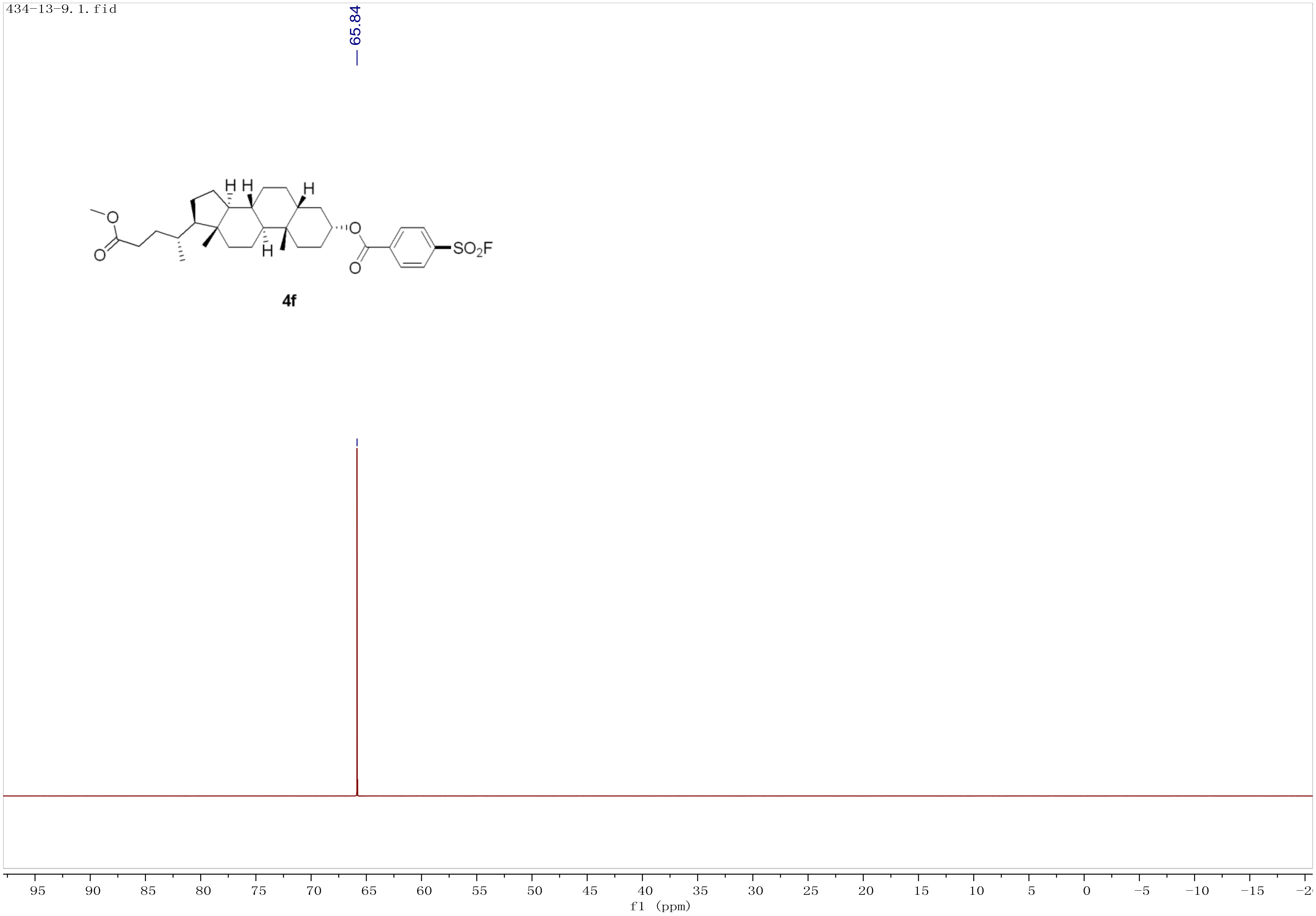

Supplementary Fig. 140 ^{19}F NMR spectrum of compound 4f ($CDCl_3$, 376 MHz, 298K)

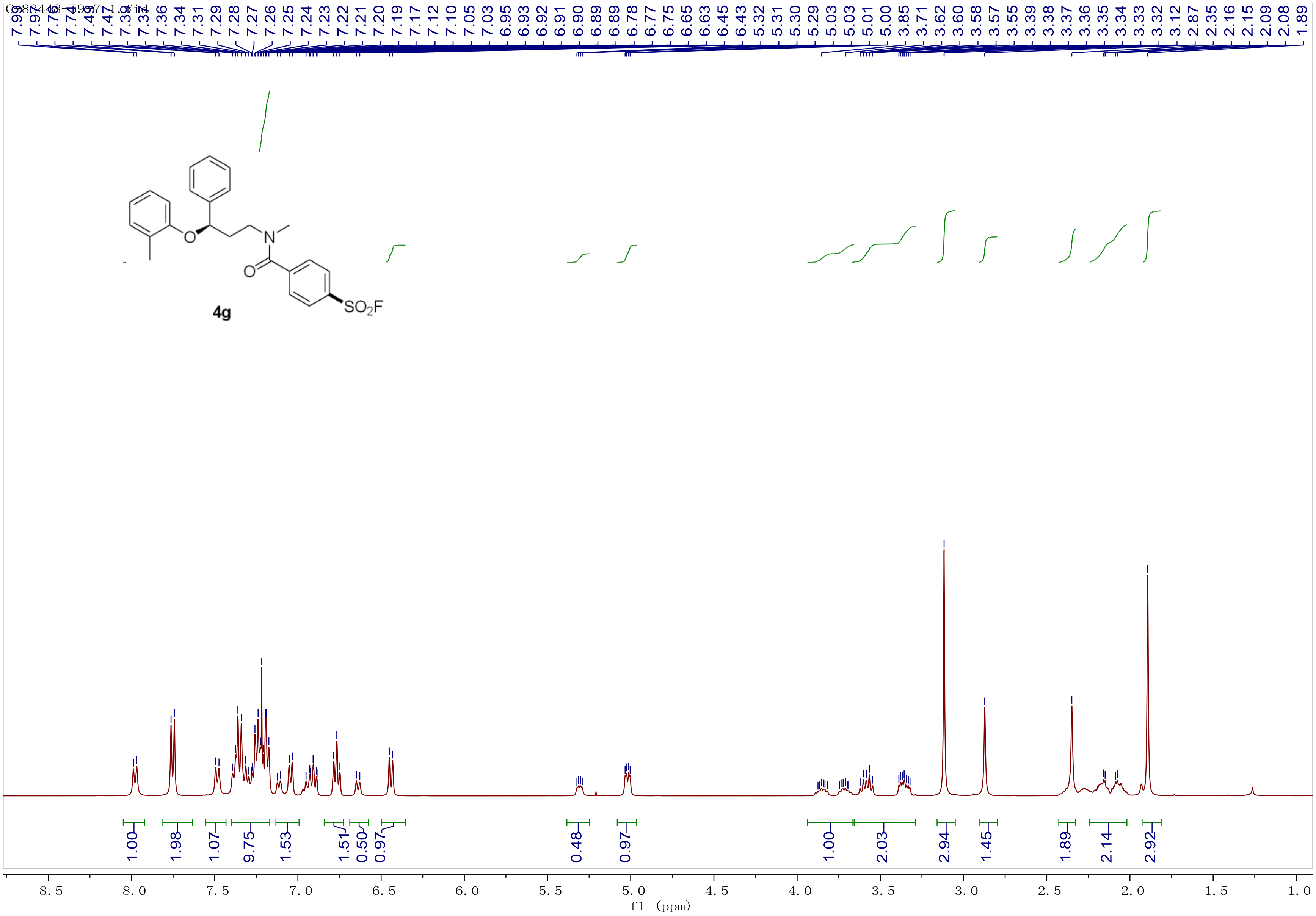

Supplementary Fig. 141 ¹H NMR spectrum of compound 4g ($CDCl_3$, 400 MHz, 298K)

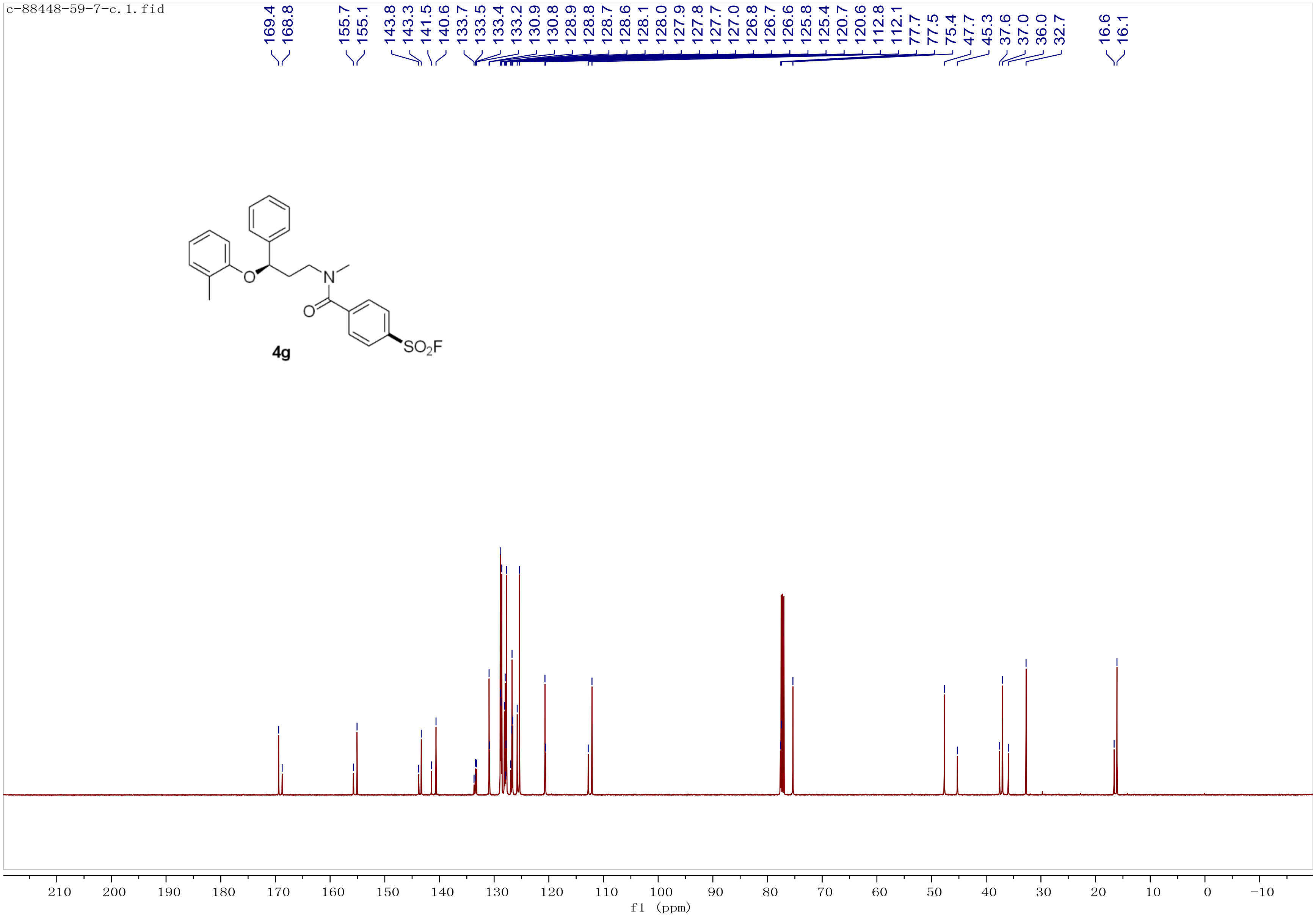

Supplementary Fig. 142 ^{13}C NMR spectrum of compound 4g ($CDCl_3$, 126 MHz, 298K)

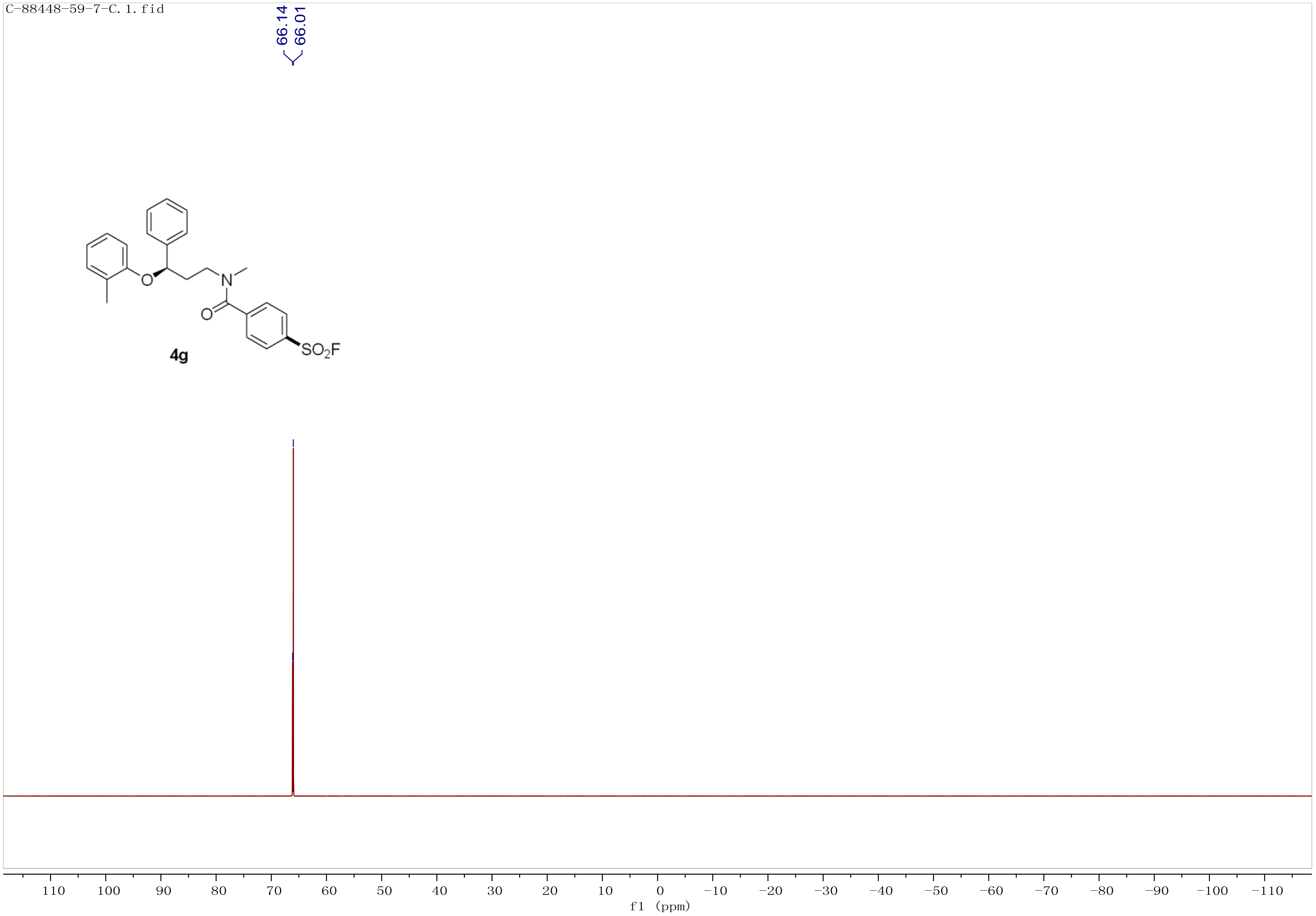

Supplementary Fig. 143 ^{19}F NMR spectrum of compound 4g ($CDCl_3$, 376 MHz, 298K)

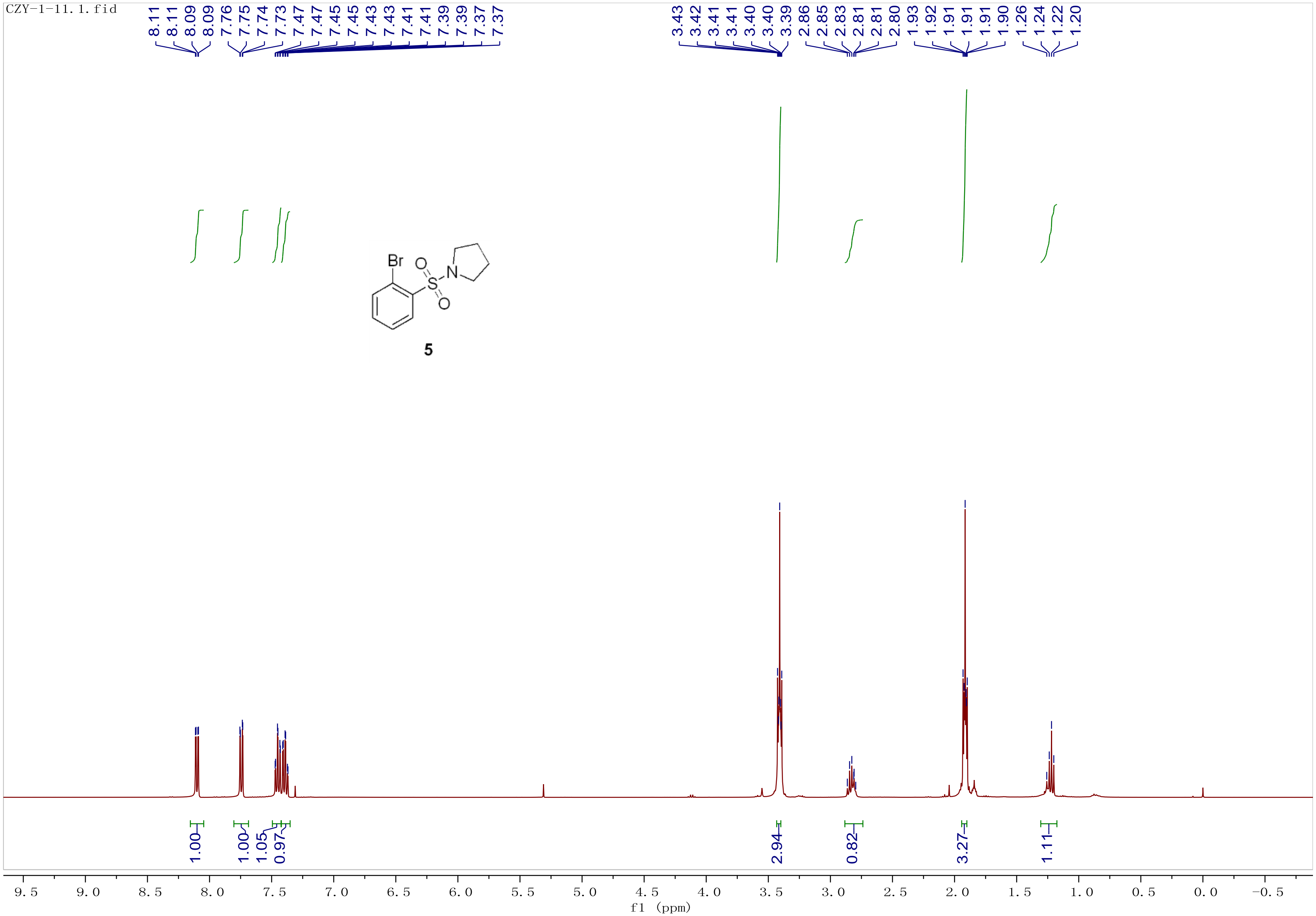

Supplementary Fig. 144 ^{1}H NMR spectrum of compound 5 ($CDCl_3$, 471 MHz, 298K)

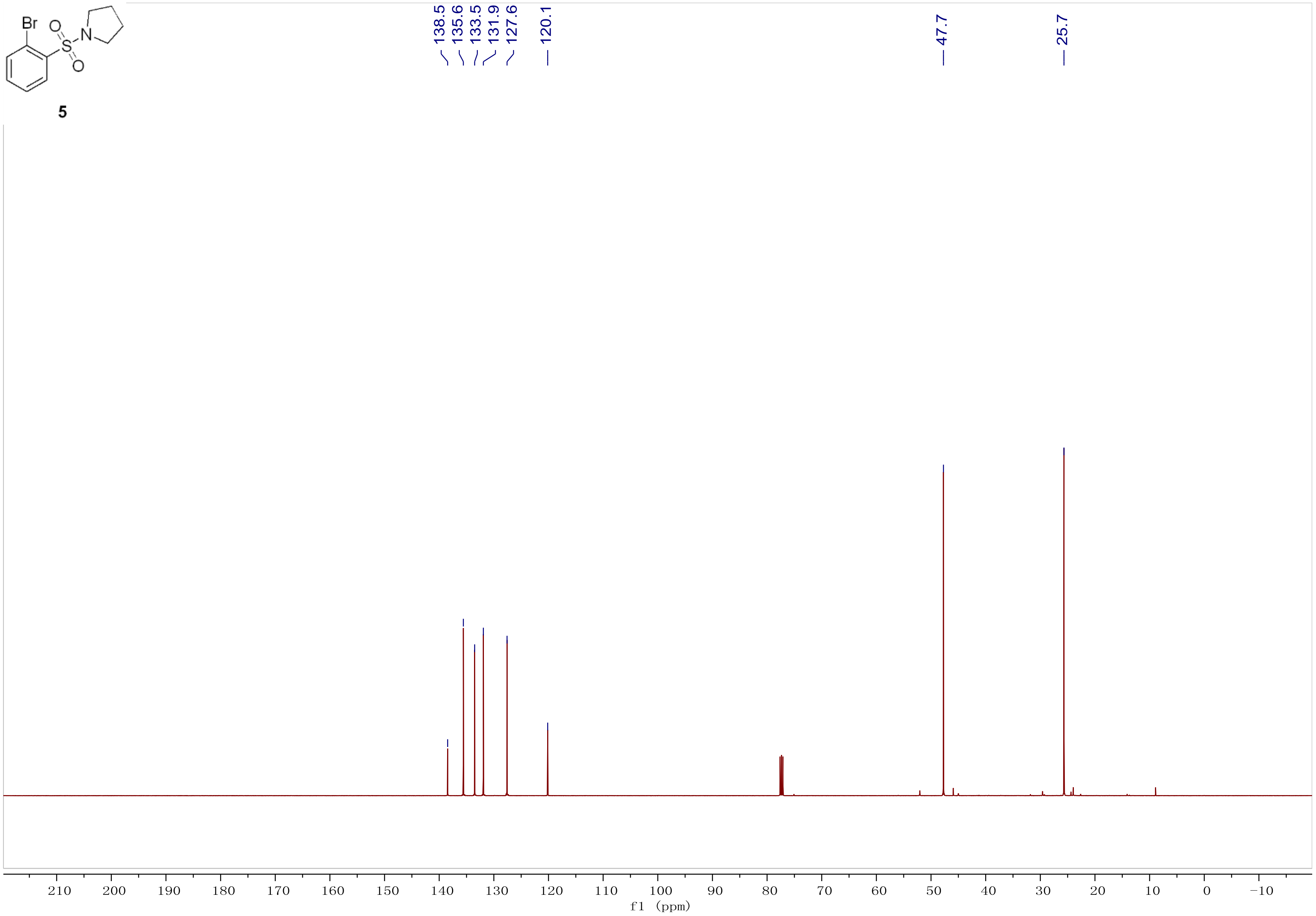

Supplementary Fig. 145 ^{1}H NMR spectrum of compound 5 ($CDCl_3$, 400 MHz, 298K)

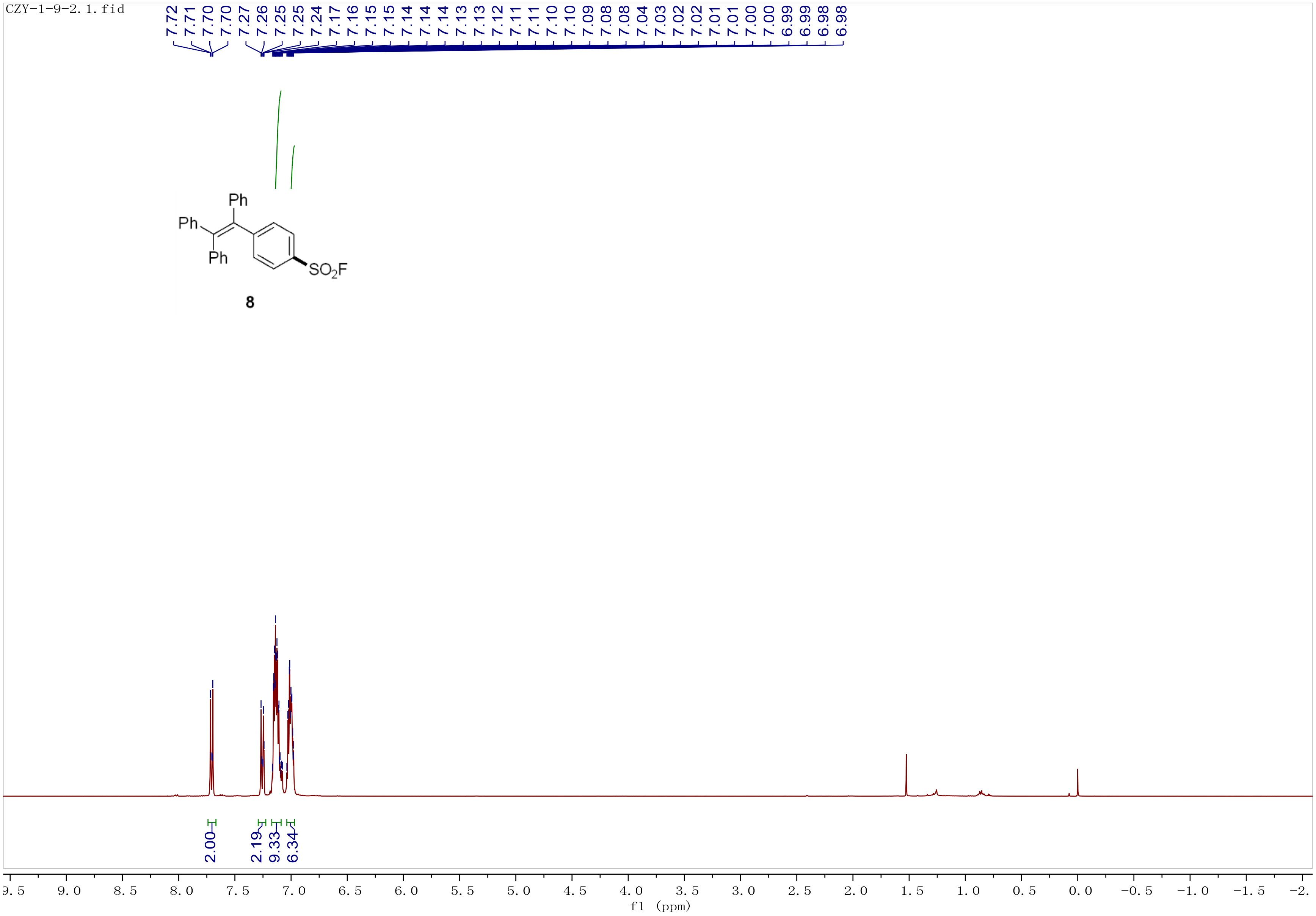

Supplementary Fig. 146 ^{13}C NMR spectrum of compound 8 ($CDCl_3$, 126 MHz, 298K)

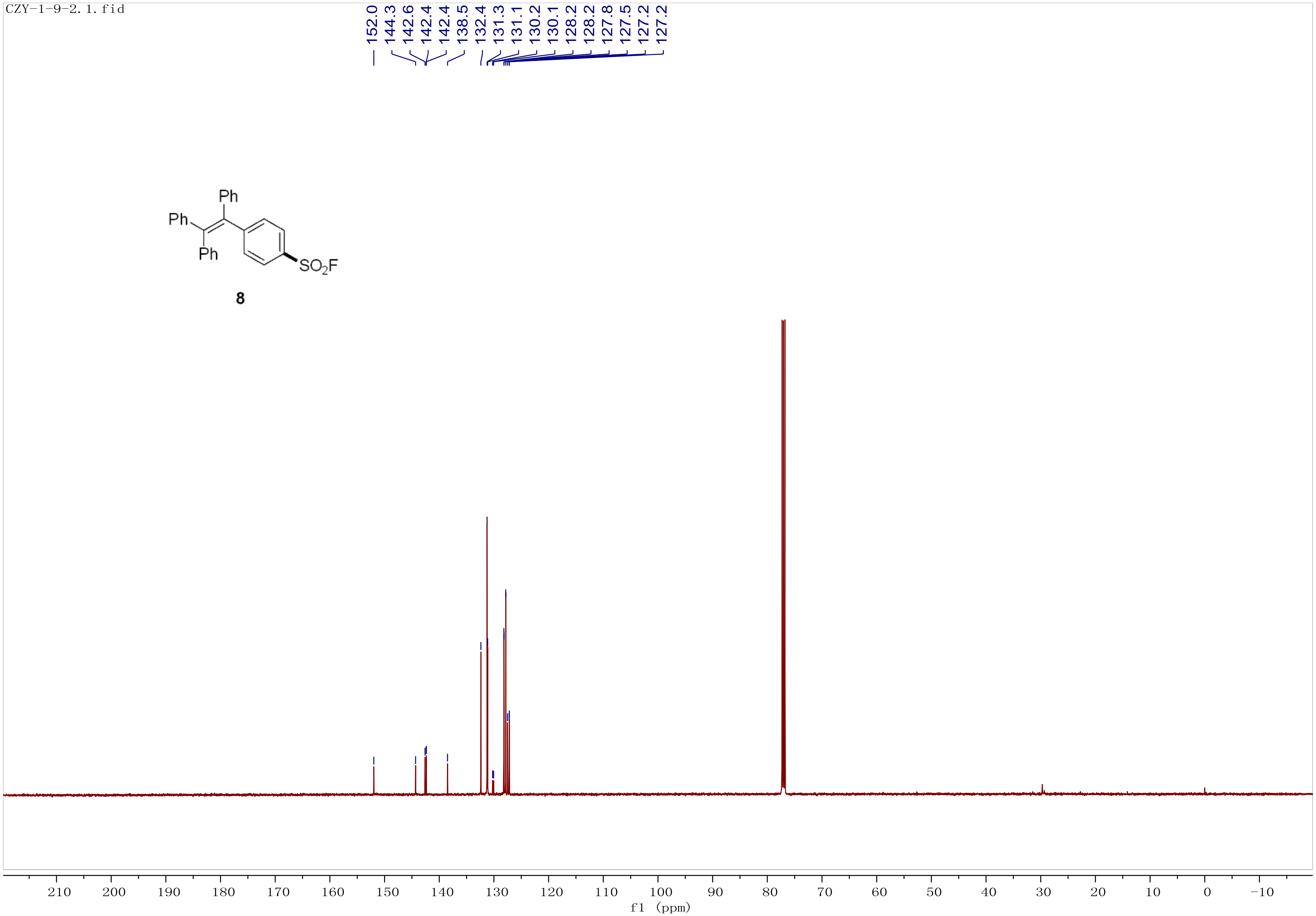

Supplementary Fig. 147 ^{13}C NMR spectrum of compound 8 ($CDCl_3$, 126 MHz, 298K)

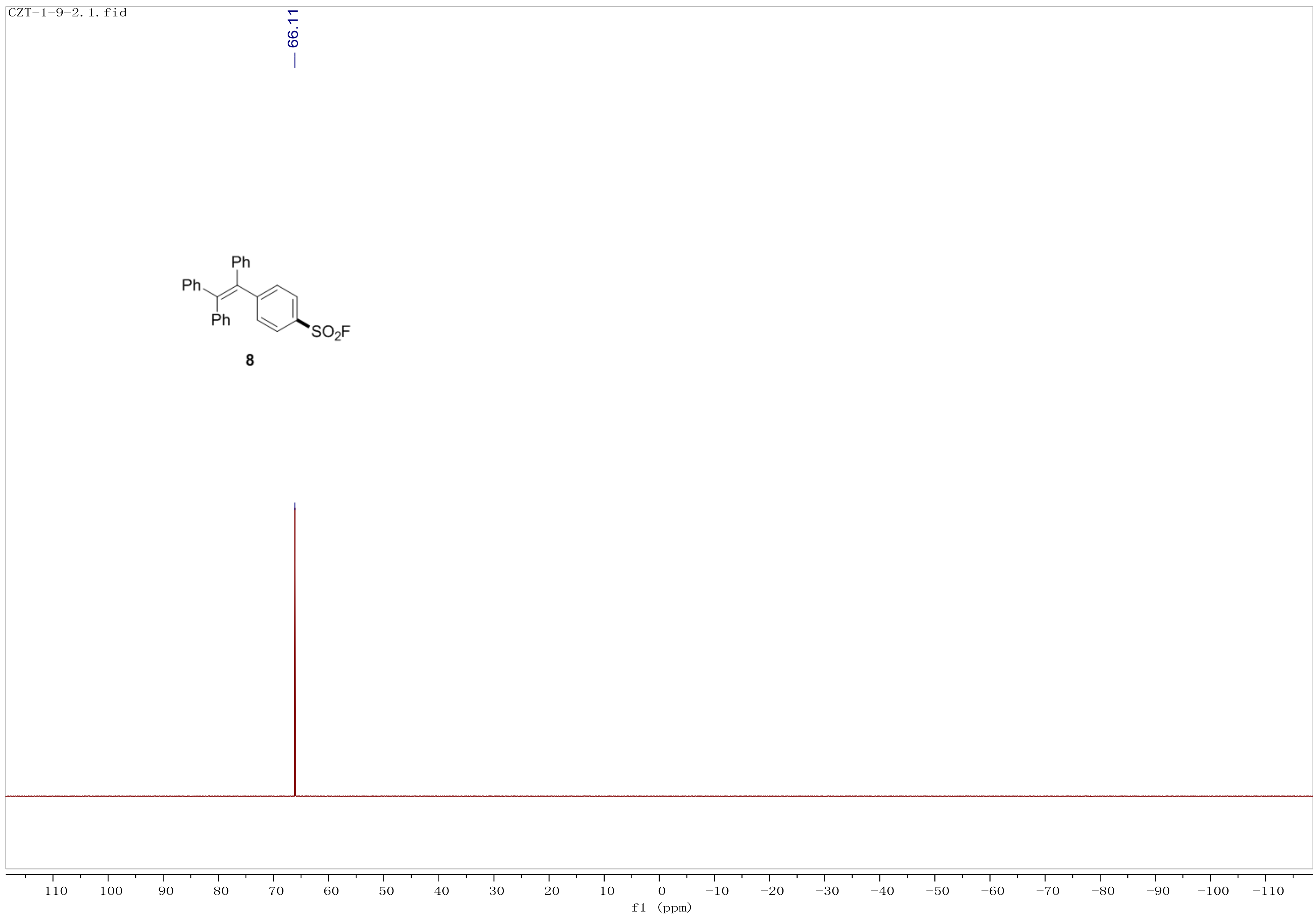

Supplementary Fig. 148 ^{19}F NMR spectrum of compound 8 ($CDCl_3$, 376 MHz, 298K)

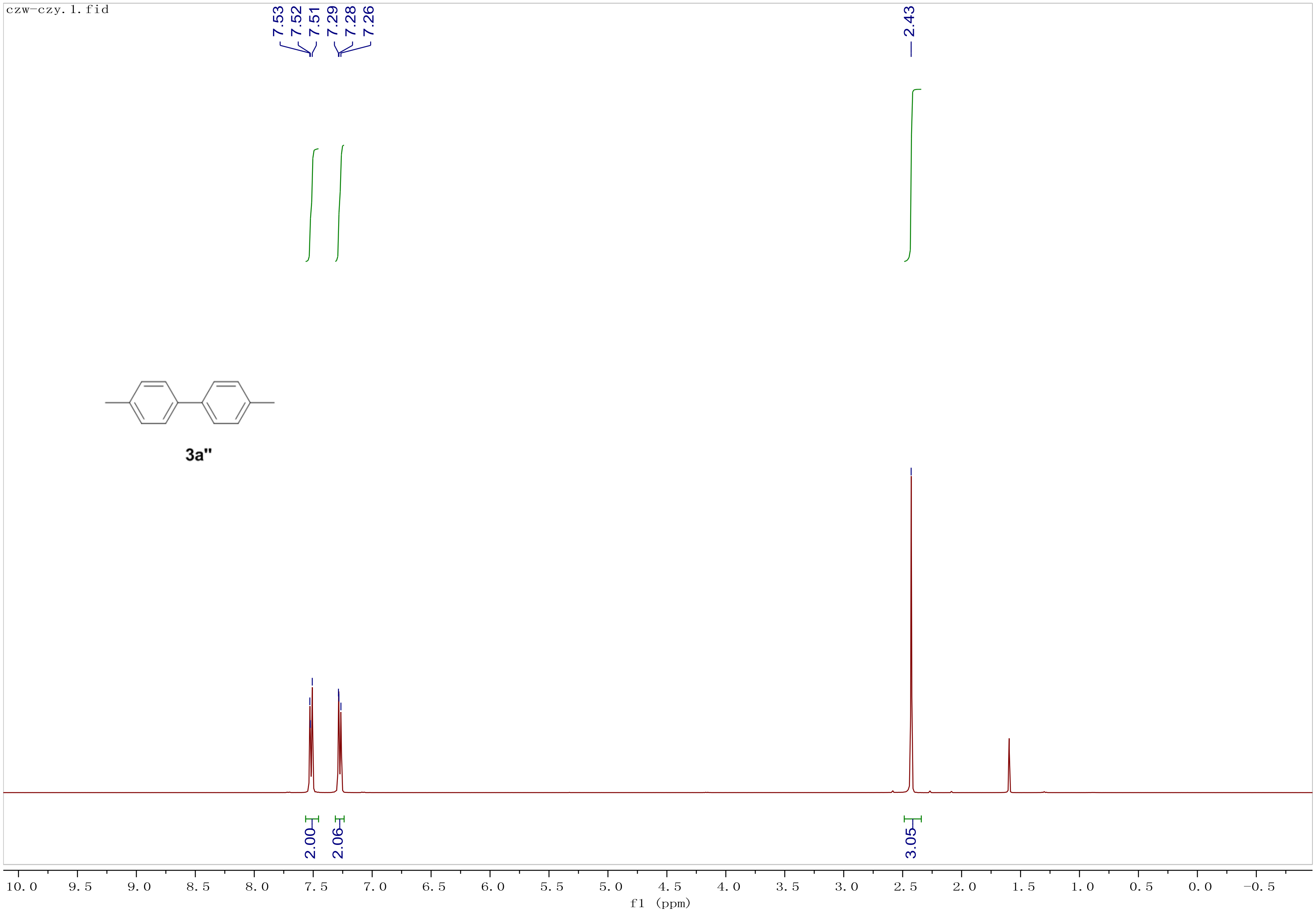

Supplementary Fig. 149 ^{1}H NMR spectrum of compound 3a'' ($CDCl_3$, 400 MHz, 298K)

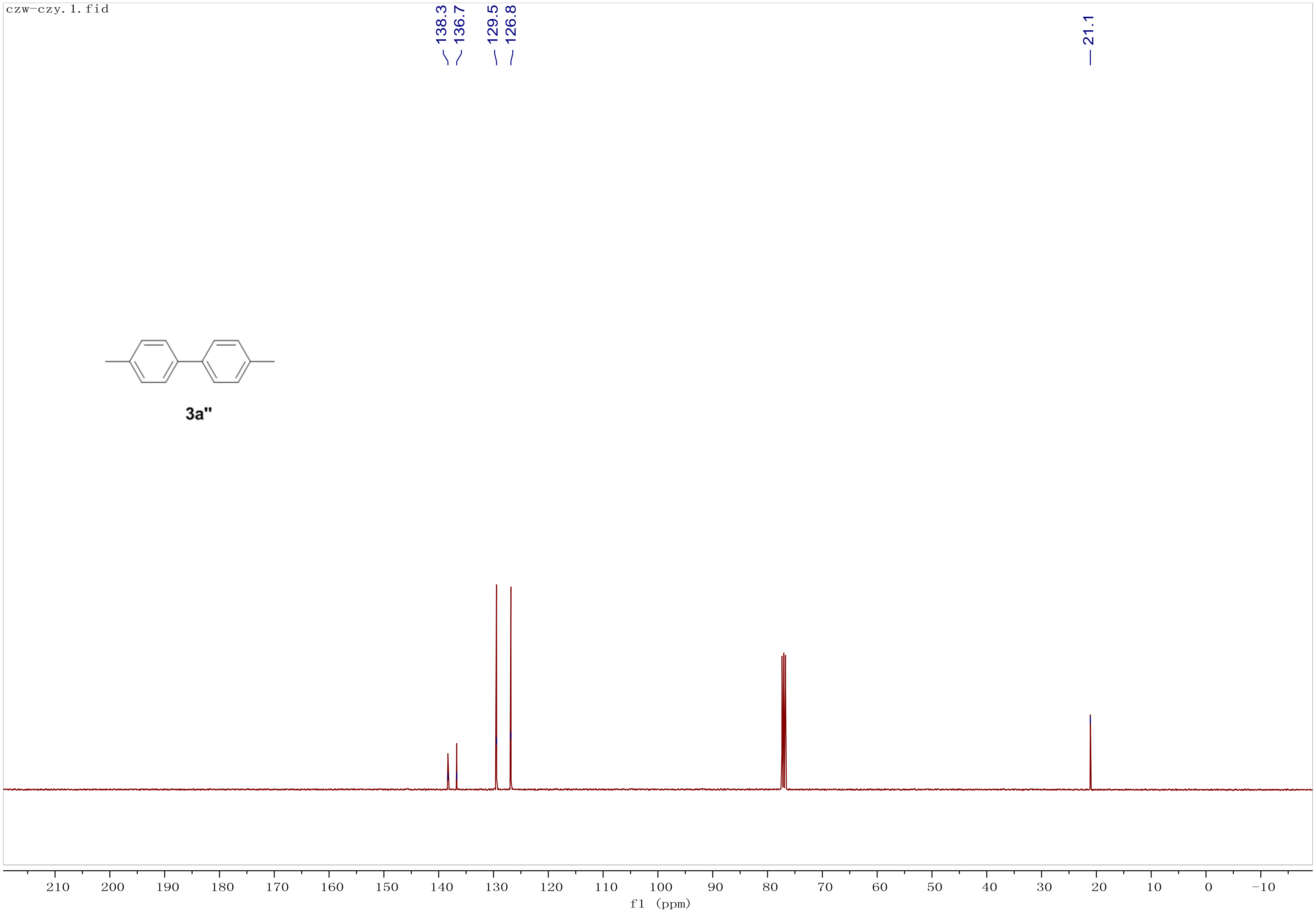

Supplementary Fig. 150 ^{13}C NMR spectrum of compound 3a'' ($CDCl_3$, 101 MHz, 298K)

12. References

1. Liu, W., Yang, X., Gao, Y., & Li, C.-J. Simple and efficient generation of aryl radicals from aryl triflates: Synthesis of aryl boronates and aryl iodides at room temperature. *J. Am. Chem. Soc.* **139**, 8621–8627 (2017).
2. Dou, Q., Geng, L., Cheng, B., Li, C.-J., & Zeng, H. Photoinduced transition-metal and external photosensitizer free cross-coupling of aryl triflates with trialkyl phosphites. *Chem. Commun.* **57**, 8429–8432 (2021).
3. Davies, A. T., Curto, J. M., Bagley, S. W., & Willis, M. C. One-pot palladium-catalyzed synthesis of sulfonyl fluorides from aryl bromides. *Chem. Sci.* **8**, 1233–1237 (2017).
4. Tribby, A. L., Rodríguez, I., Shariffudin, S., & Ball, N. D. Pd-catalyzed conversion of aryl iodides to sulfonyl fluorides using SO_2 surrogate DABSO and Selectfluor. *J. Org. Chem.* **82**, 2294–2299 (2017).
5. Lou, T. S-B., Bagley, S. W., & Willis, M. C. Cyclic Alkenylsulfonyl fluorides: Palladium-catalyzed synthesis and functionalization of compact multifunctional reagents. *Angew. Chem. Int. Ed.* **58**, 18859–18863 (2019).
6. Laudadio, G., Bartolomeu, A. A., Verwijlen, L. M. H. M., Cao, Y., Oliveira, K. T., & Noël, T. Sulfonyl fluoride synthesis through electrochemical oxidative coupling of thiols and potassium fluoride. *J. Am. Chem. Soc.* **141**, 11832–11836 (2019).
7. Tang, L., Yang, Y., Wen, L., Yang, X., & Wang, Z. Catalyst-free radical fluorination of sulfonyl hydrazides in water. *Green Chem.* **18**, 1224–1228 (2016).
8. Lo, P. K. T., Chen, Y., & Willis, M. C. Nickel(II)-catalyzed synthesis of sulfinates from aryl and heteroaryl boronic acids and the sulfur dioxide surrogate DABSO. *ACS Catal.* **9**, 10668–10673 (2019).
9. Liu, Y., Yu, D., Guo, Y., Xiao, J.-C., Chen, Q.-Y., & Liu, C. Arenesulfonyl fluoride synthesis via copper-catalyzed fluorosulfonylation of arenediazonium salts. *Org. Lett.* **22**, 2281–2286 (2020).
10. Zhong, T., Pang, M-K., Chen, Z.-D., Zhang, B., Weng, J., & Lu, G. Copper-free Sandmeyer-type reaction for the synthesis of sulfonyl fluorides. *Org. Lett.* **22**, 3072–3078 (2020).
11. Li, J.-T., Zhou, X., Chen, Q.-L., Chen, Z.-D., Lu, G., & Weng, J. Copper-catalyzed direct decarboxylative fluorosulfonylation of aliphatic carboxylic acids. *Chem. Commun.* **58**, 9409–9412 (2022).

12. Ma, Z., Liu, Y., Ma, X., Hu, X., Guo, Y., Chen, Q.-Y., & Liu, C. Aliphatic sulfonyl fluoride synthesis via reductive decarboxylative fluorosulfonylation of aliphatic carboxylic acid NHPI esters. *Org. Chem. Front.* **9**, 1115–1120 (2022).

13. Jutand, A., & Négri, S. Activation of aryl and vinyl triflates by palladium and electron transfer – electrosynthesis of aromatic and α,β-unsaturated carboxylic acids from carbon dioxide. *Eur. J. Org. Chem.* **9**, 1811–1821 (1998).

14. Liu, W., Li, J., Querard, P., & Li, C.-J. Transition-metal-free C–C, C–O, and C–N cross-couplings enabled by light. *J. Am. Chem. Soc.* **141**, 6755–6764 (2019).

15. Frisch, M. J. et al. Gaussian 16, Revision A.03, Gaussian, Inc., Wallingford CT, 2016.

16. Zhao, Y., & Truhlar, D. G. The M06 suite of density functionals for main group thermochemistry, thermochemical kinetics, noncovalent interactions, excited states, and transition elements: two new functionals and systematic testing of four M06-class functionals and 12 other functionals. *Theor. Chem. Acc.* **120**, 215–241 (2008).

17. Marenich, A. V., Cramer, C. J., & Truhlar, D. G. Universal solvation model based on solute electron density and on a continuum model of the solvent defined by the bulk dielectric constant and atomic surface tensions. *J. Phys. Chem. B.* **113**, 6378–6396 (2009).

18. Xiao, M., & Lu, T. J. Generalized charge decomposition analysis (GCDA) method. *Adv. Phys. Chem.* **4**, 111–124 (2015).

19. Lu, T., & Chen, F. Multiwfn: A multifunctional wavefunction analyzer. *J. Comput. Chem.* **33**, 580–592 (2012).
